# Spatial-temporal profiling of prodiginines and serratamolides produced by endophytic *Serratia marcescens* harbored in *Maytenus serrata*

**DOI:** 10.1038/s41598-018-23538-5

**Published:** 2018-03-27

**Authors:** Dennis Eckelmann, Michael Spiteller, Souvik Kusari

**Affiliations:** Institute of Environmental Research (INFU), Department of Chemistry and Chemical Biology, Chair of Environmental Chemistry and Analytical Chemistry, TU Dortmund, Otto-Hahn-Straße 6, 44221 Dortmund, Germany

## Abstract

An endophytic bacterium, *Serratia marcescens* MSRBB2, isolated from inner bark of a Cameroonian *Maytenus serrata* plant, was subjected to the OSMAC (*O*ne *S*train *Ma*ny *C*ompounds) approach and metabolic profiling using HPLC-HRMS^n^. We identified 7 prodiginines along with 26 serratamolides. Their biosynthetic pathways were elucidated by feeding with labeled precursors in combination with HRMS^n^. Dual-culture confrontation/restriction assays of the bacterial endophyte were devised with coexisting fungal endophytes (*Pestalotiopsis virgatula*, *Aspergillus caesiellus* and *Pichia* spp.) as well as with unrelated, non-endophytic fungi belonging to the same genera. The assays were combined with scanning electron microscopy (SEM) as well as matrix-assisted laser desorption ionization imaging high-resolution mass spectrometry (MALDI-imaging-HRMS) for visualizing, both in high spatial and temporal resolution, the distribution and interplay of the compounds during microbial interactions. We demonstrated the effect of prodigiosin produced by endophytic *S*. *marcescens* MSRBB2 as an allelochemical that specifically inhibits coexisting endophytic fungi. Our results provide new insights into the physiological and ecological relevance of prodiginines and serratamolides within the context of allelopathy and chemical defense interaction occurring between coexisting endophytes harbored in *M*. *serrata*.

## Introduction

Plants are sessile organisms that incessantly encounter a plethora of environmental stresses, both biotic and abiotic^1^. Beyond this simplistic notion, however, is the fact that plants are ‘holobionts’ coexisting with complex microbiome that are adept in adroitly acclimating and adapting to the external stress factors^2,3^. The multitrophic and multifaceted communication network between host plants and associated microorganisms such as endophytic, phyllosphere, rhizosphere, soil as well as air microbiome^4–6^, lead to coevolution of selected ecological functions in Nature including genetic, biochemical and phenotypic plasticity^7^. The endophytic microbiome plays a vital role within the mutualistic network of host plants and associated micro- and macro-organisms by delivering crucial eco-specific and niche-specific functions related to the fitness of the hosts, for instance by producing chemical defense compounds, communication molecules, allelochemicals, chemical triggers, biosynthetic precursors, and epigenetic modulators^6,8–16^.

In our continuing efforts to study the functional traits of endophytes, particularly in relation to endophyte-mediated host fitness benefits, we recently embarked on investigating plants belonging to the Celastraceae family. Celastraceae plants of the *Putterlickia* and *Maytenus* genera have been known to contain a highly bioactive compound called maytansine possessing remarkable antibiotic activities along with high cytotoxicity^17,18^. In 2014, we discovered that maytansine is actually a biosynthetic product of root-associated endophytic bacterial community in *Putterlickia verrucosa* and *Putterlickia retrospinosa* plants^19^. This interesting outcome provided the scientific basis to investigate the actual producers responsible for maytansine biosynthesis in *Maytenus* plants. Thus far, we substantiated that the biosynthesis of maytansine in *M*. *serrata* is shared between the endophytic bacterial community colonizing the stem and the host plant containing non-culturable cryptic endophytes^20^. More recently, we elucidated the ecological role of maytansine as a chemical defense compound that is selectively produced and localized in vulnerable tissues such as seeds and seedlings during the germination of *Maytenus senegalensis* plants^21^.

The above discoveries led us to question the function and ecological relevance of endophytes harbored in Celastraceae plants, which do not produce maytansine. In particular, we were interested to unravel the open question: what are the biosynthetic and eco-specific roles of these endophytes? To answer this question, we embarked on elucidating the biosynthetic capacities of endophytes harbored in *Putterlickia* and *Maytenus* plants, which are incapable of producing maytansine or its analogs.

**Figure 1:**
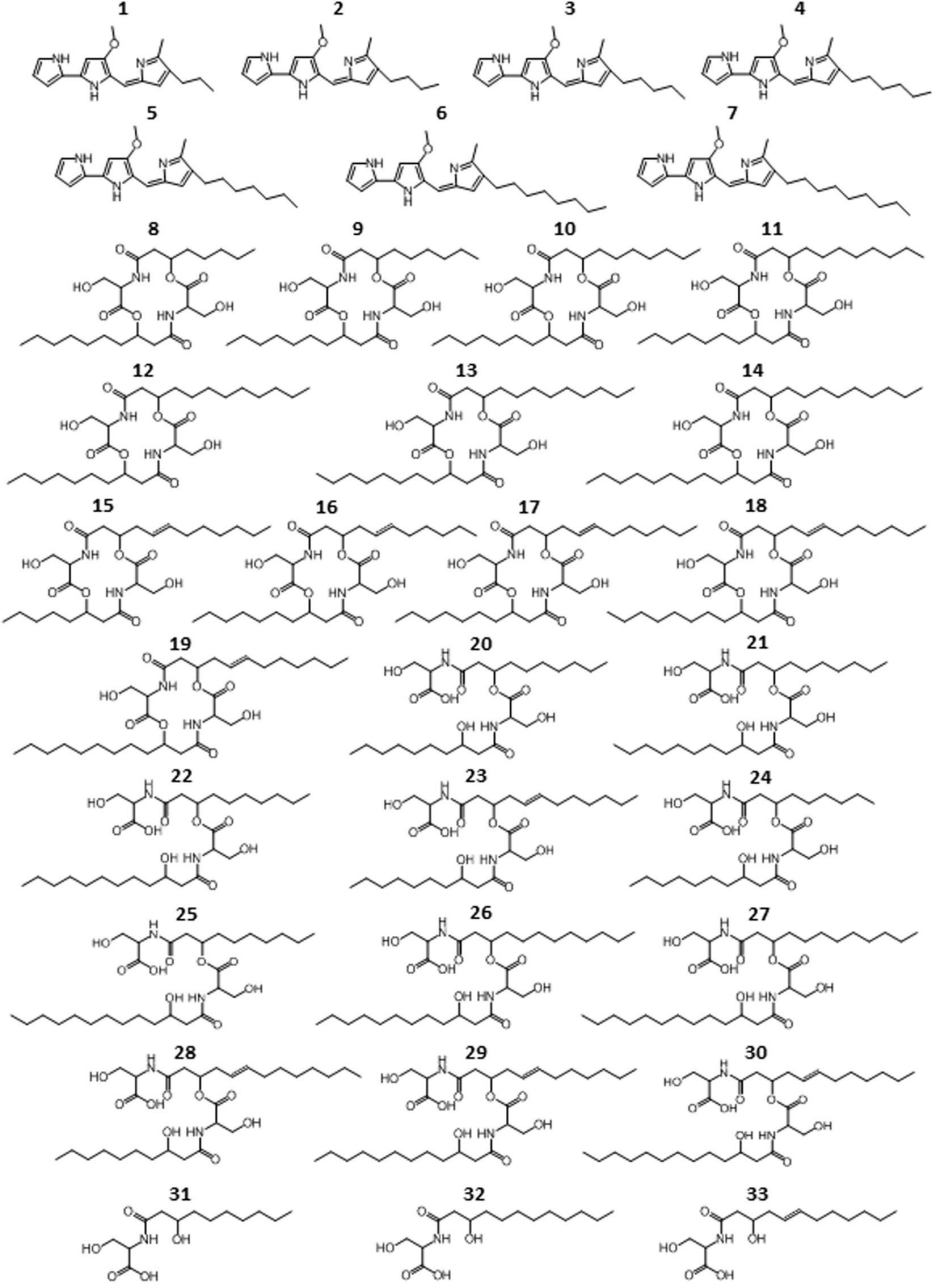
Chemical structures of secondary metabolites produced by bacterial endophyte *S*. *marcescens* MSRBB2. Prodiginines **1**–**7** (prodigiosin **3**); serratamolides **8**–**33** (serrawettin W1 **10**, serratamic acid **31**).

Herein we report the isolation and characterization of an endophytic bacterium, *Serratia marcescens* MSRBB2, harboring inner bark tissues of a *Maytenus serrata* plant prospected from Bambui, Cameroon. Chemical investigation of this bacterial endophyte employing the OSMAC (One Strain Many Compounds) approach^22^ combined with HPLC-HRMS^n^ led us to discover that the endophyte is capable of biosynthesizing 7 prodiginines (compounds **1**–**7**, Fig. 1) including prodigiosin (compound **3**), as well as 26 serratamolides (compounds **8**–**33**, Fig. 1) including serrawettin W1 (compound **10**) and serratamic acid (compound **31**). *S*. *marcescens* is a Gram-negative bacterium widespread in the environment and can be found in soil, water, and plants^23^. It is well known for the production of two different groups of secondary metabolites, the prodiginines and the serratamolides. Prodiginines are tripyrrole red pigments with different alkyl substituents with prodigiosin being the most investigated compound since it possess diverse bioactivities such as immunosuppressive, anticancer, antifungal, antibacterial, antiprotozoal, and antimalarial activities^24–26^. Furthermore, serratamolides are cyclodepsipeptides and biosurfactants with hemolytic, antibiotic and anticancer activities, with serrawettin W1 as the most investigated metabolite^27–31^. Serratamolides are also known as wetting agents, which enhance the motility of *S*. *marcescens* and therefore the capability of colonizing different environmental surfaces^32–35^.

Although *S*. *marcescens* is an intensely studied bacterium with regard to its biosynthetic pathways and biological activities of selected metabolites such as prodiginines^36,37^ and serratamolides^38^, we report it for the first time to the best of our knowledge as an endophyte of *M*. *serrata*. Given that endophytic *S*. *marcescens* MSRBB2 is capable of producing known as well as for this species new prodiginines and serratamolides, we firstly evaluated their biosynthetic pathway(s) by feeding experiments with labeled precursors coupled to HRMS^n^. Moreover, we devised dual-culture and confrontation assays with coexisting endophytes, *Pestalotiopsis virgatula* MSRBF1 and *Aspergillus caesiellus* MSRBF2, isolated from the same tissues as well as with endophytic *Pichia* spp. MSRRF1 isolated from the roots of the same plant. Finally, we coupled these assays to matrix-assisted laser desorption ionization imaging high-resolution mass spectrometry (MALDI-imaging-HRMS) in order to visualize, both in high spatial and temporal resolution, the distribution and dynamics of the compounds during the interaction of the endophytes. We discuss our results within the context of chemical interactions occurring between coexisting endophytes *in situ* emphasizing on the overall ecological role of prodiginines and serratamolides produced by endophytic *S*. *marcescens* MSRBB2 harbored in *M*. *serrata*.

## Results and Discussion

### Endophytic *S*. *marcescens* MSRBB2 produces prodiginines and serratamolides

We unraveled the secondary metabolite profile of the isolated endophytic bacterium, *S*. *marcescens* strain MSRBB2, by HPLC-HRMS and HRMS^n^. We identified 33 compounds comprising of 7 prodiginines and 26 serratamolides (compounds **1–33**, Fig. 1), and further detected several other derivatives of serratamolides by their monoisotopic masses. Besides prodigiosin, herein we report to the best of our knowledge for the first time, the production of prodiginines by *S*. *marcescens* with different alkyl chain lengths at the C3-position. These structural analogs have shorter (C_3_-C_4_, compounds **1**–**2**, Fig. 1) or longer (C_6_-C_9_, compounds **4**–**7**) alkyl chains compared to prodigiosin (C_5_, compound **3**), which could be identified by their characteristic fragmentation patterns by HRMS^n^ experiments (Supplementary Figures S1–S14). Furthermore, we propose the fragmentation pathways for each derivative (Supplementary Figures S15–S21). By chromatographic analysis, prodigiosin was found to be the main metabolite among all the prodiginines biosynthesized by endophytic *S*. *marcescens* MSRBB2 (Supplementary Figure S22). Strikingly, some of these derivatives were formerly found to be produced by marine bacteria such as *Pseudoalteromonas rubra*^39^, *Zooshikella rubidus*^40^, and *Hahella chejuensis*^41,42^.

In addition to prodiginines, endophytic *S*. *marcescens* MSRBB2 produces a plethora of serratamolides (compounds **8–33**, Fig. 1). HPLC-HRMS analysis revealed the well-known compound serrawettin W1^27^ (C_10_ + C_10_; compound **10**, Fig. 1) as the main metabolite among this class of natural products biosynthesized by endophytic *S*. *marcescens* MSRBB2 (Supplementary Figures S23–S27). Compounds **8**–**14**, **17**, **21** and **23** (Fig. 1) have also been reported earlier^30,43^. In addition to the serrawettin congeners with different alkyl chains (compounds **8**–**14**, Fig. 1), endophytic *S*. *marcescens* MSRBB2 produces new serratamolides with an additional double bond (compounds **15**, **16**, **18**, **19**), open-ring structures (compounds **20**, **22**, **24**, **25**, **26**, **27**), and open-ring structures with an additional double bond (compounds **28**, **29**, **30**). Similar to the prodiginines, structural elucidation of the serratamolides was performed using characteristic HPLC-HRMS^2^ fragmentation patterns/pathways (Supplementary Figures S28–S79). Admittedly, mass spectrometric methods alone cannot confirm the position of the double bond (dashed line). As expected, most of the compounds produced by endophytic *S*. *marcescens* MSRBB2 were in low, physiologically relevant quantities that could not be isolated for NMR measurements. Nevertheless, from the biosynthetic viewpoint, position of the double bond should be at position C5 corresponding to the known compound **17**^30^ (Fig. 1). Further, we identified some monomers of the main cyclic serratamolides, serratamic acid^44^ (C_10_; compound **31** (Fig. 1)), an analog with a C_12_ alkyl chain (C_12_; compound **32**), and a derivative with a C_12_ alkyl chain and an additional double bond (C_12:1_; compound **33**). We were able to isolate and purify the main metabolites prodigiosin, serrawettin W1, and serratamic acid that were produced in higher quantities by endophytic *S*. *marcescens* MSRBB2, which enabled us to re-confirm their structures by 1D and 2D NMR (Supplementary Figures S80–S91). Thus far, our present results exemplify the well-known fact that endophytes can serve as novel resources of bioactive natural products with implications in medical, agricultural, and industrial fields^45^.

### A common biosynthetic pathway of prodiginines elucidated by labeling studies

The biosynthetic pathway of prodigiosin (compound **3**, Fig. 1) has been examined in the respective producer microorganisms in the last years employing molecular biology approaches^26^. Some sporadic labeling studies by feeding experiments with ^13^C, ^15^N, and ^14^C labeled amino acids and other precursor molecules have also been published^36,46–50^. In the present study, in order to study the biosynthetic pathway of all prodiginines produced by endophytic *S*. *marcescens* MSRBB2, we performed feeding studies with ^15^NH_4_Cl, [1–^13^C]-L-proline, [methyl-D_3_]-L-methionine, and [1,2–^13^C_2_]-sodium acetate. This further allowed us to decipher whether the alkyl-chain derivatives of prodigiosin have a common biosynthetic pathway.

Using the OSMAC approach^22^, firstly, we cultivated endophytic *S*. *marcescens* MSRBB2 in minimal media (M6, M9, Koser citrate, and Simmons citrate broth) to achieve a high incorporation rate of the labeled precursors. Unfortunately, no pigment production could be observed (low production with M9). Therefore, we readjusted the composition of the original complex media (nutrient broth) that we used for metabolic profiling of the bacterium, by reducing the strength (i.e. half and one-fourth strength, respectively, by diluting NB with sterile ddH_2_O). We could observe a correlation between richness of the media, pigment production, and rate of incorporation of labels. Higher pigment production as well as lower incorporation rate was observed with richer media composition and vice versa.

On the one hand, due to intense analyses by HPLC-HRMS, we were able to show the incorporation of labels by the particular mass shifts in mass spectra compared to unlabeled prodiginines (Supplementary Figures S92–S116). On the other hand, we could determine the position of the label incorporations by HRMS^n^ experiments and propose the characteristic fragmentation pathways (Supplementary Figures S117–S170). Figure 2 shows the proposed biosynthetic pathway of prodiginines produced by endophytic *S*. *marcescens* MSRBB2 based on our labeling studies concomitant to previous investigations^26,41^. Prodiginines are biosynthesized by a condensation of the monopyrrole MAP (2-methyl-3-amylpyrrole) and the bipyrrole MBC (4-methoxy-2,2′-bipyrrole-5-carbaldehyde). The different prodiginines are produced by the same bipyrrole.

**Figure 2:**
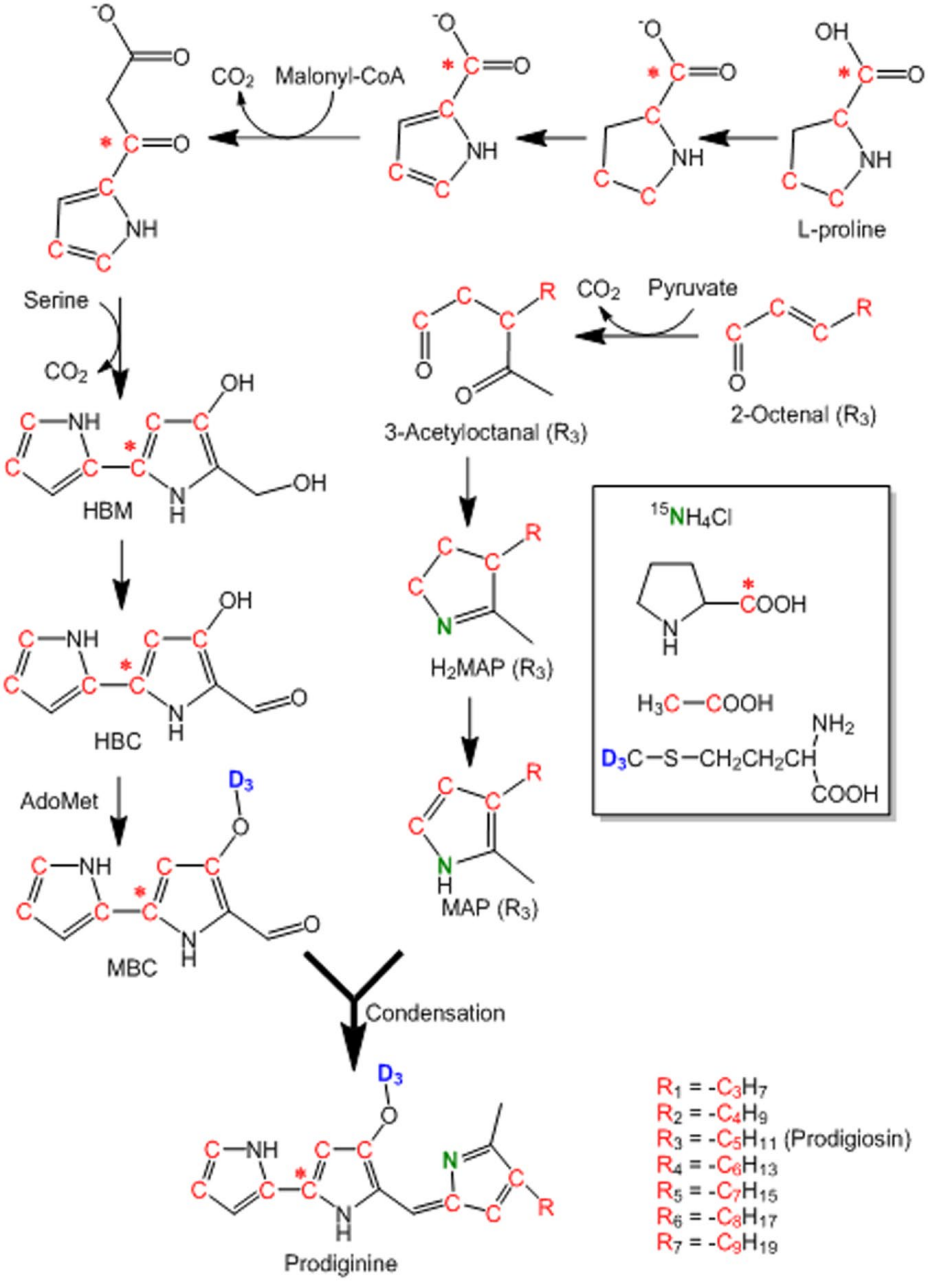
Proposed biosynthetic pathway of prodiginines produced by bacterial endophyte *S*. *marcescens* MSRBB2 with assigned incorporation of labeled precursors (^15^NH_4_Cl, [1–^13^C]-L-proline, [methyl-D_3_]-L-methionine, [1,2–^13^C_2_]-sodium acetate). The pathway was modified based on earlier publications^26,41^. MAP: 2-methyl-3-amylpyrrole; HBM: 4-hydroxy-2,2′-bipyrrole-5-methylalcohol; HBC: 4-hydroxy-2,2′-bipyrrole-5-carboxaldehyde; MBC: 4-methoxy-2,2′-bipyrrole-5-carboxaldehyde; AdoMet: S-adenosylmethionine.

The alkyl-chain derivatives are derived from different monopyrroles (homologues of MAP), thus, indicating a low substrate specificity for the enzymes involved. The initial precursors of MAP have been shown to be derived by enzymes of fatty acid biosynthetic pathway or by auto-oxidation of unsaturated fatty acids^26^. In the present study, we show that for different alkyl-chain derivatives, the monopyrrole homologues are derived from a common biosynthetic route. In our labeling studies, the ^15^N of ^15^NH_4_Cl was typically incorporated as the monopyrrole nitrogen for the different prodiginines. Moreover, the different alkyl-chains emanated from acetate units. The number of labeled acetate units that was incorporated depended on the length of the alkyl-chain. Table 1 summarizes the observed incorporation rates of the acetate-units in the prodiginines from different feeding studies. Similar to previous labeling studies on prodigiosin, our results also show that a maximum of seven acetate units could be incorporated (maximum mass shift of 14 Da). Additional MS^2^ spectra of labeled prodigiosin (five, six, and seven [1,2–^13^C_2_]-acetate units incorporated) are shown in the supplementary information (Supplementary Figures S171–S173). Three acetate units were incorporated in the MBC moiety, with two in ring A via the Krebs cycle and formation of proline and one unit in ring B^36^. Further, three acetate units were incorporated in the alkyl chain and one more in ring C of prodigiosin^36^. For the other prodiginines, the mass shift depended on the length of the alkyl-chain and varied accordingly. For example, 2-methyl-3-propyl-prodiginine showed a maximal mass shift of 12 (shorter alkyl-chain). Admittedly, we could not detect (<LOD) the theoretical maximal mass shift(s) for all prodiginines owing to different production rates of endophytic *S*. *marcescens* MSRBB2 leading to mixed incorporations with unlabeled acetate units or other precursors. Taken together, our labeling experiments revealed that the alkyl-chain derivatives of prodigiosin produced by endophytic *S*. *marcescens* MSRBB2 have a common biosynthetic pathway. Endophytic *S*. *marcescens* MSRBB2 biosynthesizes different prodiginines by condensation of MBC and different MAP homologues, underlining the relaxed specificity of the condensing enzyme, corroborating Williamson *et al*.^26^. The incorporation of MAP homologues with different alkyl-chain lengths was recently demonstrated in a mutasynthesis study with a *Pseudomonas putida* mutant strain deficient in the MAP biosynthetic gene *pigD*^51^, thereby corroborating our present results on the diverse prodiginines produced by endophytic *S*. *marcescens* MSRBB2.

**Table 1:** Peak height ratios from high resolution mass spectrometry of prodiginines labeled with [1,2–^13^C_2_]-acetate produced by *S*. *marcescens* MSRBB2 in different feeding studies (diluted NB to half and one-fourth strength in sterile ddH_2_O with 2 mg/mL [1,2–^13^C_2_]-sodium acetate; n.d. = not detected; P = Prodiginine).

No.	Prodiginine	Observed peak height (% of M)							
		M + 2	M + 4	M + 6	M + 8	M + 10	M + 12	M + 14	M + 16
1	2-methyl-3-propyl-P	138	69	30	11	9	6	—	—
2	2-methyl-3-butyl-P	508	468	98	15	n.d.	n.d.	—	—
3*	2-methyl-3-pentyl-P	557	1676	2523	1990	740	85	8	—
4	2-methyl-3-hexyl-P	302	349	203	64	3	n.d.	n.d.	—
5*	2-methyl-3-heptyl-P	577	1949	3702	4148	2662	804	87	2
7	2-methyl-3-nonyl-P	266	348	192	58	8	n.d.	n.d.	n.d.

### Dual-culture assay of endophytic *S*. *marcescens* MSRBB2 and associated endophytic fungi

The ecological relevance of prodiginines, especially prodigiosin, has remained an open question with several eco-specific notions put forward so far. Theories range from prodigiosin being a ‘waste’ product and metabolic sink of primary metabolism^52^ to being a vital compound that plays an important role in the survival of the producing organism(s)^53–55^. This is compounded by the fact that a higher percentage of environmental isolates of *S*. *marcescens* have been found to produce the red pigment compared to clinical isolates where most are non-pigmented^23,55–57^. Against this background, we aimed to decipher the plausible ecological role of prodiginines biosynthesized by endophytic *S*. *marcescens* MSRBB2 by studying the interaction and chemical communication of *S*. *marcescens* with associated endophytic microorganisms. Therefore, we isolated fungal endophytes from the same host plant. Three different endophytic fungal strains could be isolated: two endophytic fungi identified as *Pestalotiopsis virgatula* (strain MSRBF1) and *Aspergillus caesiellus* (strain MSRBF2) were isolated from the inner bark tissues (same tissue from which endophytic *S*. *marcescens* MSRBB2 was isolated), and an endophytic *Pichia* spp. (strain MSRRF1) was isolated from the roots of the same plant.

**Figure 3:**
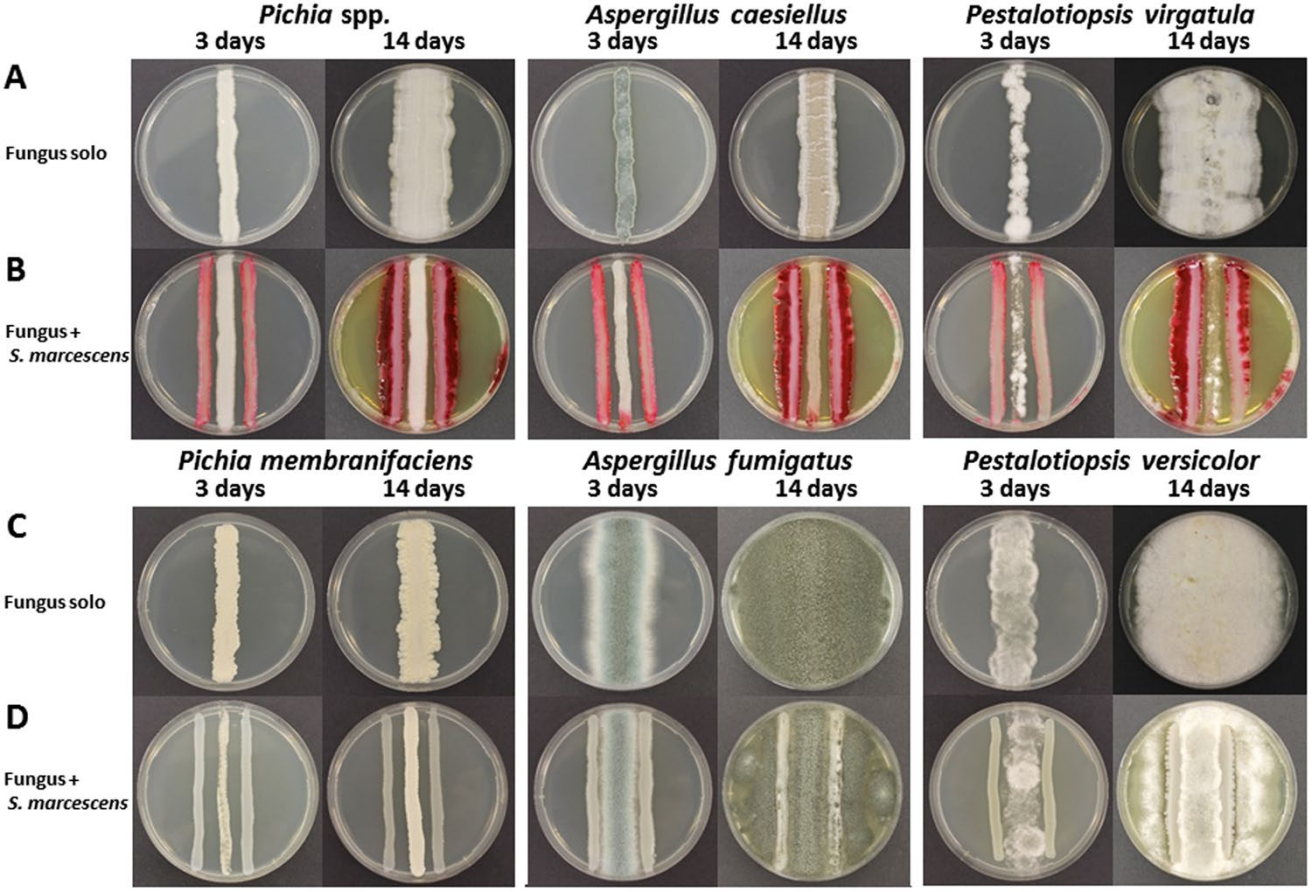
Dual-culture assay of bacterial endophyte *S*. *marcescens* MSRBB2 and fungal endophytes (**A**,**B**) viz. *Pichia* spp., *Aspergillus caesiellus*, and *Pestalotiopsis virgatula* isolated from the same host plant and unrelated fungi (**C**,**D**) viz. *Aspergillus fumigatus* (DSM 21023), *Pichia membranifaciens* (DSM 70366), and *Pestalotiopsis versicolor* (DSM 62887). Optical images of fungal mono-cultivations (**A**,**C**) and co-cultivations with *S*. *marcescens* MSRBB2 (**B**,**D**) after 3 and 14 days. Triggered prodiginines production of *S*. *marcescens* MSRBB2 by presence of associated fungal endophytes harboring the same host plant was observed.

We devised dual-culture (confrontation/restriction) assays by co-cultivating endophytic *S*. *marcescens* MSRBB2 with associated endophytic fungi harboring the same tissue. This enabled us to investigate the chemical basis of interaction and communication between these coexisting endophytic microorganisms (Fig. 3). The different endophytic fungi were challenged with endophytic *S*. *marcescens* MSRBB2 (Fig. 3B) to study the growth, production and spatial as well as temporal dynamics of metabolites produced by the endophytic bacterium. By comparing the axenic cultures of the endophytic fungi (Fig. 3A) with the dual-cultures (Fig. 3B), it was revealed that the growth of each of the coexisting endophytic fungus was strongly restricted by endophytic *S*. *marcescens* MSRBB2. This restriction effect did not occur due to a nutrient competition or physical barrier posed by organismal biomass as confirmed by bacterial controls (*Escherichia coli* and *Staphylococcus aureus*, Supplementary Figures S174–S176). In addition, we observed that the production of prodiginines by endophytic *S*. *marcescens* MSRBB2 increased in the presence of associated endophytic fungi. To further investigate whether endophytic fungi associated with endophytic *S*. *marcescens* MSRBB2 were specifically able to trigger an increased production of prodiginines, we performed dual-culture assays with unrelated, non-endophytic fungi belonging to the same genera (Fig. 3C,D). *Aspergillus fumigatus* (DSM 21023), *Pichia membranifaciens* (DSM 70366), and *Pestalotiopsis versicolor* (DSM 62887) were employed. Endophytic *S*. *marcescens* MSRBB2 did not produce prodiginines in the presence of the unrelated fungi (Fig. 3C,D). Moreover, *A*. *fumigatus* and *P*. *virgatula* were able to completely overgrow endophytic *S*. *marcescens* MSRBB2. These results demonstrate that endophytic *S*. *marcescens* MSRBB2 is able to specifically recognize the presence of associated endophytic fungi harboring the same tissues (or same ecological niche) triggering the production of prodiginines.

In order to pinpoint the nature of antagonism between endophytic *S*. *marcescens* MSRBB2 and associated endophytic fungi, we further analyzed the dual-cultures by scanning electron microscopy (SEM) and compared the results with those of fungal axenic cultures (Supplementary Figures S177–S197). Interestingly, SEM did not reveal any decomposition, disruption, or collapse of conidia, conidiophore, hypha, or mycelium on challenging the endophytic fungi with endophytic *S*. *marcescens* MSRBB2. In order to confirm whether the observed restriction/inhibition was caused by prodigiosin, which is known to have antifungal activity^58–60^, we applied two strategies. Firstly, we wanted to exclude the possibility that the observed fungal inhibition was due to extracellular enzymes such as chitinase. Some *S*. *marcescens* strains are known to produce and release chitinase enzymes that can cause growth inhibition and decomposition of mycelia and inhibition of conidial formation^61^. Therefore, we performed an agar-based assay in which we inoculated a chitinase detection agar to show that endophytic *S*. *marcescens* MSRBB2 in general is able to produce chitinase (Supplementary Figure S204A). Furthermore, owing to the fact that glucose is a known inhibitor of chitinase expression in *S*. *marcescens*^62^ and our dual-culture assays were performed on PDA (having a glucose concentration of 20 g/L), we additionally performed the assay using chitinase detection media supplemented with 20 g/L of glucose. By this assay, we could rule out the possibility of extracellular chitinase being responsible for the observed restriction/inhibition of the fungi in our dual-culture assays (Fig. S204B). Thus, we can exclude a chitinase production in the dual-culture assay. Finally, in order to pinpoint the effect of prodigiosin, we isolated and purified prodigiosin produced by endophytic *S*. *marcescens* MSRBB2. We performed different dose- and time-dependent disc-assays with purified prodigiosin against the related fungi. Prodigiosin clearly showed a dose-dependent restriction/inhibition effect on the three fungi (Supplementary Figures S198–S203).

Taken together, we could show that endophytic *S*. *marcescens* MSRBB2 was able to restrict the growth of coexisting endophytic fungi without killing them. Our results serve as another example of allelopathy^63–65^ where prodigiosin is employed as an allelochemical or a chemical defense compound by endophytic *S*. *marcescens* MSRBB2 to restrict coexisting endophytes. Strikingly, endophytic *S*. *marcescens* MSRBB2 cannot completely kill associated endophytic fungi using prodigiosin as an allelochemical. It is plausible that coevolution of these microorganisms inhabiting the same ecological niche (same tissues and/or host plant) has led to adaptation of the endophytic fungi to the secondary metabolites (e.g. prodigiosin) produced by endophytic *S*. *marcescens* MSRBB2 similar to what has been observed in other earlier studies^66^. Taken together, our results exemplify part of the “balanced antagonism” concept existing between endophytic microorganisms harboring the same ecological niche^45,67^ wherein two or more endophytes colonize any given tissue and maintain endophytic lifestyles by employing a plethora of both antagonistic and synergistic means.

### Interaction of endophytic *S*. *marcescens* MSRBB2 and fungal endophytes visualized by MALDI-imaging-HRMS

**Figure 4:**
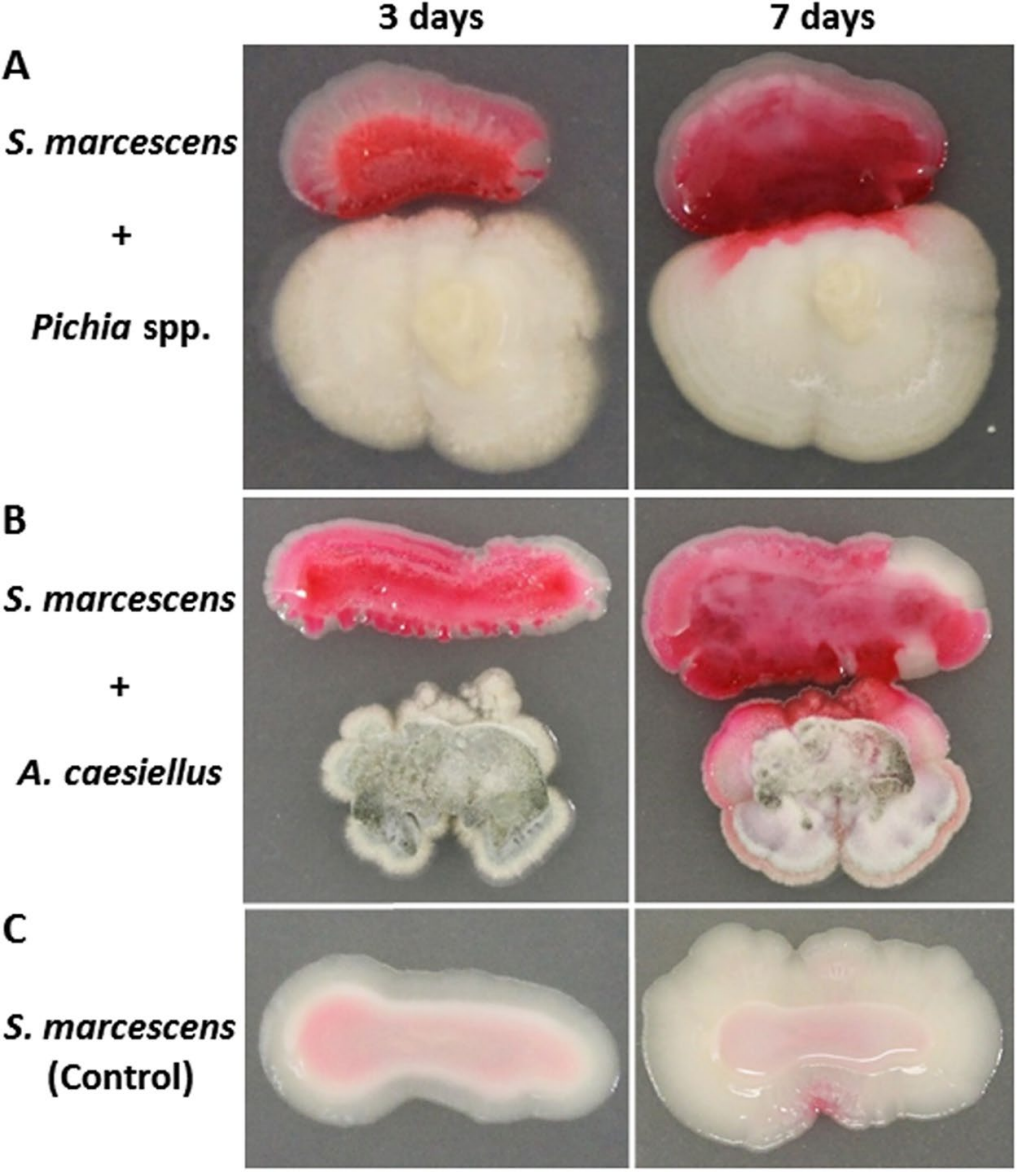
Monitored co-cultivation of bacterial endophyte *S*. *marcescens* MSRBB2 (top) and fungal endophytes ((**A**) *Pichia* spp. (bottom); (**B**) *Aspergillus caesiellus* (bottom)). Accumulation and enhanced production of prodiginines (red pigments) at contact site in the presence of fungi. Control of *S*. *marcescens* MSRBB2 in absence of fungi (**C**).

**Figure 5:**
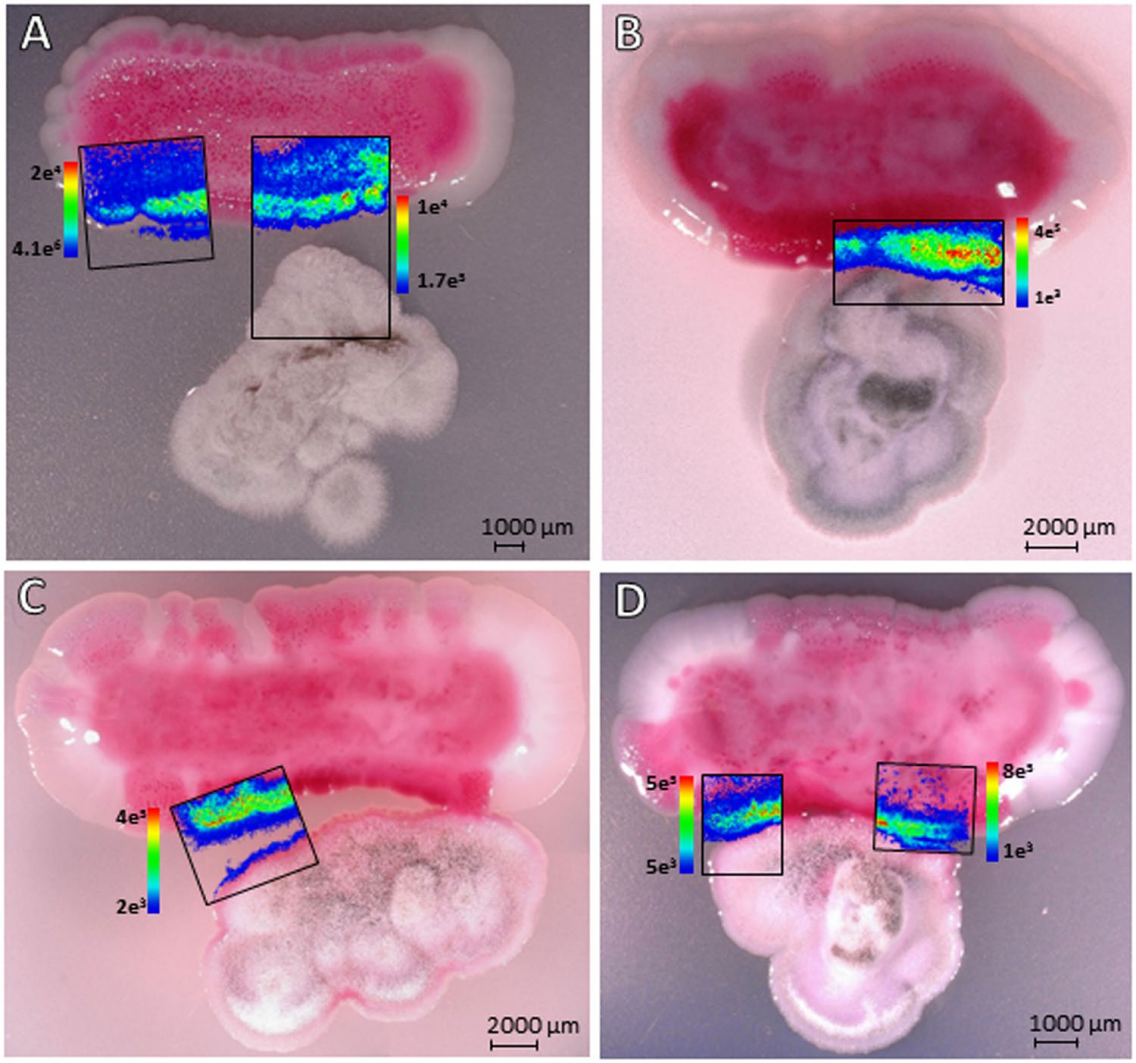
Localization of prodigiosin (compound **3** (Fig. 1), [M + H]^+^  ± 2 ppm) by MALDI-imaging-HRMS of the interaction of bacterial endophyte *S*. *marcescens* MSRBB2 (top) and fungal endophyte *Aspergillus caesiellus* (bottom) after different days of co-cultivation ((**A**) 3 days; (**B**) 5 days; (**C**,**D**) 7 days).

To further study the interaction of endophytic *S*. *marcescens* MSRBB2 and the coexisting endophytic fungi, we performed MALDI-imaging-HRMS of the dual-cultures to visualize the spatial distribution of the produced prodiginines. We co-cultivated endophytic *S*. *marcescens* MSRBB2 with *Pichia* spp. MSRRF1 and *A*. *caesiellus* MSRBF2. Unfortunately, due to the height of mycelia and aerial hyphae generation, *P*. *virgatula* was not suitable for MALDI-imaging sample preparation (planar height profile). Therefore, we monitored the co-cultivation of endophytic *S*. *marcescens* MSRBB2 with *A*. *caesiellus* and *Pichia* spp. visually for at least 7 days and investigated the spatial as well as temporal distribution of secondary metabolites by MALDI-imaging-HRMS. An enhanced production of prodiginines was observed during co-cultivation, which typically accumulated towards the side facing the associated fungi (red pigments; Fig. 4A,B). This was not the case in the absence of fungi (Fig. 4C). During co-cultivation, an enhanced production of prodiginines was observed that not only spread throughout for the entire bacterial colony, but also accumulated at the areas of direct contact with fungal biomass (intense red pigment production). This triggered production was further visualized in high spatial resolution and confirmed by MALDI-imaging-HRMS. Figure 5 shows the spatial and temporal distribution of prodigiosin during the interaction of *S*. *marcescens* MSRBB2 and fungal endophyte *A*. *caesiellus*. Prodigiosin was detected with high intensities at the edge of the bacterium facing the fungus, which gradually accrued towards the fungus. Moreover, at later time-points (e.g. 7 days), *S*. *marcescens* MSRBB2 physically touched the fungus and started surrounding it, while it continued to localize prodigiosin at its interface with the fungus (Fig. 5C,D). This was additionally confirmed by microscopy of the dual-cultures (Supplementary Figure S205).

**Figure 6:**
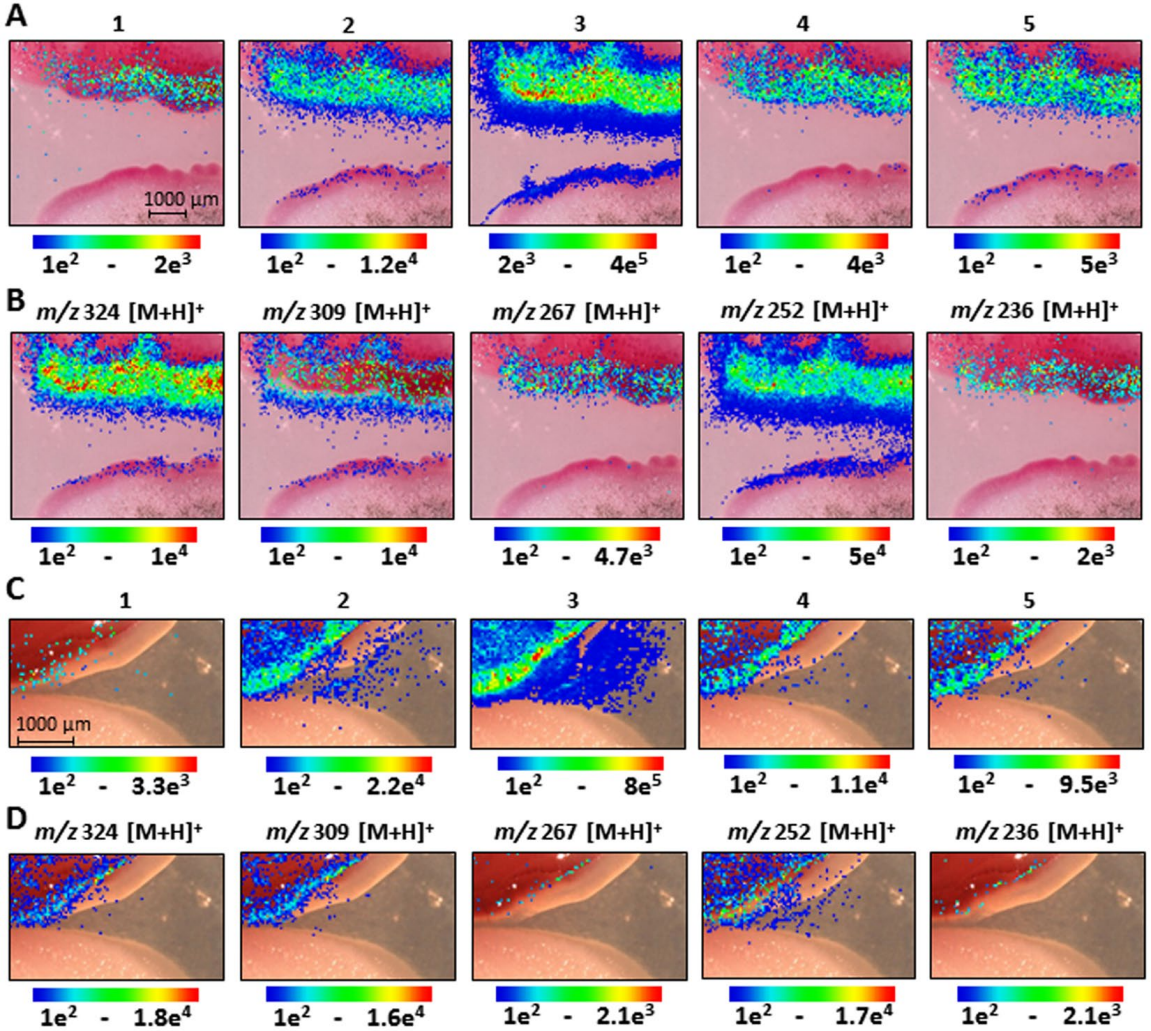
MALDI-imaging-HRMS of the interaction of bacterial endophyte *S*. *marcescens* MSRBB2 (top) and fungal endophytes (**A**,**B**) *Aspergillus caesiellus* (bottom); (**C**,**D**) *Pichia* spp. (bottom)) after 7 days. Optical image with assigned scan area and localization of prodiginines (compound **1**–**5** (Fig. 1), [M + H]^+^  ± 2 ppm). Different fragments of MALDI-imaging-HRMS^2^ of prodigiosin (compound **3**, B,D).

Besides prodigiosin, we also investigated the spatial and/or temporal distribution of other identified prodiginines **1**–**5** (**6** and **7** < LOD, Fig. 1) by MALDI-imaging-HRMS. Figure 6A and C show the ion images of prodiginines **1**–**5** (Fig. 1) of co-cultivations of *S*. *marcescens* MSRBB2 with *Pichia* spp. MSRRF1 as well as *A*. *caesiellus* MSRBF2 after 7 days. Remarkably, all compounds showed the same production and localization pattern as that of prodigiosin (compound **3**, Fig. 1). These data not only exemplified the results of our labeling studies (common biosynthetic pathway) but also indicated a similar physiological role of the prodiginines. We further confirmed the identity of prodigiosin by MALDI-imaging-HRMS^2^ measurements and its characteristic fragments (Fig. 6B,D) over different biological replicates and several time points (Supplementary Figures S206–S210). As a control, we prepared axenic cultures of endophytic *S*. *marcescens* MSRBB2 and performed MALDI-imaging-HRMS, which revealed that the compounds did not accumulate at the edge of the colony when the bacterium could not sense a coexisting fungus at its vicinity (Supplementary Figures S211–S212). Finally, the accumulation and enhanced production of prodiginines was further re-confirmed in parallel by extraction and comparison using HPLC-HRMS (Supplementary Figure S213).

### *In situ* visualization of serratamolides by MALDI-imaging-HRMS

Serratamolides, especially serrawettin W1, are known to have wetting properties that enhance the motility, spreading, and colonization of new surfaces by *S*. *marcescens*^28,32–35,43^. Using cues from earlier reports^32^, in the present study, we firstly verified this general phenomena for our strain (endophytic *S*. *marcescens* MSRBB2). At 30 °C, the endophytic bacterium was able to produce serratamolides. However, at 37 °C the production of prodigiosin and serratamolides ceased concomitant to highly reduced motility of endophytic *S*. *marcescens* MSRBB2 (Supplementary Figure S214).

**Figure 7:**
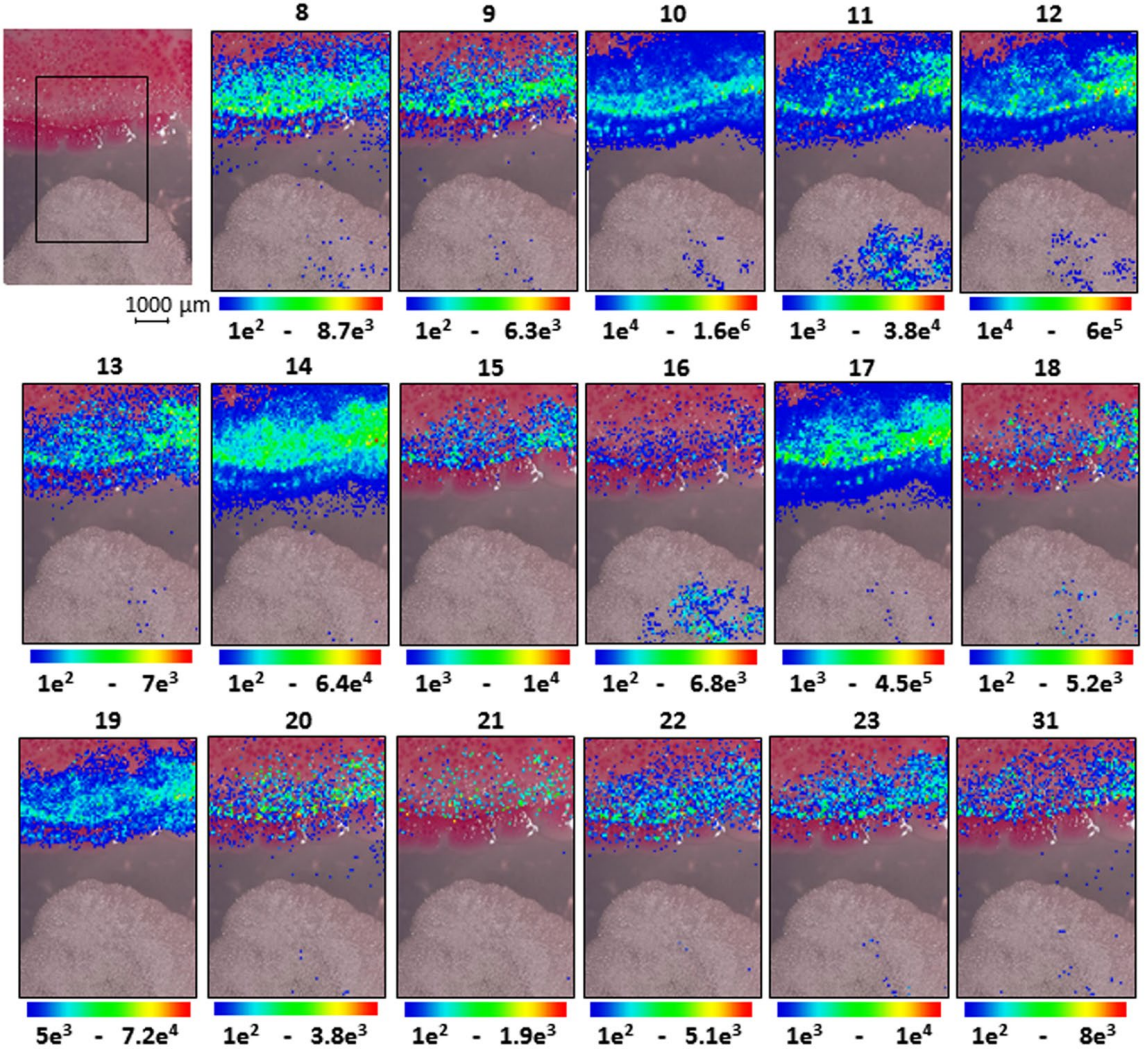
MALDI-imaging-HRMS of the co-cultivation of bacterial endophyte *S*. *marcescens* MSRBB2 (top) and fungal endophyte *Aspergillus caesiellus* (bottom) after 3 days. Optical image with assigned scan area and localization of serratamolides **8**–**23** and **31** (Fig. 1, [M + K]^+^  ± 2 ppm).

**Figure 8:**
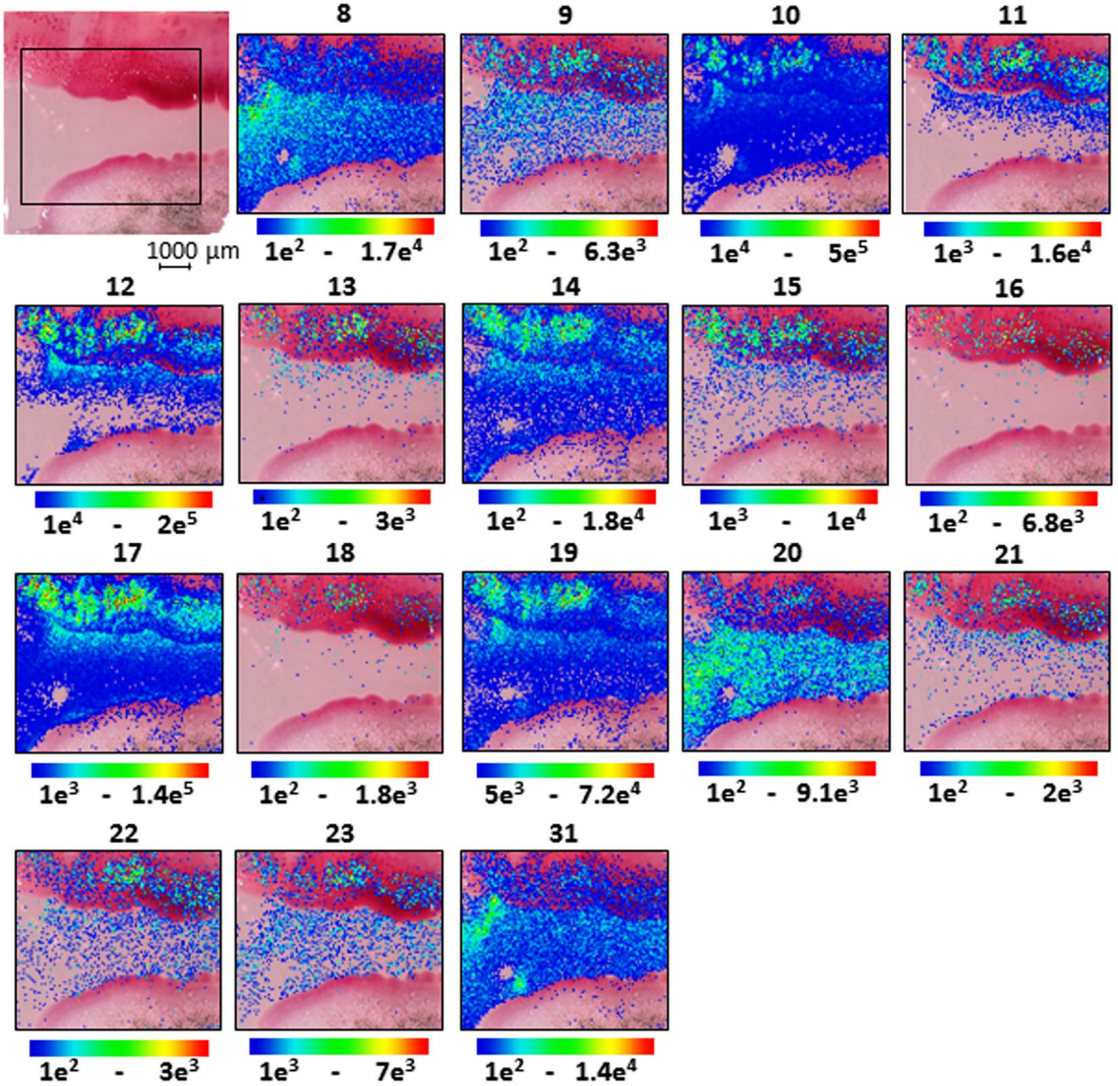
MALDI-imaging-HRMS of the co-cultivation of bacterial endophyte *S*. *marcescens* MSRBB2 (top) and fungal endophyte *Aspergillus caesiellus* (bottom) after 7 days. Optical image with assigned scan area and localization of serratamolides **8**–**23** and **31** (Fig. 1, [M + K]^+^  ± 2 ppm).

Thereafter, we investigated the utilization and plausible physiological role of serratamolides (abundant ones, compounds **8**–**23**, **31**, (Fig. 1)) produced by endophytic *S*. *marcescens* MSRBB2 when challenged with the coexisting endophytic fungus *A*. *caesiellus* by MALDI-imaging-HRMS *in situ* at different time points. After 3 days, the compounds accumulated at the edge of the *S*. *marcescens* MSRBB2 colony towards the fungus (Fig. 7). As biosurfactants, serratamolides reduce the surface tension of the agar and support the encountering of new surfaces, therefore enhancing the overall fitness and survival of the producer. After 7 days, we could clearly detect not only accumulation of the serratamolides at the original *S*. *marcescens* MSRBB2 colony but also an intense release into the surrounding media typically towards side facing the fungus (Fig. 8). The serratamolides are produced and released to enhance the motility of endophytic *S*. *marcescens* MSRBB2, helping the bacterium to colonize faster and overgrow the fungal biomass during co-cultivation experiments as observed by SEM. Further MALDI-imaging-HRMS measurements at different time points (temporal) revealed the same phenomena (Supplementary Figures S215–S218). Interestingly, the axenic culture of *S*. *marcescens* MSRBB2 showed reduced production of serratamolides compared to that when challenged with endophytic *A*. *caesiellus* (Supplementary Figures S219–S220). The presence of the fungus led to an enhanced production and directed accumulation of serratamolides, similar to that of prodiginines. Interestingly, serrawettin W1 was found to be concurrently produced with prodigiosin by pigmented *S*. *marcescens* strains, similar to earlier studies on prodigiosin-producing bacteria^32,34^, thereby ratifying that biosynthesis of both compounds can be regulated by a transcriptional regulator (HexS)^68^. Sunaga *et al*.^69^ further showed that the protein encoding gene *pswP* regulates the production of both compounds. Therefore, in the present study, it was not surprising to note that both serratamolides and prodiginines had a related spatial distribution when produced by endophytic *S*. *marcescens* MSRBB2.

All serratamolides including serratamic acid (compound **31**, Fig. 1) also showed the same spatial distribution, indicating a common biosynthetic pathway and regulation of all serratamolides. The biosynthesis of serrawettin W1 have been found to depend on a uni-modular non-ribosomal peptide synthetase (NRPS), specifying only L-serine^38^. The other precursor of serrawettin W1, 3-D-hydroxydecanoyl, emanate from the fatty acid biosynthetic pathway^38^. In case of endophytic *S*. *marcescens* MSRBB2, it is plausible that the involved NRPS has low substrate specificity, which results in the high number of biosynthesized serratamolides with different alkyl chains. Further, the intra-molecular ester linkage formation for cyclization seems unnecessary for the release from the thioesterase domain of NRPS, resulting in serratamolides with open-ring structures and monomers indicating these compounds as intermediates^38^. Another possibility for the occurrence of open-ring derivatives and monomer-like serratamic acid is decomposition by hydrolysis of the cyclic serratamolides (Supplementary Figure S221). In addition to the labeling studies for prodiginines, we could also show the incorporation of labeled [1,2–^13^C_2_]-sodium acetate into the abundant serratamolides. This revealed the acetate-units as building blocks of the different hydroxy-fatty acids as precursors of serratamolides (Supplementary Table S1).

### Leaf puncture assay

We also investigated possible harmful effects of the secondary metabolites produced by endophytic *S*. *marcescens* MSRBB2 to the host plant by a leaf puncture assay (Supplementary Figure S222). The isolated main metabolites prodigiosin and serrawettin W1 were taken as representatives of the two metabolite groups and tested against the *Maytenus* plants *M*. *senegalensis*, *M*. *heterophylla*, and *M*. *canariensis* (fresh *M*. *serrata* was not available). We could not detect necrosis, lesions, or other phytotoxic effects, signifying that the host plant is not impaired by these metabolites. Instead, it is more likely that the host plant benefits from serratamolides and prodiginines production owing to their bioactivities^58–61,70^.

## Conclusions

In plants, different microbial endophytes often colonize a single host leading to multifaceted chemical and molecular interactions between associated endophytes and the host plant. Therefore, the commonly used approach of isolating a single endophyte and investigating the established axenic culture in diverse artificial experimental conditions could lead to poor understanding of organismal interactions ongoing in Nature^6,8^. Therefore, in the present study, we tried to imitate possible ecological scenarios by co-culturing endophytic *S*. *marcescens* MSRBB2 with associated fungal endophytes residing the same ecological niche (same tissues and/or host plant). On the one hand, we could show restriction of fungal growth by prodiginines, especially prodigiosin, produced by endophytic *S*. *marcescens* MSRBB2. In fact, we observed that only the presence of coexisting fungi at the vicinity of endophytic *S*. *marcescens* MSRBB2 triggered the production of prodiginines (allelochemicals). Unrelated, non-endophytic fungi belonging to the same genera as those that coexist with endophytic *S*. *marcescens* MSRBB2 in the same ecological niche were not able to trigger the production of prodiginines. Concurrently, they showed a far lesser (or no) inhibition or even completely overgrew endophytic *S*. *marcescens* MSRBB2 in co-cultivation assays. The present study provides a scientific handle to further investigate the exact molecular and chemical triggers and recognition factors in play between endophytic *S*. *marcescens* MSRBB2 and coexisting endophytic fungi.

## Materials and Methods

### Isolation and identification of endophytes

The endophytic bacterium *S*. *marcescens* MSRBB2 and the fungal endophytes *Pestalotiopsis virgatula* MSRBF1, *Aspergillus caesiellus* MSRBF2 and endophytic *Pichia* spp. MSRRF1 were isolated from fresh bark and root of *Maytenus serrata* (Celastraceae), respectively. The host plant was bioprospected from Bambui in Cameroon in December 2014, identified by an experienced botanist (Mr. V. Nana, the National Herbarium of Cameroon in Yaoundé), and authenticated by comparison with a previously collected *M*. *serrata* plant (voucher number 26298 HNC; dated September 2014)^20^. The process of isolation, maintenance, and preservation of endophytes were done according to the published methods^19^. Representative images of *S*. *marcescens* MSRBB2 during the isolation procedure and of the isolated endophytic fungi are shown in the supplementary information (Supplementary Figures S223–S224).

Endophytic *S*. *marcescens* MSRBB2 was identified at Bruker Daltonik GmbH (Bremen, Germany) by the MALDI biotyper (MALDI-TOF mass spectrometer Microflex) and database comparison with a score value of 2.34 (species consistency). Furthermore, the endophytic bacterium was confirmed as *S*. *marcescens* by 16s rDNA sequencing using the primers 27 f (5′-AGA GTT TGA TCM TGG CTC AG-3′) and 1492r (5′-ACG GYT ACC TTG TTA CGA CTT-3′), and the fungal endophytes were identified by ITS sequencing as *Pestalotiopsis virgatula*, *Aspergillus caesiellus*, and *Pichia* spp. using previously established methods^67^. The 16S rDNA and ITS sequences have been deposited at the EMBL-Bank (awaiting accession numbers).

### Feeding studies with stable isotope labeled precursors

For the investigation of the biosynthesis of the produced prodiginines and serratamolides, we performed feeding studies with endophytic *S*. *marcescens* MSRBB2 with diluted nutrient broth (1/2 and 1/4^th^ strength of the specified composition) (Nutrient broth No 1, Sigma-Aldrich, Steinheim, Germany) amended with stable isotope labeled precursors (Cambridge Isotope Laboratories, Inc., Tewksbury, USA). 50 mL of each nutrient broth composition was inoculated with endophytic *S*. *marcescens* MSRBB2 and stationary incubated at 30 °C for 2–3 days until first pigment production was visible. Thereafter, 50 mg of ^15^NH_4_Cl (98% ^15^N), 50 mg of [1–^13^C]-L-proline (99% ^13^C), 75 mg [methyl-D_3_]-L-methionine (98% D), and 50 or 100 mg [1,2–^13^C_2_]-sodium acetate (99% ^13^C) was added separately to 50 mL nutrient broth as aqueous solution via sterile filtration (syringe filters, pore size 0.2 µm, CA, Roth, Karlsruhe, Germany) and further incubated for 7 days. The fermentations were stopped and extracts were obtained by evaporation using a rotary evaporator (Laborota 4001, Heidolph, Schwabach, Germany) in a 40 °C water bath at 150 rpm. The extracts were re-dissolved in methanol, transferred and reduced to 0.5 mL sample volume for HPLC-HRMS measurements.

### Dual-culture assay, disc assays, chitin agar assay, and scanning electron microscopy

For the dual-culture assay of the endophytic fungi with endophytic *S*. *marcescens* MSRBB2 and other pathogens, the microorganisms were cultured prior to the assay on potato dextrose agar (fungi) or nutrient agar (bacteria), respectively, at 30 °C. Bacterial and fungal material was collected with a loop, transferred into 1 ml sterile double distilled water, and homogenized by shaking. With a sterile swab, the fungal suspension was streaked as a line in the middle of a Petri dish containing potato dextrose agar. Endophytic *S*. *marcescens* MSRBB2 and the pathogens, namely *S*. *aureus* (DSM 799) and *E*. *coli* (DSM 682) were streaked in a similar fashion as a line with approximately 1 cm distance on both sides of the fungal inoculum. For the dual-culture (confrontation/restriction) assay, co-cultivations in triplicates were incubated at 30 °C for 14 days, monitored visually, and documented (including photographic evidence). In addition, the dual-culture assay was performed with unrelated, non-endophytic fungi (*Aspergillus fumigatus* (DSM 21023), *Pichia membranifaciens* (DSM 70366), and *Pestalotiopsis versicolor* (DSM 62887)). In case of *P*. *membranifaciens*, the incubation and assays were performed at 23 °C due to the organismal growth behavior. Possible structural changes of conidia, conidiophore, hypha, or mycelium of the fungi were investigated by scanning electron microscopy (SEM) using previously published methods^71^. In short, areas of approximately 1 cm^2^ agar were cut with a razor blade, frozen with liquid nitrogen and lyophilized. Freeze-dried samples were fixed with an electrically conductive tape and images recorded using a scanning electron microscope S-4500 (Hitachi High-Technologies, Krefeld, Germany) with a field-emission gun in low-voltage mode (1 kV).

The chitinase detection agar assay was performed using previously reported procedures^72^ and additionally modified. Briefly, chitinase detection agar (15 g agar, 1 g citric acid monohydrate, 2 g KH_2_PO_4_, 0.3 g MgSO_4_x7H_2_O, 3 g (NH_4_)_2_SO_4_, 4.5 g colloidal chitin and 0.15 g Bromocresol purple (per liter), adjusted to pH 4.7) was prepared using published methods^72^ having the same composition and with additional glucose (20 g/L) (corresponding to the concentration of glucose in the used PDA). Endophytic *S*. *marcescens* MSRBB2 was collected with a loop from NA, transferred into 1 ml sterile double distilled water, and homogenized by shaking. 15 µL bacterial solution was transferred onto sterile 6 mm paper discs and air-dried. The inoculated discs were transferred onto Petri plates containing the two chitinase detection agars, incubated at 30 °C for 14 days, monitored visually, and documented (including photographic evidence). For the prodigiosin disc assay, the fungal cell suspensions were prepared similar to the dual-culture assay. The fungi were streaked in a Y-shape and as a line on a Petri dish (PDA) with a sterile swab. 20 µL (Y-shape assay) and 10 µL (line assay) of different prodigiosin concentrations (1, 0.1, 0.01, and 0.001 mg/mL dissolved in MeOH) were transferred onto 6 mm sterile paper discs. After drying, the paper discs were transferred onto the plate around the centroid of the Y-shape (Y-shape assay) and onto the middle of the streaked line (line assay). Blank discs were prepared with MeOH and incubated equally (30 °C) for 7 days.

### Co-cultivation for MALDI-imaging-HRMS

For studying the interaction of endophytic *S*. *marcescens* MSRBB2 and the endophytic fungi, the dual-cultures were adjusted for MALDI-imaging-HRMS sample preparation requirements. Endophytic fungi were streaked as a small line or punctually onto potato dextrose agar (PDA). Biomass of *S*. *marcescens* MSRBB2 was transferred with a loop and suspended into sterile double distilled water in an Eppendorf tube. 15 µL of this solution was placed as a line in front of the fungi at a distance of 1 cm. Each co-culturing was done with at least 10 replicates and incubated at 30 °C. Axenic cultures of endophytic *S*. *marcescens* MSRBB2 were prepared in the same manner. The growth and the production of prodigiosin was temporally monitored visually and the documented (including photographic evidence) from 3 to 7 days. At different time points, MALDI-imaging-HRMS measurements were also performed. In addition, we analyzed methanol extracts of the cultivations by HPLC-HRMS.

### Sample preparation for MALDI-imaging-HRMS

Areas of interest of the PDA with the seeded or cultivated microorganisms were cut to rectangular pieces with a razor blade and transferred onto a glass slide and fixed with thin 25 µm adhesive tape. A SMALDI Prep spray device (TransMIT GmbH, Germany) was utilized for matrix deposition and formation of uniform matrix layer on the samples. HCCA (alpha-cyano-4-hydroxycinnamic acid, 7 mg/mL) in acetone:water 1:1 (*v:v*) containing 0.1% FA was used as matrix solution and the spray application was performed using the following parameters: 20 µL/min matrix flow rate, 3 L/min gas (N_2_) flow rate, 30 min spray cycle, and 100 rpm sample plate revolution speed. Photos of each sample were created before spraying with the optical microscope Leica S8AP0 (Leica Microsystems GmbH, Germany) or the digital microscope VHX-5000 (Keyence Deutschland GMBH, Germany), respectively.

### MALDI-imaging-HRMS

The MALDI-imaging high resolution mass spectrometry experiments were carried out with an atmospheric pressure scanning microprobe matrix-assisted laser desorption/ionization source (AP-SMALDI; TransMIT GmbH, Germany) coupled to a Q Exactive high-resolution mass spectrometer (Thermo Scientific Inc., Bremen, Germany) following previously published procedures^21,73^, slightly modified. In short, a 60 Hz pulsed N_2_ laser MNL 100 series (LTB Lasertechnik GmbH, Germany) was used for UV beam generation at 337.1 nm. For comparability, scan resolution was adjusted to 40 µm for each sample and measured in full scan positive ion mode at *m/z* 100–1000 mass range with internal lock mass correction utilizing the exact mass of [HCCA + K]^+^ at *m/z* 228.00575. Furthermore, measurements were performed with a mass resolution of 140 000 @ *m/z* 200, S-lens level 65, a spray voltage of 2.0 kV, and an injection time of 200–300 ms. Attenuator value of the AP ion source was set at 20°. MS^2^ measurements of prodigiosin [M + H]^+^ were executed at *m/z* 324.2 ± 0.5 amu with a scan range of *m/z* 100–340, higher-energy collisional dissociation (HCD) with 45 eV and a resolution of 35 000 @ *m/z* 200. Data processing and mapping of mass pixels corresponding to the target compounds was done with the software package ImageQuest (v. 1.1.0; Thermo Fisher Scientific, Germany). Ion images were generated with a bin width of ± 2.0 ppm for full scans and ± 3.0 ppm for MS^2^ experiments. The mass pixels are shown as false colors.

### HPLC-HRMS^n^ and NMR

The extracts were analyzed on a LTQ Orbitrap XL mass spectrometer (Thermo Scientific, USA) and a LTQ Orbitrap mass spectrometer (Thermo Scientific, USA) respectively, both equipped with a HESI-II source coupled to Agilent (Santa Clara, USA) 1200 HPLC system including pump, PDA detector, column oven and auto-sampler. A Luna C18 (2) column (50 × 3 mm, 3 μm particle size) from Phenomenex (Torrance, USA) was used for the HPLC-HRMS and HRMS^n^ measurements with a H_2_O (+0.1% FA) (A) and CH_3_CN (+0.1% FA) (B) gradient (flow rate 350 μL/min). The gradient program was as follows: linear gradient from 5% to 100% B over 28 min, 100% B isocratic for 2 min, the system returned within 0.5 min to initial conditions of 95% A and was equilibrated for 4.5 min. For certain experiments and MS^n^ measurements, the gradient was slightly modified. The mass spectrometer were operated in positive mode with a nominal mass resolving power of 60 000 at *m/z* 400 with a scan rate of 1 Hz under following parameters and N-butyl benzenesulfonamide was used as lock mass ([M + H]^+^ *m/z* 214.08963) for fullscans. He served as collision gas and N_2_ was used as sheath and auxiliary gas. The MS^n^ measurements were performed with collision-induced dissociation with 35 eV (seldom slightly modified).

For the isolation and purification of compounds, 10 Erlenmeyer flasks with 300 mL nutrient broth each were incubated with *S*. *marcescens* MSRBB2 for two weeks under static conditions at 30 °C. The extracts were lyophilized, dissolved in dichloromethane and pre-purified with a short silica gel column. The solvent was evaporated and the extract dissolved in methanol for HPLC-HRMS analysis and further purification by preparative HPLC. We used a Gilson (Middleton, USA) preparative HPLC including a pump (322) and a UV/VIS-152 detector with a Nucleodur C18 Isis column (5 μm particle size, 250 × 16 mm, Macherey-Nagel, Düren, Germany). The gradient (H_2_O (+0.1% FA) (A) and CH_3_CN (+0.1% FA) (B), flow rate 3 mL/min) was as follows: 30% B isocratic for 3 min, linear gradient from 30% to 100% B over 44 min, 100% B isocratic for 10 min, the system returned within 0.5 min to initial conditions of 30% B and was equilibrated for 2.5 min. 1D and 2D NMR spectra were recorded on a Bruker AV 600 and/or 700 Avance III HD (CryoProbe) with deuterated chloroform (prodigiosin, serrawettin W1) or deuterated methanol (serratamic acid).

### Leaf puncture bioassay

The phytotoxicity assay was performed according to previously published methods^74^, suitably modified. Fresh leaves were collected from living plant material from the INFU greenhouse (*M*. *heterophylla* and *M*. *canariensis* originated from South Africa; *M*. *senegalensis* originated from Botanical Garden Berlin^21^). For *M*. *heterophylla* and *M*. *senegalensis*, four replicates of five 2–3 cm leaf sections were prepared with a razor blade. Due to the small size of *M*. *canariensis* leaves, we have taken 40 intact leaves. Each investigated species was needle punctured 40 times. Prodigiosin and serrawettin W1 were dissolved in MeOH and diluted with double-distilled water to a concentration of 5 × 10^−3^ M. As negative control, a blank solution with identical ratios was prepared (H_2_O:MeOH 4:1, *v*:*v*). A droplet of the test solution (1 µL) was applied on the punctures and the samples placed on water-soaked filter paper in Petri dishes, sealed with parafilm and incubated at 25 °C with 12 h intervals of light and dark. The assay was monitored daily and after 3 and 6 days, microscopic images were taken.

### Data availability

All data generated or analyzed during this study are included in this published article (and its Supplementary Information files).

## Electronic supplementary material

Supplementary Information
