# Supplementary material for: Spatial-temporal profiling of prodiginines and serratamolides produced by endophytic Serratia marcescens harbored in Maytenus serrata
Source: Sci Rep. 2018 Mar 27;8:5283. doi: 10.1038/s41598-018-23538-5 (PMC5869619; doi:10.1038/s41598-018-23538-5)
Supplement: Supplementary file 1 — Supplementary Information [file 41598_2018_23538_MOESM1_ESM.pdf]

SCIENTIFIC REPORTS

ELECTRONIC SUPPLEMENTARY INFORMATION

Spatial-temporal profiling of prodiginines and serratamolides produced by endophytic *Serratia marcescens* harbored in *Maytenus serrata*

Dennis Eckelmann, Michael Spiteller, Souvik Kusari*

TABLE OF CONTENTS

I. SUPPLEMENTARY FIGURES

Fig. S1. HRMS^2 of 2-methyl-3-propyl prodiginine (compound **1** $[M+H]^+$: *m/z* 296, $C_{18}H_{22}N_3O$).

Fig. S2. HRMS^2 of 2-methyl-3-butyl prodiginine (compound **2** $[M+H]^+$: *m/z* 310, $C_{19}H_{24}N_3O$).

Fig. S3. HRMS^2 of prodigiosin (compound **3** $[M+H]^+$: *m/z* 324, $C_{20}H_{26}N_3O$).

Fig. S4. HRMS^2 of 2-methyl-3-hexyl prodiginine (compound **4** $[M+H]^+$: *m/z* 338, $C_{21}H_{28}N_3O$).

Fig. S5. HRMS^2 of 2-methyl-3-heptyl prodiginine (compound **5** $[M+H]^+$: *m/z* 352, $C_{22}H_{30}N_3O$).

Fig. S6. HRMS^2 of 2-methyl-3-octyl prodiginine (compound **6** $[M+H]^+$: *m/z* 366, $C_{23}H_{32}N_3O$).

Fig. S7. HRMS^2 of 2-methyl-3-nonyl prodiginine (compound **7** $[M+H]^+$: *m/z* 380, $C_{24}H_{34}N_3O$).

Fig. S8. HRMS^3 of 2-methyl-3-propyl prodiginine (compound **1** $[M+H]^+$: *m/z* 296 via 281).

Fig. S9. HRMS^3 of 2-methyl-3-butyl prodiginine (compound **2** $[M+H]^+$: *m/z* 310 via 295).

Fig. S10. HRMS^3 of prodigiosin (compound **3** $[M+H]^+$: *m/z* 324 via 309).

Fig. S11. HRMS^3 of 2-methyl-3-hexyl prodiginine (compound **4** $[M+H]^+$: *m/z* 338 via 323).

Fig. S12. HRMS^3 of 2-methyl-3-heptyl prodiginine (compound **5** $[M+H]^+$: *m/z* 352 via 337).

Fig. S13. HRMS^3 of 2-methyl-3-octyl prodiginine (compound **6** $[M+H]^+$: *m/z* 366 via 351).

Fig. S14. HRMS^3 of 2-methyl-3-nonyl prodiginine (compound **7** $[M+H]^+$: *m/z* 380 via 365).

Fig. S15. Proposed mass spectral fragmentation pathway of 2-methyl-3-propyl prodiginine (compound **1** $[M+H]^+$: *m/z* 296, $C_{18}H_{22}N_3O$).

Fig. S16. Proposed mass spectral fragmentation pathway of 2-methyl-3-butyl prodiginine (compound **2** $[M+H]^+$: *m/z* 310, $C_{19}H_{24}N_3O$).

Fig. S17. Proposed mass spectral fragmentation pathway of prodigiosin (compound **3** $[M+H]^+$: *m/z* 324, $C_{20}H_{26}N_3O$).

Fig. S18. Proposed mass spectral fragmentation pathway of 2-methyl-3-hexyl prodiginine (compound **4** $[M+H]^+$: *m/z* 338, $C_{21}H_{28}N_3O$).

Fig. S19. Proposed mass spectral fragmentation pathway of 2-methyl-3-heptyl prodiginine (compound **5** $[M+H]^+$: *m/z* 352, $C_{22}H_{30}N_3O$).

Fig. S20. Proposed mass spectral fragmentation pathway of 2-methyl-3-octyl prodiginine (compound **6** $[M+H]^+$: *m/z* 366, $C_{23}H_{32}N_3O$).

Fig. S21. Proposed mass spectral fragmentation pathway of 2-methyl-3-nonyl prodiginine (compound **7** $[M+H]^+$: *m/z* 380, $C_{24}H_{34}N_3O$).

Fig. S22. HPLC-HRMS analysis of prodiginines (**1**-**7**). TIC, extracted ion chromatograms of prodiginines, and area in comparison to the main metabolite prodigiosin.

Fig. S23. HPLC-HRMS analysis of serratamolides (**8**-**14**). TIC, extracted ion chromatograms of serratamolides, and area in comparison to the main metabolite serrawettin W1.

Fig. S24. HPLC-HRMS analysis of serratamolides (**15**-**19**). TIC, extracted ion chromatograms of serratamolides, and area in comparison to the main metabolite serrawettin W1*.

Fig. S25. HPLC-HRMS analysis of serratamolides (**20**-**25**). TIC, extracted ion chromatograms of serratamolides, and area in comparison to the main metabolite serrawettin W1*.

Fig. S26. HPLC-HRMS analysis of serratamolides (**26**-**30**). TIC, extracted ion chromatograms of serratamolides, and area in comparison to the main metabolite serrawettin W1*.

Fig. S27. HPLC-HRMS analysis of serratamolides (**31**-**33**). TIC, extracted ion chromatograms of serratamolides, and area in comparison to the main metabolite serrawettin W1*.

Fig. S28. $HRMS^2$ of serratamolide C_8+C_{10} (compound **8** $[M+H]^+$: *m/z* 487, $C_{24}H_{43}N_2O_8$).

Fig. S29. Proposed mass spectral fragmentation pathway of serratamolide C_8+C_{10} (compound **8** $[M+H]^+$: *m/z* 487, $C_{24}H_{43}N_2O_8$).

Fig. S30. $HRMS^2$ of serratamolide C_9+C_{10} (compound **9** $[M+H]^+$: *m/z* 501, $C_{25}H_{45}N_2O_8$).

Fig. S31. Proposed mass spectral fragmentation pathway of serratamolide C_9+C_{10} (compound **9** $[M+H]^+$: *m/z* 501, $C_{25}H_{45}N_2O_8$).

Fig. S32. $HRMS^2$ of serrawettin W1 / serratamolide C_{10}+C_{10} (compound **10** $[M+H]^+$: *m/z* 515, $C_{26}H_{47}N_2O_8$).

Fig. S33. Proposed mass spectral fragmentation pathway of serrawettin W1 / serratamolide C_{10}+C_{10} (compound **10** $[M+H]^+$: *m/z* 515, $C_{26}H_{47}N_2O_8$).

Fig. S34. HRMS^2 of serratamolide C_{10}+C_{11} (compound **11** $[M+H]^+$: *m/z* 529, $C_{27}H_{49}N_2O_8$).

Fig. S35. Proposed mass spectral fragmentation pathway of serratamolide C_{10}+C_{11} (compound **11** $[M+H]^+$: *m/z* 529, $C_{27}H_{49}N_2O_8$).

Fig. S36. HRMS^2 of serratamolide C_{10}+C_{12} (compound **12** $[M+H]^+$: *m/z* 543, $C_{28}H_{51}N_2O_8$).

Fig. S37. Proposed mass spectral fragmentation pathway of serratamolide C_{10}+C_{12} (compound **12** $[M+H]^+$: *m/z* 543, $C_{28}H_{51}N_2O_8$).

Fig. S38. HRMS^2 of serratamolide C_{11}+C_{12} (compound **13** $[M+H]^+$: *m/z* 557, $C_{29}H_{53}N_2O_8$).

Fig. S39. Proposed mass spectral fragmentation pathway of serratamolide C_{11}+C_{12} (compound **13** $[M+H]^+$: *m/z* 557, $C_{29}H_{53}N_2O_8$).

Fig. S40. HRMS^2 of serratamolide C_{12}+C_{12} (compound **14** $[M+H]^+$: *m/z* 571, $C_{30}H_{55}N_2O_8$).

Fig. S41. Proposed mass spectral fragmentation pathway of serratamolide C_{12}+C_{12} (compound **14** $[M+H]^+$: *m/z* 571, $C_{30}H_{55}N_2O_8$).

Fig. S42. HRMS^2 of serratamolide C_8+$C_{12:1}$ (compound **15** $[M+H]^+$: *m/z* 513, $C_{26}H_{45}N_2O_8$).

Fig. S43. Proposed mass spectral fragmentation pathway of serratamolide C_8+$C_{12:1}$ (compound **15** $[M+H]^+$: *m/z* 513, $C_{26}H_{45}N_2O_8$).

Fig. S44. HRMS^2 of serratamolide C_{10}+$C_{11:1}$ (compound **16** $[M+H]^+$: *m/z* 527, $C_{27}H_{47}N_2O_8$).

Fig. S45. Proposed mass spectral fragmentation pathway of serratamolide C_{10}+$C_{11:1}$ (compound **16** $[M+H]^+$: *m/z* 527, $C_{27}H_{47}N_2O_8$).

Fig. S46. HRMS^2 of serratamolide C_{10}+$C_{12:1}$ (compound **17** $[M+H]^+$: *m/z* 541, $C_{28}H_{49}N_2O_8$).

Fig. S47. Proposed mass spectral fragmentation pathway of serratamolide C_{10}+$C_{12:1}$ (compound **17** $[M+H]^+$: *m/z* 541, $C_{28}H_{49}N_2O_8$).

Fig. S48. HRMS^2 of serratamolide C_{11}+$C_{12:1}$ (compound **18** $[M+H]^+$: *m/z* 555, $C_{29}H_{51}N_2O_8$).

Fig. S49. Proposed mass spectral fragmentation pathway of serratamolide C_{11}+$C_{12:1}$ (compound **18** $[M+H]^+$: *m/z* 555, $C_{29}H_{51}N_2O_8$).

Fig. S50. HRMS^2 of serratamolide C_{12}+$C_{12:1}$ (compound **19** $[M+H]^+$: *m/z* 569, $C_{30}H_{53}N_2O_8$).

Fig. S51. Proposed mass spectral fragmentation pathway of serratamolide C_{12}+$C_{12:1}$ (compound **19** $[M+H]^+$: *m/z* 569, $C_{30}H_{53}N_2O_8$).

Fig. S52. HRMS^2 of ring-opened serratamolide C_{10}+C_{10} (compound **20** $[M+H]^+$: *m/z* 533, $C_{26}H_{49}N_2O_9$).

Fig. S53. Proposed mass spectral fragmentation pathway of ring-opened serratamolide C_{10}+C_{10} (compound **20** $[M+H]^+$: *m/z* 533, $C_{26}H_{49}N_2O_9$).

Fig. S54. HRMS^2 of ring-opened serratamolide C_{10}+C_{11} (compound **21** $[M+H]^+$: *m/z* 547, $C_{27}H_{51}N_2O_9$).

Fig. S55. Proposed mass spectral fragmentation pathway of ring-opened serratamolide C_{10}+C_{11} (compound **21** $[M+H]^+$: *m/z* 547, $C_{27}H_{51}N_2O_9$).

Fig. S56. HRMS^2 of ring-opened serratamolide C_{10}+C_{12} (compound **22** $[M+H]^+$: *m/z* 561, $C_{28}H_{53}N_2O_9$).

Fig. S57. Proposed mass spectral fragmentation pathway of ring-opened serratamolide C_{10}+C_{12} (compound **22** $[M+H]^+$: *m/z* 561, $C_{28}H_{53}N_2O_9$).

Fig. S58. HRMS^2 of ring-opened serratamolide C_{10}+$C_{12:1}$ (compound **23** $[M+H]^+$: *m/z* 559, $C_{28}H_{51}N_2O_9$).

Fig. S59. Proposed mass spectral fragmentation pathway of ring-opened serratamolide C_{10}+$C_{12:1}$ (compound **23** $[M+H]^+$: *m/z* 559, $C_{28}H_{51}N_2O_9$).

Fig. S60. HRMS^2 of ring-opened serratamolide C_9+C_{10} (compound **24** $[M+H]^+$: *m/z* 519, $C_{25}H_{47}N_2O_9$).

Fig. S61. Proposed mass spectral fragmentation pathway of ring-opened serratamolide C_9+C_{10} (compound **24** $[M+H]^+$: *m/z* 519, $C_{25}H_{47}N_2O_9$).

Fig. S62. HRMS^2 of ring-opened serratamolide C_{10}+C_{13} (compound **25** $[M+H]^+$: *m/z* 575, $C_{29}H_{55}N_2O_9$).

Fig. S63. Proposed mass spectral fragmentation pathway of ring-opened serratamolide C_{10}+C_{13} (compound **25** $[M+H]^+$: *m/z* 575, $C_{29}H_{55}N_2O_9$).

Fig. S64. HRMS^2 of ring-opened serratamolide C_{12}+C_{12} (compound **26** $[M+H]^+$: *m/z* 589, $C_{30}H_{57}N_2O_9$).

Fig. S65. Proposed mass spectral fragmentation pathway of ring-opened serratamolide C_{12}+C_{12} (compound **26** $[M+H]^+$: *m/z* 589, $C_{30}H_{57}N_2O_9$).

Fig. S66. HRMS^2 of ring-opened serratamolide C_{12}+C_{13} (compound **27** $[M+H]^+$: *m/z* 603, $C_{31}H_{59}N_2O_9$).

Fig. S67. Proposed mass spectral fragmentation pathway of ring-opened serratamolide C_{12}+C_{13} (compound **27** $[M+H]^+$: *m/z* 603, $C_{31}H_{59}N_2O_9$).

Fig. S68. HRMS^2 of ring-opened serratamolide C_{10}+$C_{13:1}$ (compound **28** $[M+H]^+$: *m/z* 573, $C_{29}H_{53}N_2O_9$).

Fig. S69. HRMS^2 of ring-opened serratamolide C_{12}+$C_{12:1}$ (compound **29** $[M+H]^+$: *m/z* 587, $C_{30}H_{55}N_2O_9$).

Fig. S70. HRMS^2 of ring-opened serratamolide C_{13}+$C_{12:1}$ (compound **30** $[M+H]^+$: *m/z* 601, $C_{31}H_{57}N_2O_9$).

Fig. S71. HRMS^2 of serratamic acid (N-(D-3-hydroxydecanoyl)-L-serine, compound **31** $[M+H]^+$: *m/z* 276, $C_{13}H_{26}NO_5$).

Fig. S72. HRMS^3 of serratamic acid (N-(D-3-hydroxydecanoyl)-L-serine, compound **31** $[M+H]^+$: *m/z* 276 via 258).

Fig. S73. Proposed mass spectral fragmentation pathway of serratamic acid (N-(D-3-hydroxydecanoyl)-L-serine, compound **31**$[M+H]^+$: *m/z* 276, $C_{13}H_{26}NO_5$).

Fig. S74. HRMS^2 of serratamic acid derivative (hydroxydodecanoyl-serine, compound **32** $[M+H]^+$: *m/z* 304, $C_{15}H_{30}NO_5$).

Fig. S75. HRMS^3 of serratamic acid derivative (hydroxydodecanoyl-serine, compound **32** $[M+H]^+$: *m/z* 304 via 286).

Fig. S76. Proposed mass spectral fragmentation pathway of serratamic acid derivative (hydroxydodecanoyl-serine, compound **32** $[M+H]^+$: *m/z* 304, $C_{15}H_{30}NO_5$).

Fig. S77. HRMS^2 of serratamic acid derivative with double bond (hydroxydodecenoyl-serine, compound **33** $[M+H]^+$: *m/z* 302, $C_{15}H_{28}NO_5$).

Fig. S78. HRMS^3 of of serratamic acid derivative with double bond (hydroxydodecenoyl-serine, compound **33** $[M+H]^+$: *m/z* 302 via 284).

Fig. S79. Proposed mass spectral fragmentation pathway of serratamic acid derivative with double bond (hydroxydodecenoyl-serine, compound **33** $[M+H]^+$: *m/z* 302, $C_{15}H_{28}NO_5$).

Fig. S80. ^{1}H-NMR spectrum of serrawettin W1 / serratamolide C_{10}+C_{10} (compound **10**, $CDCl_3$, 600 MHz).

Fig. S81. ^{13}C-NMR spectrum of serrawettin W1 / serratamolide C_{10}+C_{10} (compound **10**, $CDCl_3$, 150 MHz).

Fig. S82. COSY NMR spectrum of serrawettin W1 / serratamolide C_{10}+C_{10} (compound **10**, $CDCl_3$).

Fig. S83. HSQC NMR spectrum of serrawettin W1 / serratamolide C_{10}+C_{10} (compound **10**, $CDCl_3$).

Fig. S84. HMBC NMR spectrum of serrawettin W1 / serratamolide C_{10}+C_{10} (compound **10**, $CDCl_3$).

Fig. S85. ^{1}H-NMR spectrum of serratamic acid (N-(D-3-hydroxydecanoyl)-L-serine, compound **31**, MeOD, 700 MHz).

Fig. S86. ^{13}C-NMR spectrum of serratamic acid (N-(D-3-hydroxydecanoyl)-L-serine, compound **31**, MeOD, 175 MHz).

Fig. S87. COSY NMR spectrum of serratamic acid (N-(D-3-hydroxydecanoyl)-L-serine, compound **31**, MeOD).

Fig. S88. HSQC NMR spectrum of serratamic acid (N-(D-3-hydroxydecanoyl)-L-serine, compound **31**, MeOD).

Fig. S89. HMBC NMR spectrum of serratamic acid (N-(D-3-hydroxydecanoyl)-L-serine, compound **31**, MeOD).

Fig. S90. ^{1}H-NMR spectrum of prodigiosin (compound **3**, $CDCl_3$, 600 MHz).

Fig. S91. COSY NMR spectrum of prodigiosin (compound **3**, $CDCl_3$, 600 MHz).

Fig. S92. HRMS spectra of 2-methyl-3-propyl prodiginine (compound **1** $[M+H]^+$: *m/z* 296, $C_{18}H_{22}N_3O$).

Fig. S93. HRMS spectra of 2-methyl-3-butyl prodiginine (compound **2** $[M+H]^+$: *m/z* 310, $C_{19}H_{24}N_3O$).

Fig. S94. HRMS spectra of prodigiosin (compound **3** $[M+H]^+$: *m/z* 324, $C_{20}H_{26}N_3O$).

Fig. S95. HRMS spectra of 2-methyl-3-hexyl prodiginine (compound **4** $[M+H]^+$: *m/z* 338, $C_{21}H_{28}N_3O$).

Fig. S96. HRMS spectra of 2-methyl-3-heptyl prodiginine (compound **5** $[M+H]^+$: *m/z* 352, $C_{22}H_{30}N_3O$).

Fig. S97. HRMS spectra of 2-methyl-3-octyl prodiginine (compound **6** $[M+H]^+$: *m/z* 366, $C_{23}H_{32}N_3O$).

Fig. S98. HRMS spectra of 2-methyl-3-nonyl prodiginine (compound **7** $[M+H]^+$: *m/z* 380, $C_{24}H_{34}N_3O$).

Fig. S99. HRMS spectra of 2-methyl-3-propyl prodiginine with incorporated ^{15}N derived from $^{15}NH_4Cl$ (compound **1** $[M+H]^+$: *m/z* 297, $C_{18}H_{22}N_2{}^{15}NO$).

Fig. S100. HRMS spectra of 2-methyl-3-butyl prodiginine with incorporated ^{15}N derived from $^{15}NH_4Cl$ (compound **2** $[M+H]^+$: *m/z* 311, $C_{19}H_{24}N_2{}^{15}NO$).

Fig. S101. HRMS spectra of prodigiosin with incorporated ^{15}N derived from $^{15}NH_4Cl$ (compound **3** $[M+H]^+$: *m/z* 325, $C_{20}H_{26}N_2{}^{15}NO$).

Fig. S102. HRMS spectra of 2-methyl-3-hexyl prodiginine with incorporated ^{15}N derived from $^{15}NH_4Cl$ (compound **4** $[M+H]^+$: *m/z* 339, $C_{21}H_{28}N_2{}^{15}NO$).

Fig. S103. HRMS spectra of 2-methyl-3-heptyl prodiginine with incorporated ^{15}N derived from $^{15}NH_4Cl$ (compound **5** $[M+H]^+$: *m/z* 353, $C_{22}H_{30}N_2{}^{15}NO$).

Fig. S104. HRMS spectra of 2-methyl-3-nonyl prodiginine with incorporated ^{15}N derived from $^{15}NH_4Cl$ (compound **7** $[M+H]^+$: *m/z* 381, $C_{24}H_{34}N_2{}^{15}NO$).

Fig. S105. HRMS spectra of 2-methyl-3-propyl prodiginine with incorporated methyl-D_3 group derived from [methyl-D_3]-L-methionine (compound **1** $[M+H]^+$: *m/z* 299, $C_{18}H_{19}D_3N_3O$).

Fig. S106. HRMS spectra of 2-methyl-3-butyl prodiginine with incorporated methyl-D_3 group derived from [methyl-D_3]-L-methionine (compound **2** $[M+H]^+$: *m/z* 313, $C_{19}H_{21}D_3N_3O$).

Fig. S107. HRMS spectra of prodigiosin with incorporated methyl-D_3 group derived from [methyl-D_3]-L-methionine (compound **3** $[M+H]^+$: *m/z* 327, $C_{20}H_{23}D_3N_3O$).

Fig. S108. HRMS spectra of 2-methyl-3-hexyl prodiginine with incorporated methyl-D_3 group derived from [methyl-D_3]-L-methionine (compound **4** $[M+H]^+$: *m/z* 341, $C_{21}H_{25}D_3N_3O$).

Fig. S109. HRMS spectra of 2-methyl-3-heptyl prodiginine with incorporated methyl-D_3 group derived from [methyl-D_3]-L-methionine (compound **5** $[M+H]^+$: *m/z* 355, $C_{22}H_{27}D_3N_3O$).

Fig. S110. HRMS spectra of 2-methyl-3-nonyl prodiginine with incorporated methyl-D_3 group derived from [methyl-D_3]-L-methionine (compound **7** $[M+H]^+$: *m/z* 383, $C_{24}H_{31}D_3N_3O$).

Fig. S111. HRMS spectra of 2-methyl-3-propyl prodiginine with incorporated [1-^{13}C]-L-proline (compound **1** $[M+H]^+$: *m/z* 297, $C_{17}{}^{13}CH_{22}N_3O$).

Fig. S112. HRMS spectra of 2-methyl-3-butyl prodiginine with incorporated [1-^{13}C]-L-proline (compound **2** $[M+H]^+$: *m/z* 311, $C_{18}{}^{13}CH_{24}N_3O$).

Fig. S113. HRMS spectra of prodigiosin with incorporated [1-^{13}C]-L-proline (compound **3** $[M+H]^+$: *m/z* 325, $C_{19}{}^{13}CH_{26}N_3O$).

Fig. S114. HRMS spectra of 2-methyl-3-hexyl prodiginine with incorporated [1-^{13}C]-L-proline (compound **4** $[M+H]^+$: *m/z* 339, $C_{20}{}^{13}CH_{28}N_3O$).

Fig. S115. HRMS spectra of 2-methyl-3-heptyl prodiginine with incorporated [1-^{13}C]-L-proline (compound **5** $[M+H]^+$: *m/z* 353, $C_{21}{}^{13}CH_{30}N_3O$).

Fig. S116. HRMS spectra of 2-methyl-3-nonyl prodiginine with incorporated [1-^{13}C]-L-proline (compound **7** $[M+H]^+$: *m/z* 381, $C_{23}{}^{13}CH_{34}N_3O$).

Fig. S117. HRMS^2 of 2-methyl-3-propyl prodiginine with incorporated ^{15}N derived from $^{15}NH_4Cl$ (compound **1** $[M+H]^+$: *m/z* 297, $C_{18}H_{22}N_2{}^{15}NO$).

Fig. S118. HRMS^2 of 2-methyl-3-butyl prodiginine with incorporated ^{15}N derived from $^{15}NH_4Cl$ (compound **2** $[M+H]^+$: *m/z* 311, $C_{19}H_{24}N_2{}^{15}NO$).

Fig. S119. HRMS^2 of prodigiosin with incorporated ^{15}N derived from $^{15}NH_4Cl$ (compound **3** $[M+H]^+$: *m/z* 325, $C_{20}H_{26}N_2{}^{15}NO$).

Fig. S120. HRMS^2 of 2-methyl-3-hexyl prodiginine with incorporated ^{15}N derived from $^{15}NH_4Cl$ (compound **4** $[M+H]^+$: *m/z* 339, $C_{21}H_{28}N_2{}^{15}NO$).

Fig. S121. HRMS^2 of 2-methyl-3-heptyl prodiginine with incorporated ^{15}N derived from $^{15}NH_4Cl$ (compound **5** $[M+H]^+$: *m/z* 353, $C_{22}H_{30}N_2{}^{15}NO$).

Fig. S122. HRMS^2 of 2-methyl-3-nonyl prodiginine with incorporated ^{15}N derived from $^{15}NH_4Cl$ (compound **7** $[M+H]^+$: *m/z* 381, $C_{24}H_{34}N_2{}^{15}NO$).

Fig. S123. HRMS^3 of 2-methyl-3-propyl prodiginine with incorporated ^{15}N derived from $^{15}NH_4Cl$ (compound **1** $[M+H]^+$: *m/z* 297 via 272, $C_{18}H_{22}N_2{}^{15}NO$).

Fig. S124. HRMS^3 of 2-methyl-3-butyl prodiginine with incorporated ^{15}N derived from $^{15}NH_4Cl$ (compound **2** $[M+H]^+$: *m/z* 311 via 296, $C_{19}H_{24}N_2{}^{15}NO$).

Fig. S125. HRMS^3 of prodigiosin with incorporated ^{15}N derived from $^{15}NH_4Cl$ (compound **3** $[M+H]^+$: *m/z* 325 via 310, $C_{20}H_{26}N_2{}^{15}NO$).

Fig. S126. HRMS^3 of 2-methyl-3-hexyl prodiginine with incorporated ^{15}N derived from $^{15}NH_4Cl$ (compound **4** $[M+H]^+$: *m/z* 339 via 324, $C_{21}H_{28}N_2{}^{15}NO$).

Fig. S127. HRMS^3 of 2-methyl-3-heptyl prodiginine with incorporated ^{15}N derived from $^{15}NH_4Cl$ (compound **5** $[M+H]^+$: *m/z* 353 via 338, $C_{22}H_{30}N_2{}^{15}NO$).

Fig. S128. HRMS^3 of 2-methyl-3-nonyl prodiginine with incorporated ^{15}N derived from $^{15}NH_4Cl$ (compound **7** $[M+H]^+$: *m/z* 381 via 366, $C_{24}H_{34}N_2{}^{15}NO$).

Fig. S129. Proposed mass spectral fragmentation pathway of 2-methyl-3-propyl prodiginine with incorporated ^{15}N derived from $^{15}NH_4Cl$ (compound **1** $[M+H]^+$: *m/z* 297, $C_{18}H_{22}N_2{}^{15}NO$).

Fig. S130. Proposed mass spectral fragmentation pathway of 2-methyl-3-butyl prodiginine with incorporated ^{15}N derived from $^{15}NH_4Cl$ (compound **2** $[M+H]^+$: *m/z* 311, $C_{19}H_{24}N_2{}^{15}NO$).

Fig. S131. Proposed mass spectral fragmentation pathway of prodigiosin with incorporated ^{15}N derived from $^{15}NH_4Cl$ (compound **3** $[M+H]^+$: *m/z* 325, $C_{20}H_{26}N_2{}^{15}NO$).

Fig. S132. Proposed mass spectral fragmentation pathway of 2-methyl-3-hexyl prodiginine with incorporated ^{15}N derived from $^{15}NH_4Cl$ (compound **4** $[M+H]^+$: *m/z* 339, $C_{21}H_{28}N_2{}^{15}NO$).

Fig. S133. Proposed mass spectral fragmentation pathway of 2-methyl-3-heptyl prodiginine with incorporated ^{15}N derived from $^{15}NH_4Cl$ (compound **5** $[M+H]^+$: *m/z* 353, $C_{22}H_{30}N_2{}^{15}NO$).

Fig. S134. Proposed mass spectral fragmentation pathway of 2-methyl-3-nonyl prodiginine with incorporated ^{15}N derived from $^{15}NH_4Cl$ (compound **7** $[M+H]^+$: *m/z* 381 via 366, $C_{24}H_{34}N_2{}^{15}NO$).

Fig. S135. $HRMS^2$ of 2-methyl-3-propyl prodiginine with incorporated methyl-D_3 group derived from [methyl-D_3]-L-methionine (compound **1** $[M+H]^+$: *m/z* 299, $C_{18}H_{19}D_3N_3O$).

Fig. S136. $HRMS^2$ of 2-methyl-3-butyl prodiginine with incorporated methyl-D_3 group derived from [methyl-D_3]-L-methionine (compound **2** $[M+H]^+$: *m/z* 313, $C_{19}H_{21}D_3N_3O$).

Fig. S137. $HRMS^2$ of prodigiosin with incorporated methyl-D_3 group derived from [methyl-D_3]-L-methionine (compound **3** $[M+H]^+$: *m/z* 327, $C_{20}H_{23}D_3N_3O$).

Fig. S138. $HRMS^2$ of 2-methyl-3-hexyl prodiginine with incorporated methyl-D_3 group derived from [methyl-D_3]-L-methionine (compound **4** $[M+H]^+$: *m/z* 341, $C_{21}H_{25}D_3N_3O$).

Fig. S139. $HRMS^2$ of 2-methyl-3-heptyl prodiginine with incorporated methyl-D_3 group derived from [methyl-D_3]-L-methionine (compound **5** $[M+H]^+$: *m/z* 355, $C_{22}H_{27}D_3N_3O$).

Fig. S140. $HRMS^2$ of 2-methyl-3-nonyl prodiginine with incorporated methyl-D_3 group derived from [methyl-D_3]-L-methionine (compound **7** $[M+H]^+$: *m/z* 383, $C_{24}H_{31}D_3N_3O$).

Fig. S141. $HRMS^3$ of 2-methyl-3-propyl prodiginine with incorporated methyl-D_3 group derived from [methyl-D_3]-L-methionine (compound **1** $[M+H]^+$: *m/z* 299 via 281, $C_{18}H_{19}D_3N_3O$).

Fig. S142. $HRMS^3$ of 2-methyl-3-butyl prodiginine with incorporated methyl-D_3 group derived from [methyl-D_3]-L-methionine (compound **2** $[M+H]^+$: *m/z* 313 via 295, $C_{19}H_{21}D_3N_3O$).

Fig. S143. $HRMS^3$ of prodigiosin with incorporated methyl-D_3 group derived from [methyl-D_3]-L-methionine (compound **3** $[M+H]^+$: *m/z* 327 via 309, $C_{20}H_{23}D_3N_3O$).

Fig. S144. $HRMS^3$ of 2-methyl-3-hexyl prodiginine with incorporated methyl-D_3 group derived from [methyl-D_3]-L-methionine (compound **4** $[M+H]^+$: *m/z* 341 via 323, $C_{21}H_{25}D_3N_3O$).

Fig. S145. $HRMS^3$ of 2-methyl-3-heptyl prodiginine with incorporated methyl-D_3 group derived from [methyl-D_3]-L-methionine (compound **5** $[M+H]^+$: *m/z* 355 via 337, $C_{22}H_{27}D_3N_3O$).

Fig. S146. HRMS^3 of 2-methyl-3-nonyl prodiginine with incorporated methyl-D_3 group derived from [methyl-D_3]-L-methionine (compound **7** $[M+H]^+$: *m/z* 383 via 366, $C_{24}H_{31}D_3N_3O$).

Fig. S147. Proposed mass spectral fragmentation pathway of 2-methyl-3-propyl prodiginine with incorporated methyl-D_3 group derived from [methyl-D_3]-L-methionine (compound **1** $[M+H]^+$: *m/z* 299, $C_{18}H_{19}D_3N_3O$).

Fig. S148. Proposed mass spectral fragmentation pathway of 2-methyl-3-butyl prodiginine with incorporated methyl-D_3 group derived from [methyl-D_3]-L-methionine (compound **2** $[M+H]^+$: *m/z* 313, $C_{19}H_{21}D_3N_3O$).

Fig. S149. Proposed mass spectral fragmentation pathway of prodigiosin with incorporated methyl-D_3 group derived from [methyl-D_3]-L-methionine (compound **3** $[M+H]^+$: *m/z* 327, $C_{20}H_{23}D_3N_3O$).

Fig. S150. Proposed mass spectral fragmentation pathway of 2-methyl-3-hexyl prodiginine with incorporated methyl-D_3 group derived from [methyl-D_3]-L-methionine (compound **4** $[M+H]^+$: *m/z* 341, $C_{21}H_{25}D_3N_3O$).

Fig. S151. Proposed mass spectral fragmentation pathway of 2-methyl-3-heptyl prodiginine with incorporated methyl-D_3 group derived from [methyl-D_3]-L-methionine (compound **5** $[M+H]^+$: *m/z* 355, $C_{22}H_{27}D_3N_3O$).

Fig. S152. Proposed mass spectral fragmentation pathway of 2-methyl-3-nonyl prodiginine with incorporated methyl-D_3 group derived from [methyl-D_3]-L-methionine (compound **7** $[M+H]^+$: *m/z* 383, $C_{24}H_{31}D_3N_3O$).

Fig. S153. HRMS^2 of 2-methyl-3-propyl prodiginine with incorporated [1-^{13}C]-L-proline (compound **1** $[M+H]^+$: *m/z* 297, $C_{17}{}^{13}CH_{22}N_3O$).

Fig. S154. HRMS^2 of 2-methyl-3-butyl prodiginine with incorporated [1-^{13}C]-L-proline (compound **2** $[M+H]^+$: *m/z* 311, $C_{18}{}^{13}CH_{24}N_3O$).

Fig. S155. HRMS^2 of prodigiosin with incorporated [1-^{13}C]-L-proline (compound **3** $[M+H]^+$: *m/z* 325, $C_{19}{}^{13}CH_{26}N_3O$).

Fig. S156. HRMS^2 of 2-methyl-3-hexyl prodiginine with incorporated [1-^{13}C]-L-proline (compound **4** $[M+H]^+$: *m/z* 339, $C_{20}{}^{13}CH_{28}N_3O$).

Fig. S157. HRMS^2 of 2-methyl-3-heptyl prodiginine with incorporated [1-^{13}C]-L-proline (compound **5** $[M+H]^+$: *m/z* 353, $C_{21}{}^{13}CH_{30}N_3O$).

Fig. S158. HRMS^2 of 2-methyl-3-nonyl prodiginine with incorporated [1-^{13}C]-L-proline (compound **7** $[M+H]^+$: *m/z* 381, $C_{23}{}^{13}CH_{34}N_3O$).

Fig. S159. HRMS^3 of 2-methyl-3-propyl prodiginine with incorporated [1-^{13}C]-L-proline (compound **1** $[M+H]^+$: *m/z* 297 via 282, $C_{17}{}^{13}CH_{22}N_3O$).

Fig. S160. HRMS^3 of 2-methyl-3-butyl prodiginine with incorporated [1-^{13}C]-L-proline (compound **2** $[M+H]^+$: *m/z* 311 via 296, $C_{18}{}^{13}CH_{24}N_3O$).

Fig. S161. HRMS^3 of prodigiosin with incorporated [1-^{13}C]-L-proline (compound **3** $[M+H]^+$: *m/z* 325 via 310, $C_{19}{}^{13}CH_{26}N_3O$).

Fig. S162. HRMS^3 of 2-methyl-3-hexyl prodiginine with incorporated [1-^{13}C]-L-proline (compound **4** $[M+H]^+$: *m/z* 339 via 324, $C_{20}{}^{13}CH_{28}N_3O$).

Fig. S163. HRMS^3 of 2-methyl-3-heptyl prodiginine with incorporated [1-^{13}C]-L-proline (compound **5** $[M+H]^+$: *m/z* 353 via 338, $C_{21}{}^{13}CH_{30}N_3O$).

Fig. S164. HRMS^3 of 2-methyl-3-nonyl prodiginine with incorporated [1-^{13}C]-L-proline (compound **7** $[M+H]^+$: *m/z* 381 via 366, $C_{23}{}^{13}CH_{34}N_3O$).

Fig. S165. Proposed mass spectral fragmentation pathway of 2-methyl-3-propyl prodiginine with incorporated [1-^{13}C]-L-proline (compound **1** $[M+H]^+$: *m/z* 297, $C_{17}{}^{13}CH_{22}N_3O$).

Fig. S166. Proposed mass spectral fragmentation pathway of 2-methyl-3-butyl prodiginine with incorporated [1-^{13}C]-L-proline (compound **2** $[M+H]^+$: *m/z* 311, $C_{18}{}^{13}CH_{24}N_3O$).

Fig. S167. Proposed mass spectral fragmentation pathway of prodigiosin with incorporated [1-^{13}C]-L-proline (compound **3** $[M+H]^+$: *m/z* 325, $C_{19}{}^{13}CH_{26}N_3O$).

Fig. S168. Proposed mass spectral fragmentation pathway of 2-methyl-3-hexyl prodiginine with incorporated [1-^{13}C]-L-proline (compound **4** $[M+H]^+$: *m/z* 339, $C_{20}{}^{13}CH_{28}N_3O$).

Fig. S169. Proposed mass spectral fragmentation pathway of 2-methyl-3-heptyl prodiginine with incorporated [1-^{13}C]-L-proline (compound **5** $[M+H]^+$: *m/z* 353, $C_{21}{}^{13}CH_{30}N_3O$).

Fig. S170. Proposed mass spectral fragmentation pathway of 2-methyl-3-nonyl prodiginine with incorporated [1-^{13}C]-L-proline (compound **7** $[M+H]^+$: *m/z* 381, $C_{23}{}^{13}CH_{34}N_3O$).

Fig. S171. HRMS^2 of prodigiosin with 5 incorporated [1,2-$^{13}C_2$]-acetate units (compound **3** $[M+H]^+$: *m/z* 334, $C_{10}{}^{13}C_{10}H_{26}N_3O$).

Fig. S172. HRMS^2 of prodigiosin with 6 incorporated [1,2-$^{13}C_2$]-acetate units (compound **3** $[M+H]^+$: *m/z* 336, $C_8{}^{13}C_{12}H_{26}N_3O$).

Fig. S173. HRMS^2 of prodigiosin with 7 incorporated [1,2-$^{13}C_2$]-acetate units (compound **3** $[M+H]^+$: *m/z* 338, $C_6{}^{13}C_{14}H_{26}N_3O$).

Fig. S174. Confrontation assay of endophytic *Pichia* spp. against *S. aureus* (DSM 799) and *E. coli* (DSM 682).

Fig. S175. Confrontation assay of endophytic *A. caesiellus* against *S. aureus* (DSM 799) and *E. coli* (DSM 682).

Fig. S176. Confrontation assay of endophytic *P. virgatula* against *S. aureus* (DSM 799) and *E. coli* (DSM 682).

Fig. S177. Scanning electron microscopic images of endophytic *S. marcescens* MSRBB2.

Fig. S178. Scanning electron microscopic images of endophytic *P. virgatula*.

Fig. S179. Scanning electron microscopic images (1) of endophytic *A. caesiellus*.

Fig. S180. Scanning electron microscopic images (2) of endophytic *A. caesiellus*.

Fig. S181. Scanning electron microscopic images (1) of endophytic *Pichia* spp.

Fig. S182. Scanning electron microscopic images (2) of endophytic *Pichia* spp.

Fig. S183. Scanning electron microscopic images (1) of co-cultivation of endophytic *P. virgatula* and *S. marcescens* MSRBB2.

Fig. S184. Scanning electron microscopic images (2) of co-cultivation of endophytic *P. virgatula* and *S. marcescens* MSRBB2.

Fig. S185. Scanning electron microscopic images (3) of co-cultivation of endophytic *P. virgatula* and *S. marcescens* MSRBB2.

Fig. S186. Scanning electron microscopic images (1) of co-cultivation of endophytic *A. caesiellus* and *S. marcescens* MSRBB2.

Fig. S187. Scanning electron microscopic images (2) of co-cultivation of endophytic *A. caesiellus* and *S. marcescens* MSRBB2.

Fig. S189. Scanning electron microscopic images (3) of co-cultivation of endophytic *A. caesiellus* and *S. marcescens* MSRBB2.

Fig. S190. Scanning electron microscopic images (1) of co-cultivation of endophytic *Pichia* spp. and *S. marcescens* MSRBB2.

Fig. S191. Scanning electron microscopic images (2) of co-cultivation of endophytic *Pichia* spp. and *S. marcescens* MSRBB2.

Fig. S192. Scanning electron microscopic images of *Aspergillus fumigatus* (DSM 21023).

Fig. S193. Scanning electron microscopic images of co-cultivation of endophytic *S. marcescens* MSRBB2 and *Aspergillus fumigatus* (DSM 21023).

Fig. S194. Scanning electron microscopic images of *Pichia membranifaciens* (DSM 70366).

Fig. S195. Scanning electron microscopic images of co-cultivation of endophytic *S. marcescens* MSRBB2 and *Pichia membranifaciens* (DSM 70366).

Fig. S196. Scanning electron microscopic images of *Pestalotiopsis versicolor* (DSM 62887).

Fig. S197. Scanning electron microscopic images of co-cultivation of endophytic *S. marcescens* MSRBB2 and *Pestalotiopsis versicolor* (DSM 62887).

Fig. S198. Dose and time dependent disc line assay of different prodigiosin concentrations against *A. caesiellus*

Fig. S199. Dose and time dependent Y-shape disc assay of different prodigiosin concentrations against *A. caesiellus*

Fig. S200. Dose and time dependent disc line assay of different prodigiosin concentrations against *Pichia* spp.

Fig. S201. Dose and time dependent Y-shape disc assay of different prodigiosin concentrations against *Pichia* spp.

Fig. S202. Dose and time dependent disc line assay of different prodigiosin concentrations against *P. virgatula*

Fig. S203. Dose and time dependent Y-shape disc assay of different prodigiosin concentrations against *P. virgatula*

Fig. S204. *S. marcescens* MSRBB2 on chitinase detection agar incubated at 30 °C and monitored for 14 days. Chitinase detection media[1] (A) showing degradation of chitin by chitinase enzymes resulting in pH change and color change of Bromocresol purple dye. Chitinase detection media with supplemented glucose (20 g/L, B) showing no chitinase enzyme expression. Glucose is inhibiting chitinase expression.

Fig. S205. Digital microscope images with different magnifications of the colonization of endophytic *A. caesiellus* by *S. marcescens* MSRBB2. Hyphae of the fungus is overgrown with *S. marcescens* MSRBB2 colonies.

Fig. S206. MALDI-imaging-HRMS of the co-cultivation of bacterial endophyte *S. marcescens* MSRBB2 and fungal endophyte *A. caesiellus* after 3 days. Optical image with assigned scan area and localization of prodiginines ($[M+H]^+$ ± 2 ppm, (A)). Different fragments of MALDI-imaging-HRMS^2 of prodigiosin (B).

Fig. S207. MALDI-imaging-HRMS of the co-cultivation of bacterial endophyte *S. marcescens* MSRBB2 and fungal endophyte *A. caesiellus* after 3 days. Optical image with assigned scan area and localization of prodiginines ($[M+H]^+$ ± 2 ppm).

Fig. S208. MALDI-imaging-HRMS of the co-cultivation of bacterial endophyte *S. marcescens* MSRBB2 and fungal endophyte *A. caesiellus* after 5 days. Optical image with assigned scan area and localization of prodiginines ($[M+H]^+$ ± 2 ppm).

Fig. S209. MALDI-imaging-HRMS of the co-cultivation of bacterial endophyte *S. marcescens* MSRBB2 and fungal endophyte *A. caesiellus* after 7 days. Optical image with assigned scan area and localization of prodiginines ($[M+H]^+$ ± 2 ppm, (A)). Different fragments of MALDI-imaging-HRMS^2 of prodigiosin (B).

Fig. S210. MALDI-imaging-HRMS of the co-cultivation of bacterial endophyte *S. marcescens* MSRBB2 and fungal endophyte *A. caesiellus* after 7 days. Optical image with assigned scan area and localization of prodiginines ($[M+H]^+$ ± 2 ppm, (A)). Different fragments of MALDI-imaging-HRMS^2 of prodigiosin (B).

Fig. S211. Images of solo cultivation of endophytic *S. marcescens* MSRBB2 (Control).

Fig. S212. MALDI-imaging-HRMS of solo cultivation of bacterial endophyte *S. marcescens* MSRBB2 after 7 days. Optical image with assigned scan area and localization of prodiginines **1**-**5** ($[M+H]^+$ ± 2 ppm).

Fig. S213. HPLC-HRMS comparison of prodigiosin (Extracted ion chromatograms, $[M+H]^+$ ± 2 ppm) production by endophytic *S. marcescens* MSRBB2 of mono-cultivation and co-cultivations with fungal endophytes *A. caesiellus* and *Pichia* spp. (five Petri dishes) after 7 days incubation (30°C).

Fig. S214. HPLC-HRMS of extracts of bacterial endophyte *S. marcescens* MSRBB2 incubated at 30°C and 37°C for 24h, optical images of incubated nutrient agar Petri dishes, and extracted ion chromatograms of serrawettin w1 ($[M+H]^+$ ± 2 ppm) (A) Enlarged microscopic images (B).

Fig. S215. MALDI-imaging-HRMS of the co-cultivation of bacterial endophyte *S. marcescens* MSRBB2 and fungal endophyte *A. caesiellus* after 3 days. Optical image with assigned scan area and localization of serratamolides ($[M+K]^+$ ± 2 ppm).

Fig. S216. MALDI-imaging-HRMS of the co-cultivation of bacterial endophyte *S. marcescens* MSRBB2 and fungal endophyte *A. caesiellus* after 5 days. Optical image with assigned scan area and localization of serratamolides ($[M+K]^+$ ± 2 ppm).

Fig. S217. MALDI-imaging-HRMS of the co-cultivation of bacterial endophyte *S. marcescens* MSRBB2 and fungal endophyte *A. caesiellus* after 7 days. Optical image with assigned scan area and localization of serratamolides ($[M+K]^+$ ± 2 ppm).

Fig. S218. MALDI-imaging-HRMS of the co-cultivation of bacterial endophyte *S. marcescens* MSRBB2 and fungal endophyte *A. caesiellus* after 7 days. Optical image with assigned scan area and localization of serratamolides ($[M+K]^+$ ± 2 ppm).

Fig. S219. MALDI-imaging-HRMS of solo cultivation of bacterial endophyte *S. marcescens* MSRBB2 after 7 days. Optical image with assigned scan area and localization of serratamolides ($[M+K]^+$ ± 2 ppm).

Fig. S220. MALDI-imaging-HRMS of solo cultivation of bacterial endophyte *S. marcescens* MSRBB2 after 7 days. Optical image with assigned scan area and localization of serratamolides ($[M+K]^+$ ± 2 ppm).

Fig. S221. Possible decomposition of serratamolides by hydrolysis resulting in open-ring serratamolides and/ or “monomers”.

Fig. S222. Digital microscope images of leaf puncture bioassay of *M. canariensis*, *M. heterophylla*, and *M. senegalensis* against prodigiosin (B) and serrawettin W1 (C) after 6 days (Scale bar = 500 µm; Blank (A).

Fig. S223. Isolation of endophytic *S. marcescens* MSRBB2 from *M. serrata* stem. Primary isolation plate (A) along with magnification (C) and spread axenic isolate (B).

Fig. S224. Microscopic images of endophytic fungi *Pichia* spp. (A), *A. caesiellus* (B), and *P. virgatula* (C) isolated from *M. serrata*.

II. SUPPLEMENTARY TABLES

Table S1. Peak height ratios from high resolution mass spectrometry of serratamolides labeled with [1,2-$^{13}C_2$]-sodium acetate (n.d. = not detected; * = observed peak height in % of M+4).

III. REFERENCES

I. SUPPLEMENTARY FIGURES

Fig. S1. HRMS[2] of 2-methyl-3-propyl prodiginine (compound **1** $[M+H]^+$: *m/z* 296, $C_{18}H_{22}N_3O$).

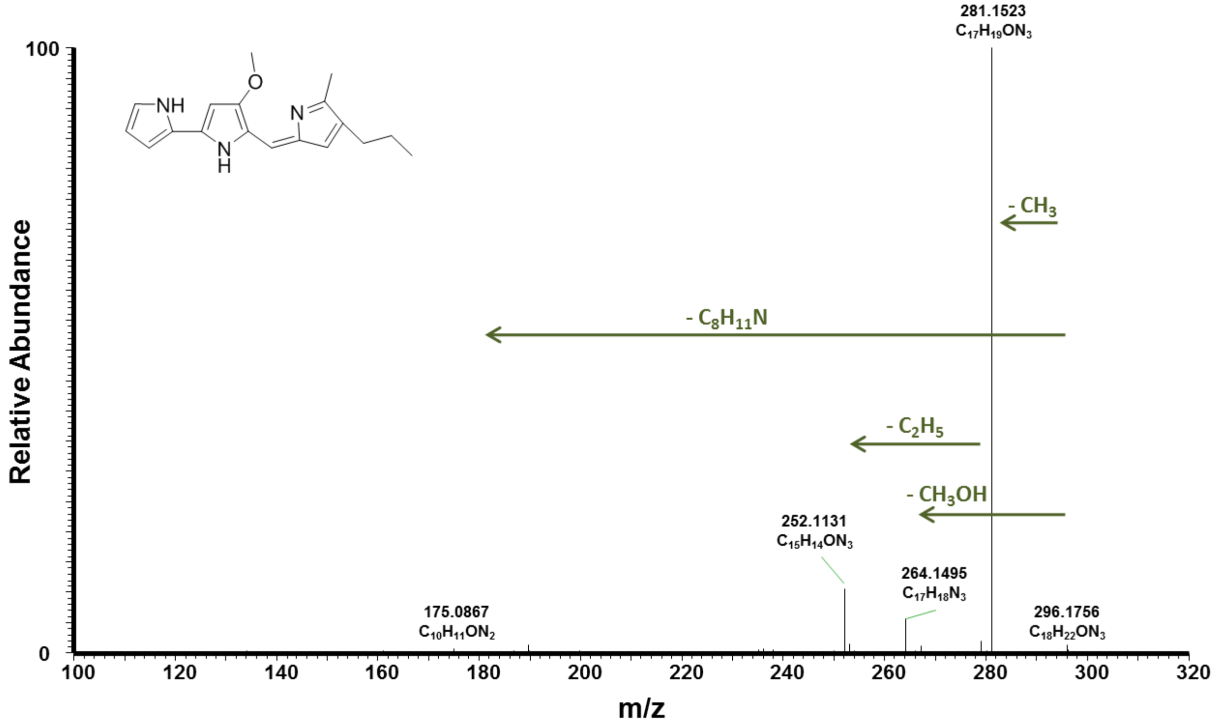

Fig. S2. HRMS[2] of 2-methyl-3-butyl prodiginine (compound **2** $[M+H]^+$: *m/z* 310, $C_{19}H_{24}N_3O$).

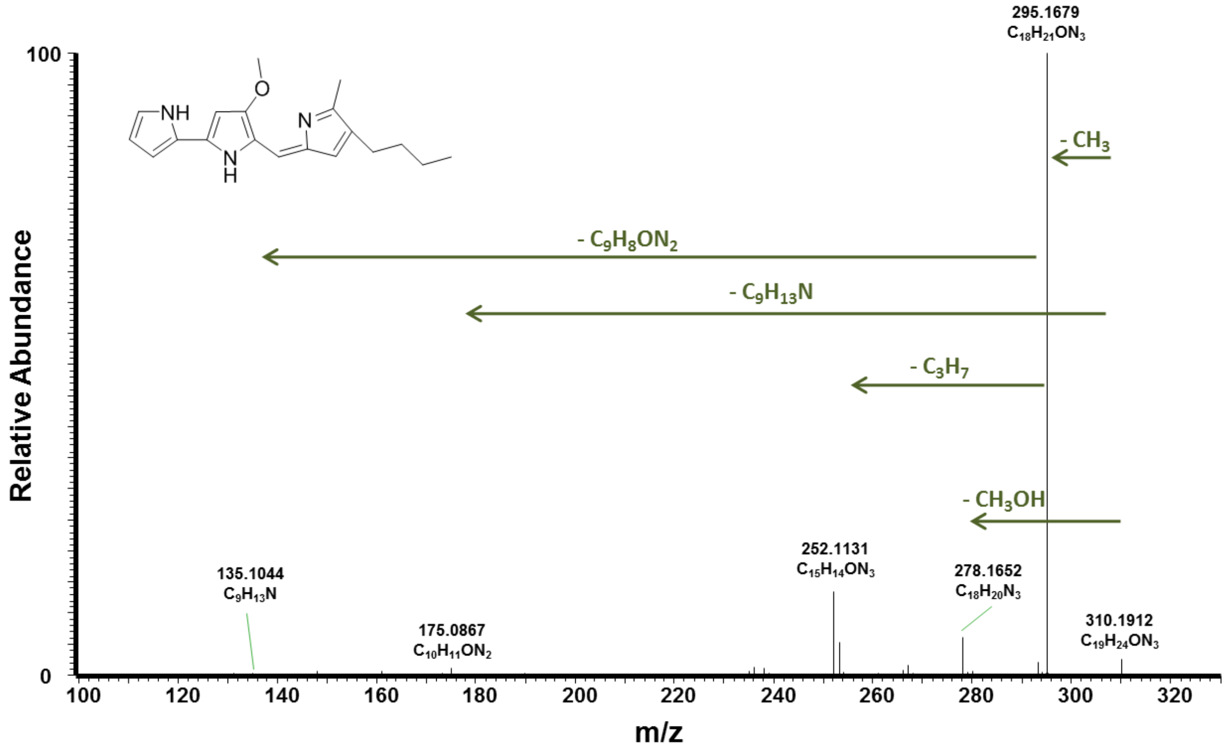

Fig. S3. HRMS[2] of prodigiosin (compound **3** $[M+H]^+$: *m/z* 324, $C_{20}H_{26}N_3O$).

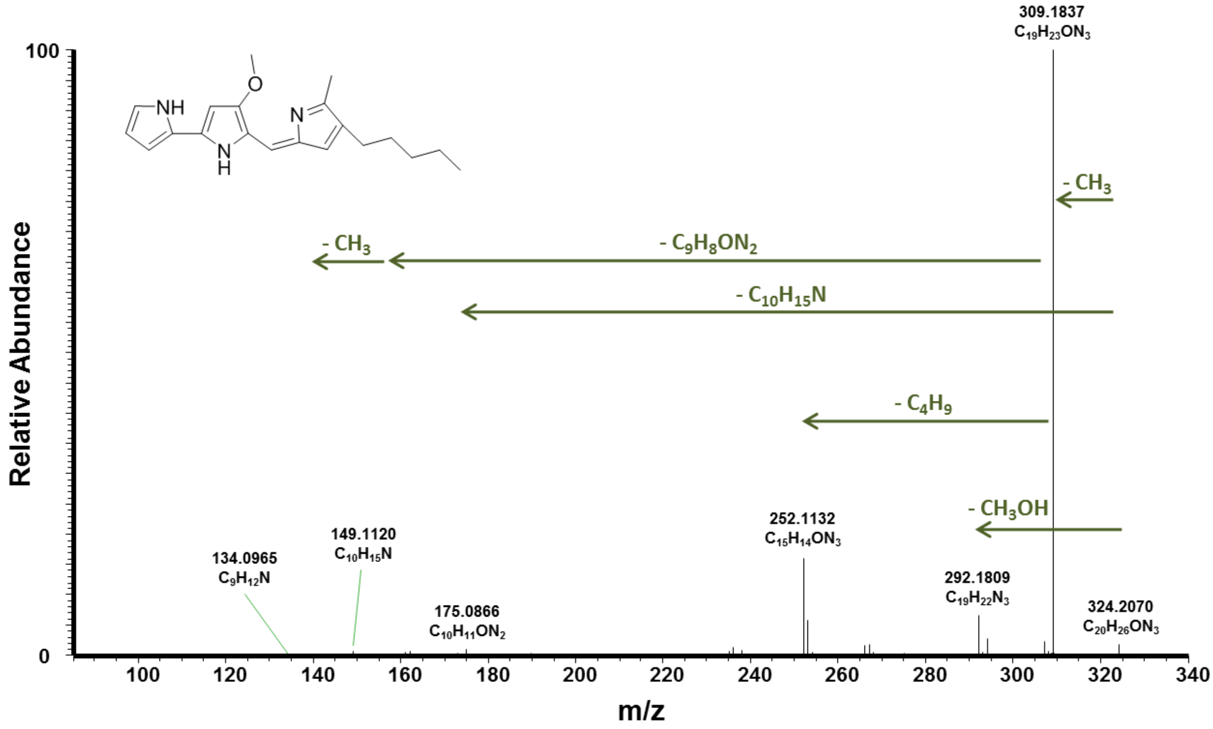

Fig. S4. HRMS[2] of 2-methyl-3-hexyl prodiginine (compound **4** $[M+H]^+$: *m/z* 338, $C_{21}H_{28}N_3O$).

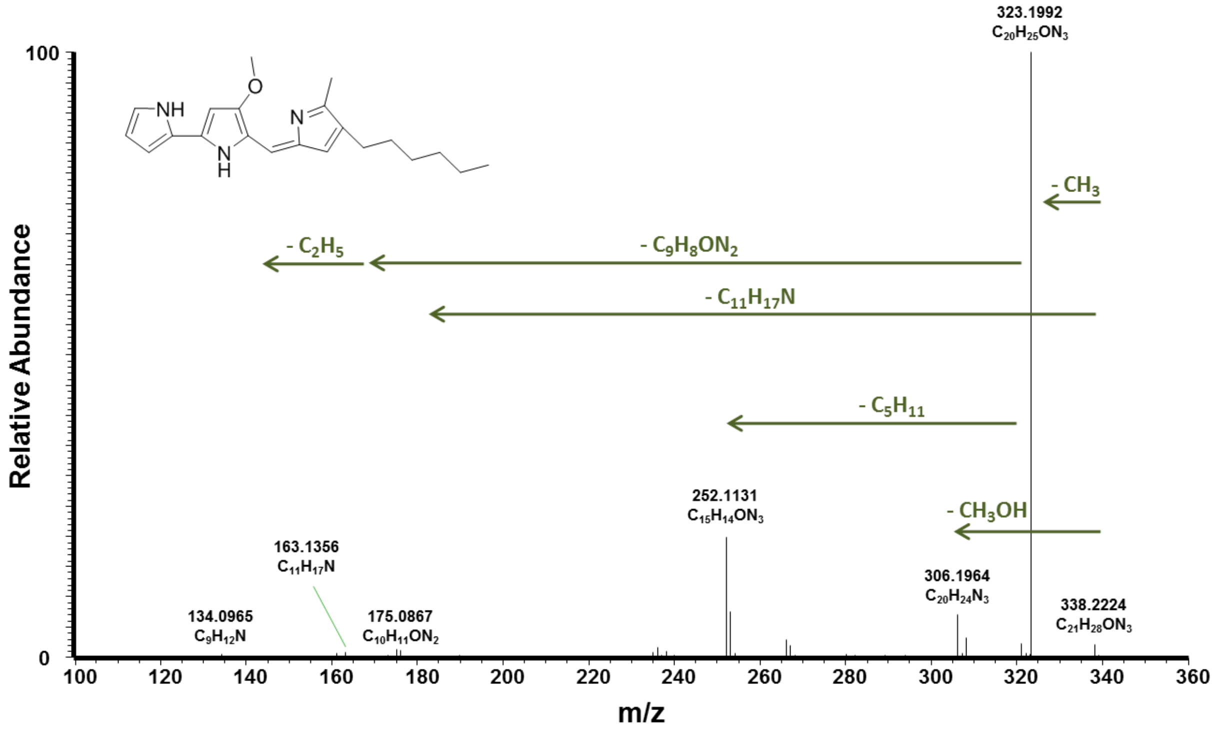

Fig. S5. HRMS^2 of 2-methyl-3-heptyl prodiginine (compound **5** $[M+H]^+$: *m/z* 352, $C_{22}H_{30}N_3O$).

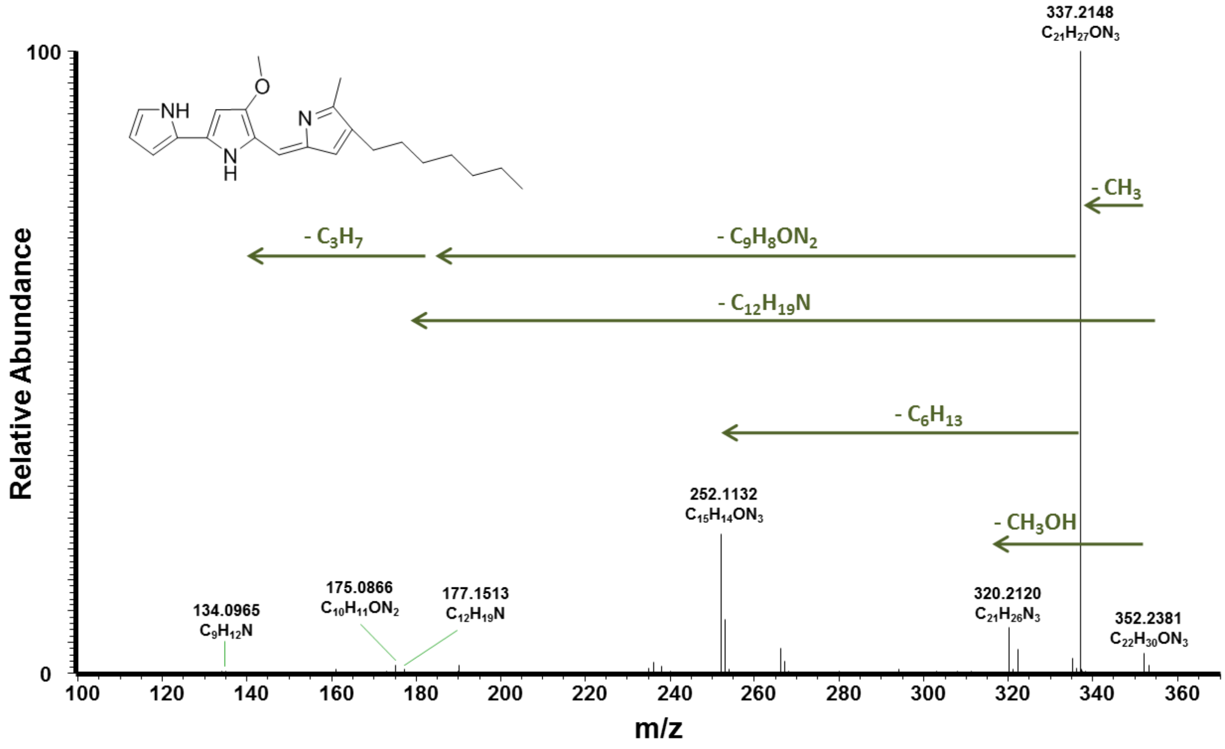

Fig. S6. HRMS^2 of 2-methyl-3-octyl prodiginine (compound **6** $[M+H]^+$: *m/z* 366, $C_{23}H_{32}N_3O$).

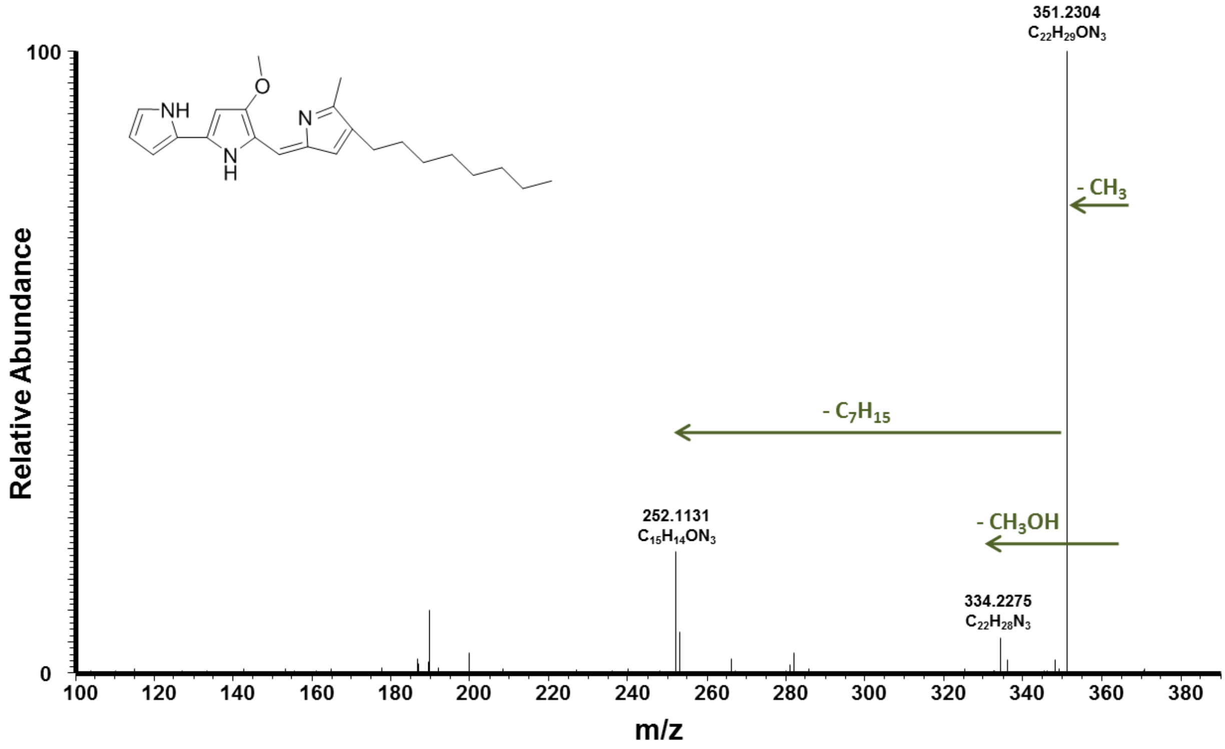

Fig. S7. HRMS[2] of 2-methyl-3-nonyl prodiginine (compound **7** $[M+H]^+$: *m/z* 380, $C_{24}H_{34}N_3O$).

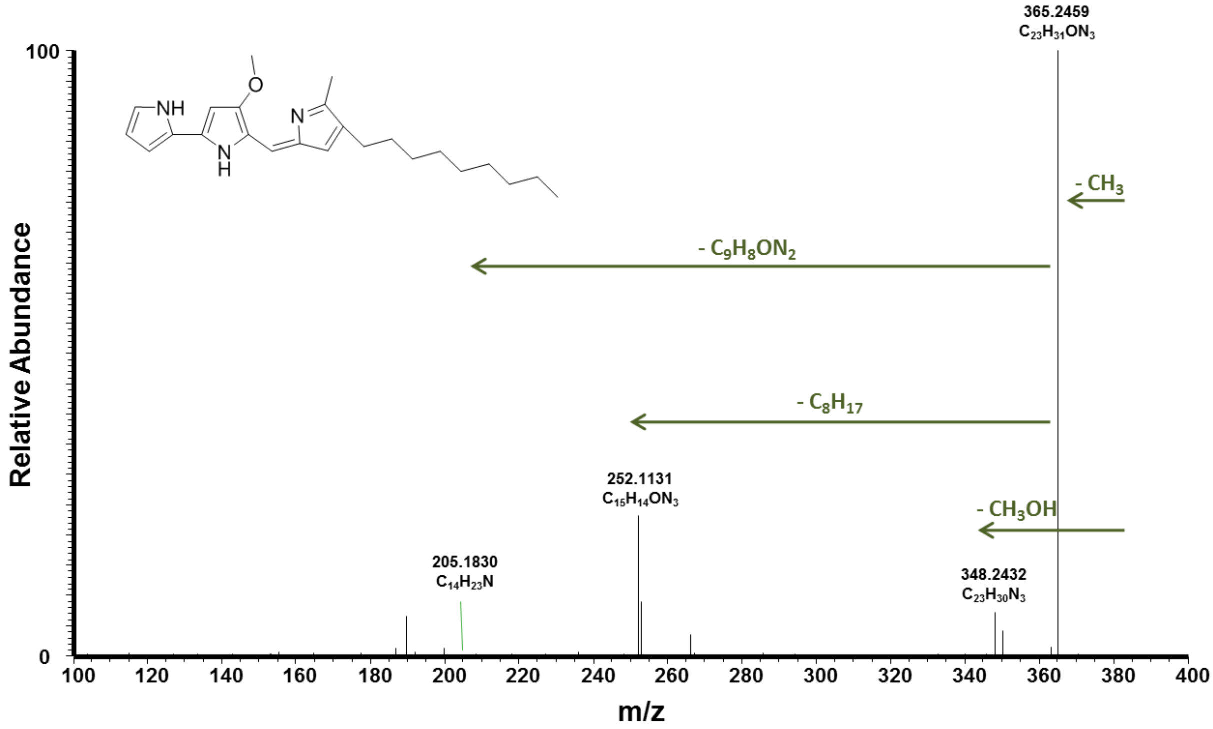

Fig. S8. HRMS[3] of 2-methyl-3-propyl prodiginine (compound **1** $[M+H]^+$: *m/z* 296 via 281).

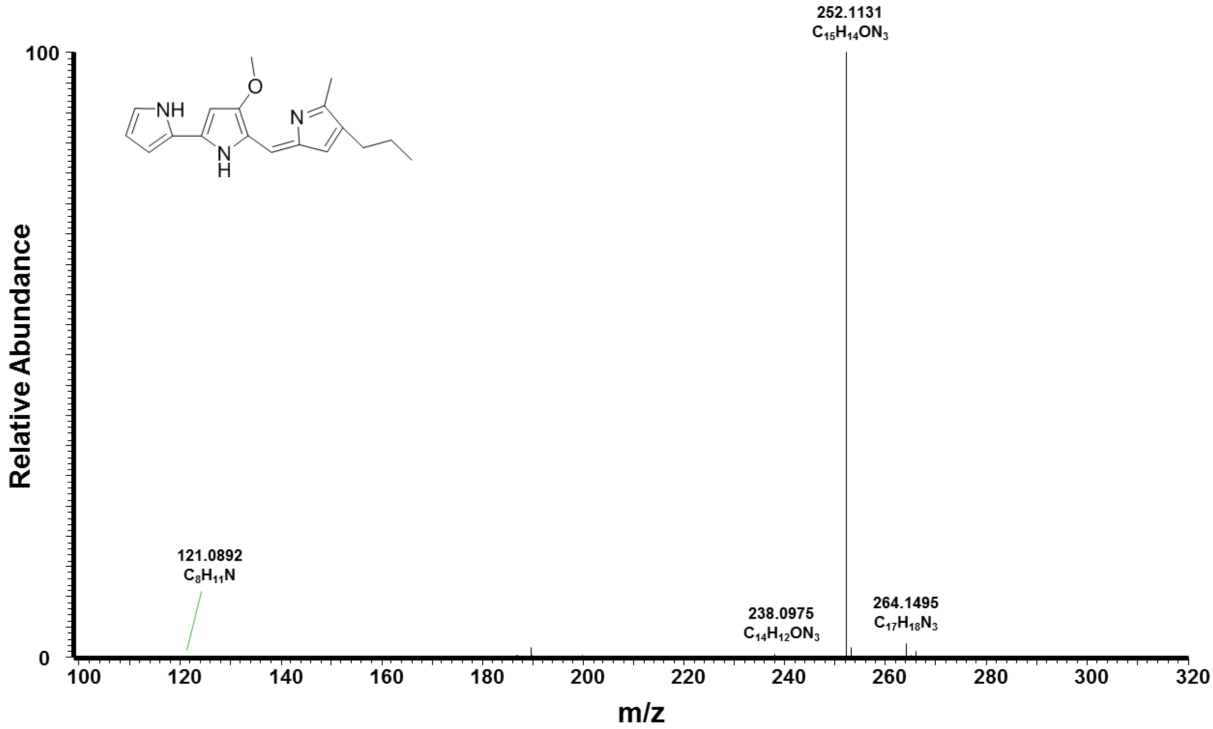

Fig. S9. HRMS^3 of 2-methyl-3-butyl prodiginine (compound **2** $[M+H]^+$: *m/z* 310 via 295).

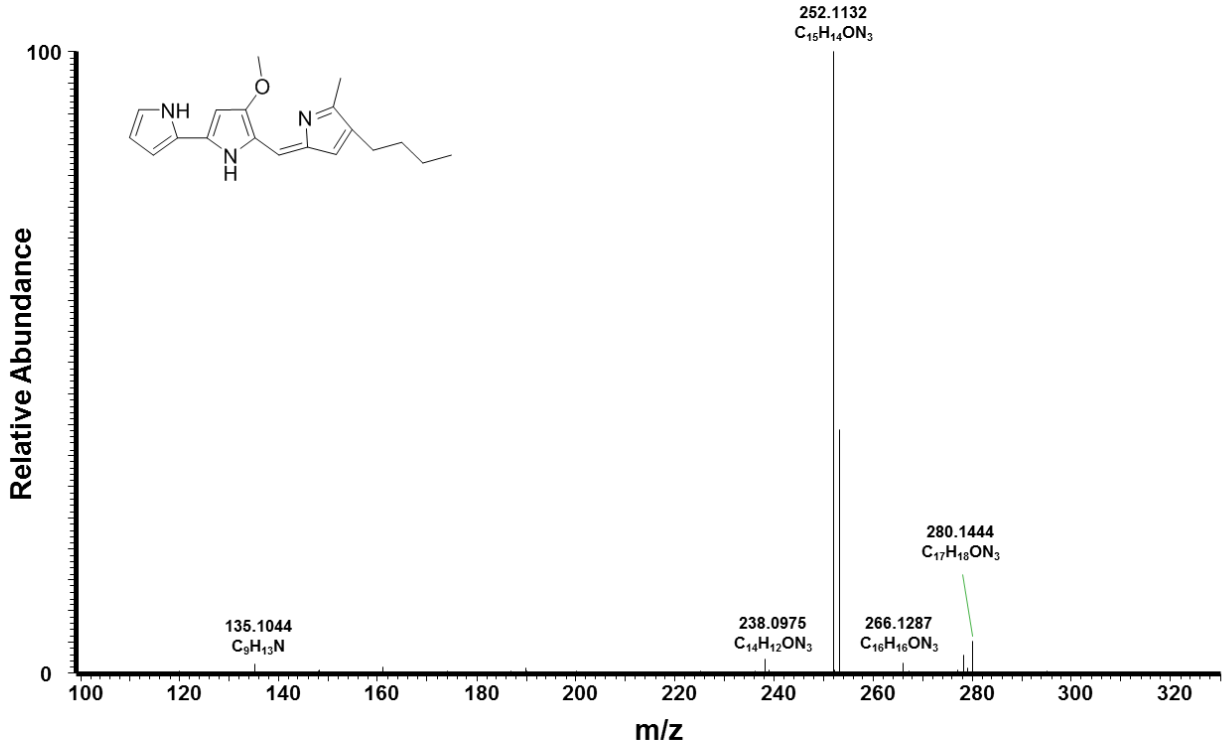

Fig. S10. HRMS^3 of prodigiosin (compound **3** $[M+H]^+$: *m/z* 324 via 309).

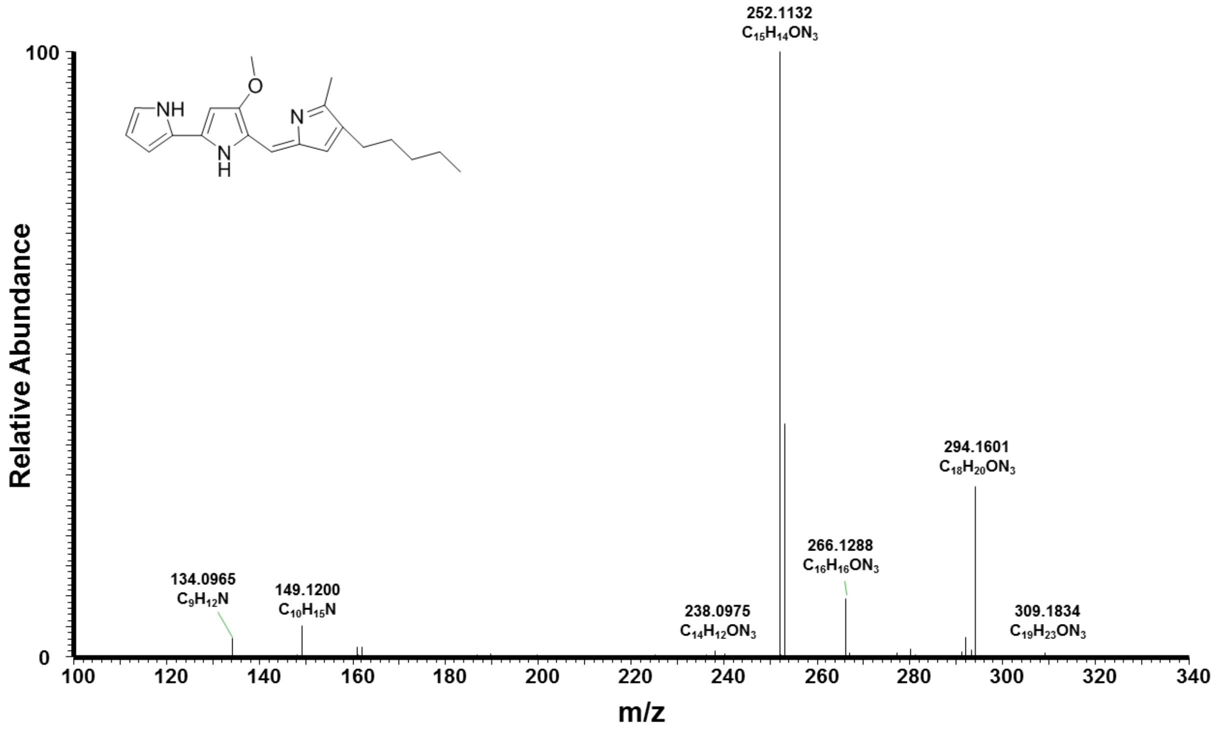

Fig. S11. HRMS^3 of 2-methyl-3-hexyl prodiginine (compound **4** $[M+H]^+$: *m/z* 338 via 323).

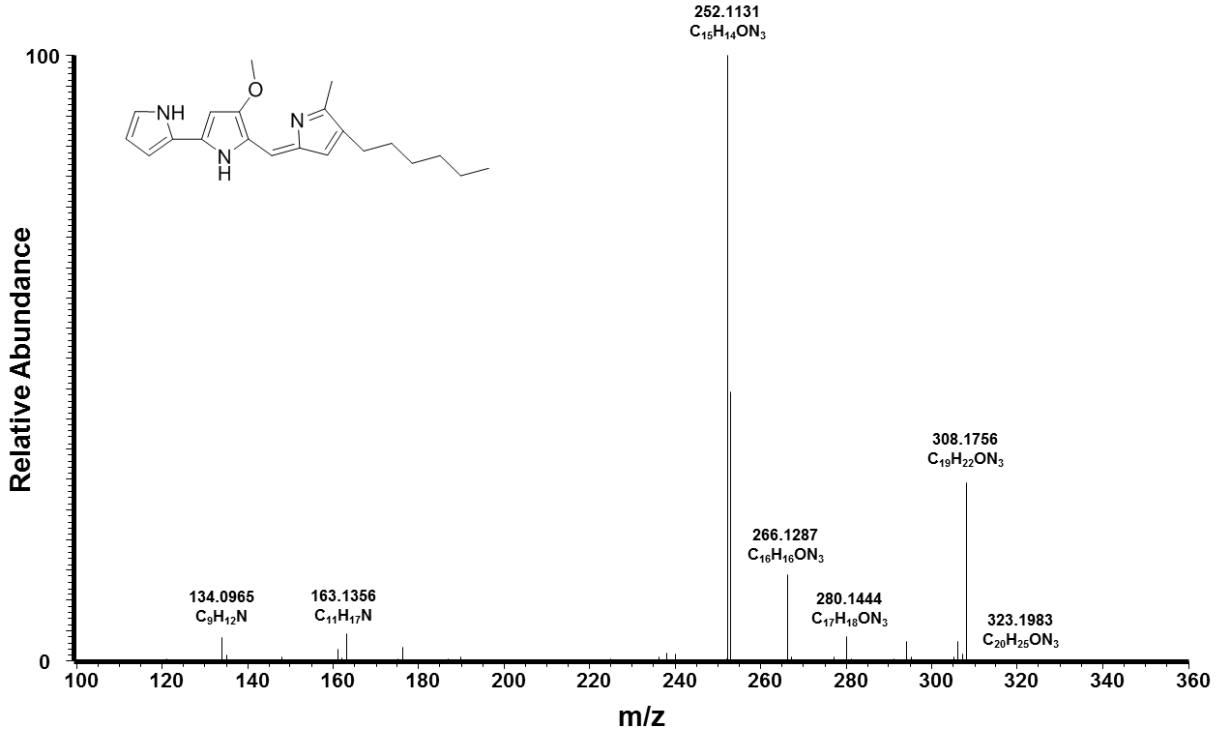

Fig. S12. HRMS^3 of 2-methyl-3-heptyl prodiginine (compound **5** $[M+H]^+$: *m/z* 352 via 337).

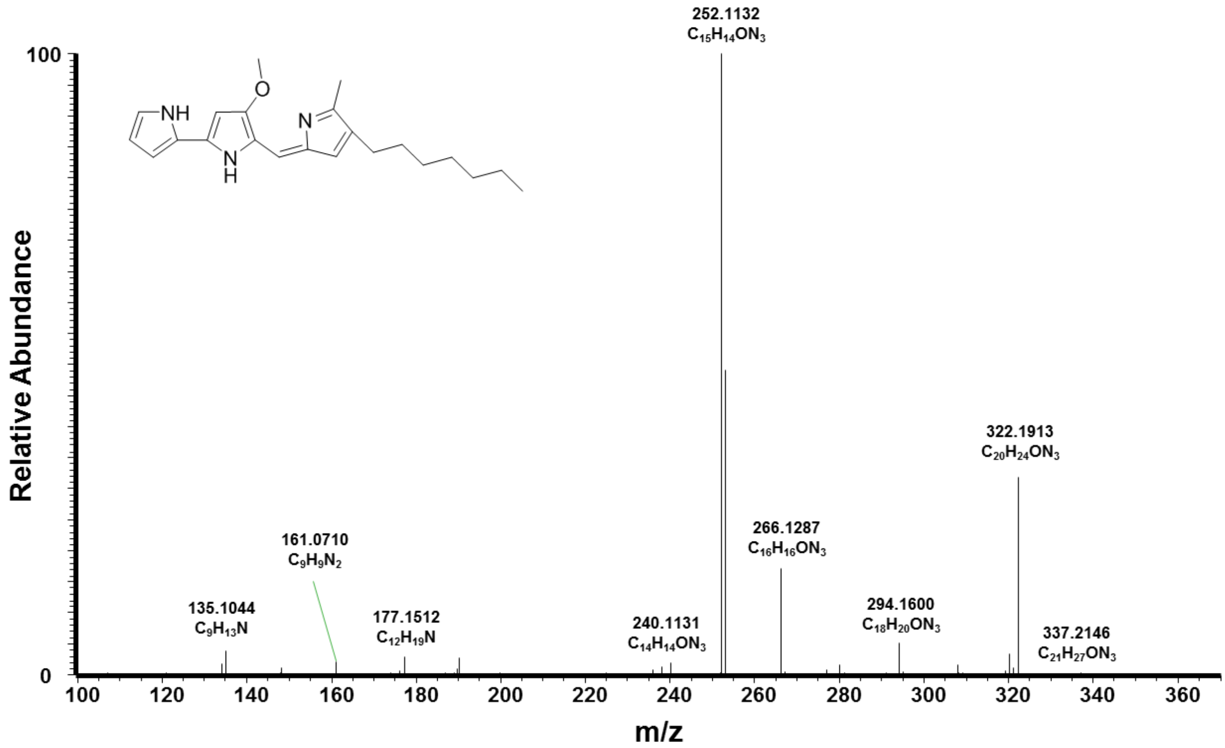

Fig. S13. HRMS[3] of 2-methyl-3-octyl prodiginine (compound **6** $[M+H]^+$: *m/z* 366 via 351).

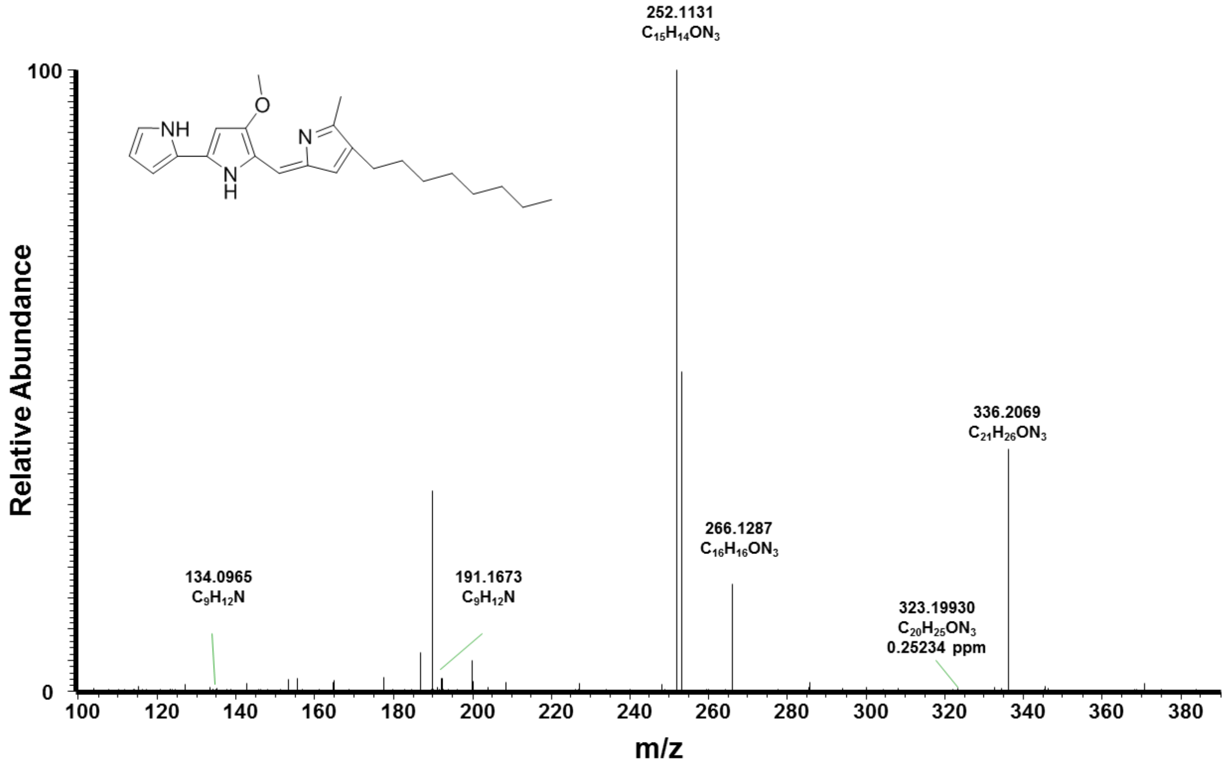

Fig. S14. HRMS[3] of 2-methyl-3-nonyl prodiginine (compound **7** $[M+H]^+$: *m/z* 380 via 365).

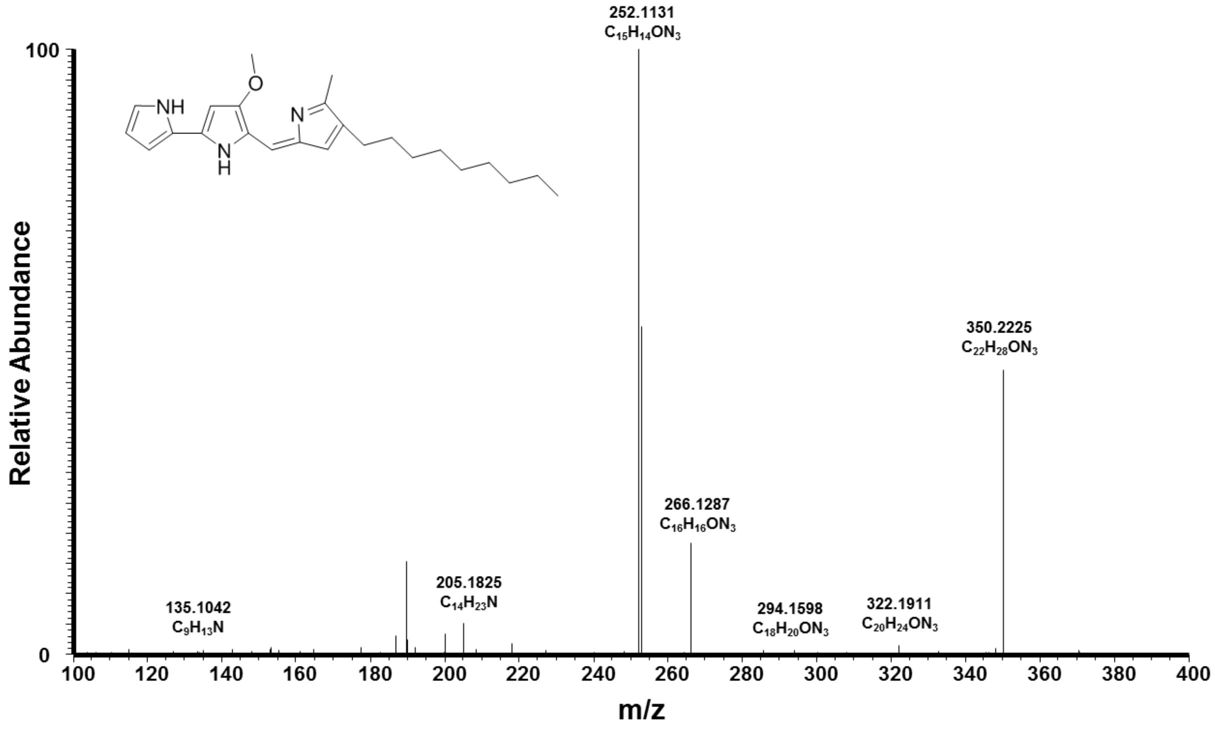

Fig. S15. Proposed mass spectral fragmentation pathway of 2-methyl-3-propyl prodiginine (compound **1** $[M+H]^+$: *m/z* 296, $C_{18}H_{22}N_3O$).

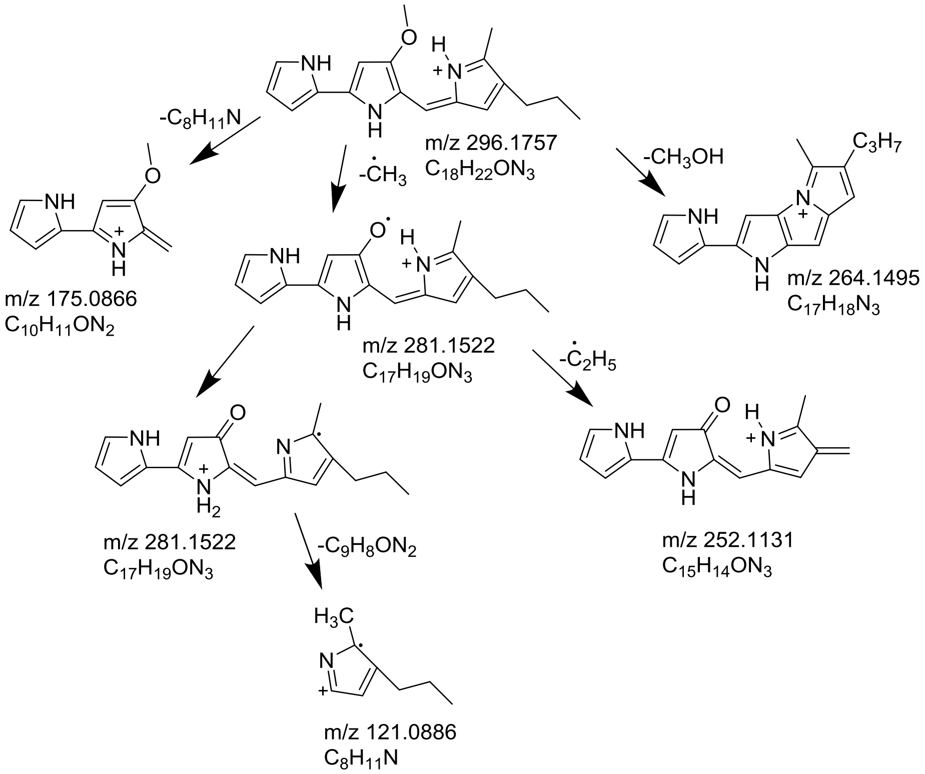

Fig. S16. Proposed mass spectral fragmentation pathway of 2-methyl-3-butyl prodiginine (compound **2** $[M+H]^+$: *m/z* 310, $C_{19}H_{24}N_3O$).

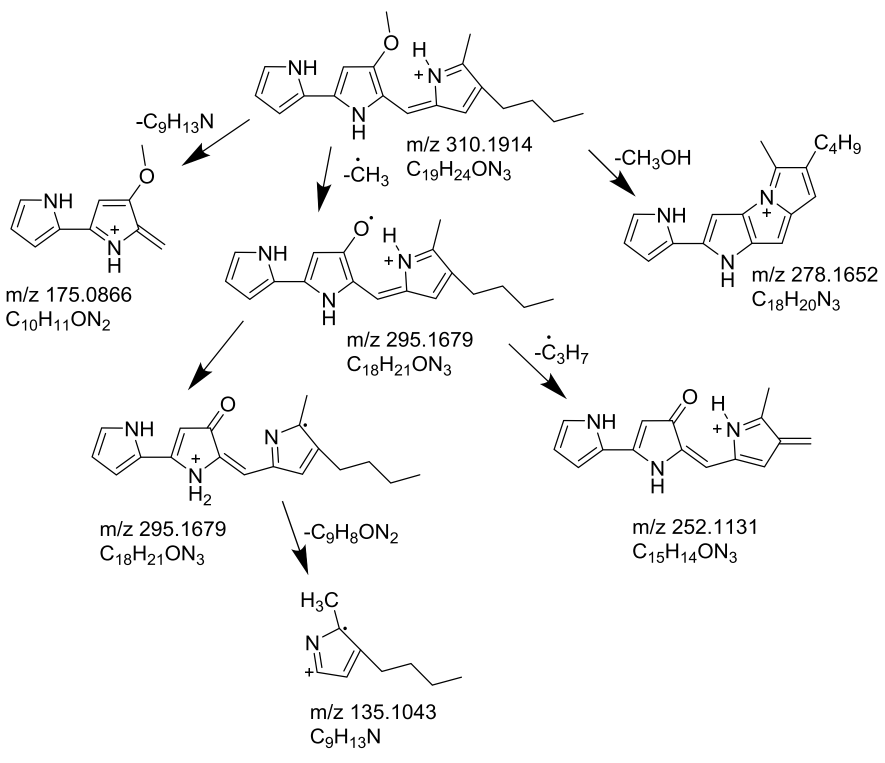

Fig. S17. Proposed mass spectral fragmentation pathway of prodigiosin (compound **3** $[M+H]^+$: *m/z* 324, $C_{20}H_{26}N_3O$).

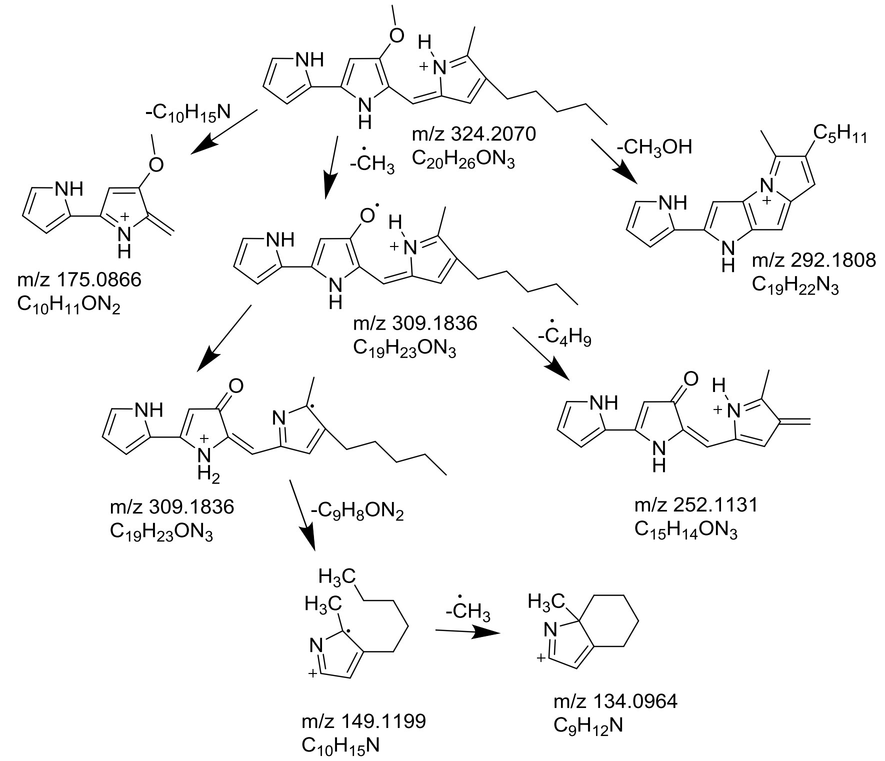

Fig. S18. Proposed mass spectral fragmentation pathway of 2-methyl-3-hexyl prodiginine (compound **4** $[M+H]^+$: *m/z* 338, $C_{21}H_{28}N_3O$).

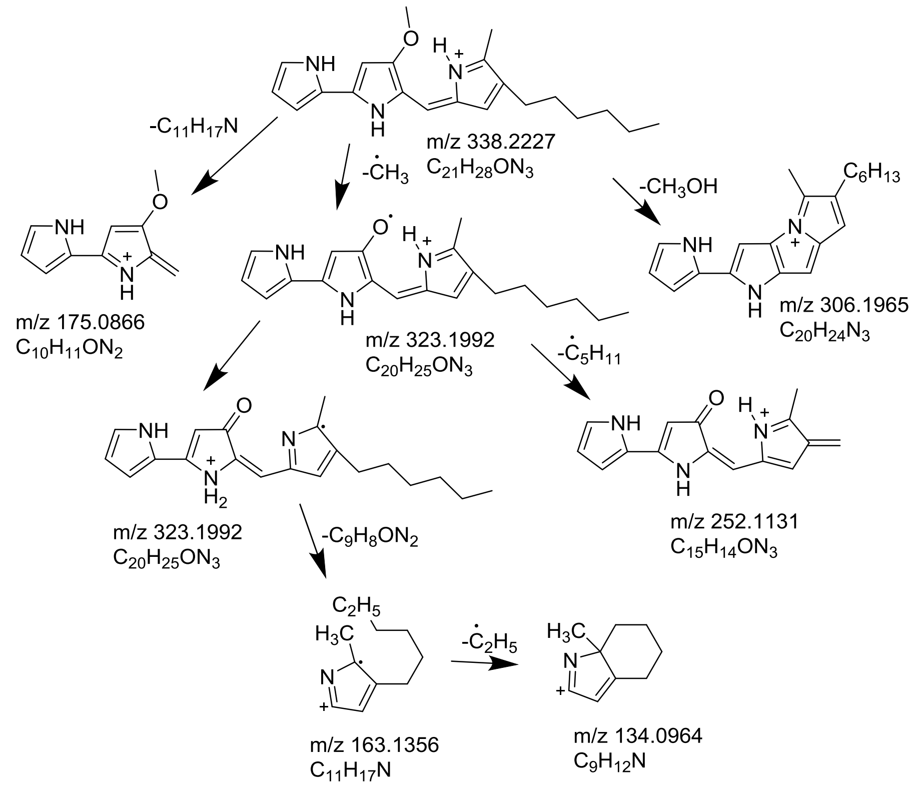

Fig. S19. Proposed mass spectral fragmentation pathway of 2-methyl-3-heptyl prodiginine (compound **5** $[M+H]^+$: *m/z* 352, $C_{22}H_{30}N_3O$).

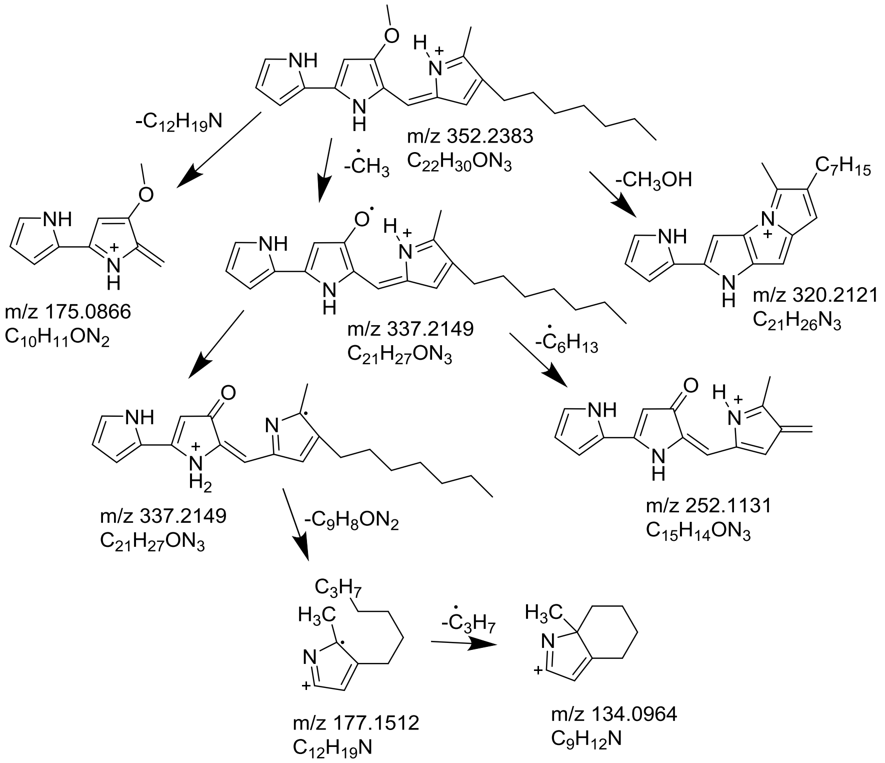

Fig. S20. Proposed mass spectral fragmentation pathway of 2-methyl-3-octyl prodiginine (compound **6** $[M+H]^+$: *m/z* 366, $C_{23}H_{32}N_3O$).

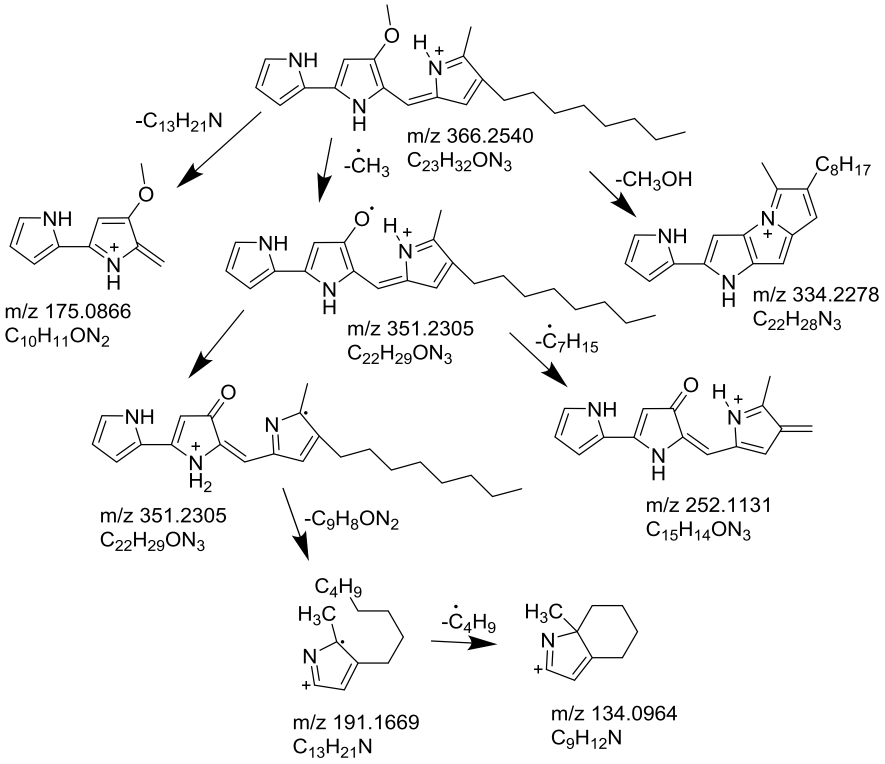

Fig. S21. Proposed mass spectral fragmentation pathway of 2-methyl-3-nonyl prodiginine (compound **7** $[M+H]^+$: *m/z* 380, $C_{24}H_{34}N_3O$).

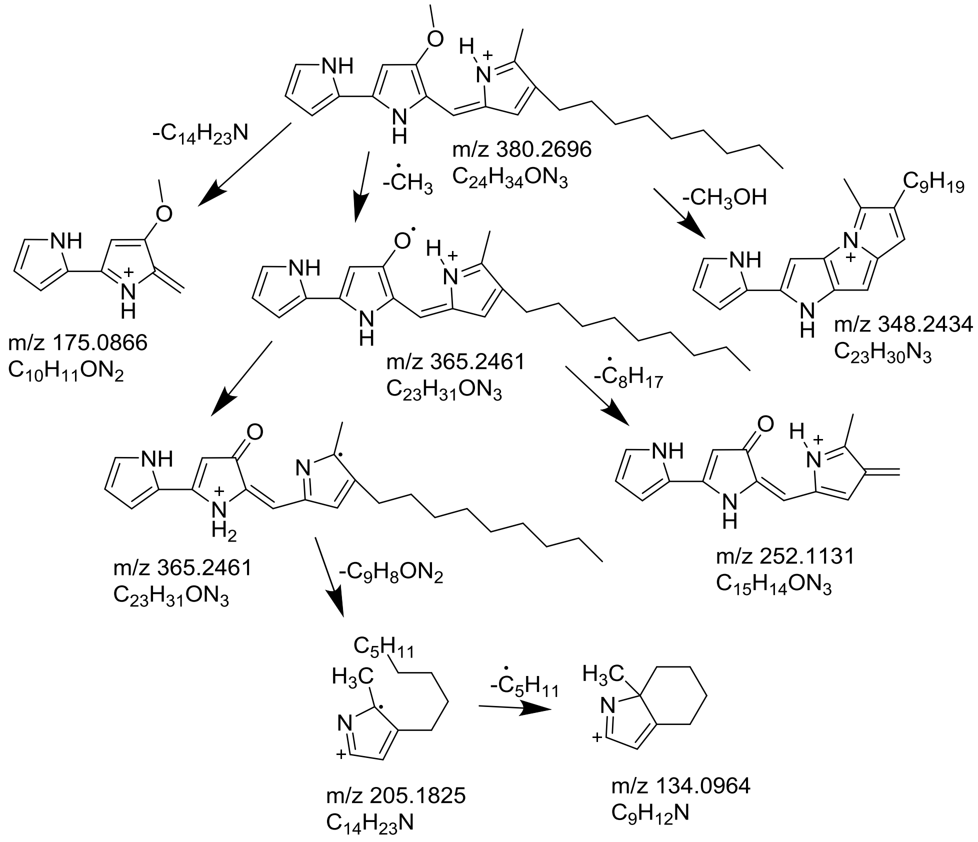

Fig. S22. HPLC-HRMS analysis of prodiginines (**1**-**7**). TIC, extracted ion chromatograms of prodiginines, and area in comparison to the main metabolite prodigiosin.

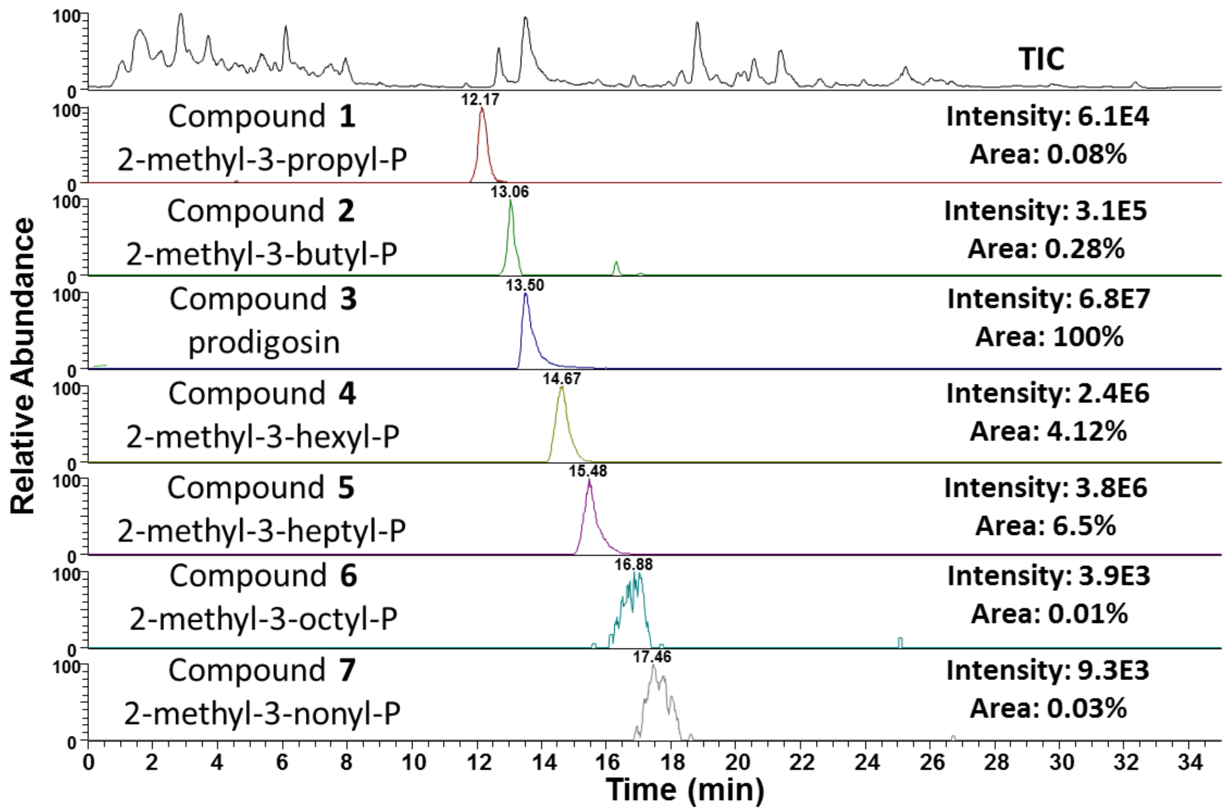

Fig. S23. HPLC-HRMS analysis of serratamolides (**8-14**). TIC, extracted ion chromatograms of serratamolides, and area in comparison to the main metabolite serrawettin W1.

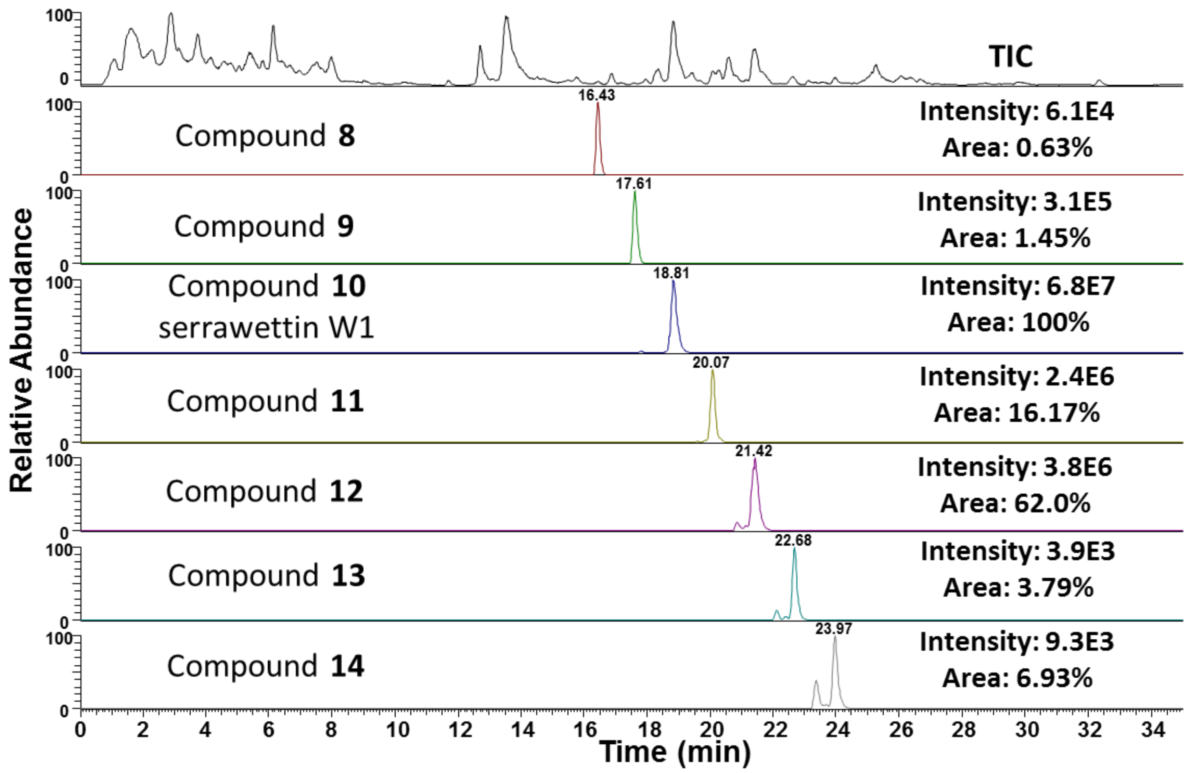

Fig. S24. HPLC-HRMS analysis of serratamolides (**15-19**). TIC, extracted ion chromatograms of serratamolides, and area in comparison to the main metabolite serrawettin W1*.

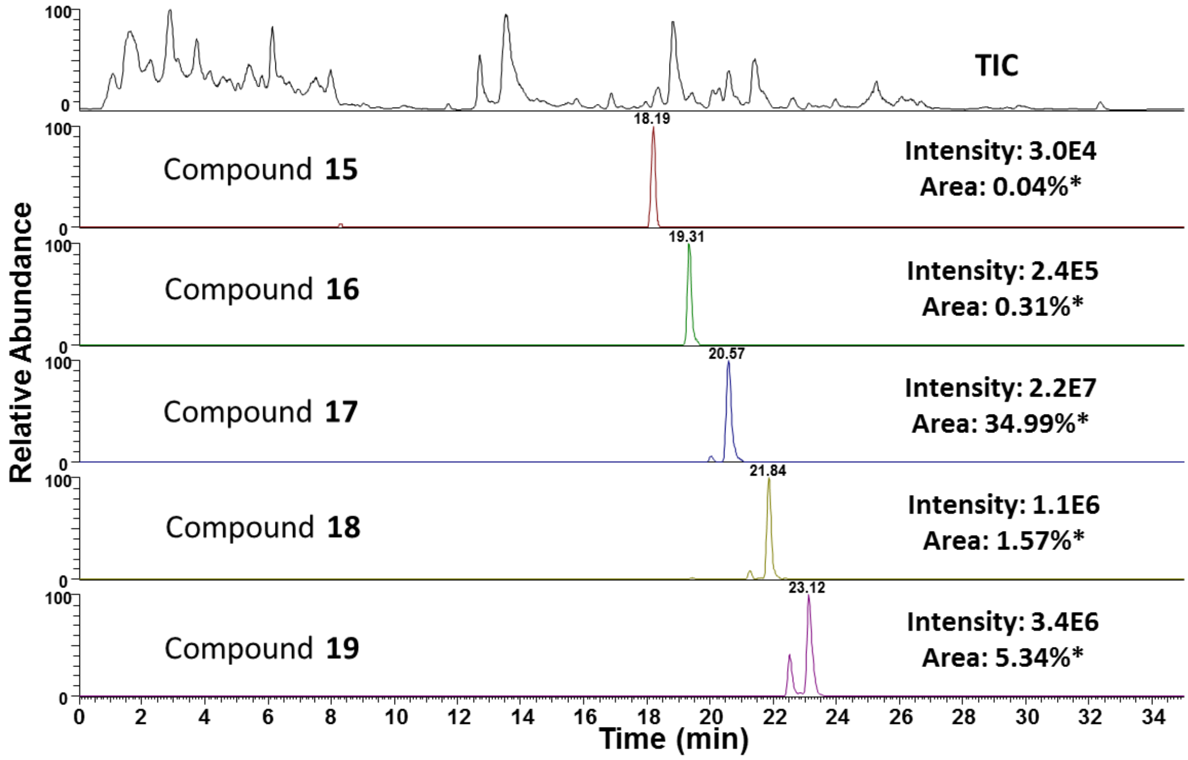

Fig. S25. HPLC-HRMS analysis of serratamolides (**20-25**). TIC, extracted ion chromatograms of serratamolides, and area in comparison to the main metabolite serrawettin W1*.

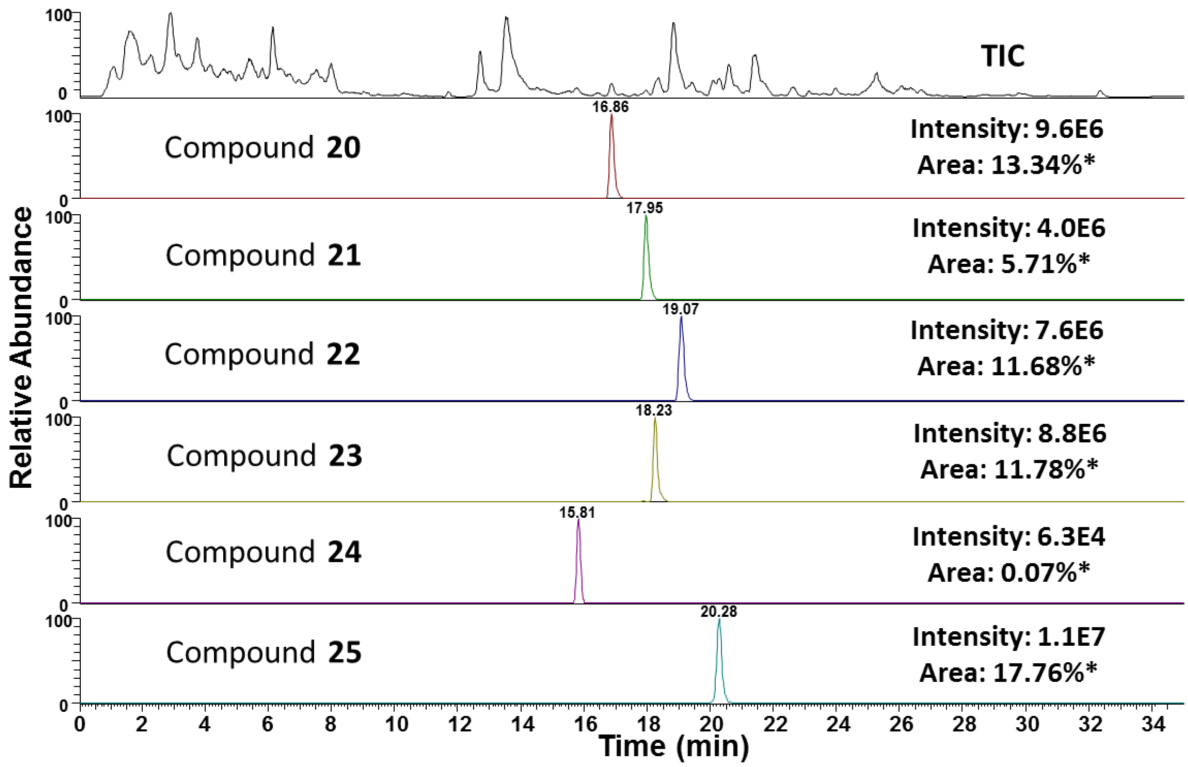

Fig. S26. HPLC-HRMS analysis of serratamolides (**26-30**). TIC, extracted ion chromatograms of serratamolides, and area in comparison to the main metabolite serrawettin W1*.

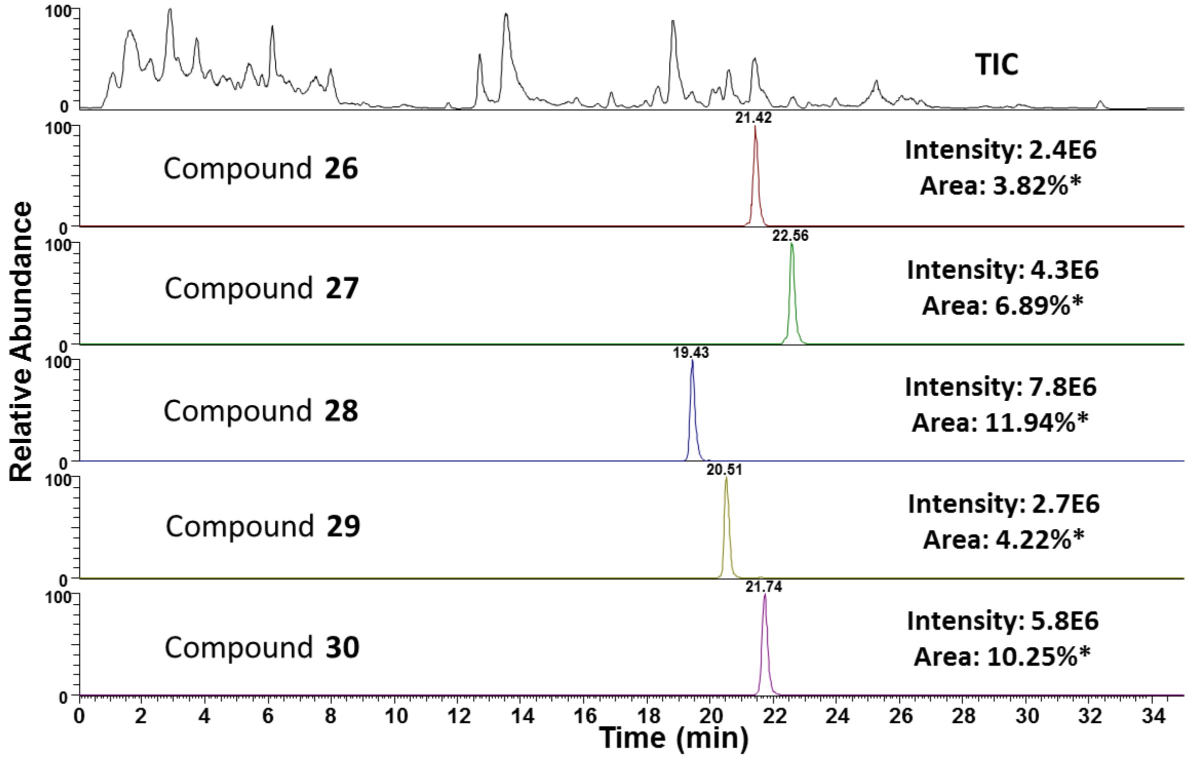

Fig. S27. HPLC-HRMS analysis of serratamolides (**31-33**). TIC, extracted ion chromatograms of serratamolides, and area in comparison to the main metabolite serrawettin W1*.

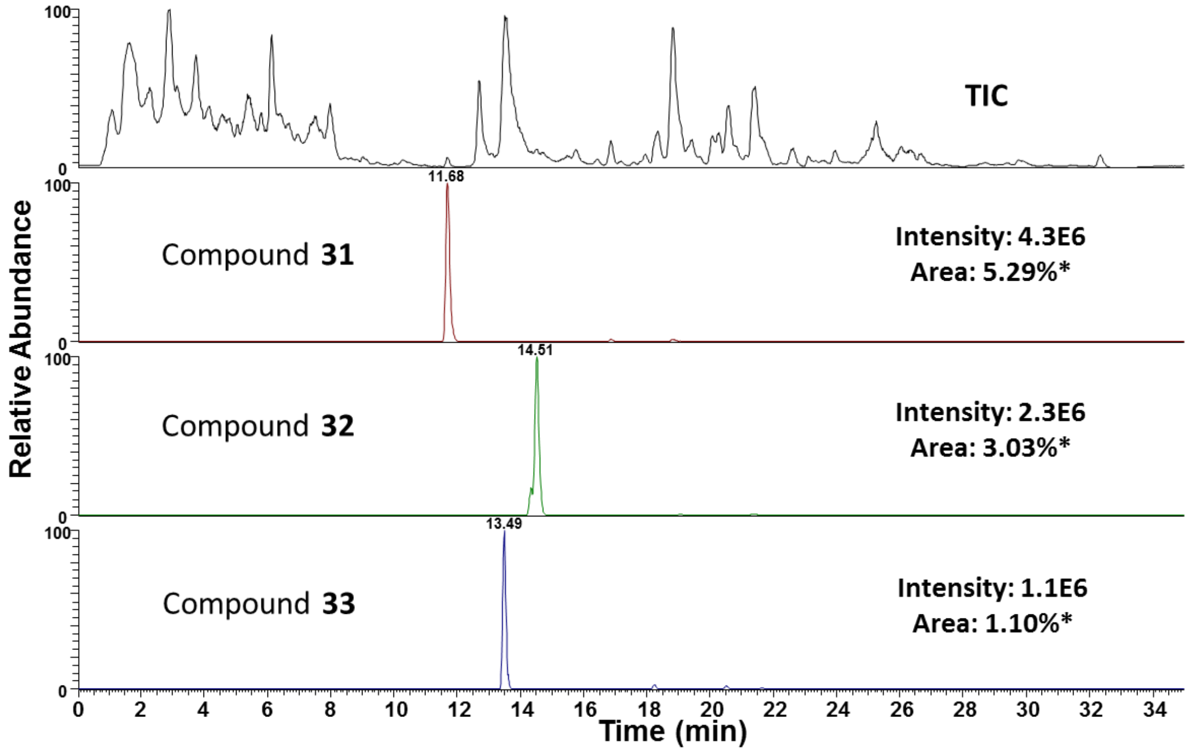

Fig. S28. HRMS^2 of serratamolide C_8+C_{10} (compound **8** $[M+H]^+$: *m/z* 487, $C_{24}H_{43}N_2O_8$).

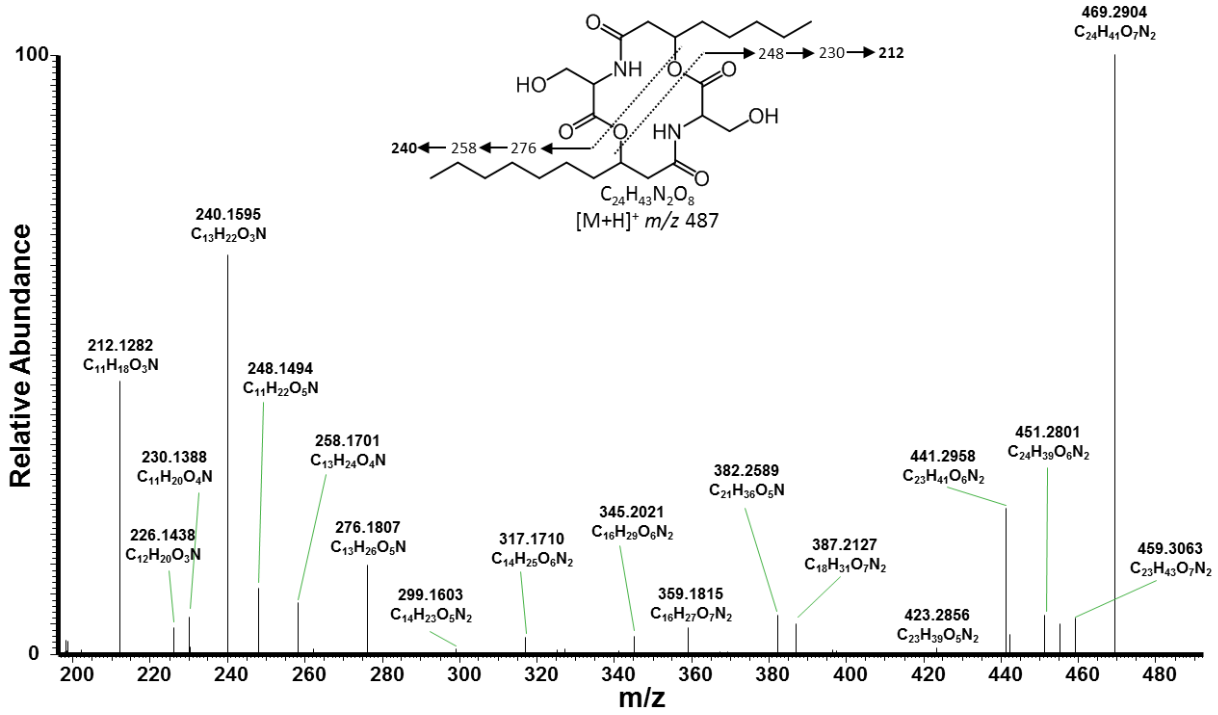

Fig. S29. Proposed mass spectral fragmentation pathway of serratamolide C_8+C_{10} (compound **8** $[M+H]^+$: *m/z* 487, $C_{24}H_{43}N_2O_8$).

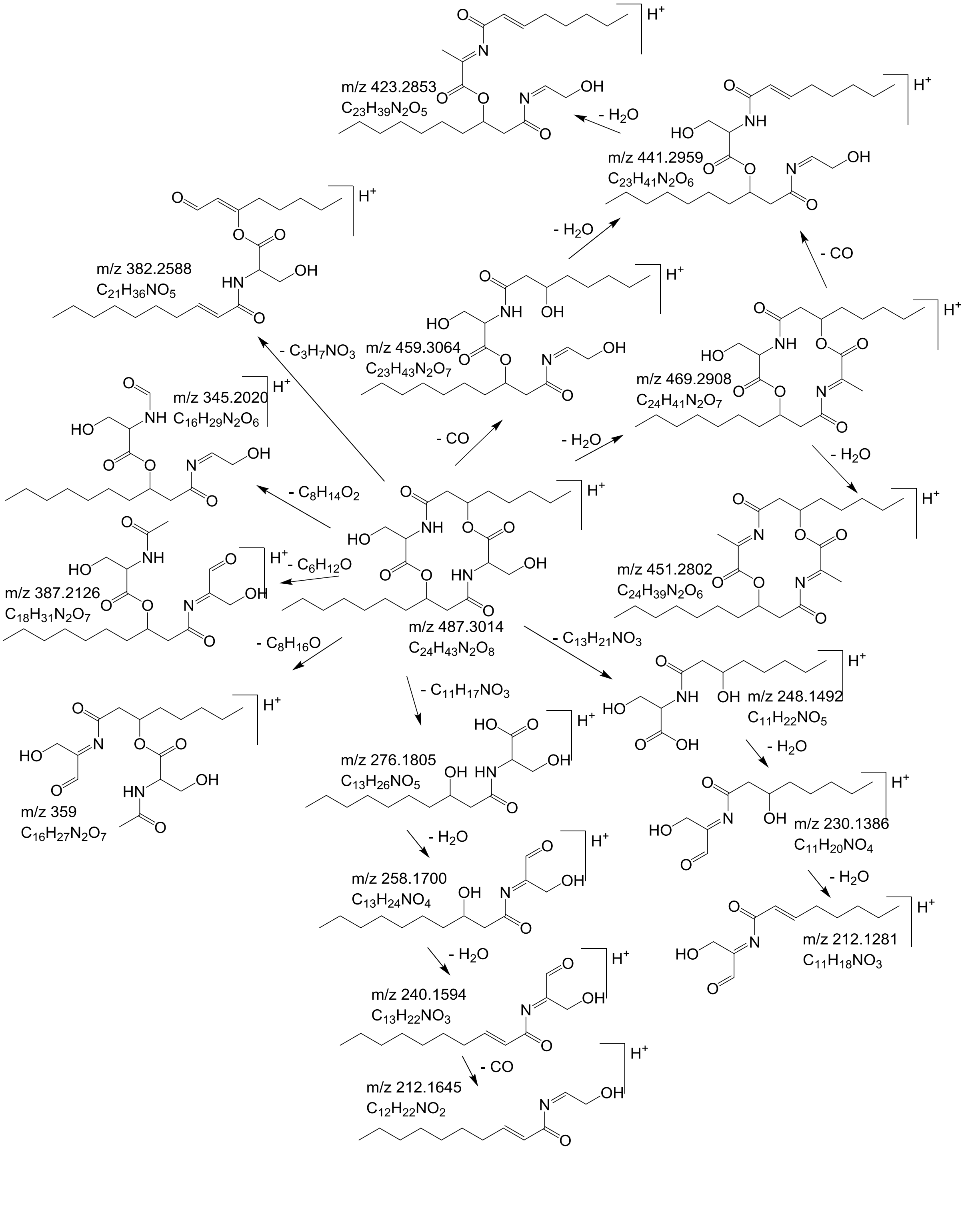

Fig. S30. HRMS[2] of serratamolide C_9+C_{10} (compound **9** $[M+H]^+$: *m/z* 501, $C_{25}H_{45}N_2O_8$).

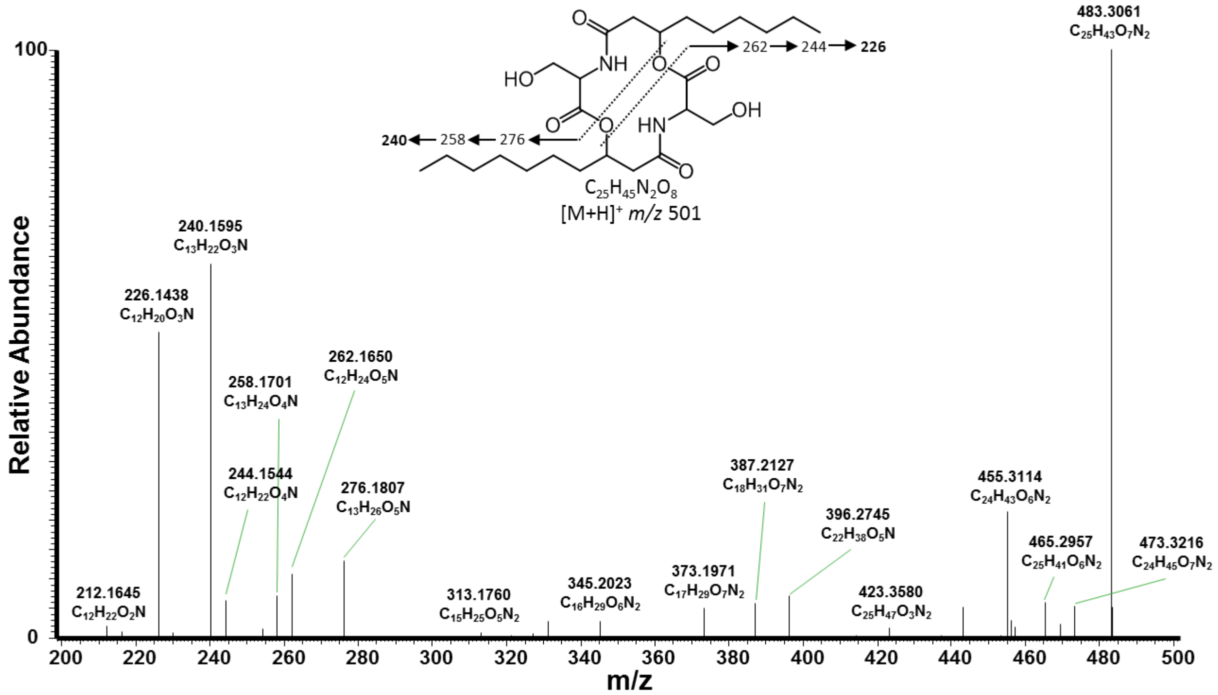

Fig. S31. Proposed mass spectral fragmentation pathway of serratamolide C_9+C_{10} (compound **9** $[M+H]^+$: *m/z* 501, $C_{25}H_{45}N_2O_8$).

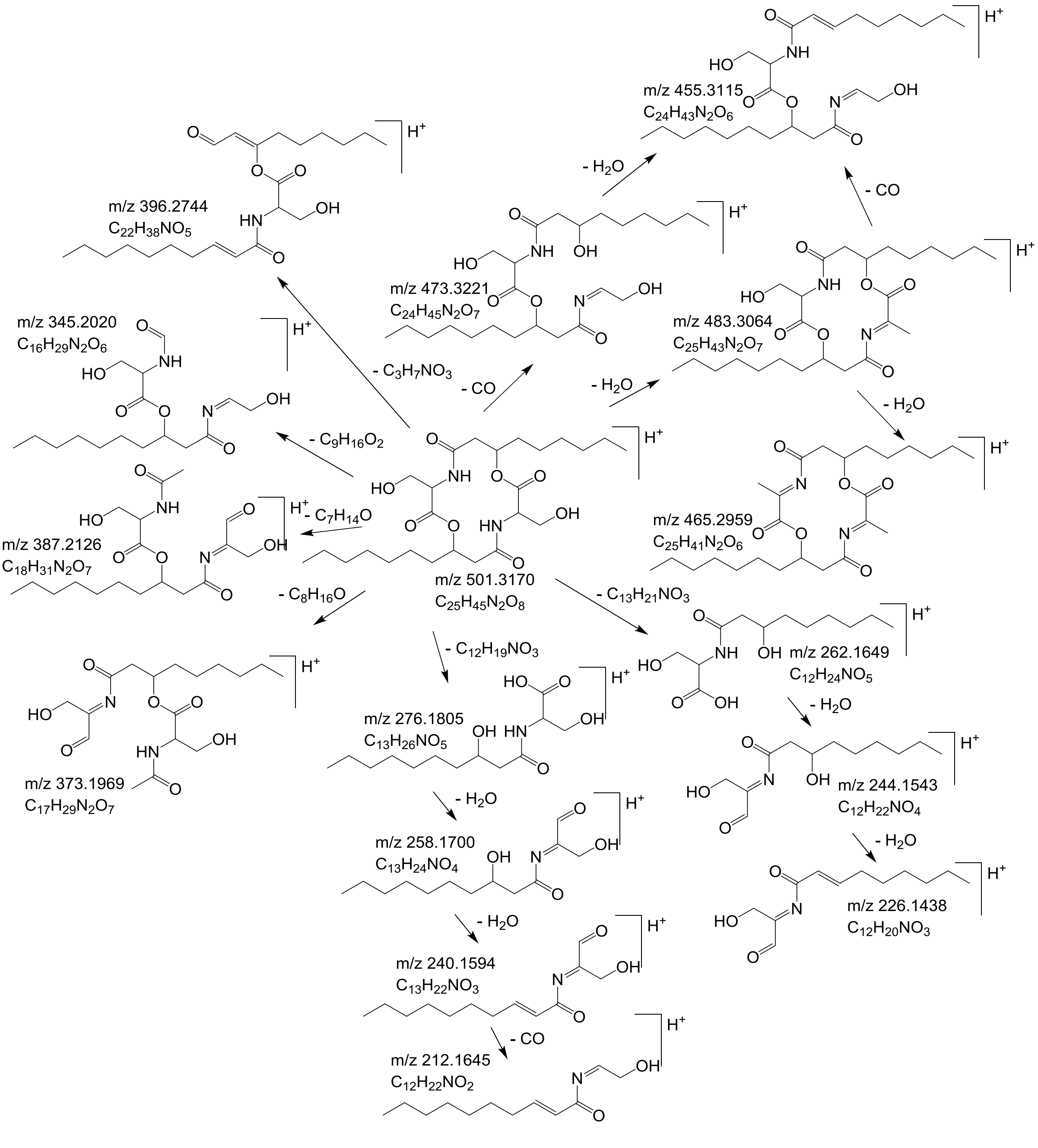

Fig. S32. HRMS[2] of serrawettin W1 / serratamolide C_{10}+C_{10} (compound **10** $[M+H]^+$: *m/z* 515, $C_{26}H_{47}N_2O_8$).

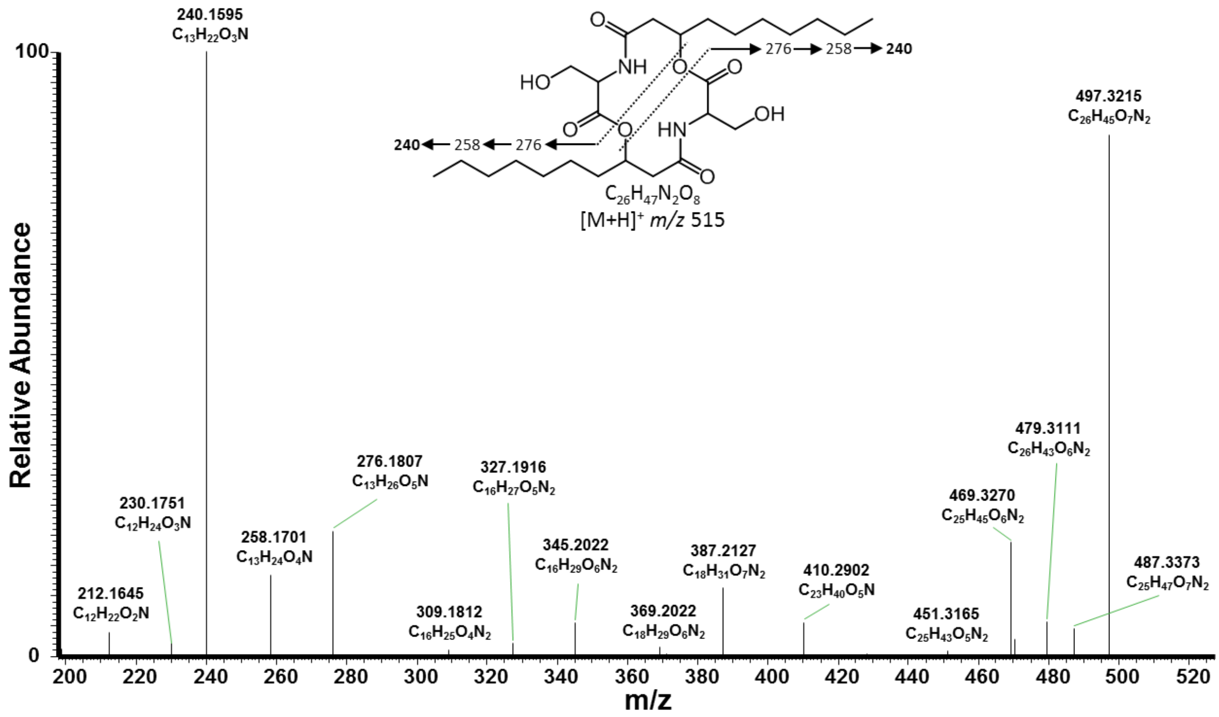

Fig. S33. Proposed mass spectral fragmentation pathway of serrawettin W1 / serratamolide C_{10}+C_{10} (compound **10** $[M+H]^+$: *m/z* 515, $C_{26}H_{47}N_2O_8$).

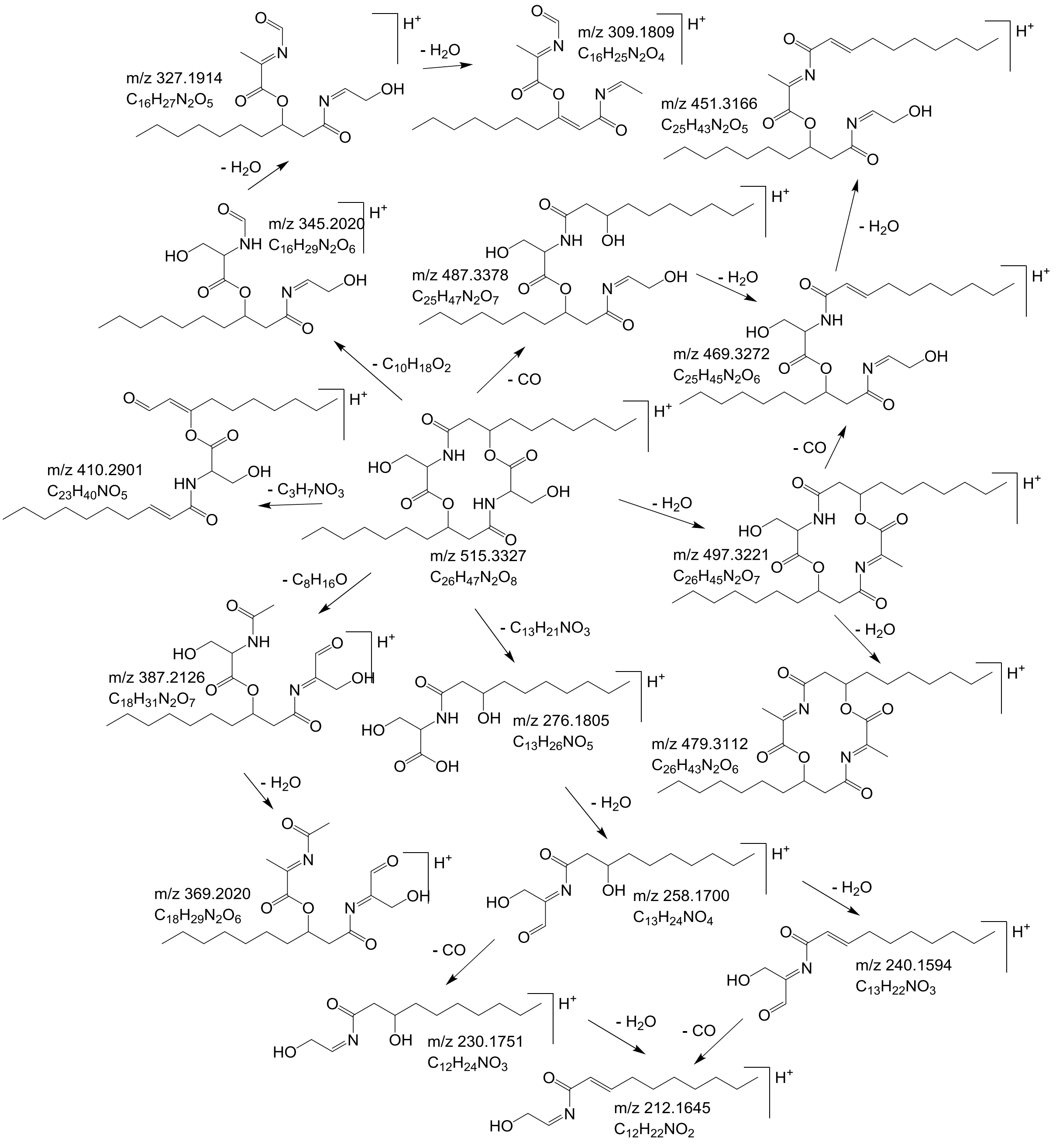

Fig. S34. HRMS^2 of serratamolide C_{10}+C_{11} (compound **11** $[M+H]^+$: *m/z* 529, $C_{27}H_{49}N_2O_8$).

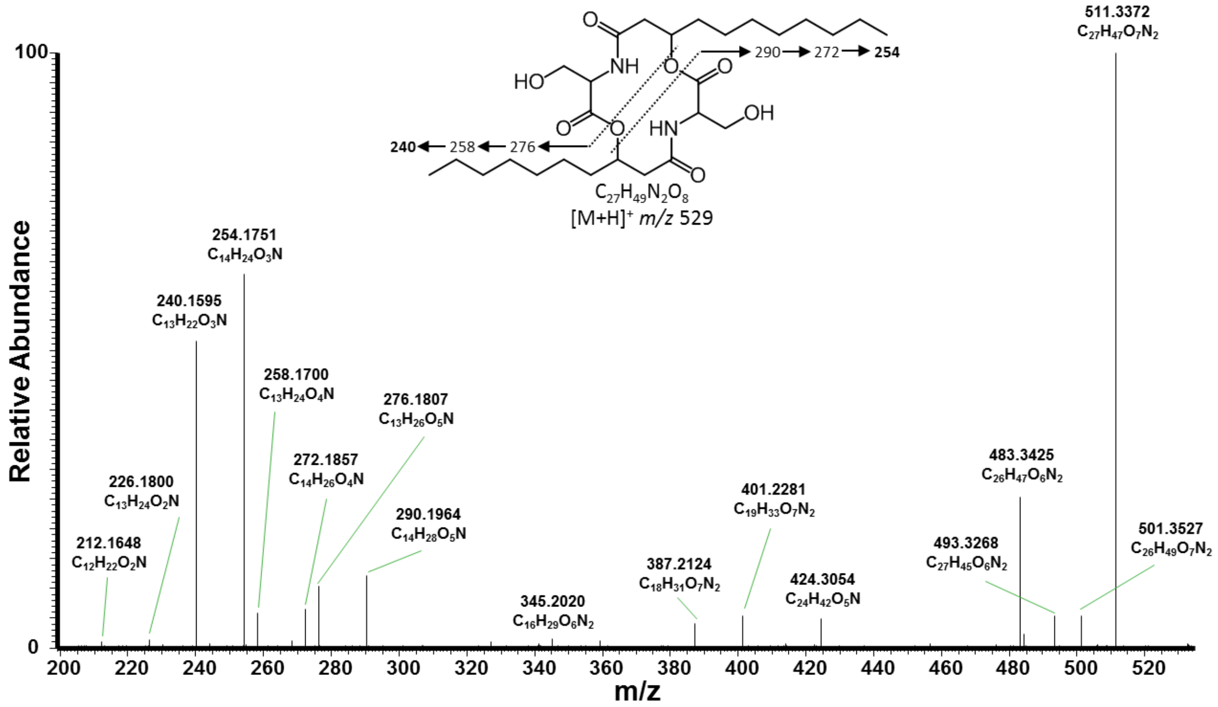

Fig. S35. Proposed mass spectral fragmentation pathway of serratamolide C_{10}+C_{11} (compound **11** $[M+H]^+$: *m/z* 529, $C_{27}H_{49}N_2O_8$).

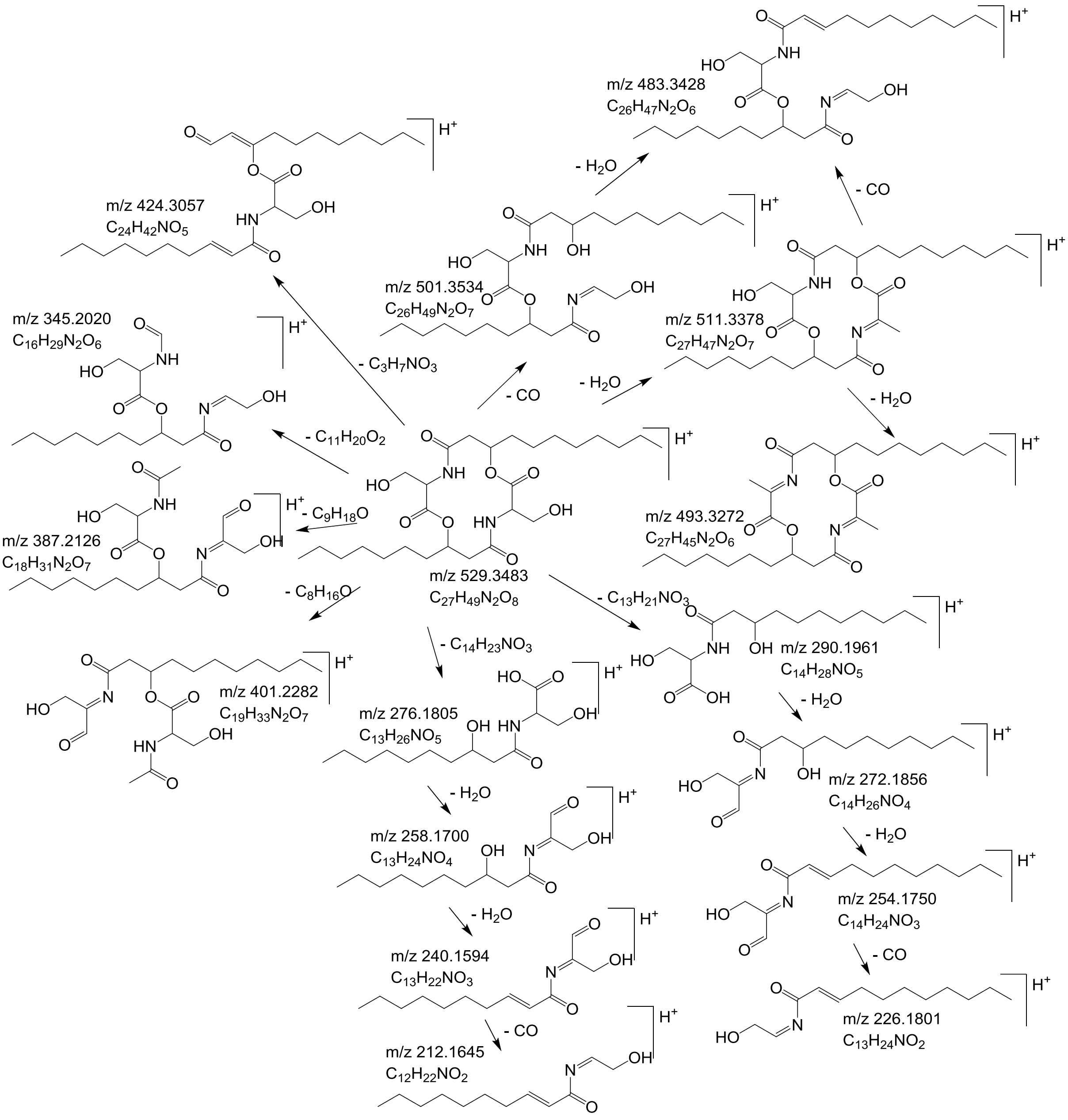

Fig. S36. HRMS[2] of serratamolide C_{10}+C_{12} (compound **12** $[M+H]^+$: *m/z* 543, $C_{28}H_{51}N_2O_8$).

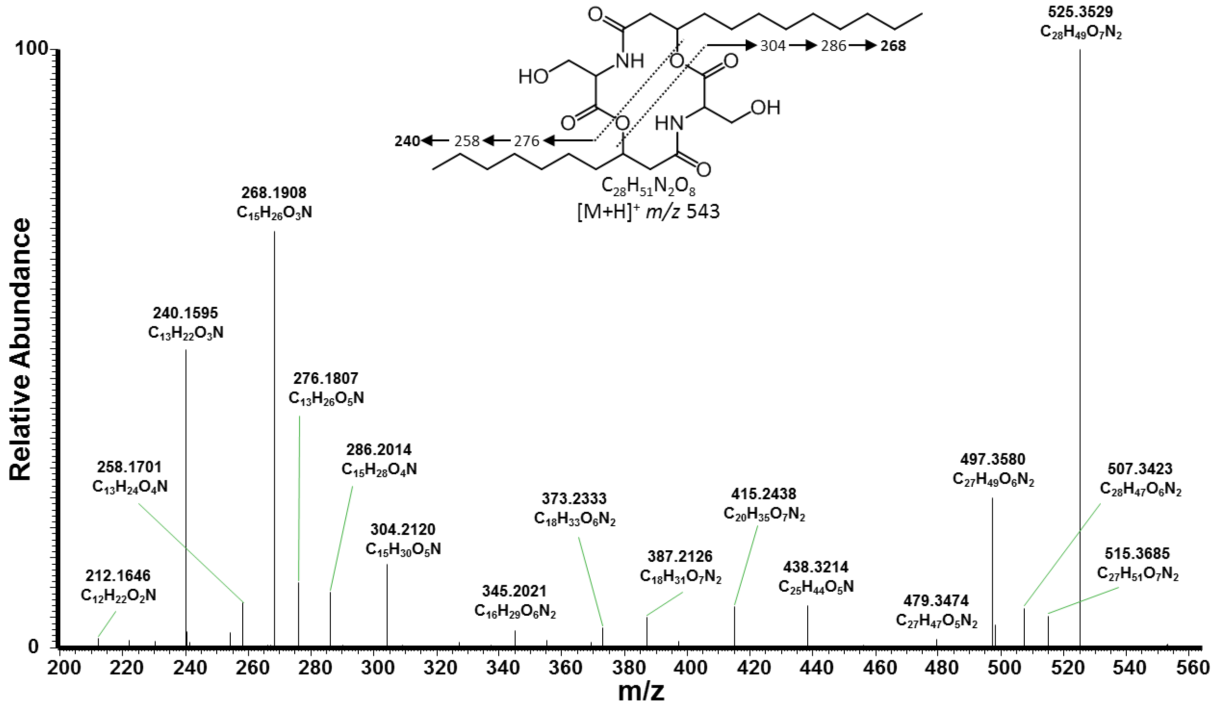

Fig. S37. Proposed mass spectral fragmentation pathway of serratamolide C_{10}+C_{12} (compound **12** $[M+H]^+$: *m/z* 543, $C_{28}H_{51}N_2O_8$).

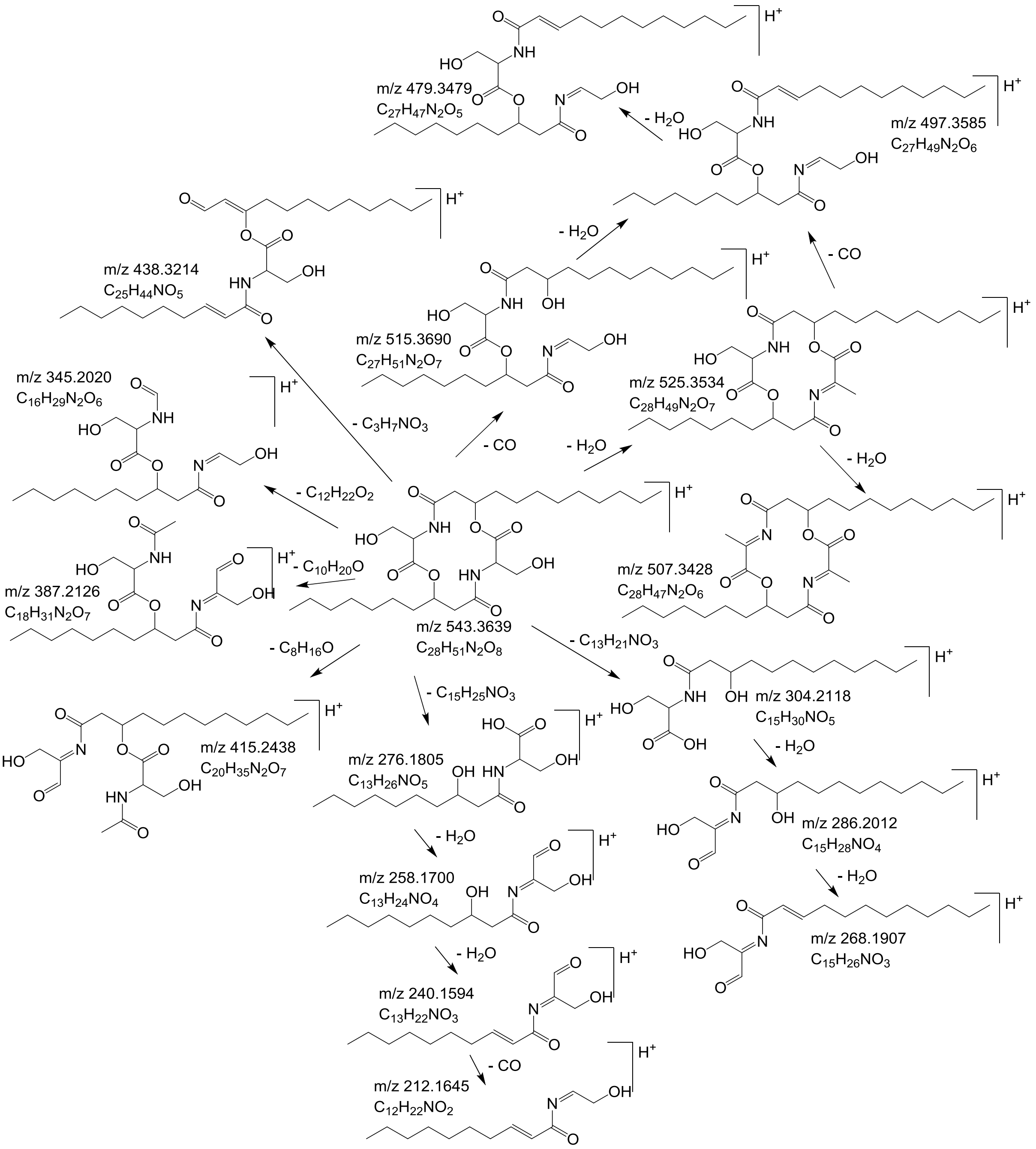

Fig. S38. HRMS[2] of serratamolide C_{11}+C_{12} (compound **13** $[M+H]^+$: *m/z* 557, $C_{29}H_{53}N_2O_8$).

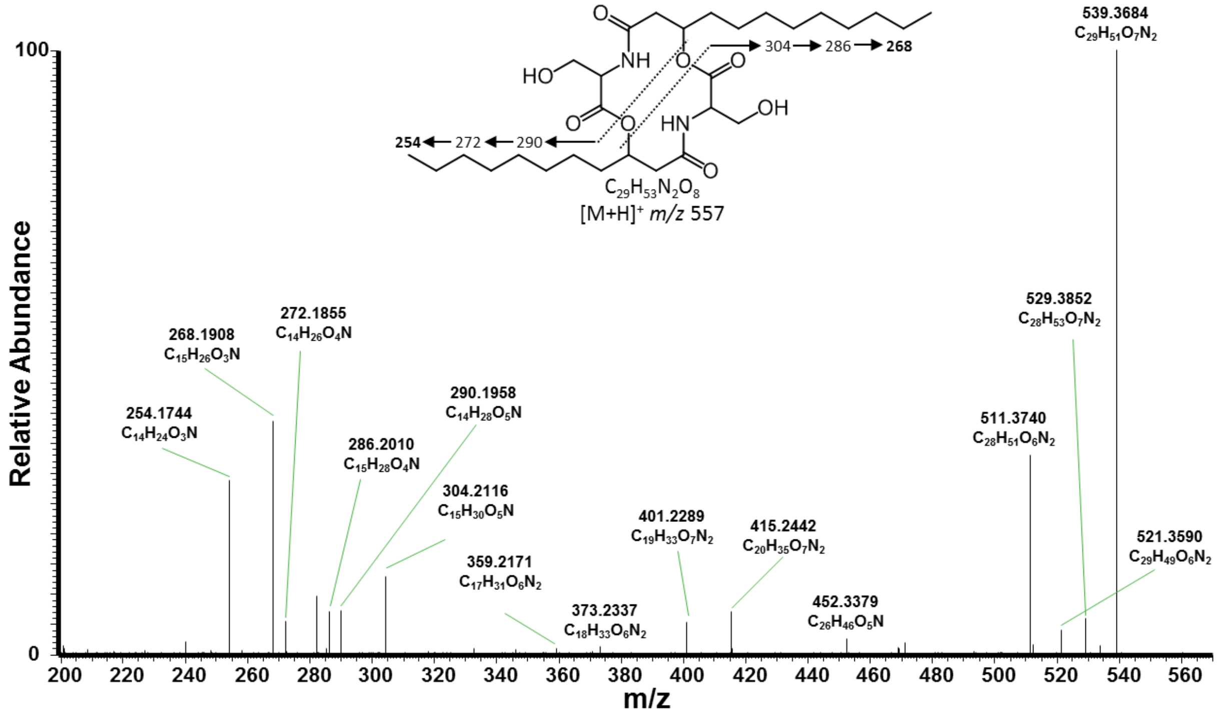

Fig. S39. Proposed mass spectral fragmentation pathway of serratamolide C_{11}+C_{12} (compound **13** $[M+H]^+$: *m/z* 557, $C_{29}H_{53}N_2O_8$).

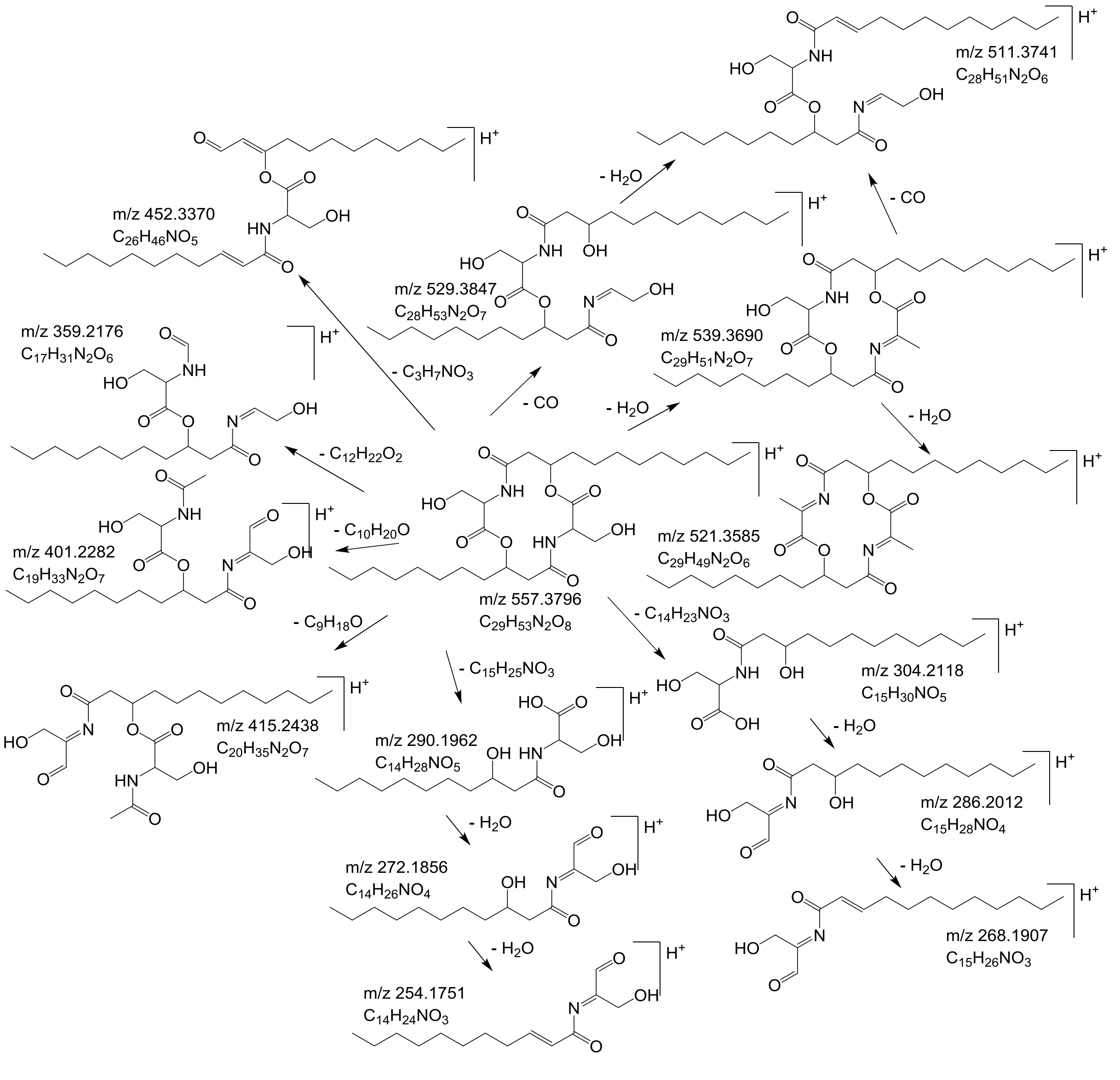

Fig. S40. HRMS[2] of serratamolide C_{12}+C_{12} (compound **14** $[M+H]^+$: *m/z* 571, $C_{30}H_{55}N_2O_8$).

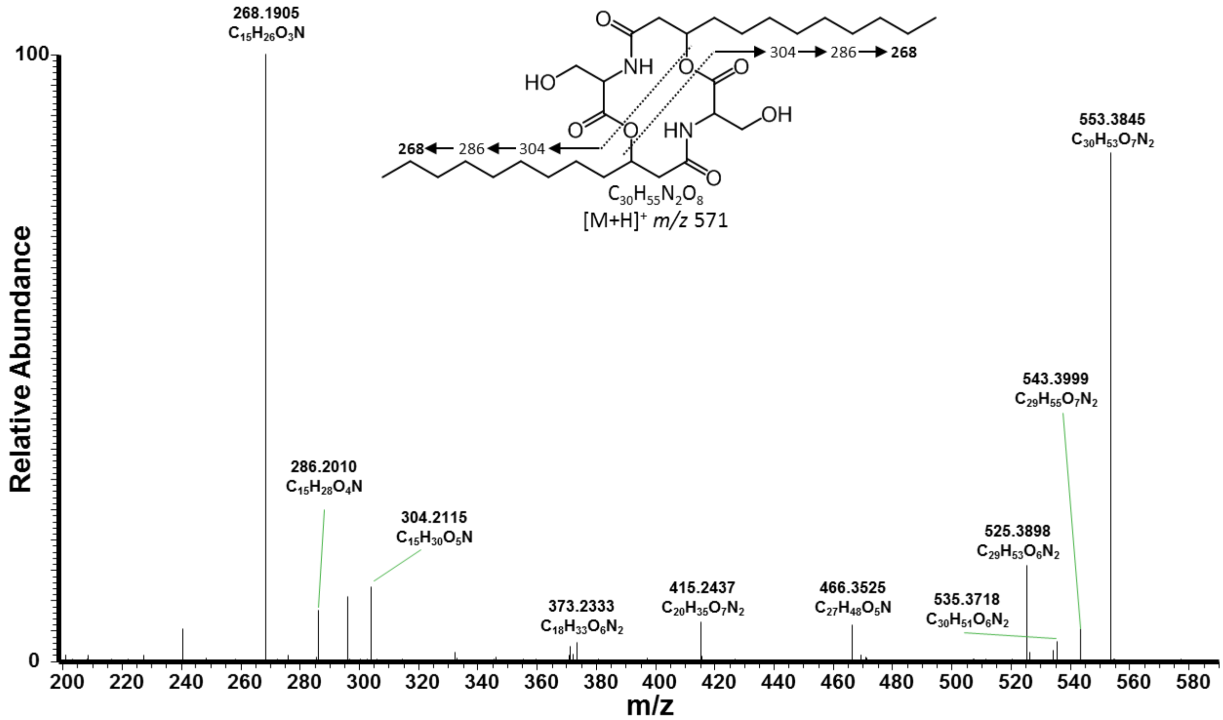

Fig. S41. Proposed mass spectral fragmentation pathway of serratamolide C_{12}+C_{12} (compound **14** $[M+H]^+$: *m/z* 571, $C_{30}H_{55}N_2O_8$).

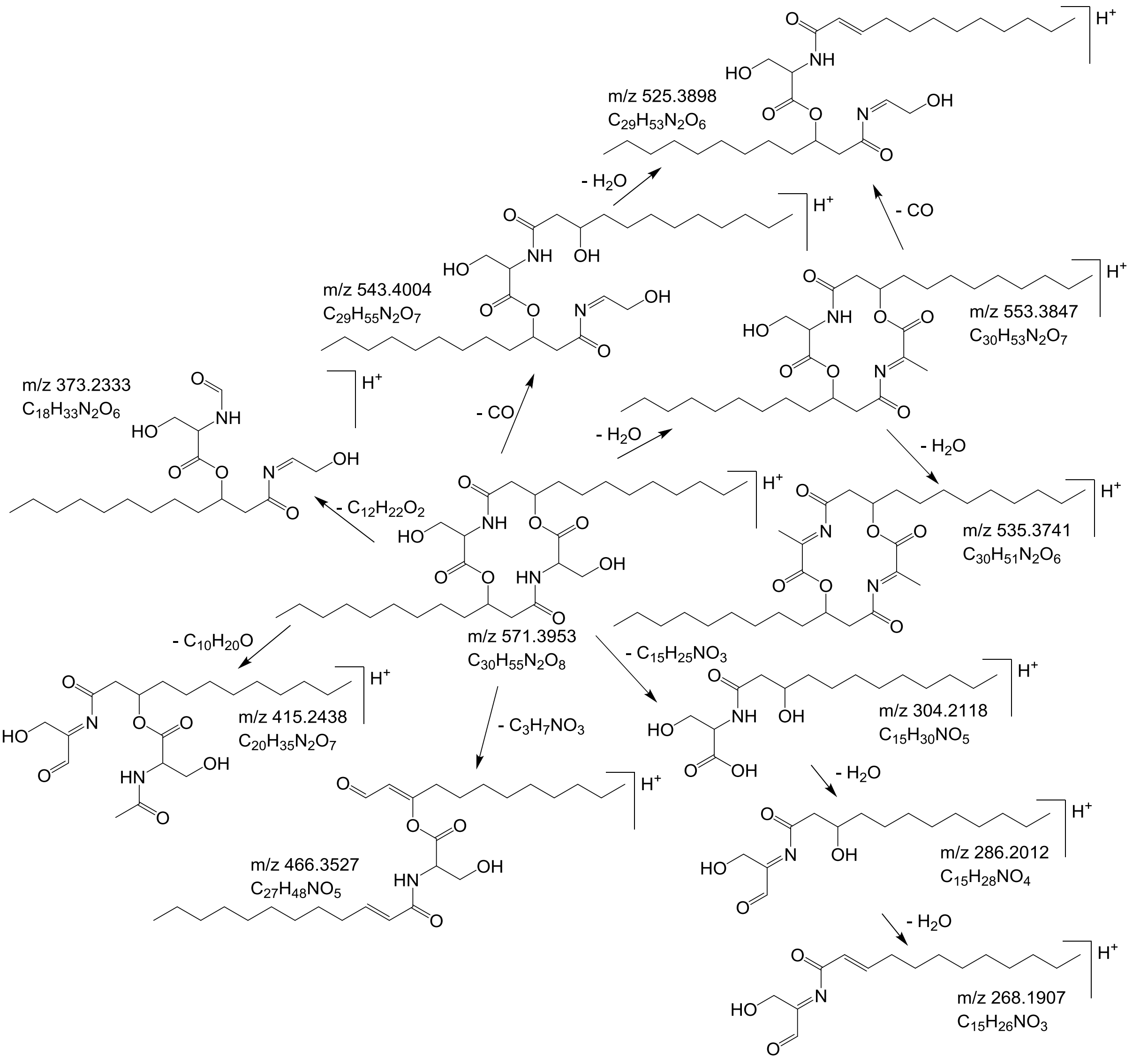

Fig. S42. HRMS[2] of serratamolide C_8+$C_{12:1}$ (compound **15** $[M+H]^+$: *m/z* 513, $C_{26}H_{45}N_2O_8$).

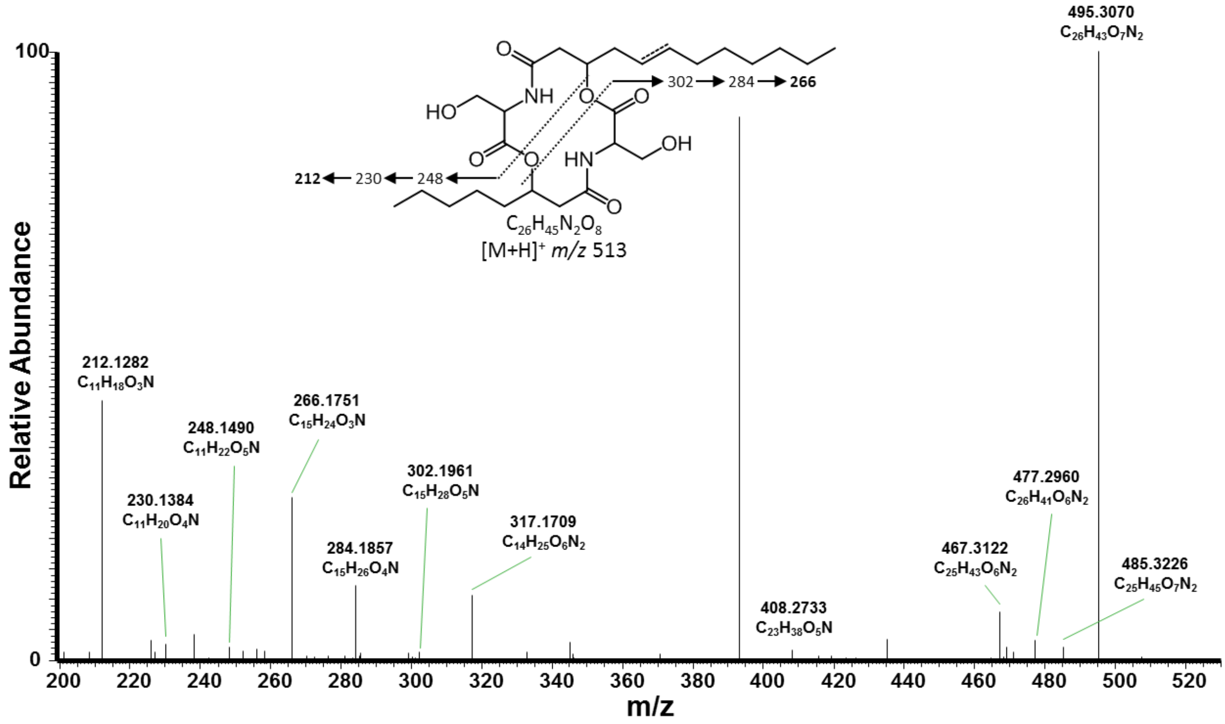

Fig. S43. Proposed mass spectral fragmentation pathway of serratamolide C_8+$C_{12:1}$ (compound **15** $[M+H]^+$: *m/z* 513, $C_{26}H_{45}N_2O_8$).

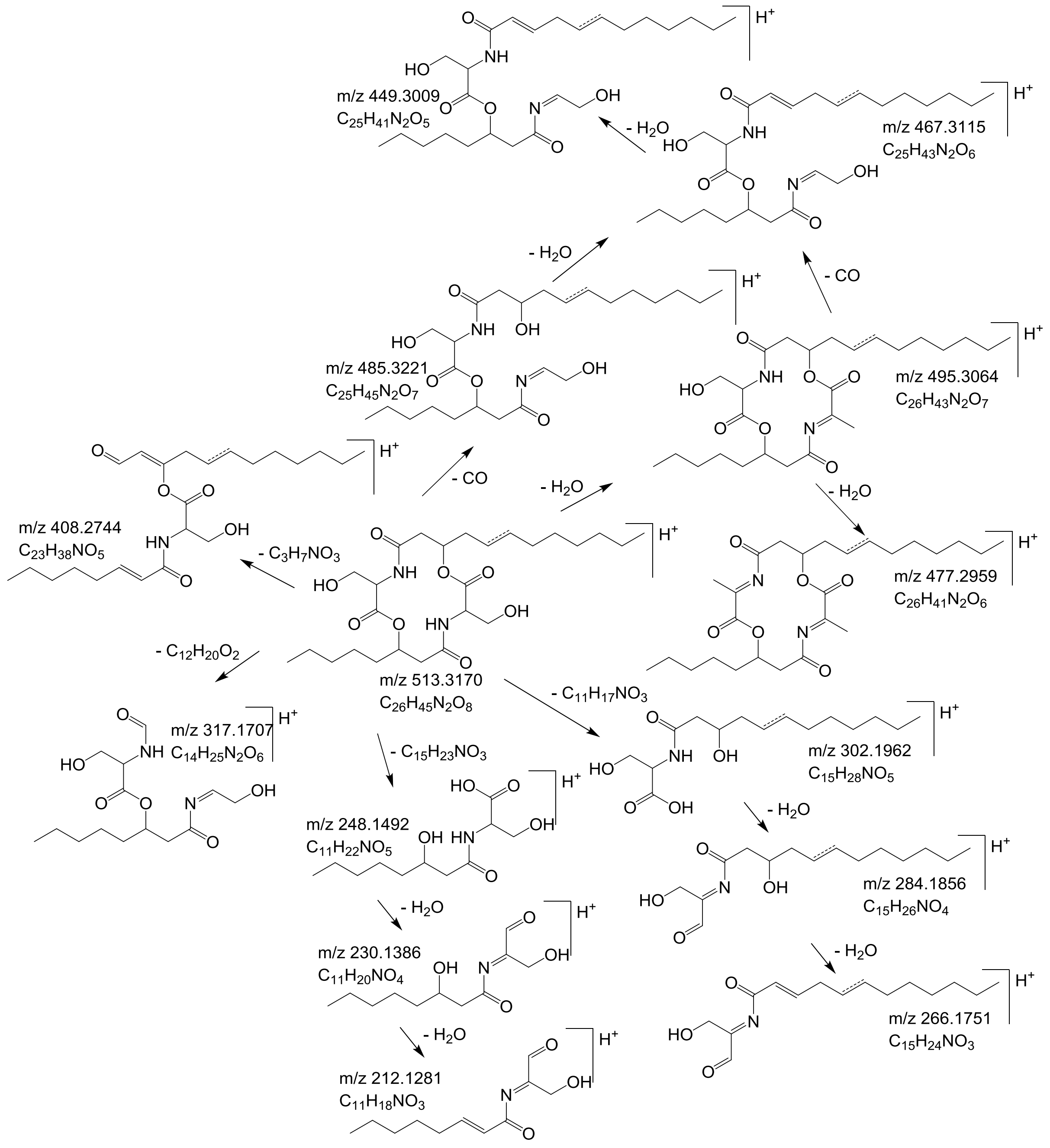

Fig. S44. HRMS^2 of serratamolide C_{10}+$C_{11:1}$ (compound **16** $[M+H]^+$: *m/z* 527, $C_{27}H_{47}N_2O_8$).

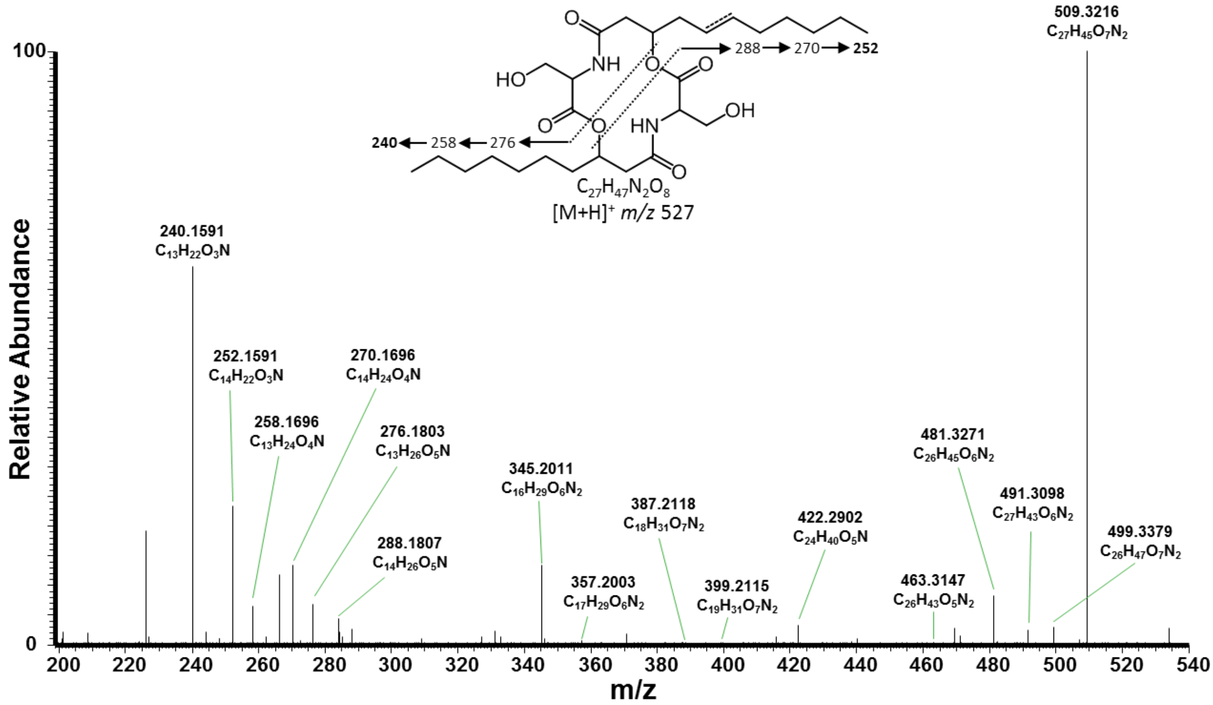

Fig. S45. Proposed mass spectral fragmentation pathway of serratamolide C_{10}+$C_{11:1}$ (compound **16** $[M+H]^+$: *m/z* 527, $C_{27}H_{47}N_2O_8$).

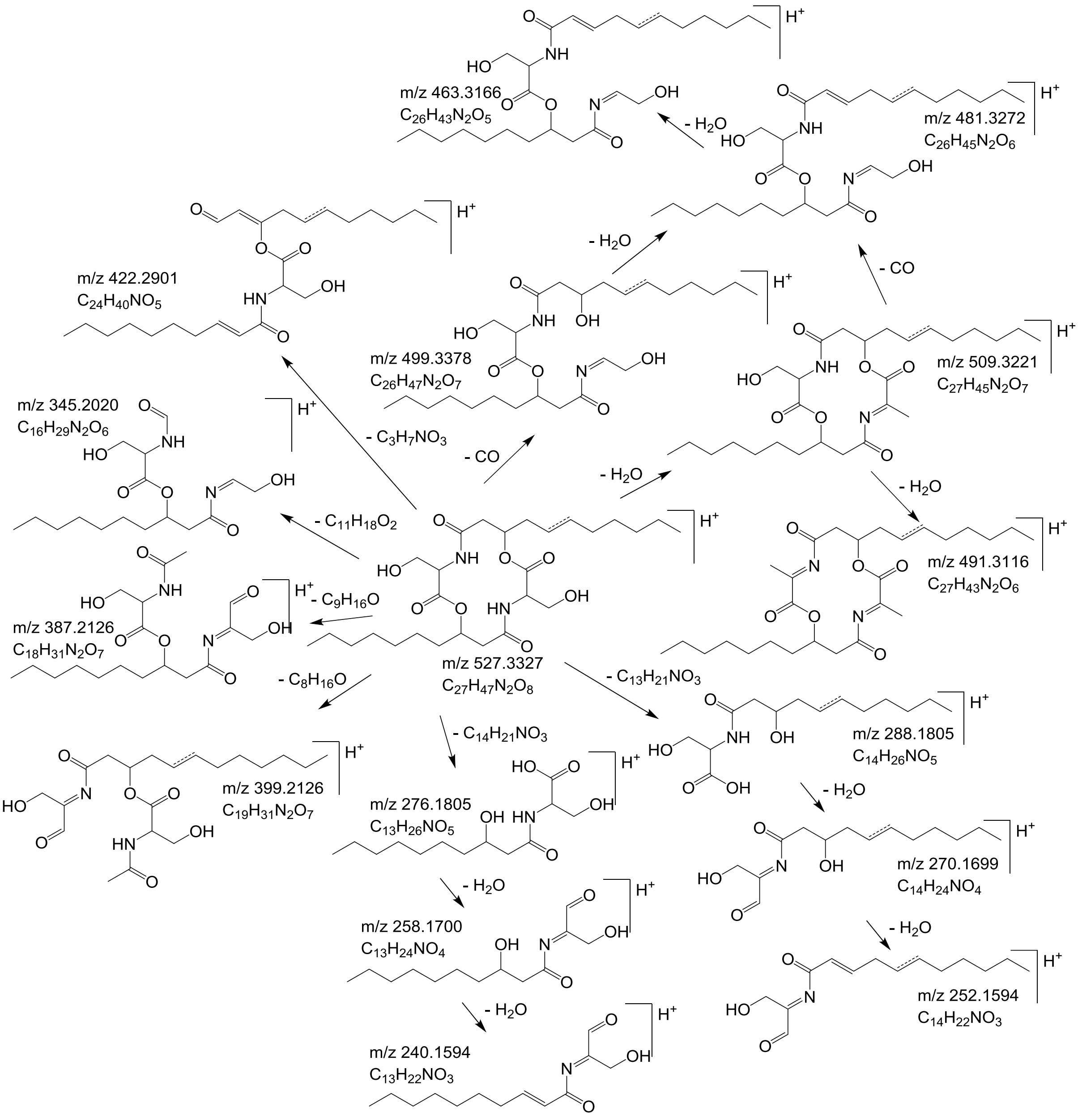

Fig. S46. HRMS[2] of serratamolide C_{10}+$C_{12:1}$ (compound **17** $[M+H]^+$: *m/z* 541, $C_{28}H_{49}N_2O_8$).

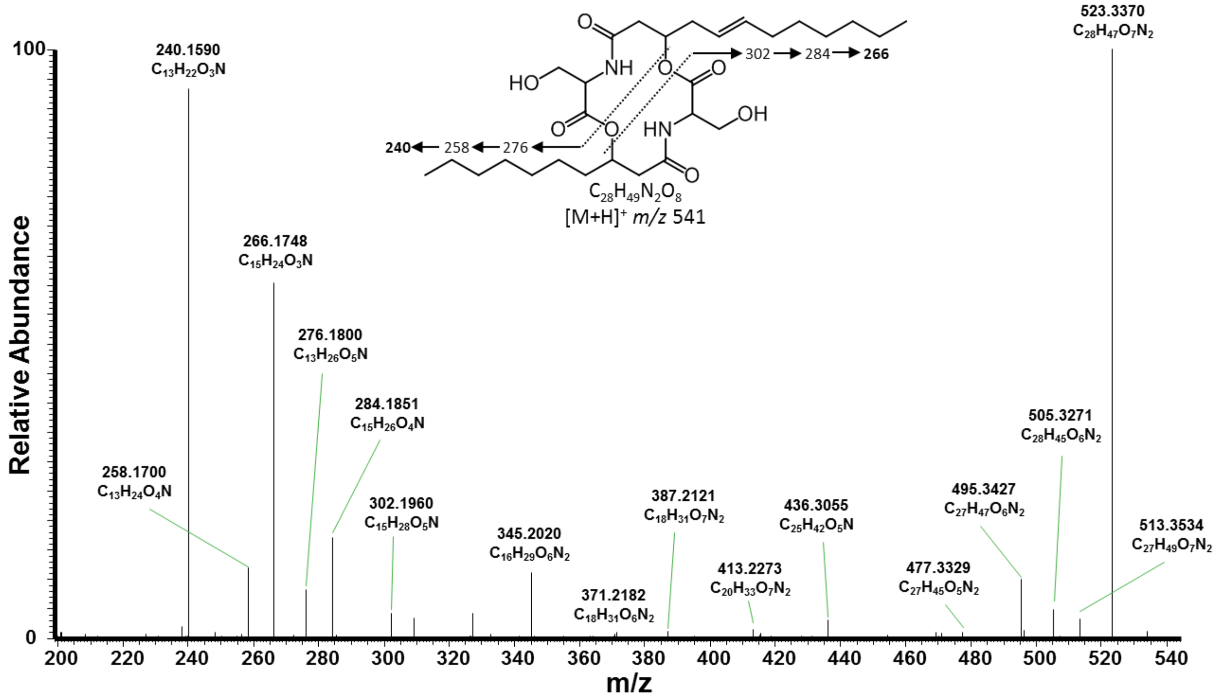

Fig. S47. Proposed mass spectral fragmentation pathway of serratamolide C_{10}+$C_{12:1}$ (compound **17** $[M+H]^+$: *m/z* 541, $C_{28}H_{49}N_2O_8$).

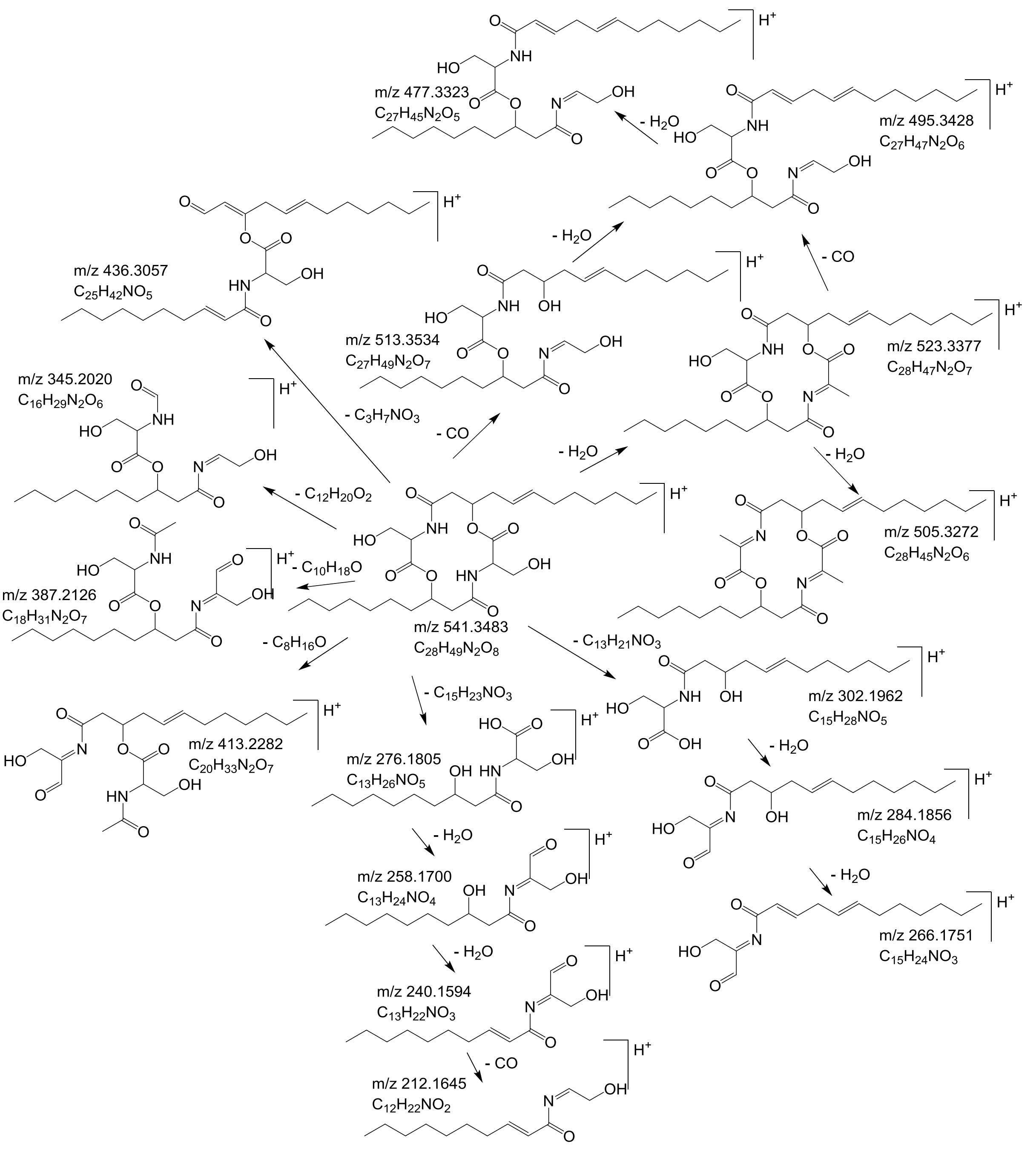

Fig. S48. HRMS[2] of serratamolide C_{11}+$C_{12:1}$ (compound **18** $[M+H]^+$: *m/z* 555, $C_{29}H_{51}N_2O_8$).

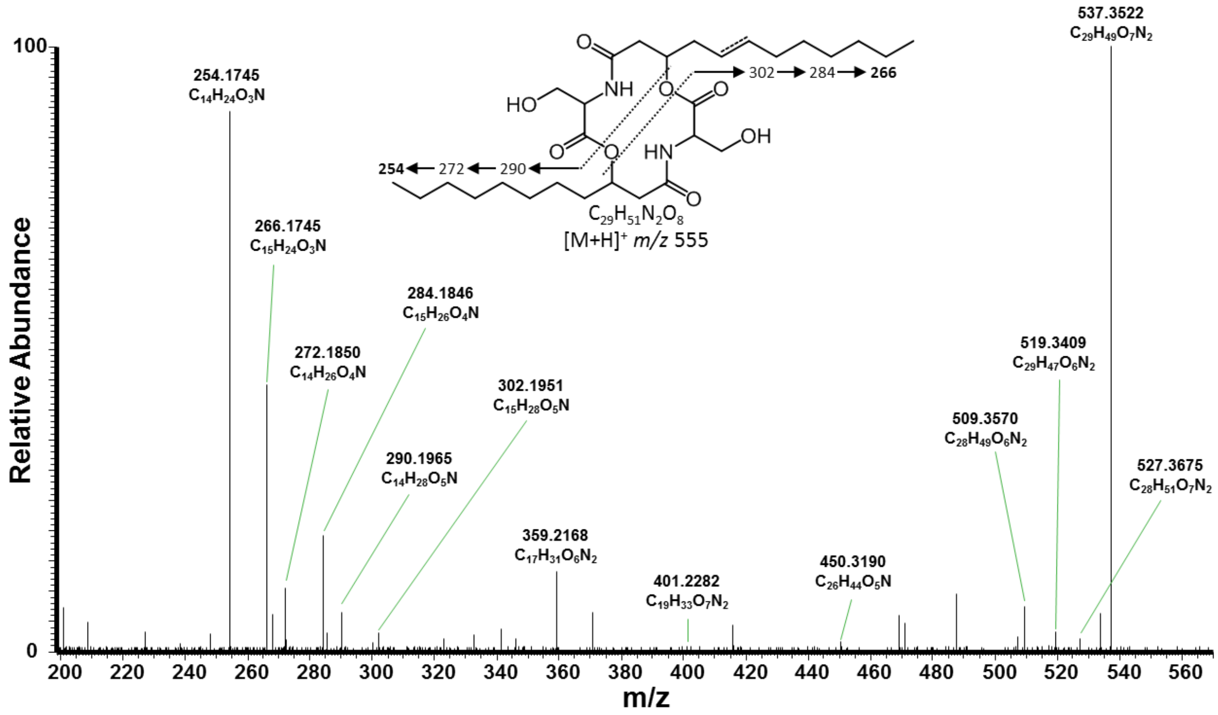

Fig. S49. Proposed mass spectral fragmentation pathway of serratamolide C_{11}+$C_{12:1}$ (compound **18** $[M+H]^+$: *m/z* 555, $C_{29}H_{51}N_2O_8$).

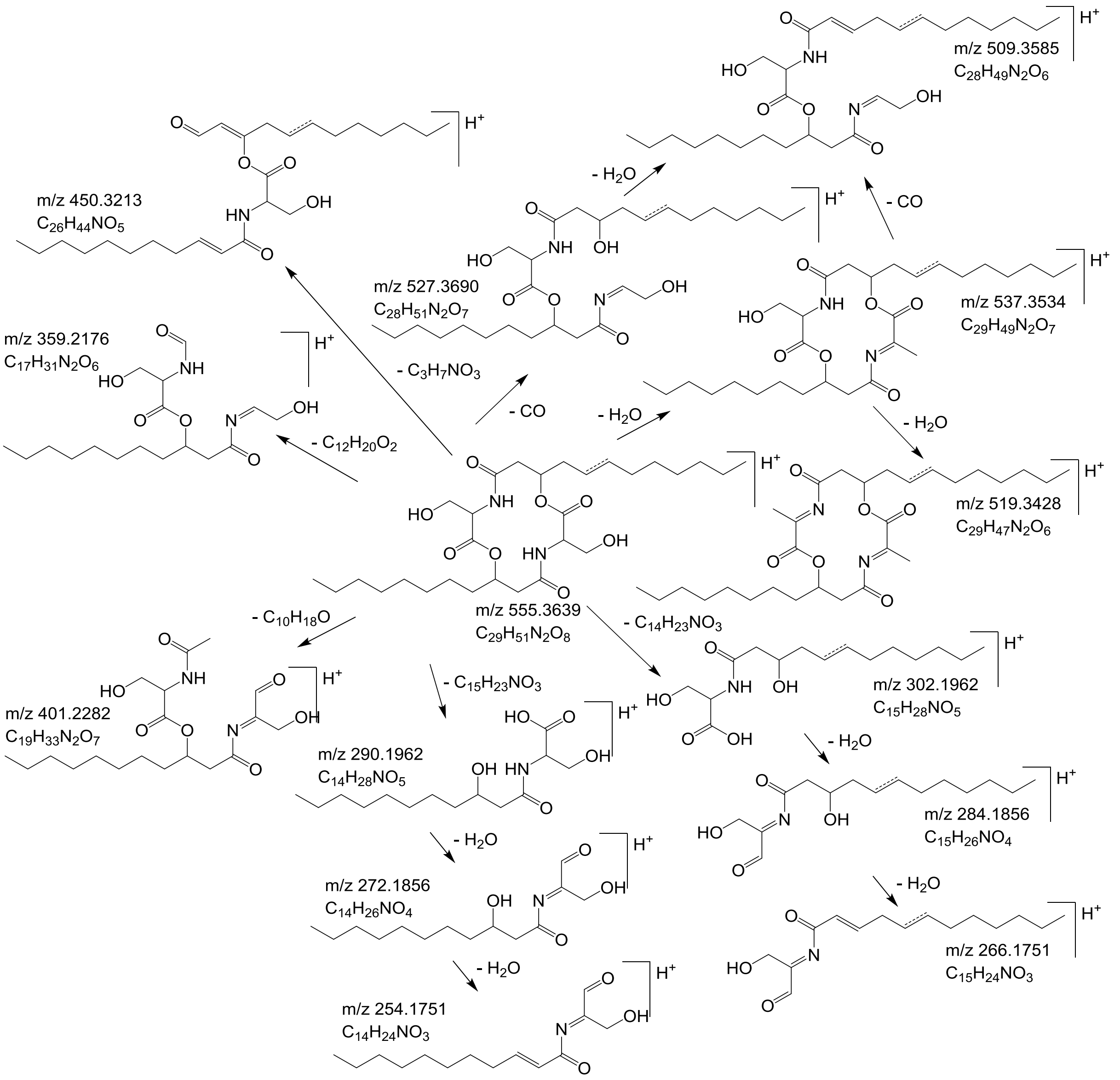

Fig. S50. HRMS[2] of serratamolide C_{12}+$C_{12:1}$ (compound **19** $[M+H]^+$: *m/z* 569, $C_{30}H_{53}N_2O_8$).

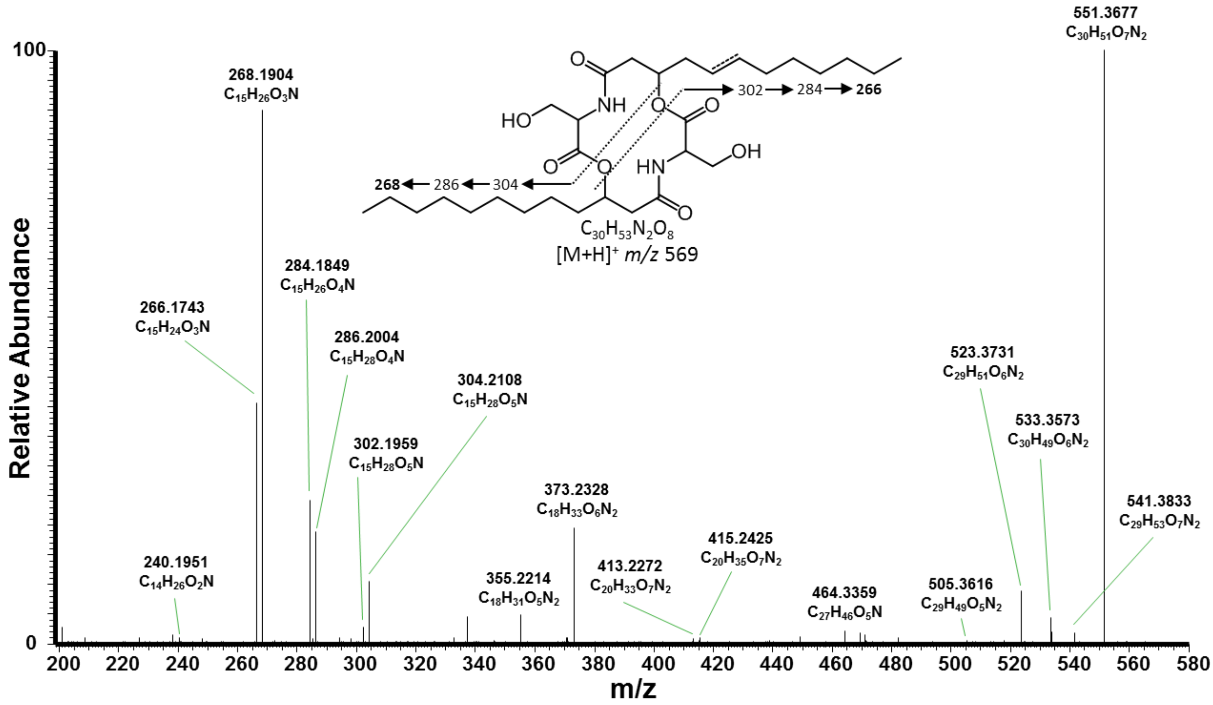

Fig. S51. Proposed mass spectral fragmentation pathway of serratamolide C_{12}+$C_{12:1}$ (compound **19** $[M+H]^+$: *m/z* 569, $C_{30}H_{53}N_2O_8$).

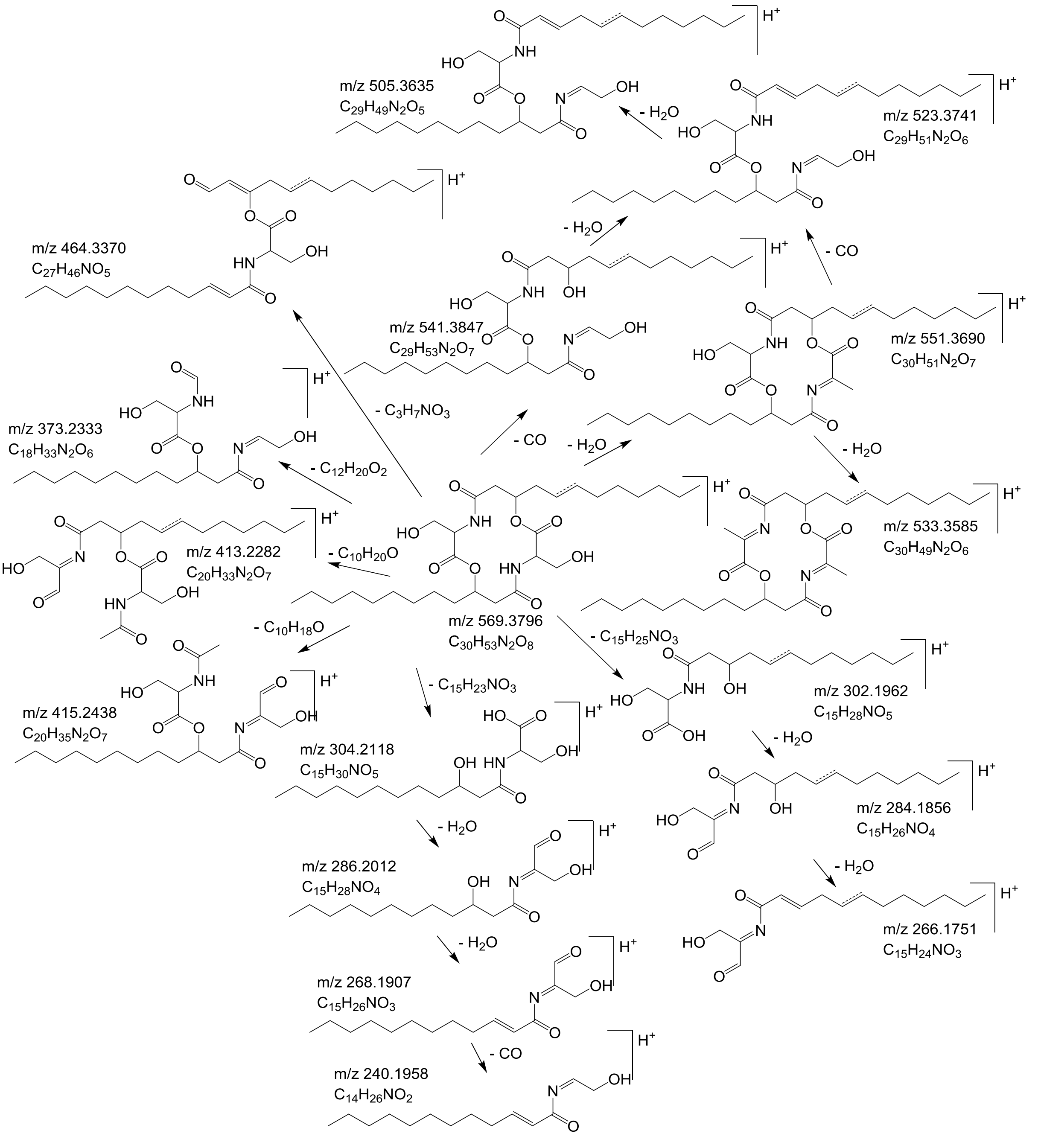

Fig. S52. HRMS[2] of ring-opened serratamolide C_{10}+C_{10} (compound **20** $[M+H]^+$: *m/z* 533, $C_{26}H_{49}N_2O_9$).

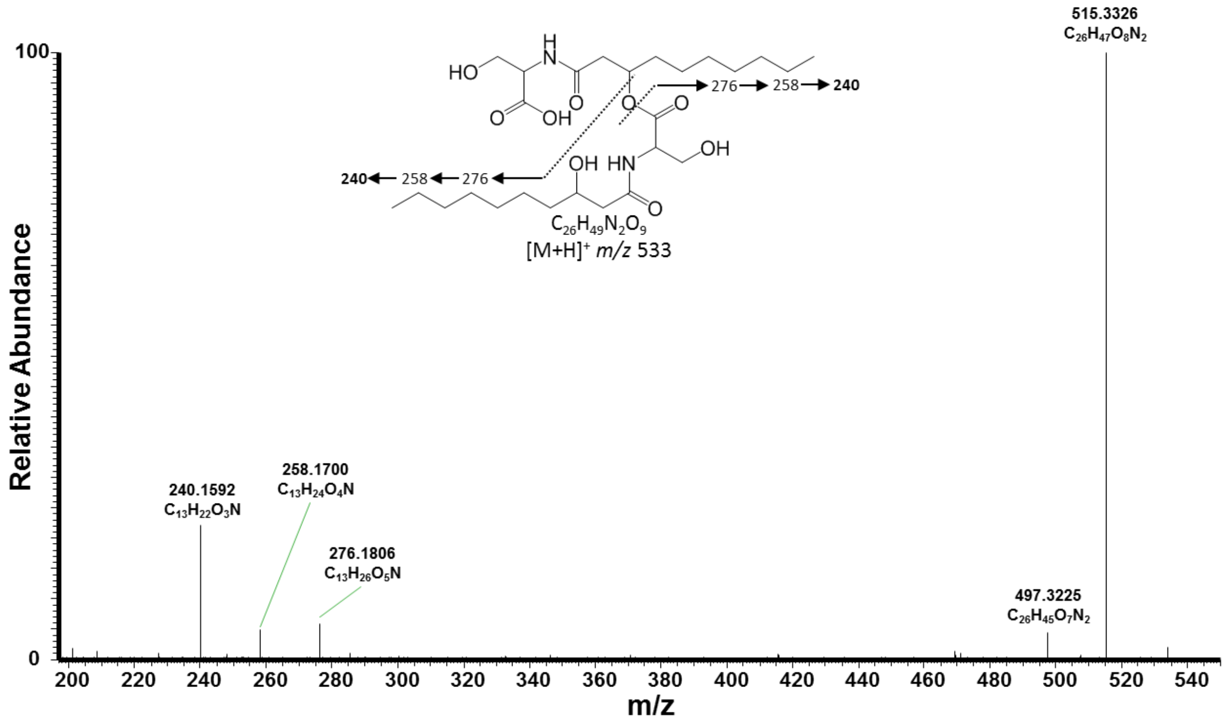

Fig. S53. Proposed mass spectral fragmentation pathway of ring-opened serratamolide C_{10}+C_{10} (compound **20** $[M+H]^+$: *m/z* 533, $C_{26}H_{49}N_2O_9$).

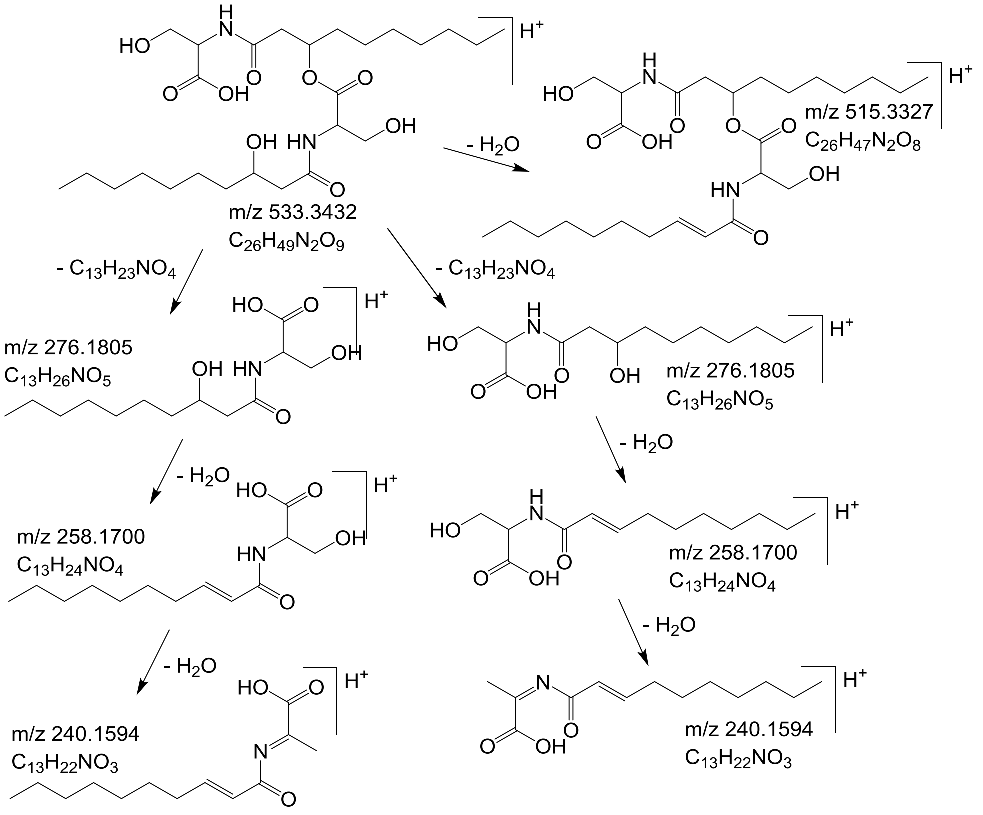

Fig. S54. HRMS[2] of ring-opened serratamolide C_{10}+C_{11} (compound **21** $[M+H]^+$: *m/z* 547, $C_{27}H_{51}N_2O_9$).

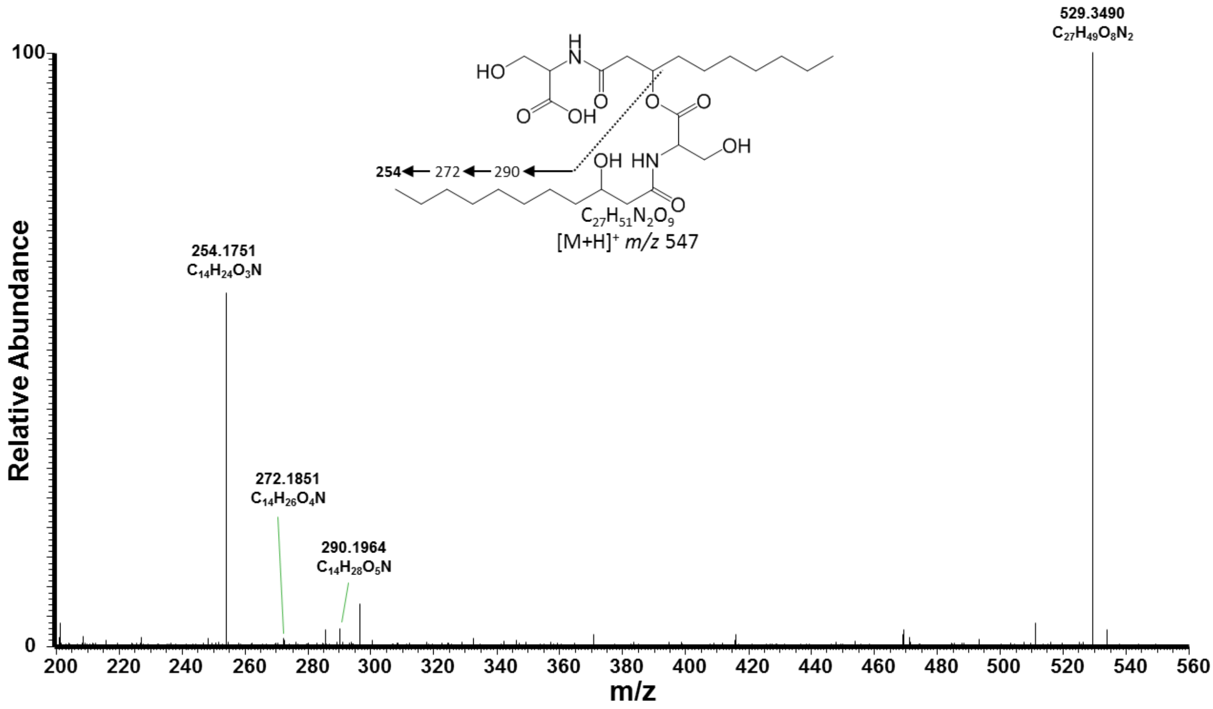

Fig. S55. Proposed mass spectral fragmentation pathway of ring-opened serratamolide C_{10}+C_{11} (compound **21** $[M+H]^+$: *m/z* 547, $C_{27}H_{51}N_2O_9$).

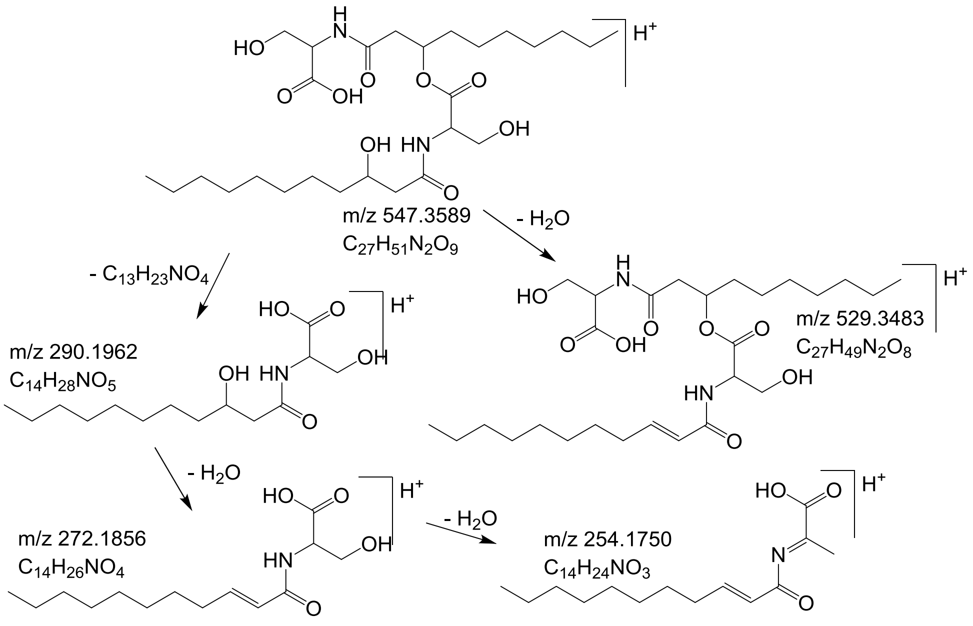

Fig. S56. HRMS[2] of ring-opened serratamolide C_{10}+C_{12} (compound **22** $[M+H]^+$: *m/z* 561, $C_{28}H_{53}N_2O_9$).

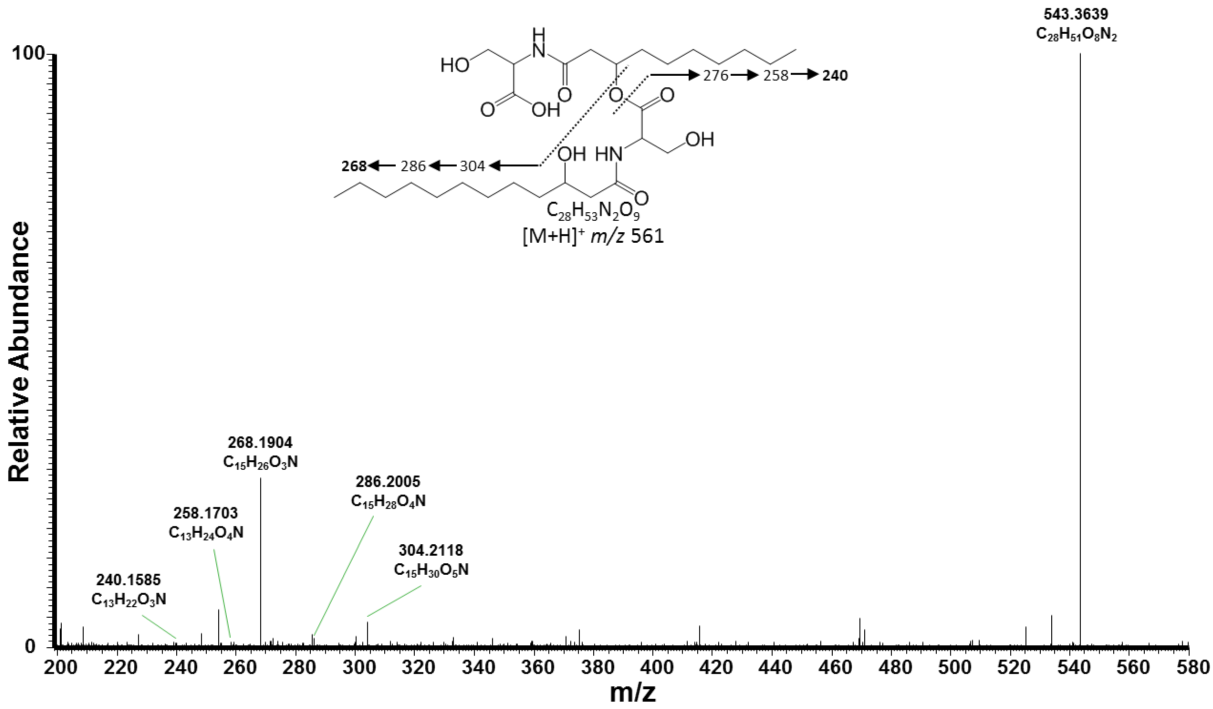

Fig. S57. Proposed mass spectral fragmentation pathway of ring-opened serratamolide C_{10}+C_{12} (compound **22** $[M+H]^+$: *m/z* 561, $C_{28}H_{53}N_2O_9$).

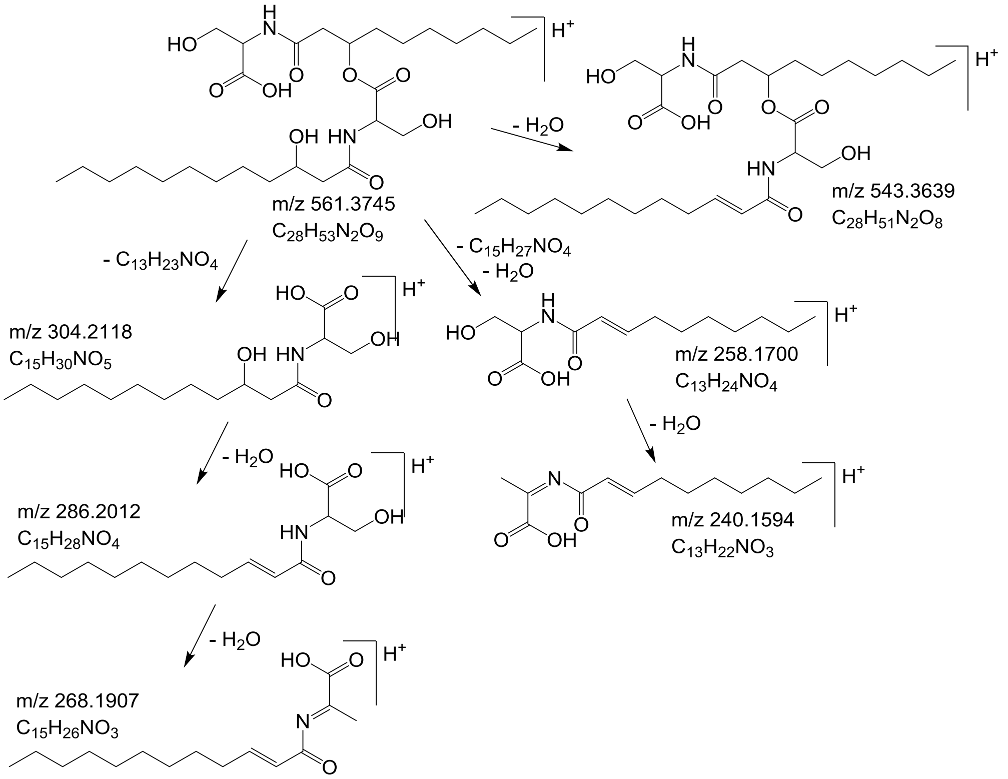

Fig. S58. HRMS[2] of ring-opened serratamolide C_{10}+$C_{12:1}$ (compound **23** $[M+H]^+$: *m/z* 559, $C_{28}H_{51}N_2O_9$).

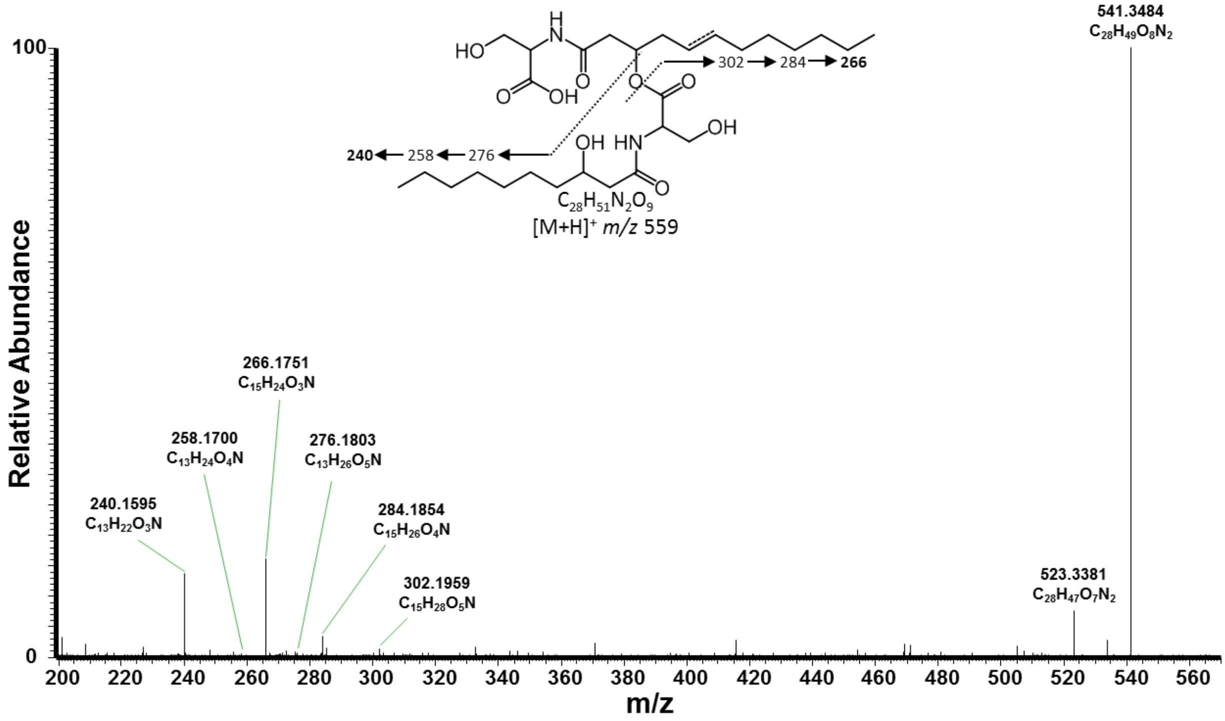

Fig. S59. Proposed mass spectral fragmentation pathway of ring-opened serratamolide C_{10}+$C_{12:1}$ (compound **23** $[M+H]^+$: *m/z* 559, $C_{28}H_{51}N_2O_9$).

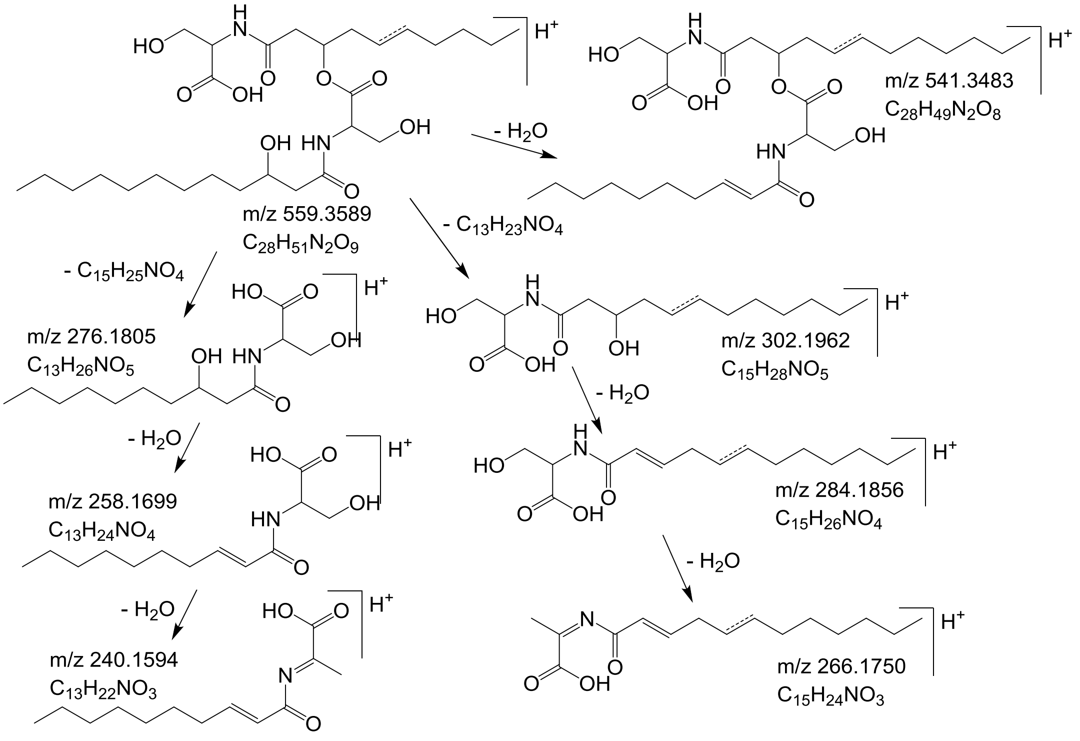

Fig. S60. HRMS[2] of ring-opened serratamolide C_9+C_{10} (compound **24** $[M+H]^+$: *m/z* 519, $C_{25}H_{47}N_2O_9$).

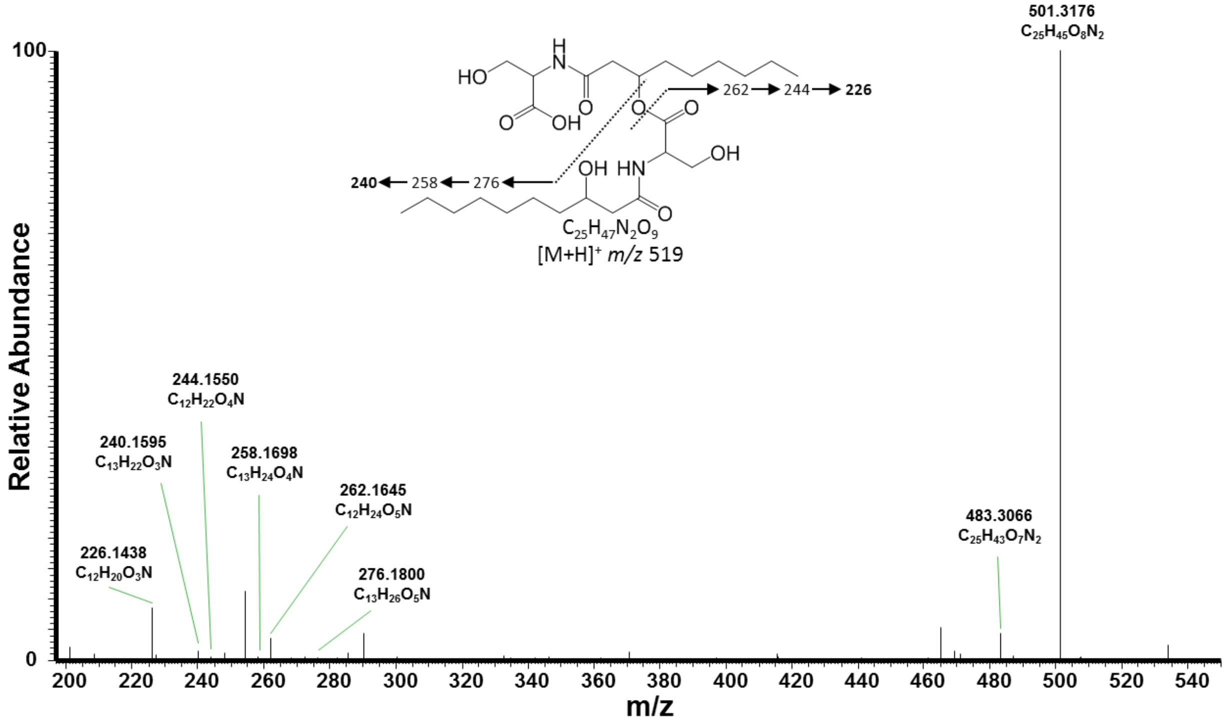

Fig. S61. Proposed mass spectral fragmentation pathway of ring-opened serratamolide C_9+C_{10} (compound **24** $[M+H]^+$: *m/z* 519, $C_{25}H_{47}N_2O_9$).

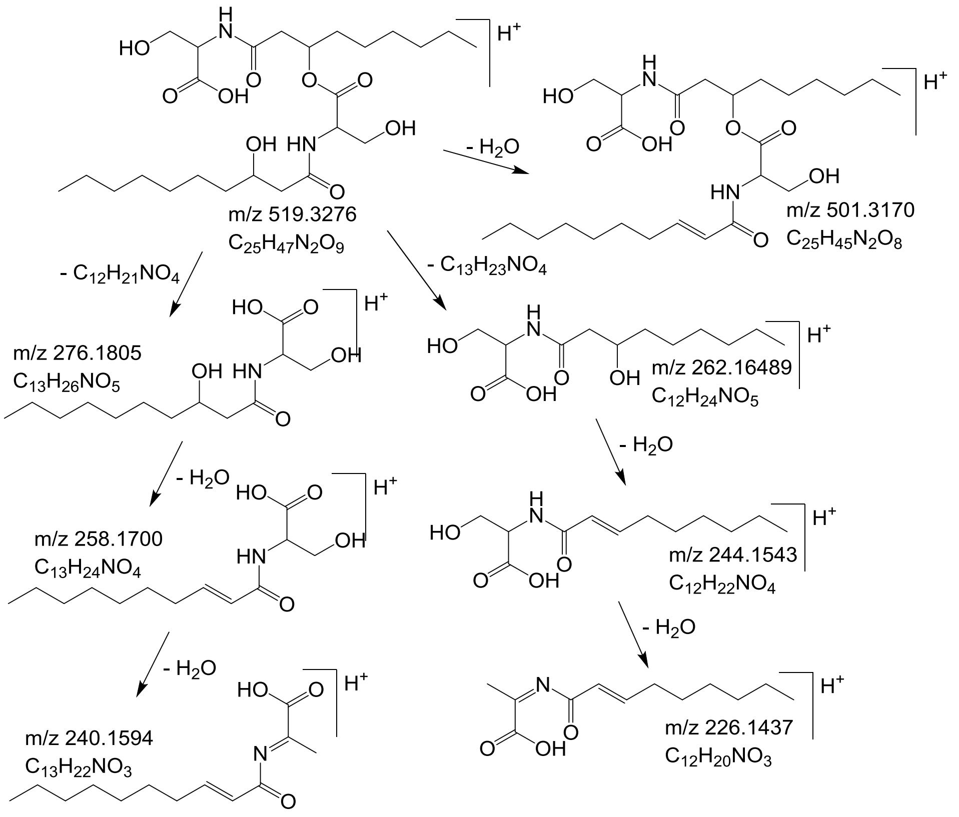

Fig. S62. HRMS[2] of ring-opened serratamolide C_{10}+C_{13} (compound **25** $[M+H]^+$: *m/z* 575, $C_{29}H_{55}N_2O_9$).

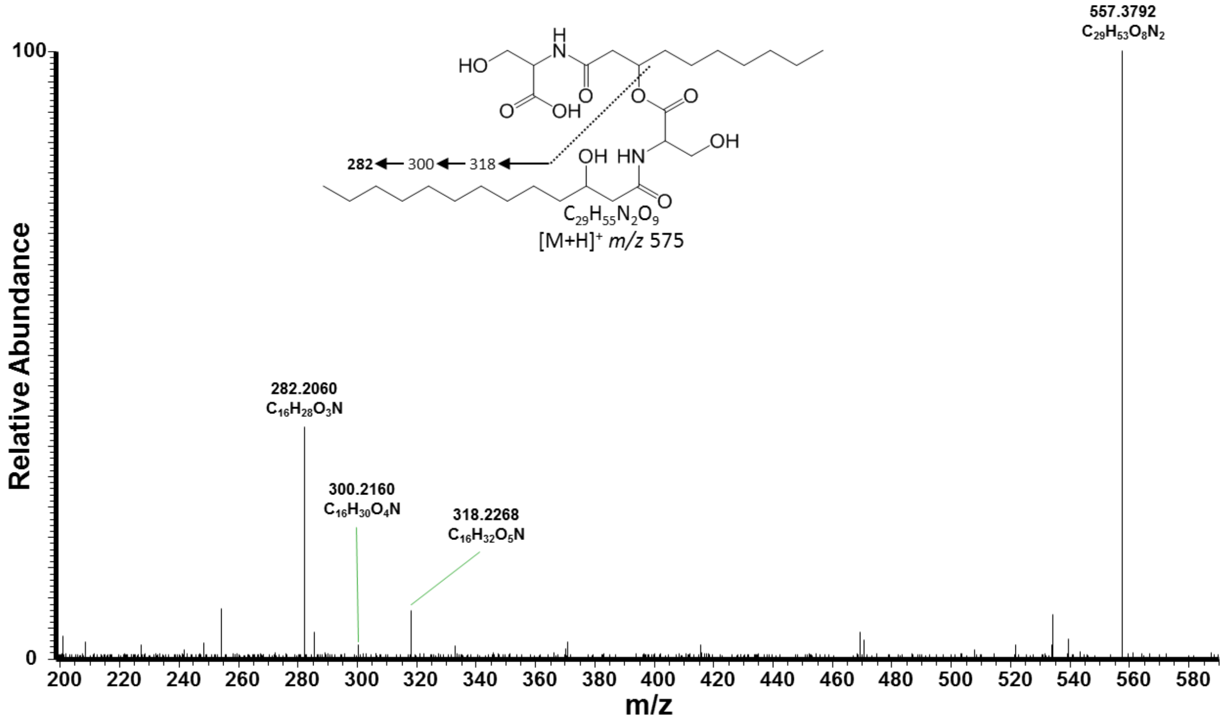

Fig. S63. Proposed mass spectral fragmentation pathway of ring-opened serratamolide C_{10}+C_{13} (compound **25** $[M+H]^+$: *m/z* 575, $C_{29}H_{55}N_2O_9$).

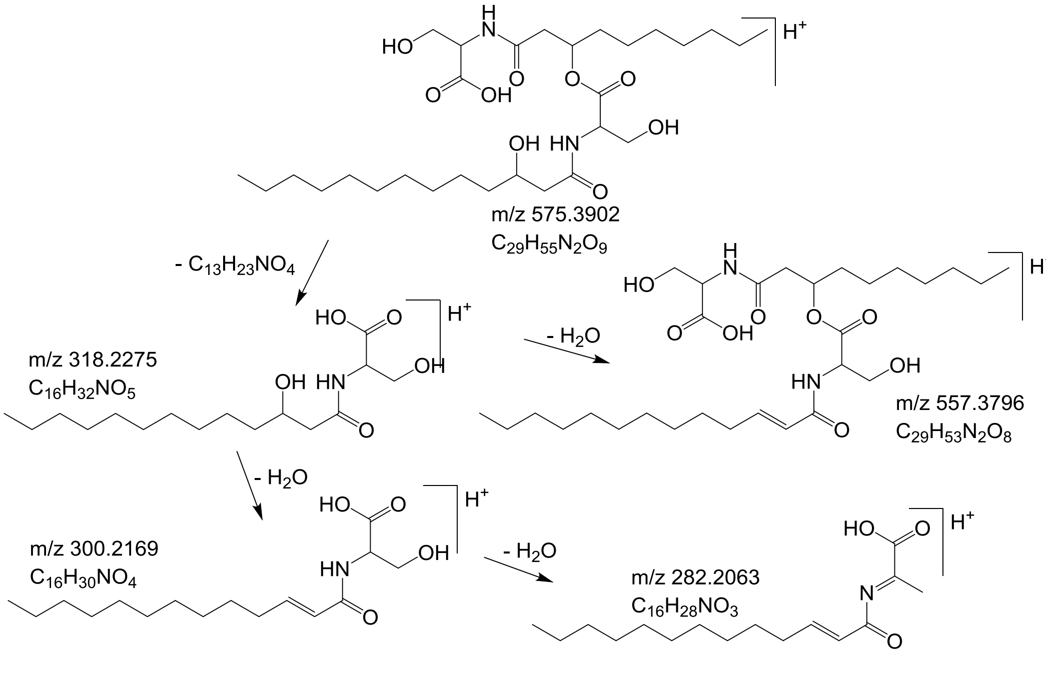

Fig. S64. HRMS^2 of ring-opened serratamolide C_{12}+C_{12} (compound **26** $[M+H]^+$: *m/z* 589, $C_{30}H_{57}N_2O_9$).

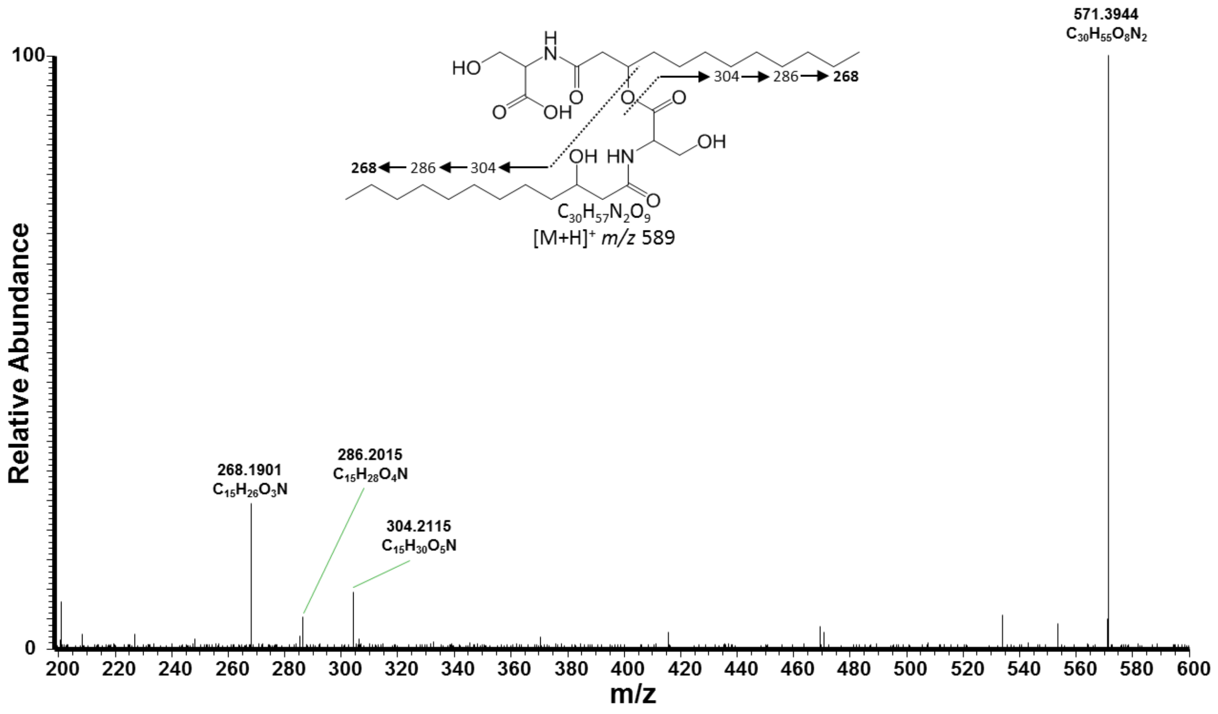

Fig. S65. Proposed mass spectral fragmentation pathway of ring-opened serratamolide C_{12}+C_{12} (compound **26** $[M+H]^+$: *m/z* 589, $C_{30}H_{57}N_2O_9$).

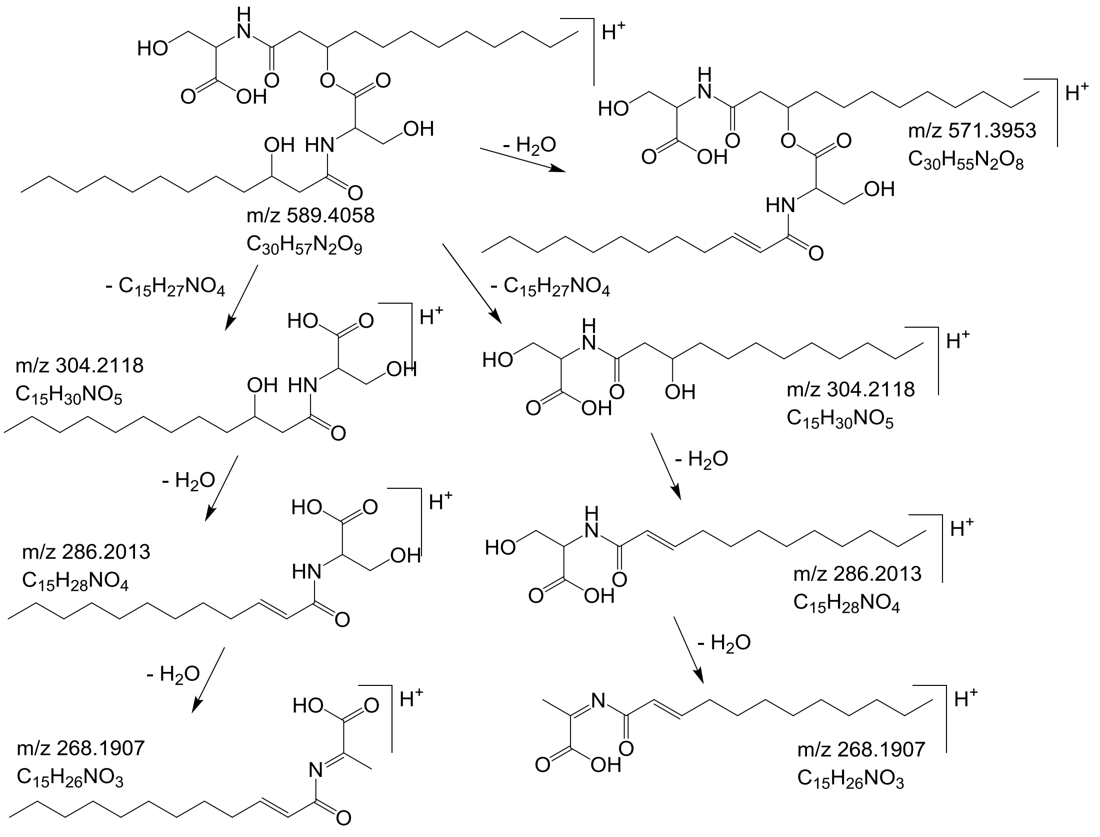

Fig. S66. HRMS[2] of ring-opened serratamolide C_{12}+C_{13} (compound **27** $[M+H]^+$: *m/z* 603, $C_{31}H_{59}N_2O_9$).

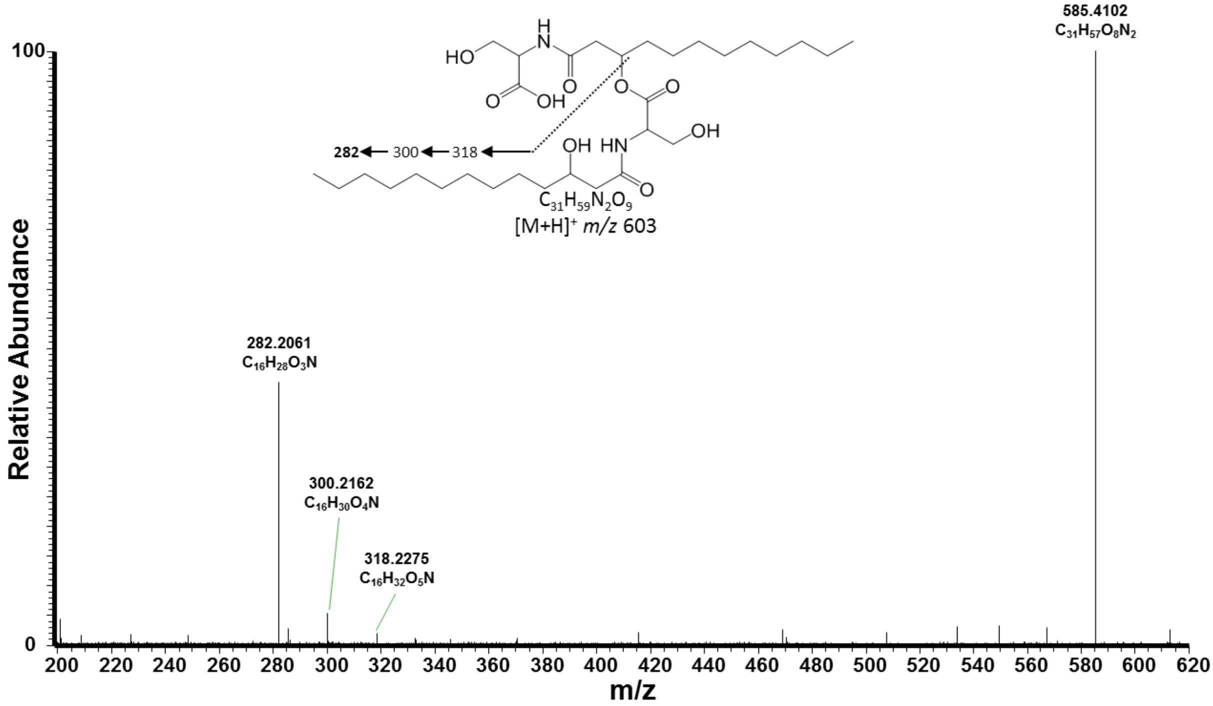

Fig. S67. Proposed mass spectral fragmentation pathway of ring-opened serratamolide C_{12}+C_{13} (compound **27** $[M+H]^+$: *m/z* 603, $C_{31}H_{59}N_2O_9$).

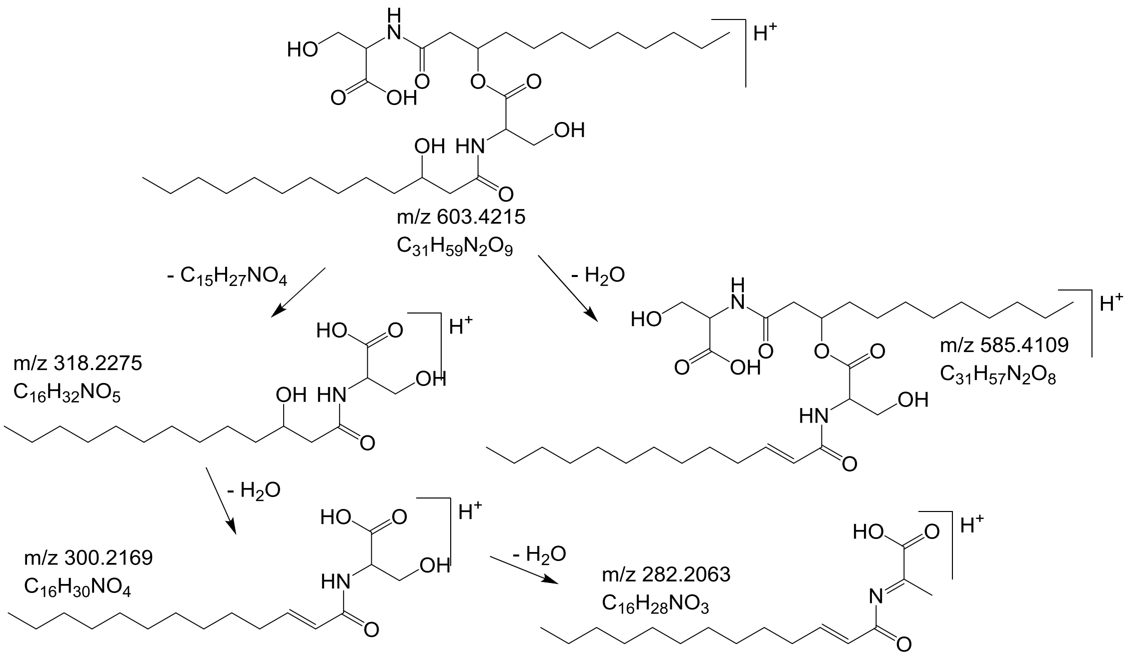

Fig. S68. HRMS^2 of ring-opened serratamolide C_{10}+$C_{13:1}$ (compound **28** $[M+H]^+$: *m/z* 573, $C_{29}H_{53}N_2O_9$).

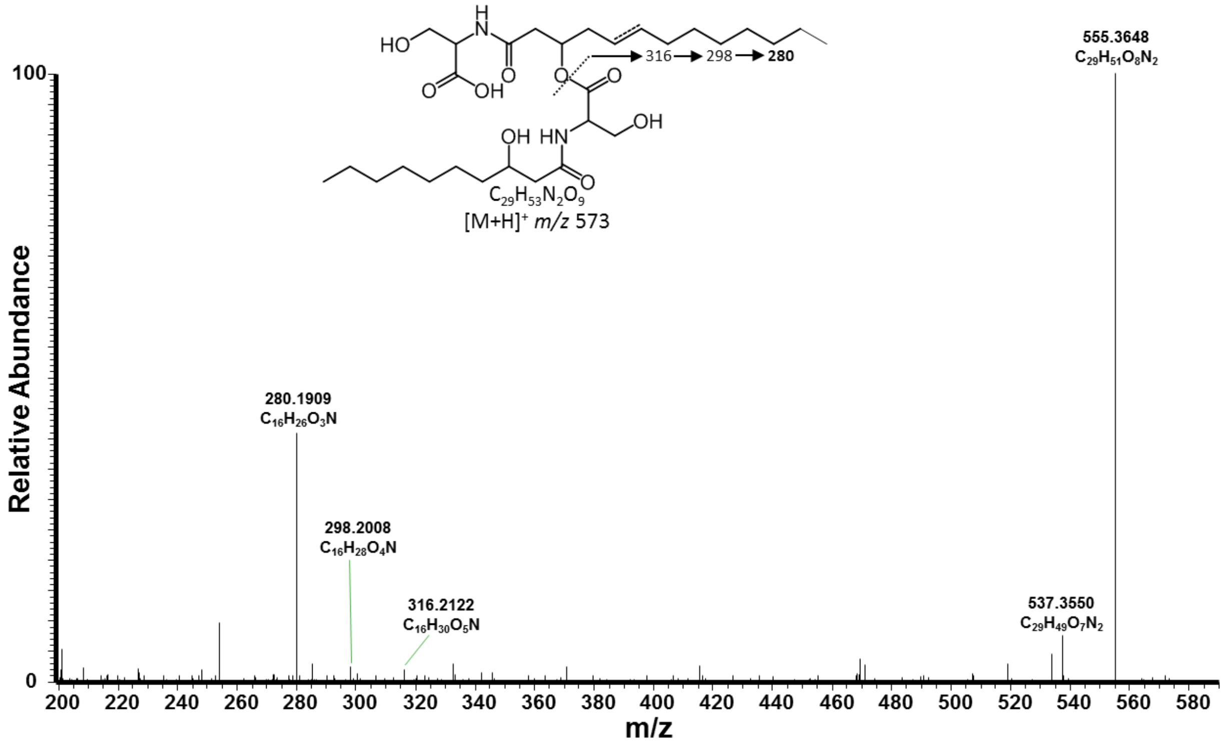

Fig. S69. HRMS^2 of ring-opened serratamolide C_{12}+$C_{12:1}$ (compound **29** $[M+H]^+$: *m/z* 587, $C_{30}H_{55}N_2O_9$).

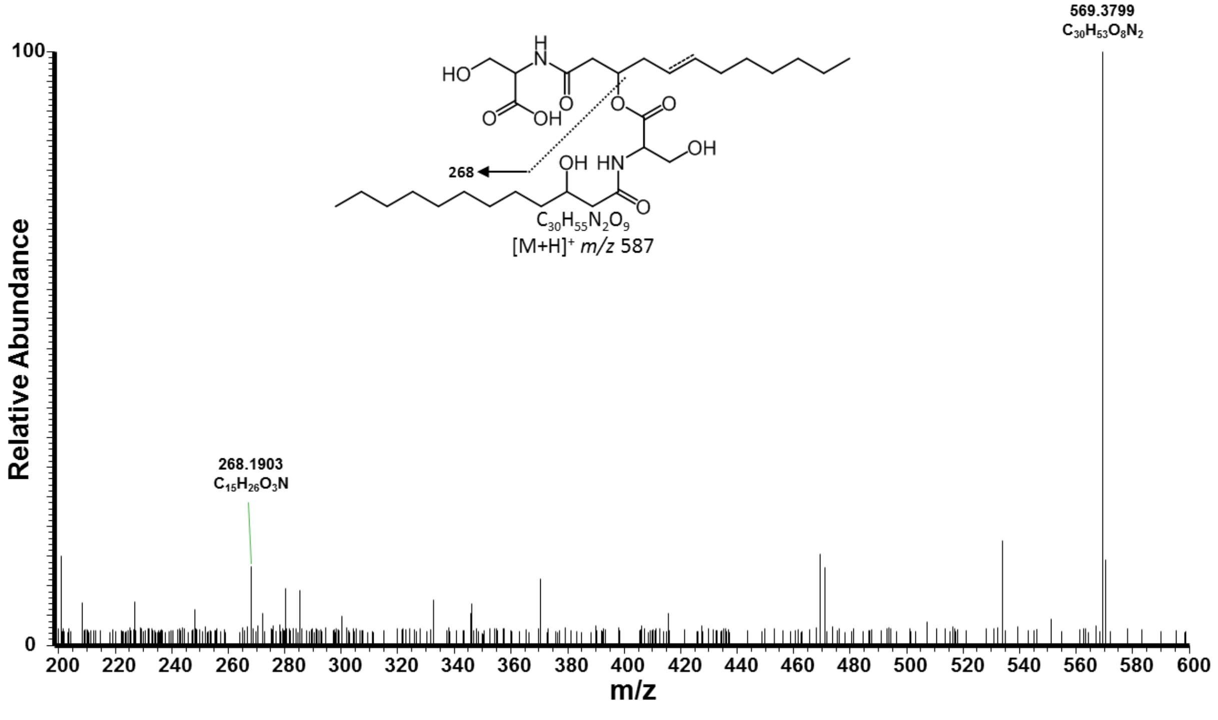

Fig. S70. HRMS[2] of ring-opened serratamolide C_{13}+$C_{12:1}$ (compound **30** $[M+H]^+$: *m/z* 601, $C_{31}H_{57}N_2O_9$).

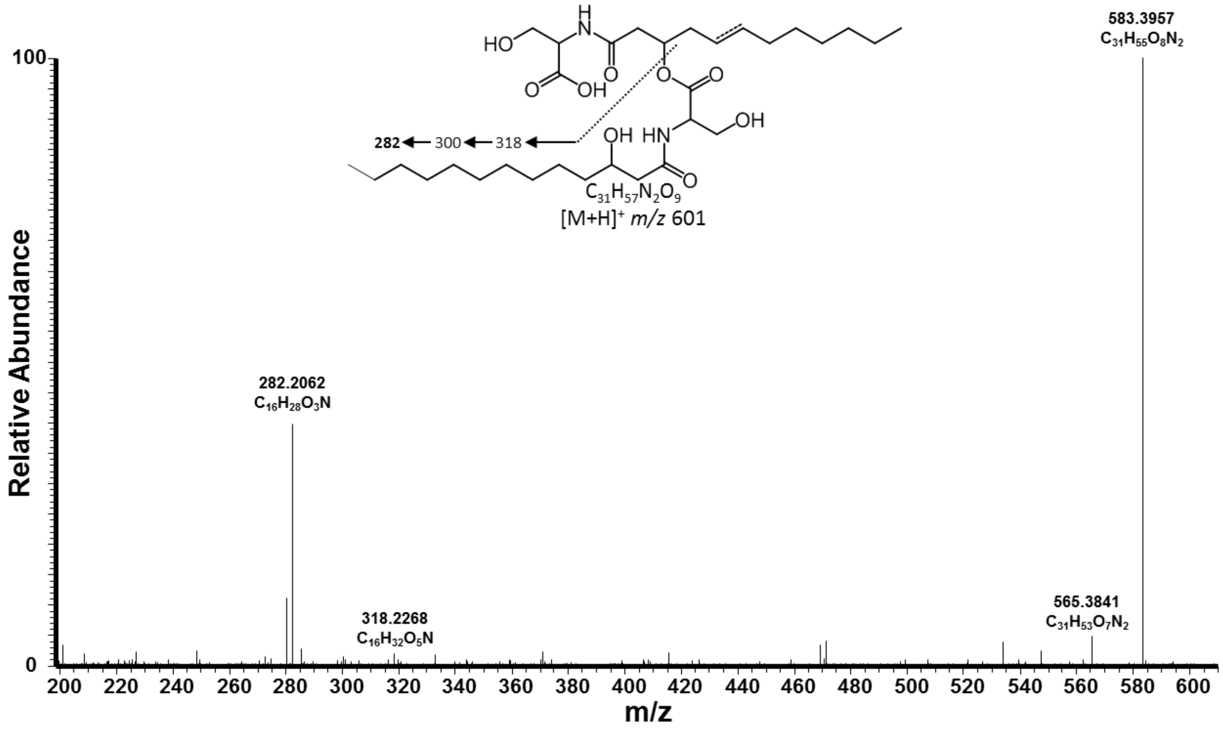

Fig. S71. HRMS[2] of serratamic acid (N-(D-3-hydroxydecanoyl)-L-serine, compound **31** $[M+H]^+$: *m/z* 276, $C_{13}H_{26}NO_5$).

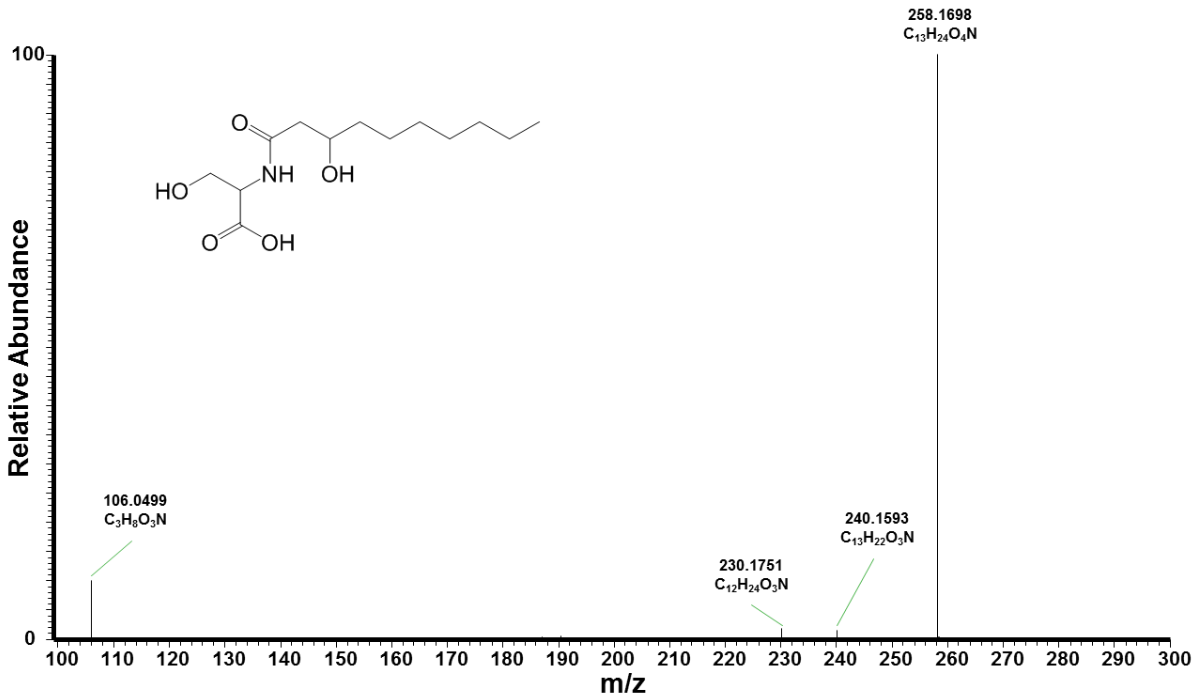

Fig. S72. HRMS[3] of serratamic acid (N-(D-3-hydroxydecanoyl)-L-serine, compound **31** $[M+H]^+$: *m/z* 276 via 258).

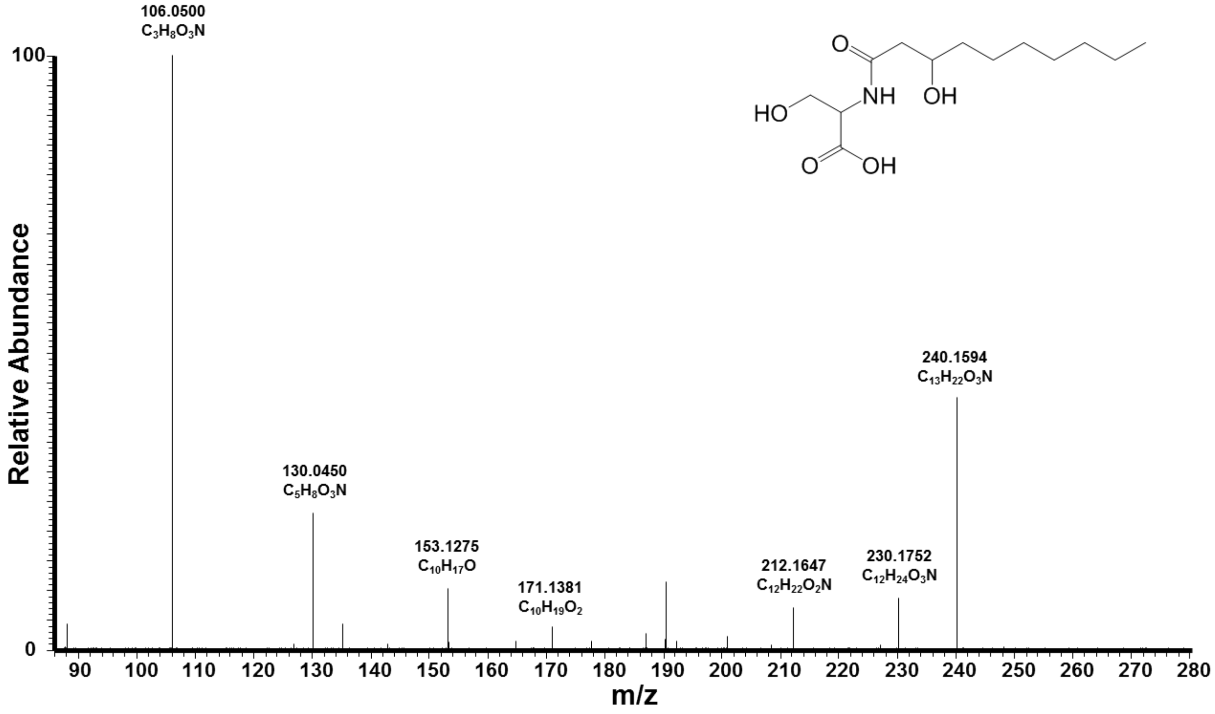

Fig. S73. Proposed mass spectral fragmentation pathway of serratamic acid (N-(D-3-hydroxydecanoyl)-L-serine, compound **31**$[M+H]^+$: *m/z* 276, $C_{13}H_{26}NO_5$).

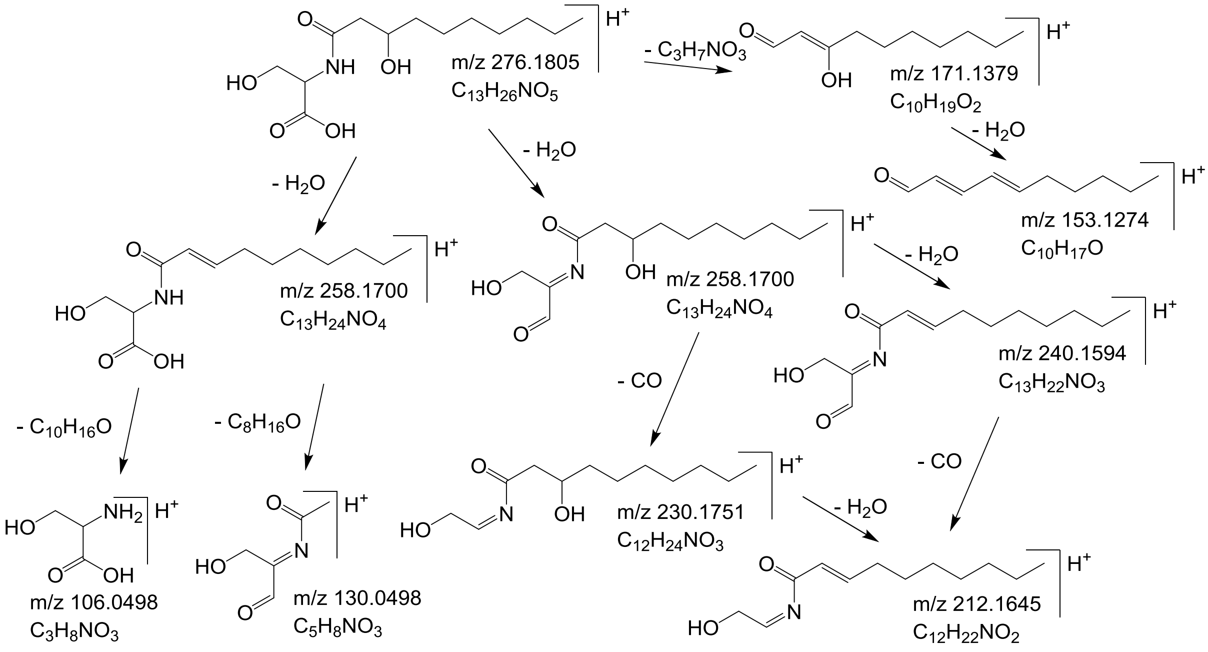

Fig. S74. HRMS[2] of serratamic acid derivative (hydroxydodecanoyl-serine, compound **32** [M+H]$^+$: *m/z* 304, $C_{15}H_{30}NO_5$).

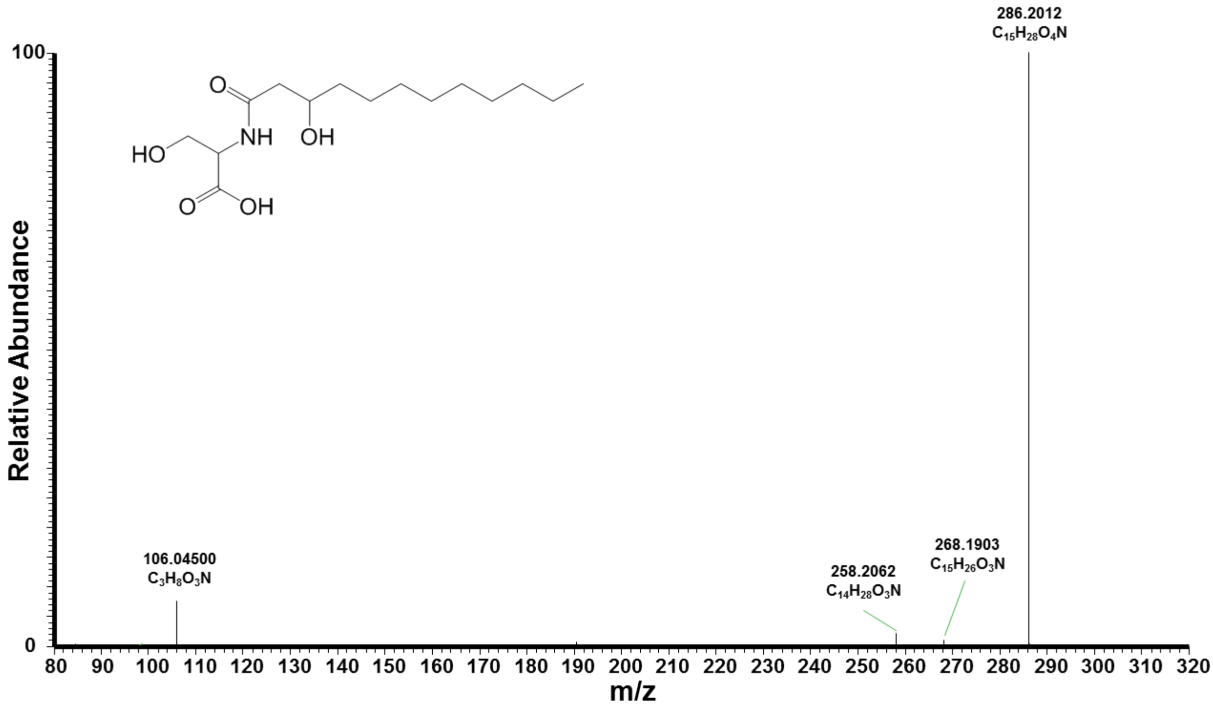

Fig. S75. HRMS[3] of serratamic acid derivative (hydroxydodecanoyl-serine, compound **32** [M+H]$^+$: *m/z* 304 via 286).

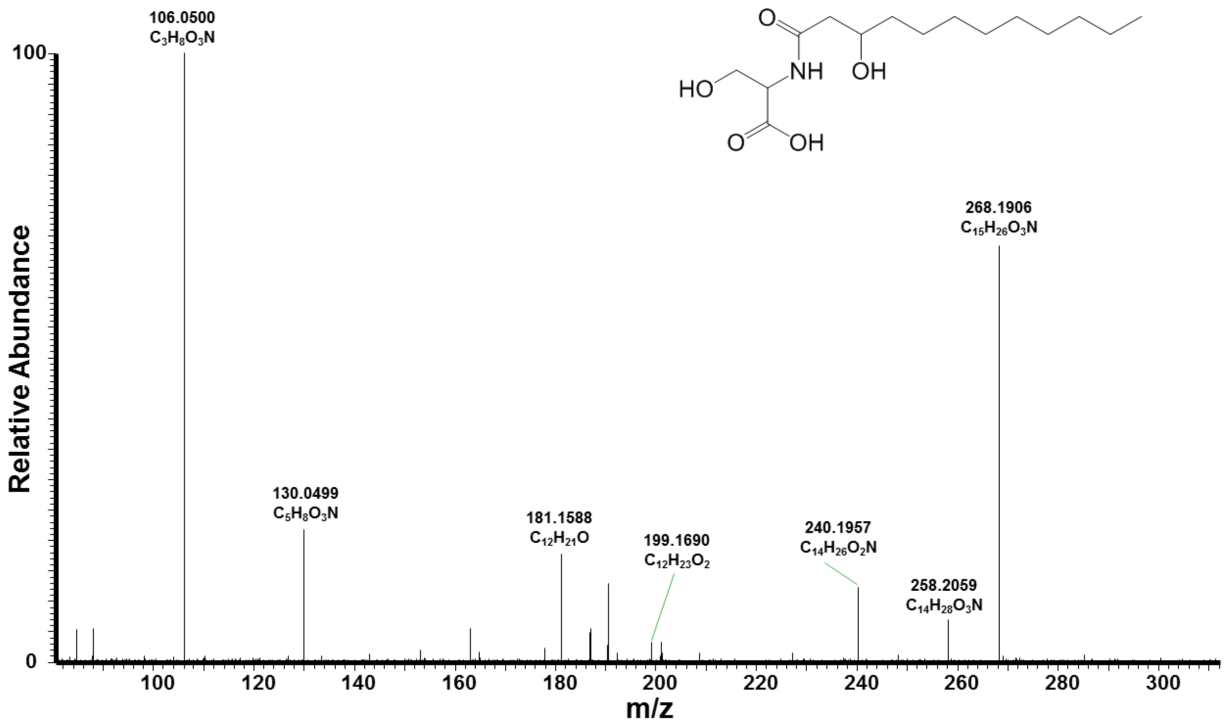

Fig. S76. Proposed mass spectral fragmentation pathway of serratamic acid derivative (hydroxydodecanoyl-serine, compound **32** $[M+H]^+$: *m/z* 304, $C_{15}H_{30}NO_5$).

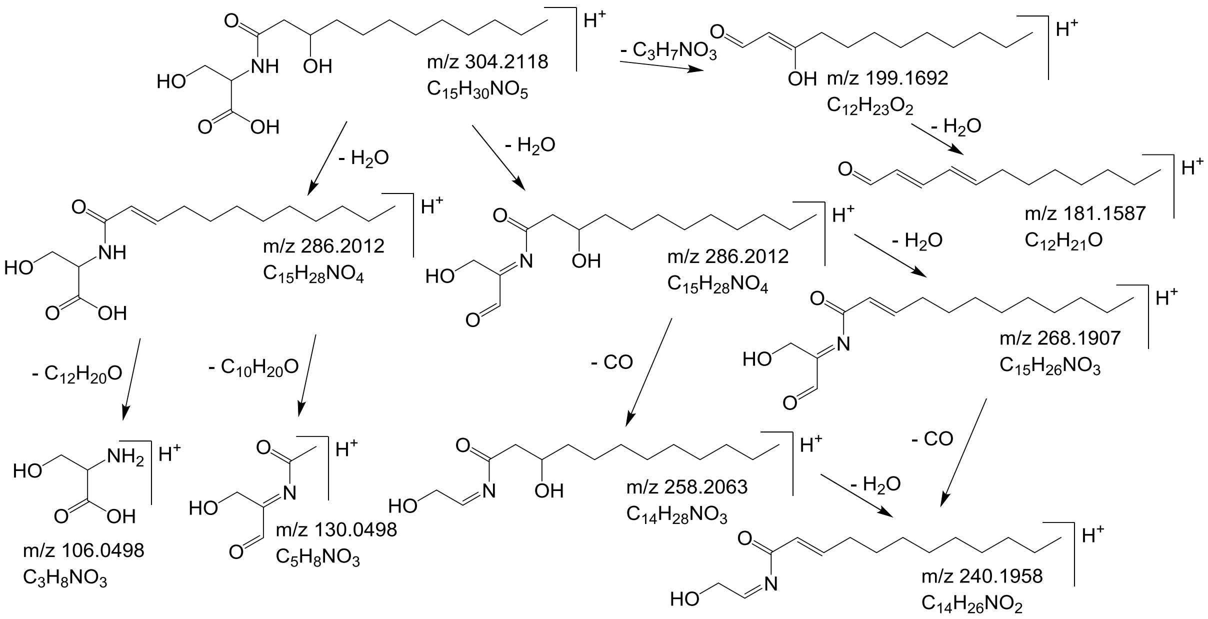

Fig. S77. HRMS^2 of serratamic acid derivative with double bond (hydroxydodecenoyl-serine, compound **33** $[M+H]^+$: *m/z* 302, $C_{15}H_{28}NO_5$).

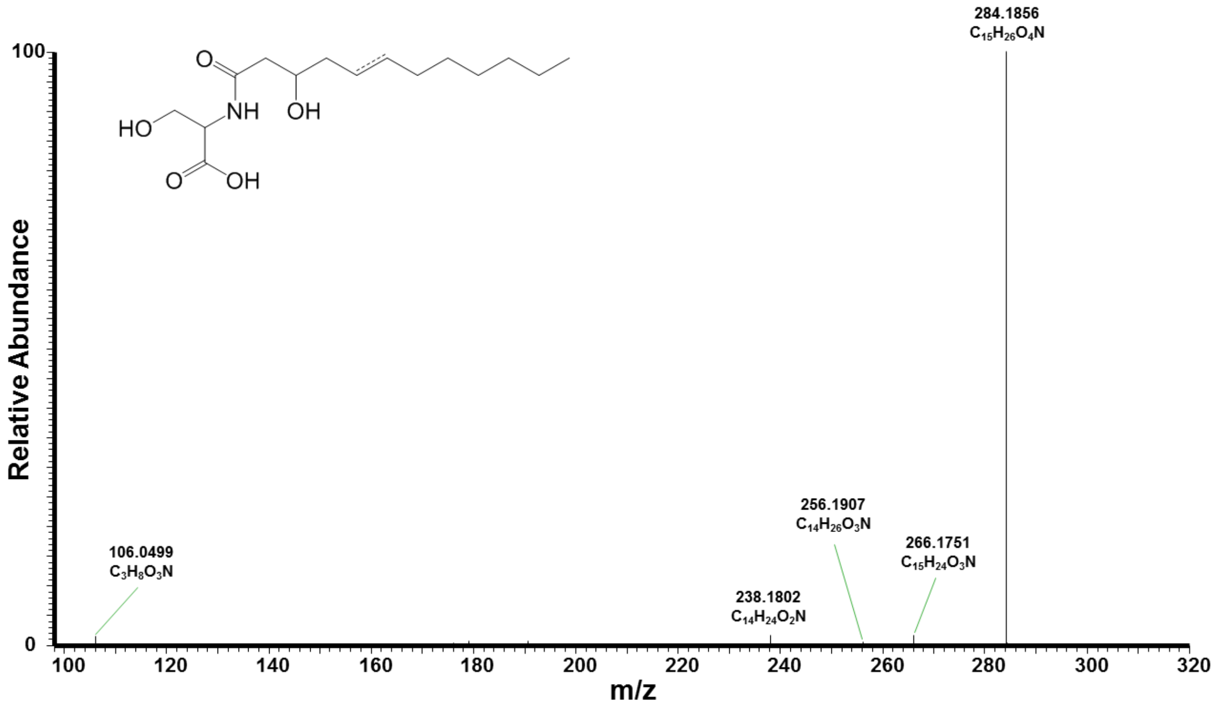

Fig. S78. HRMS^3 of of serratamic acid derivative with double bond (hydroxydodecenoyl-serine, compound **33** $[M+H]^+$: *m/z* 302 via 284).

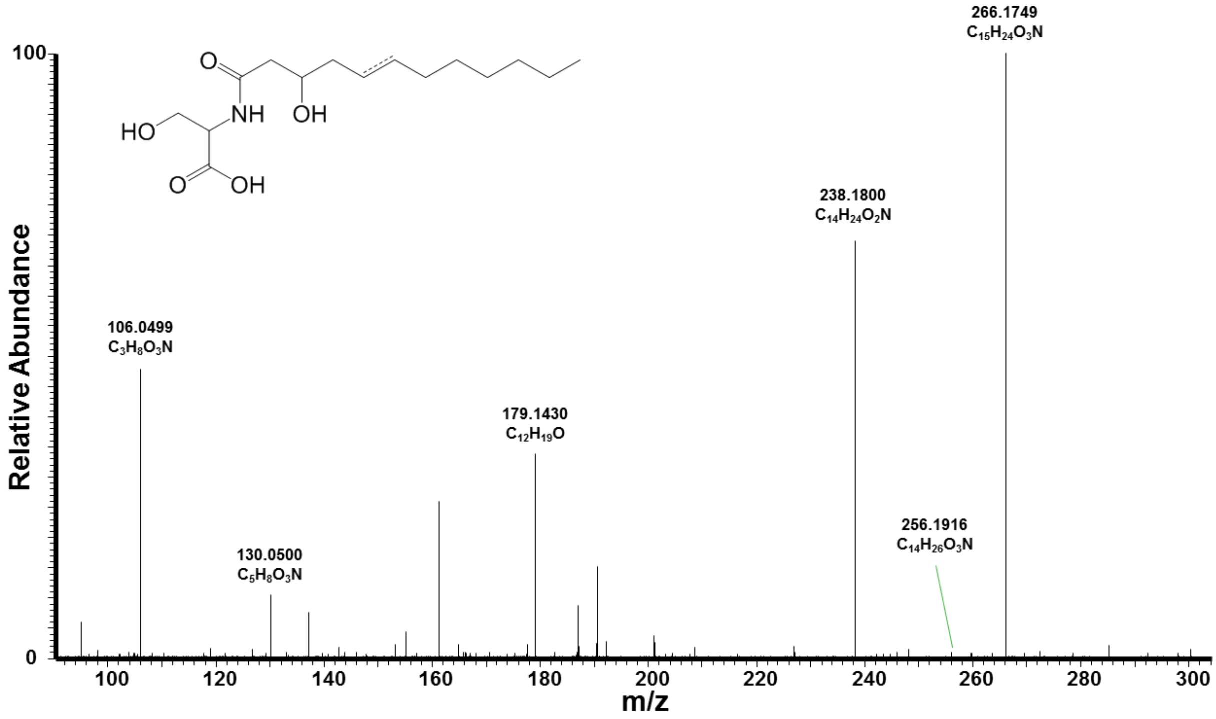

Fig. S79. Proposed mass spectral fragmentation pathway of serratamic acid derivative with double bond (hydroxydodecenoyl-serine, compound **33** $[M+H]^+$: *m/z* 302, $C_{15}H_{28}NO_5$).

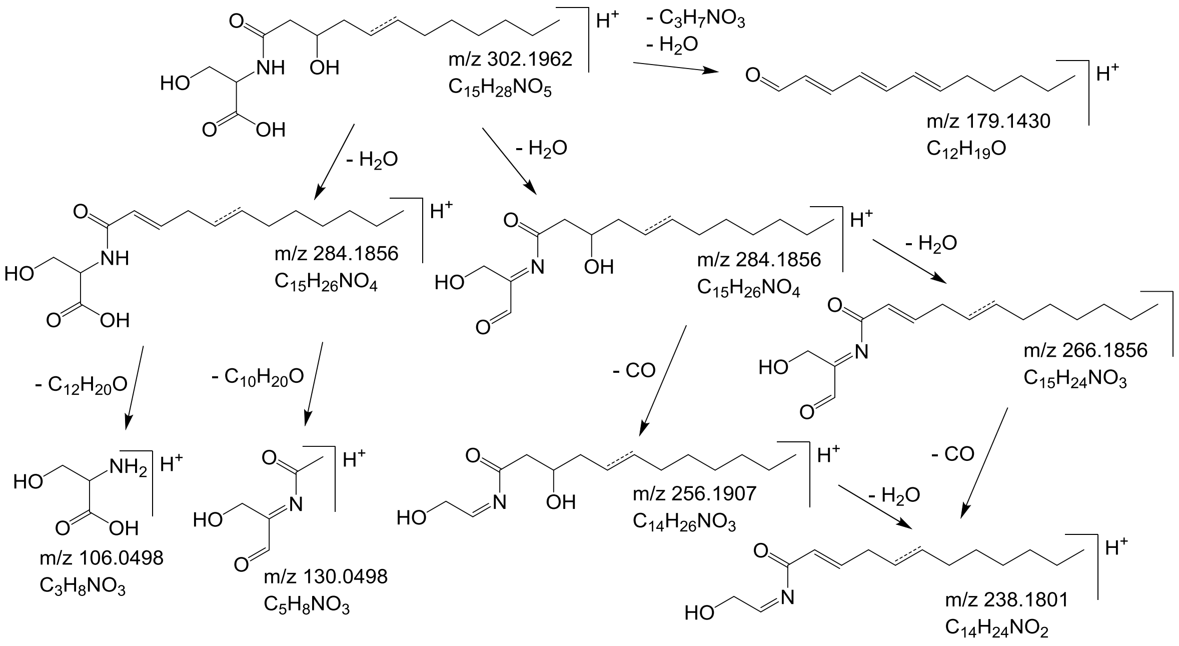

Fig. S80. ^{1}H-NMR spectrum of serrawettin W1 / serratamolide C_{10}+C_{10} (compound **10**, $CDCl_3$, 600 MHz).

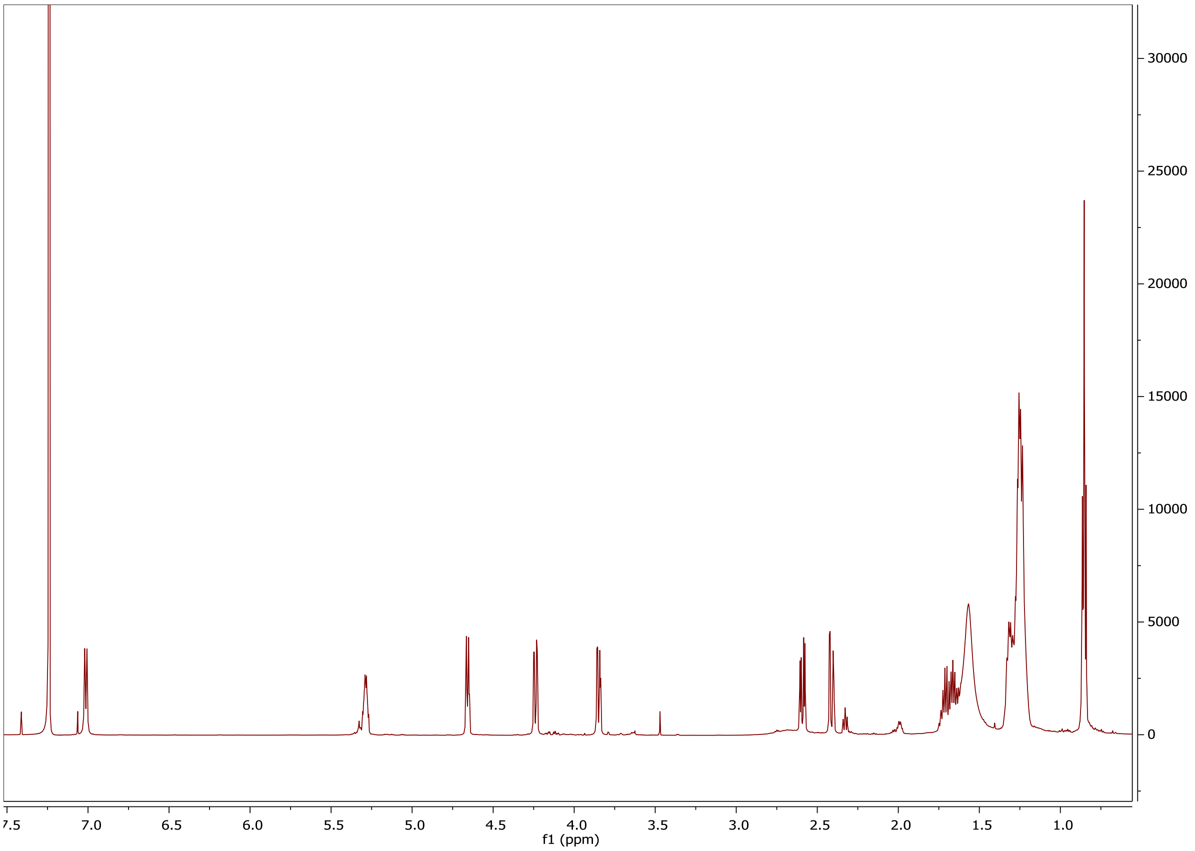

Fig. S81. ^{13}C-NMR spectrum of serrawettin W1 / serratamolide C_{10}+C_{10} (compound **10**, $CDCl_3$, 150 MHz).

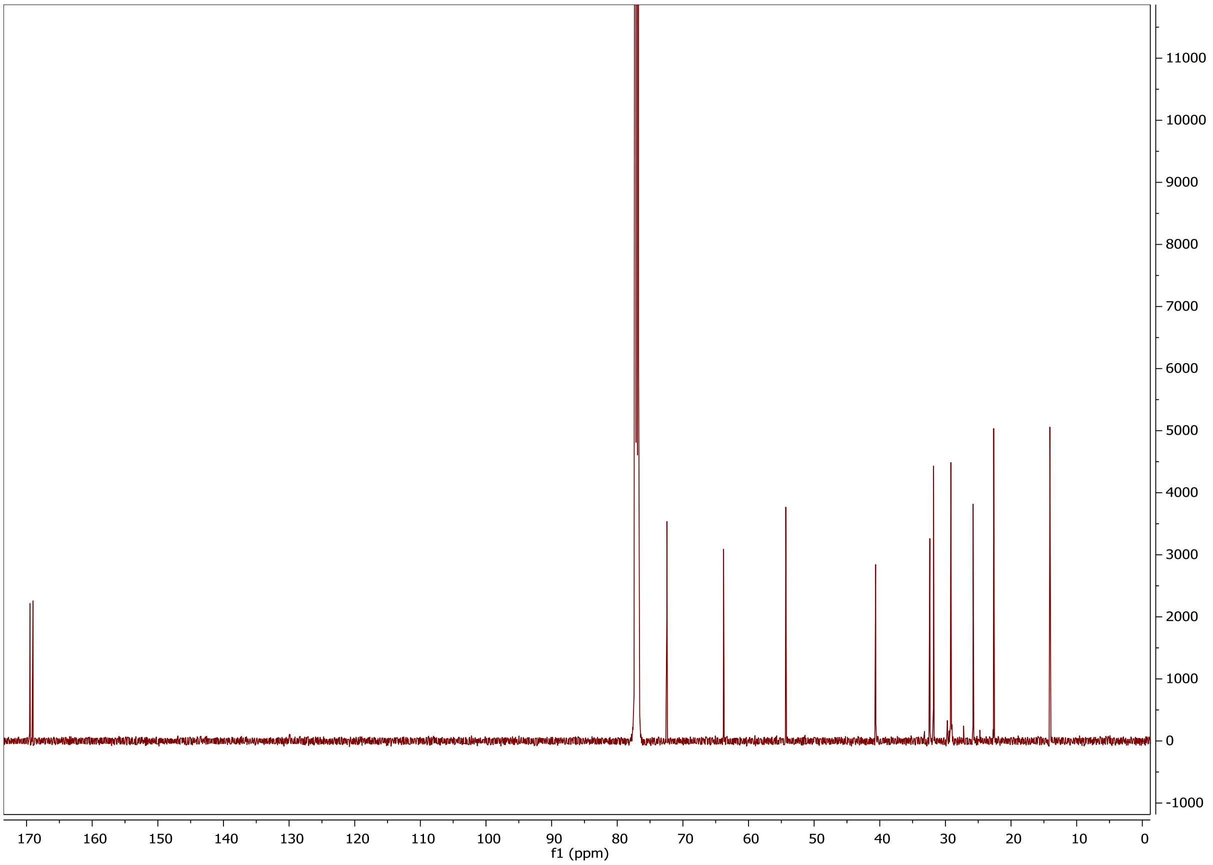

Fig. S82. COSY NMR spectrum of serrawettin W1 / serratamolide C_{10}+C_{10} (compound **10**, $CDCl_3$).

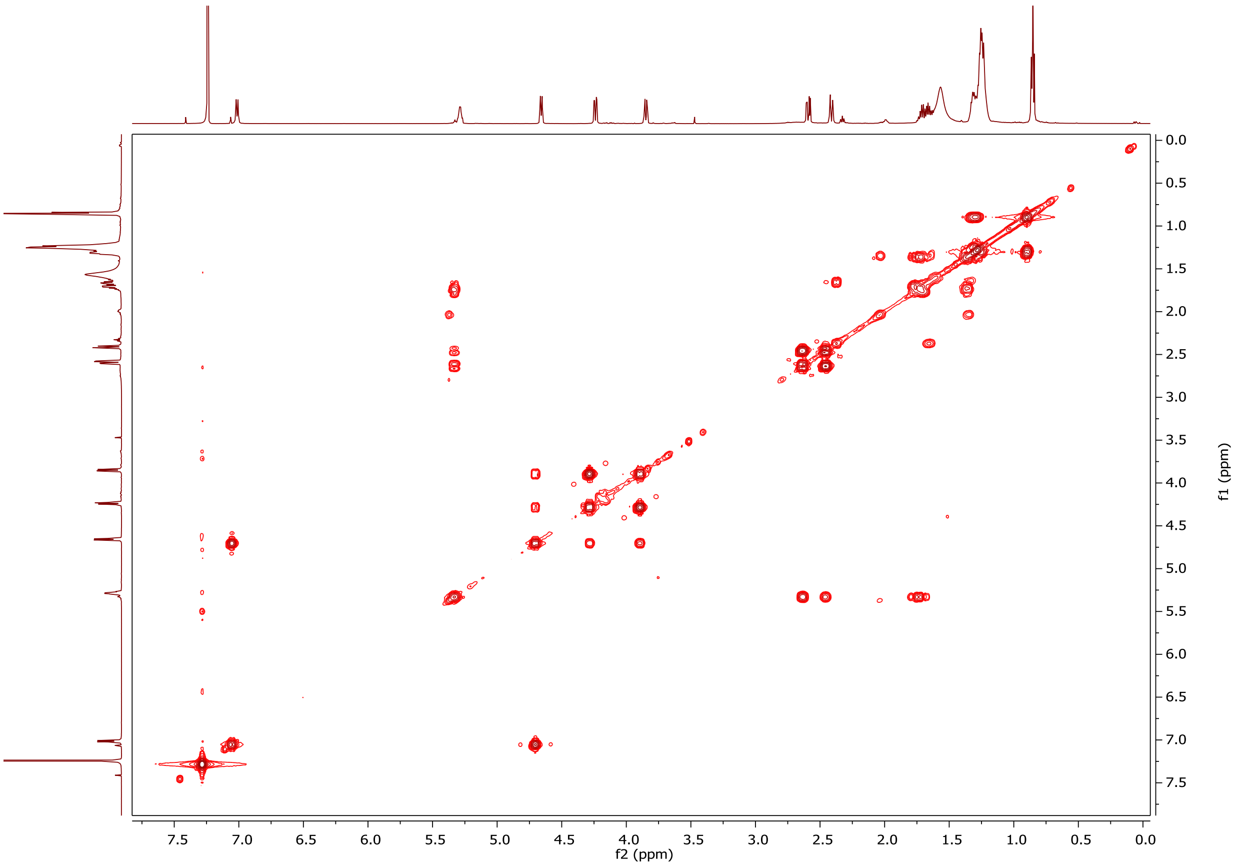

Fig. S83. HSQC NMR spectrum of serrawettin W1 / serratamolide C_{10}+C_{10} (compound **10**, $CDCl_3$).

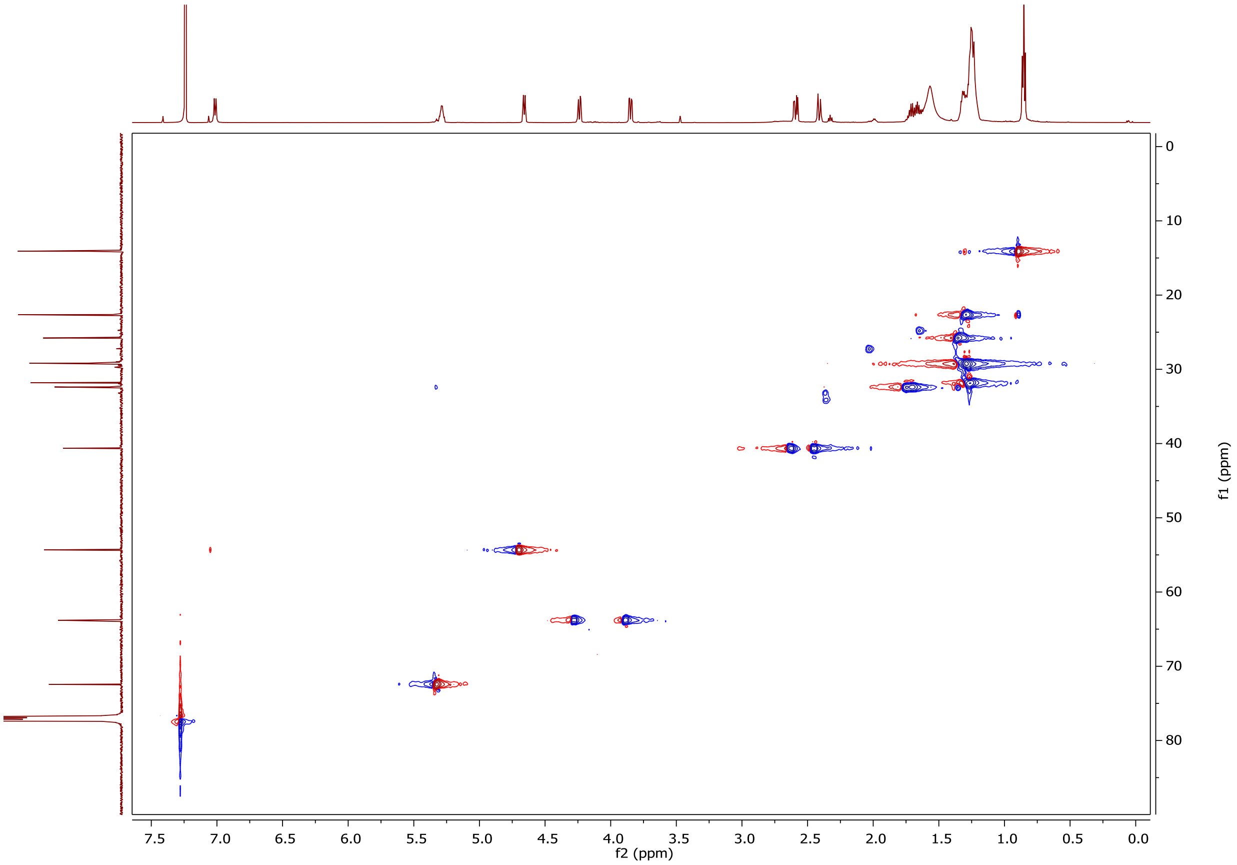

Fig. S84. HMBC NMR spectrum of serrawettin W1 / serratamolide C_{10}+C_{10} (compound **10**, $CDCl_3$).

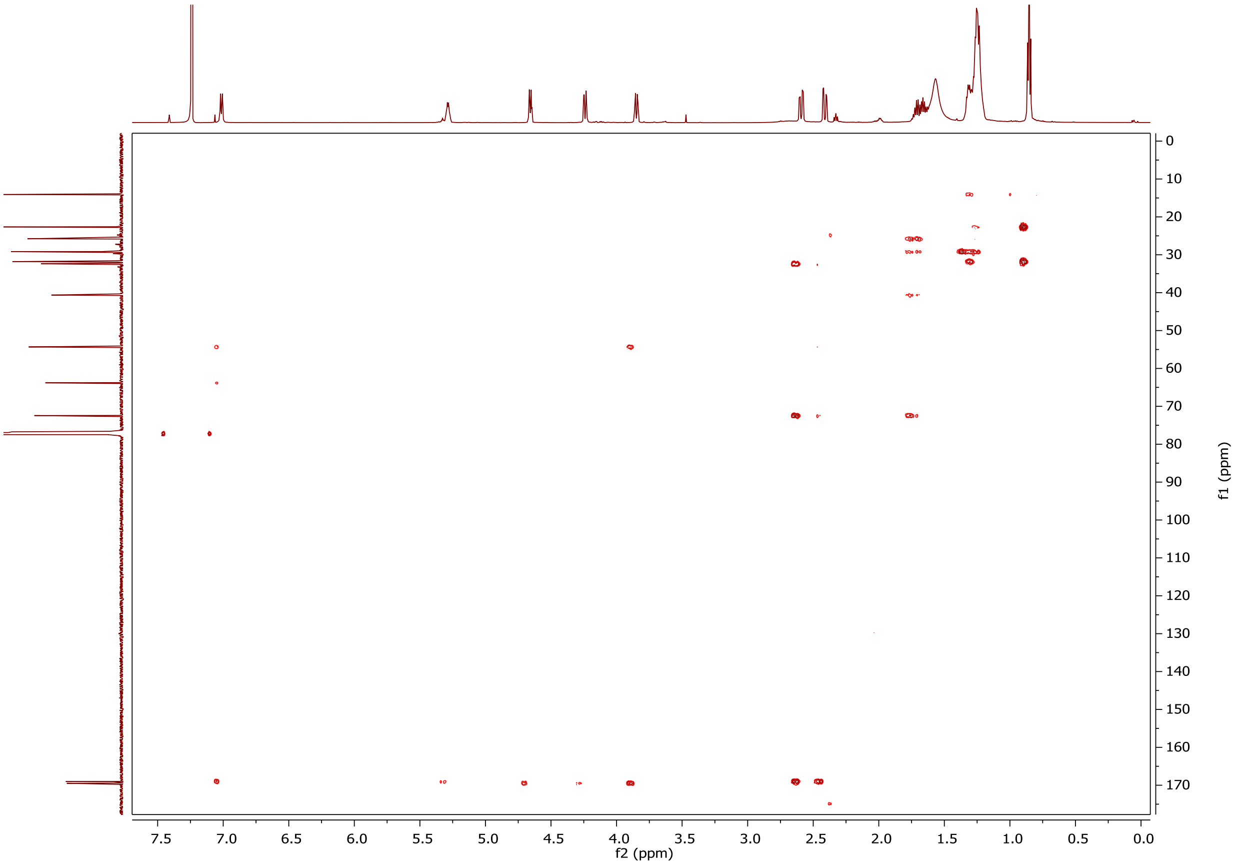

Fig. S85. ^{1}H-NMR spectrum of serratamic acid (N-(D-3-hydroxydecanoyl)-L-serine, compound **31**, MeOD, 700 MHz).

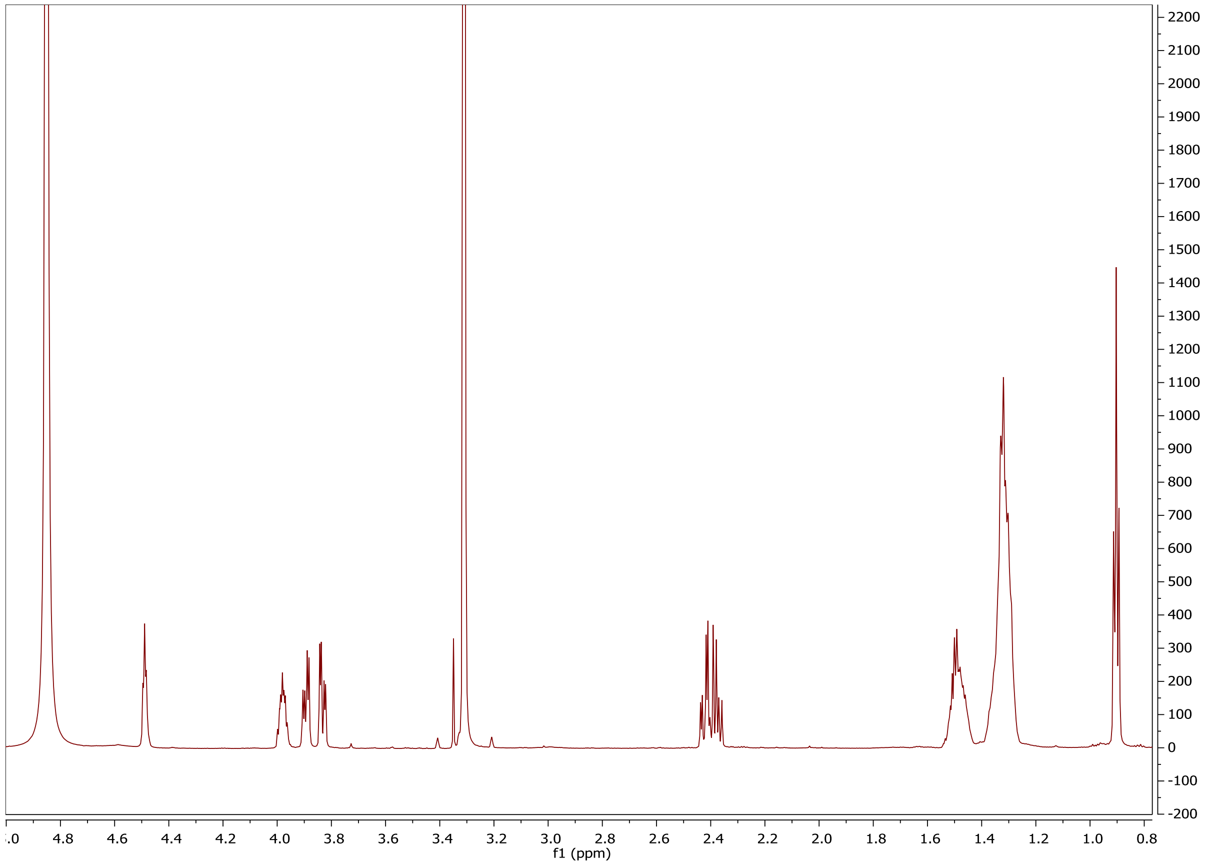

Fig. S86. ^{13}C-NMR spectrum of serratamic acid (N-(D-3-hydroxydecanoyl)-L-serine, compound **31**, MeOD, 175 MHz).

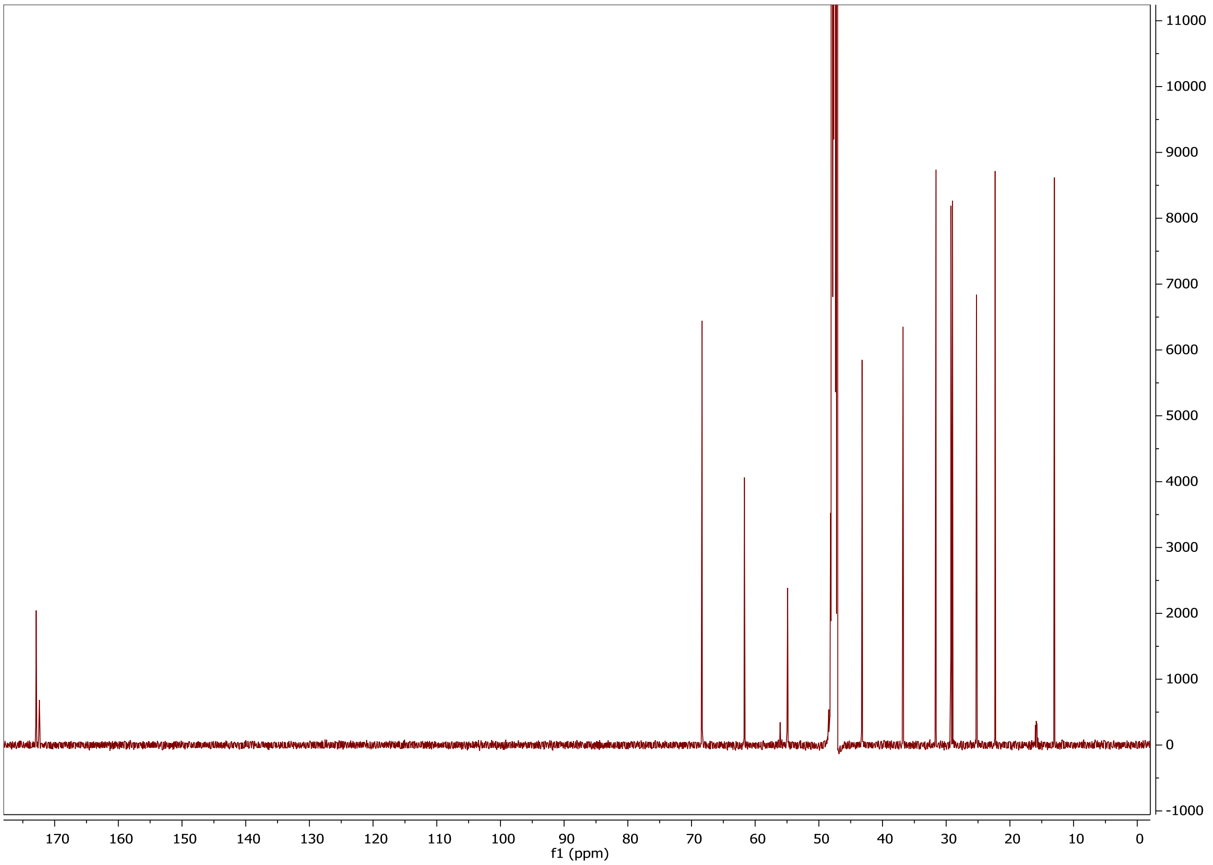

Fig. S87. COSY NMR spectrum of serratamic acid (N-(D-3-hydroxydecanoyl)-L-serine, compound **31**, MeOD).

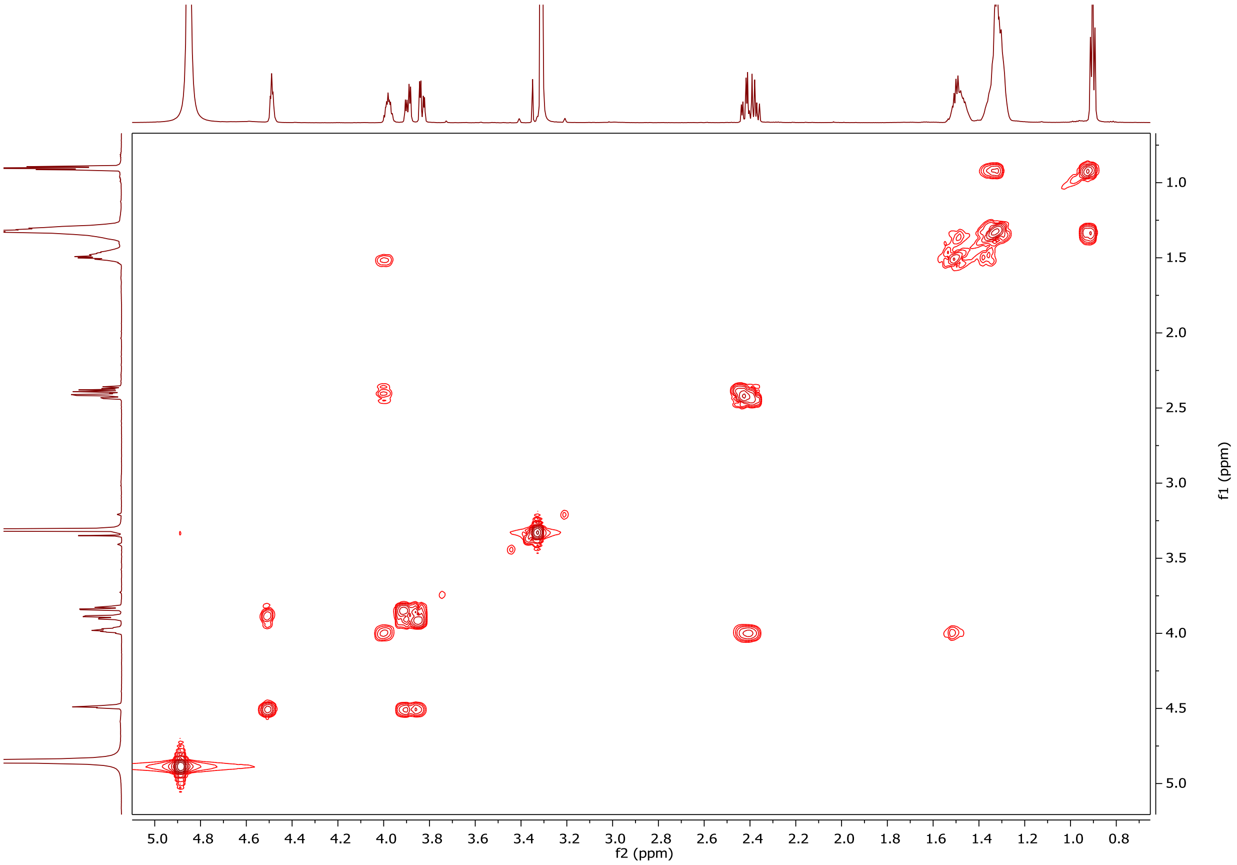

Fig. S88. HSQC NMR spectrum of serratamic acid (N-(D-3-hydroxydecanoyl)-L-serine, compound **31**, MeOD).

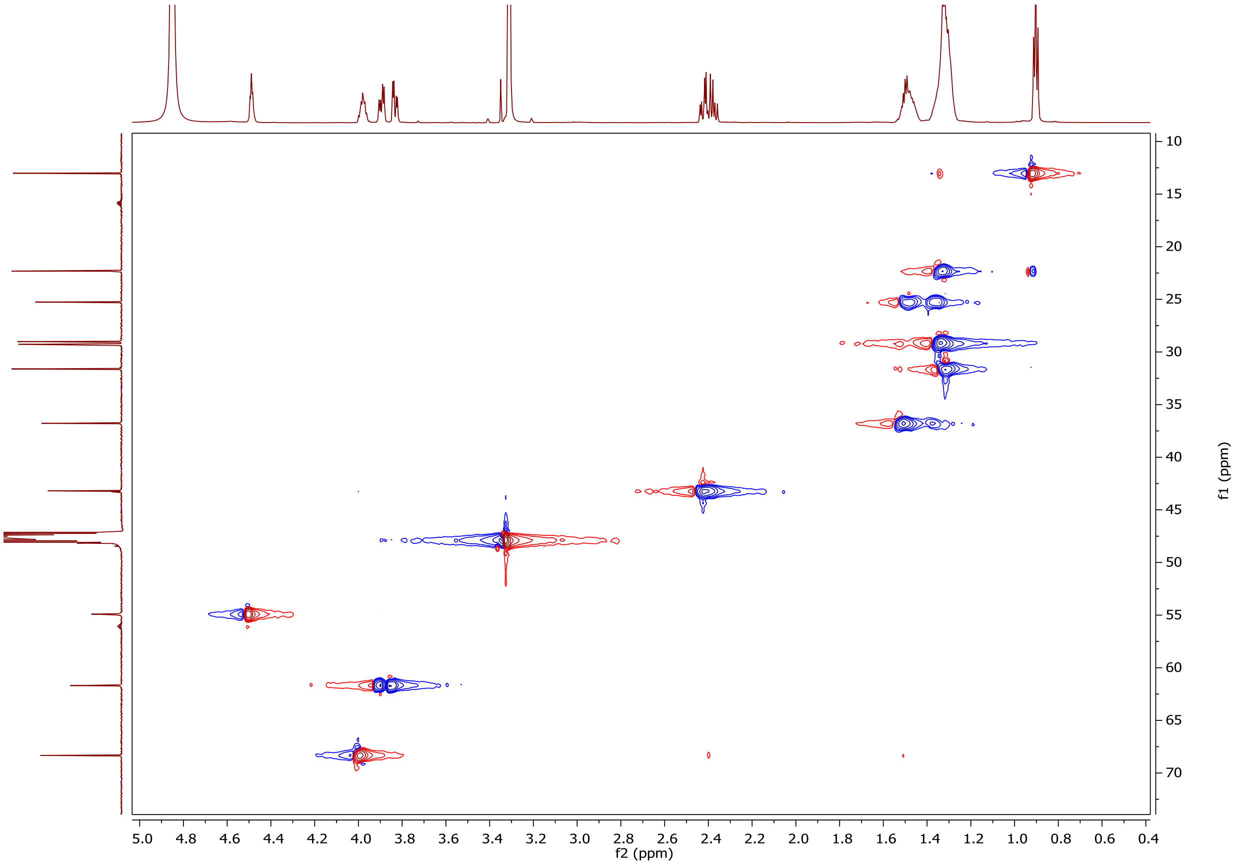

Fig. S89. HMBC NMR spectrum of serratamic acid (N-(D-3-hydroxydecanoyl)-L-serine, compound **31**, MeOD).

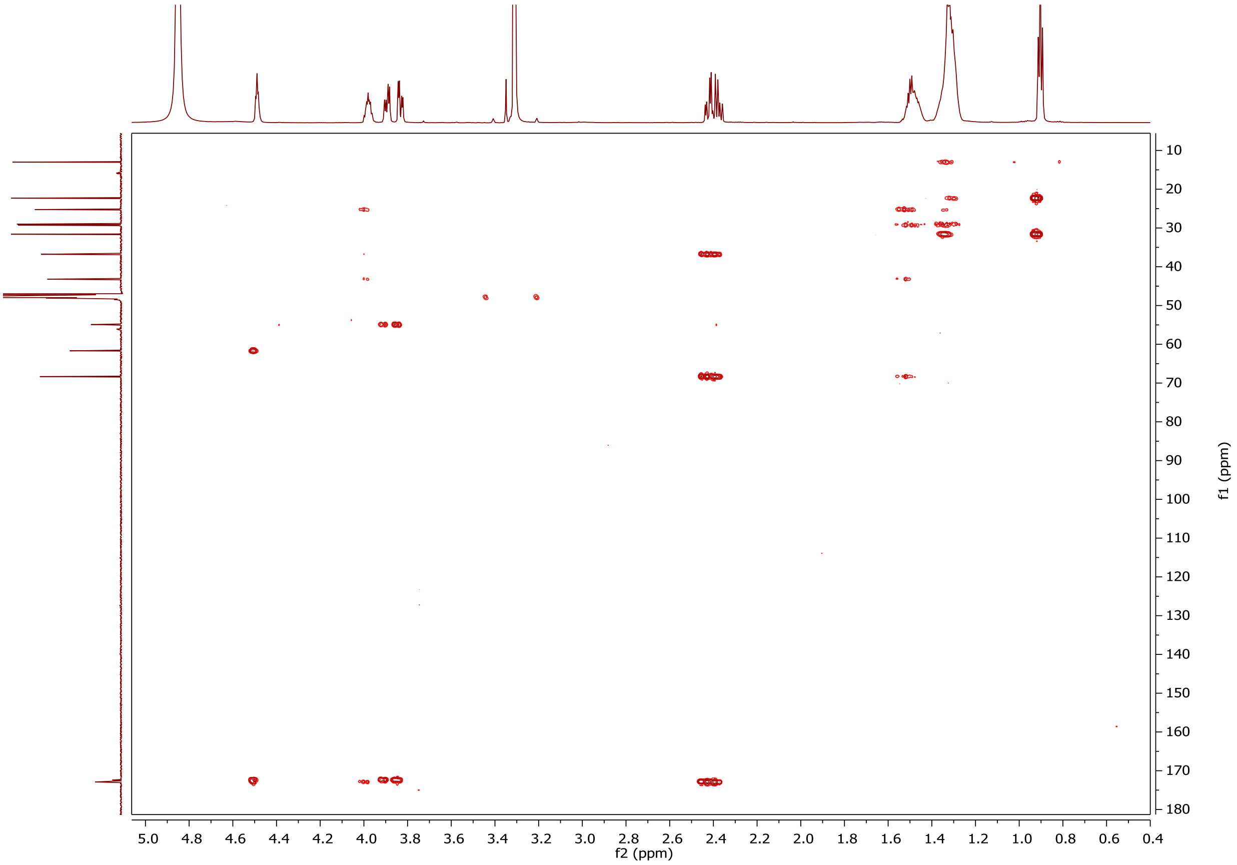

Fig. S90. ^{1}H-NMR spectrum of prodigiosin (compound **3**, $CDCl_3$, 600 MHz).

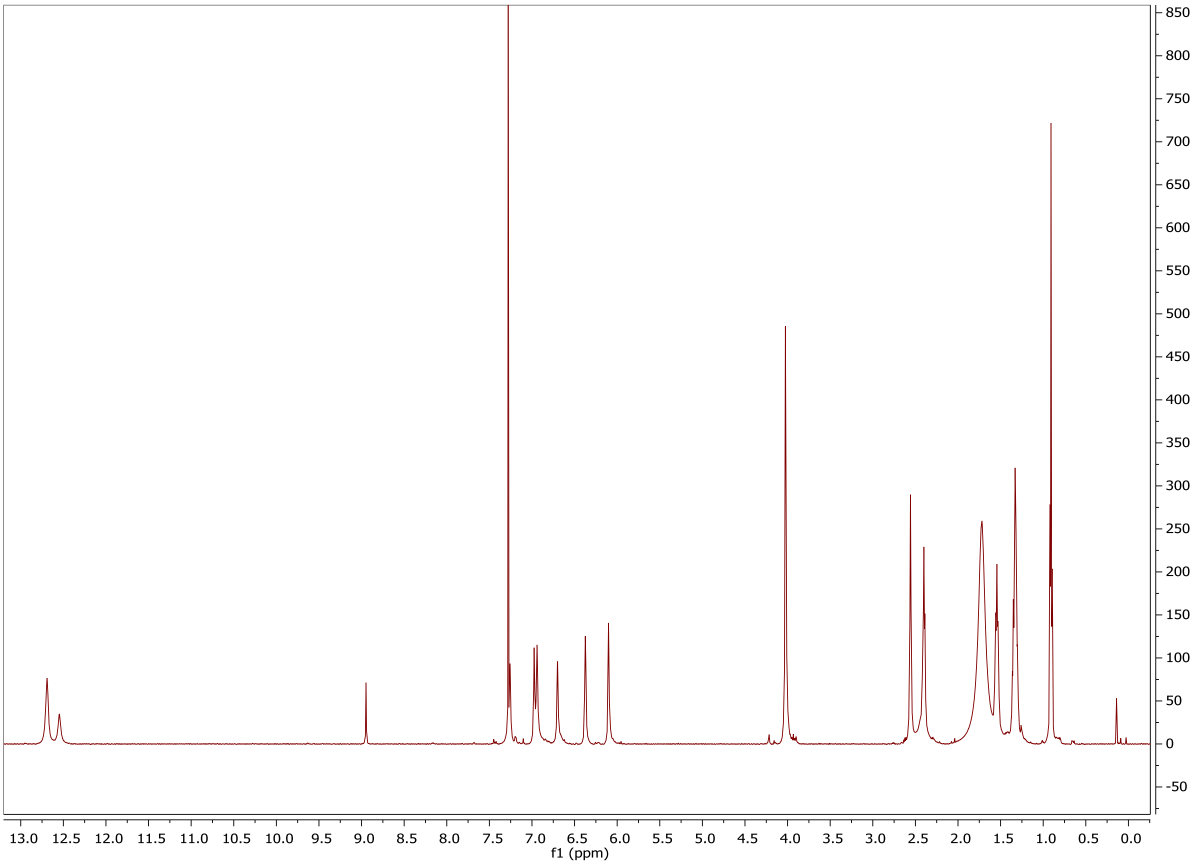

Fig. S91. COSY NMR spectrum of prodigiosin (compound **3**, $CDCl_3$, 600 MHz).

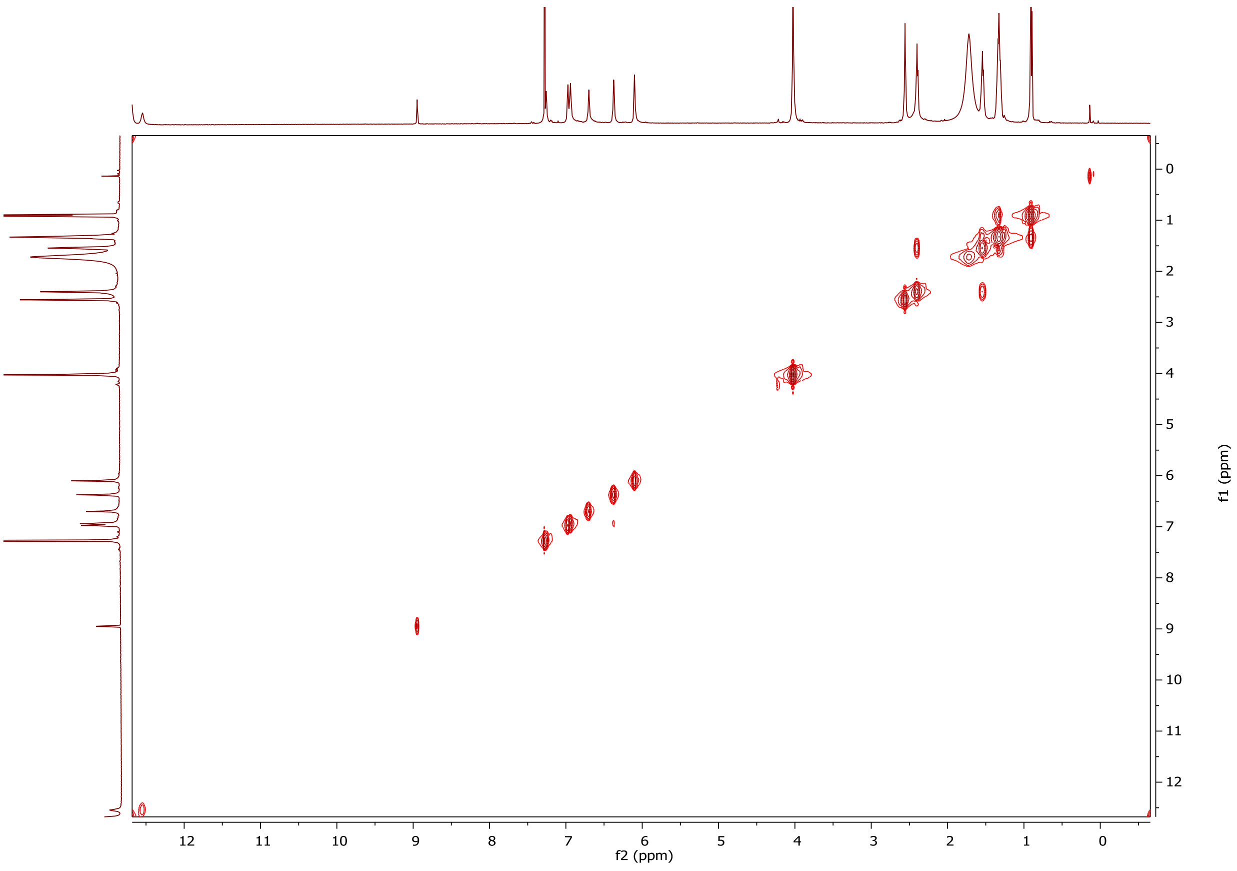

Fig. S92. HRMS spectra of 2-methyl-3-propyl prodiginine (compound **1** $[M+H]^+$: *m/z* 296, $C_{18}H_{22}N_3O$).

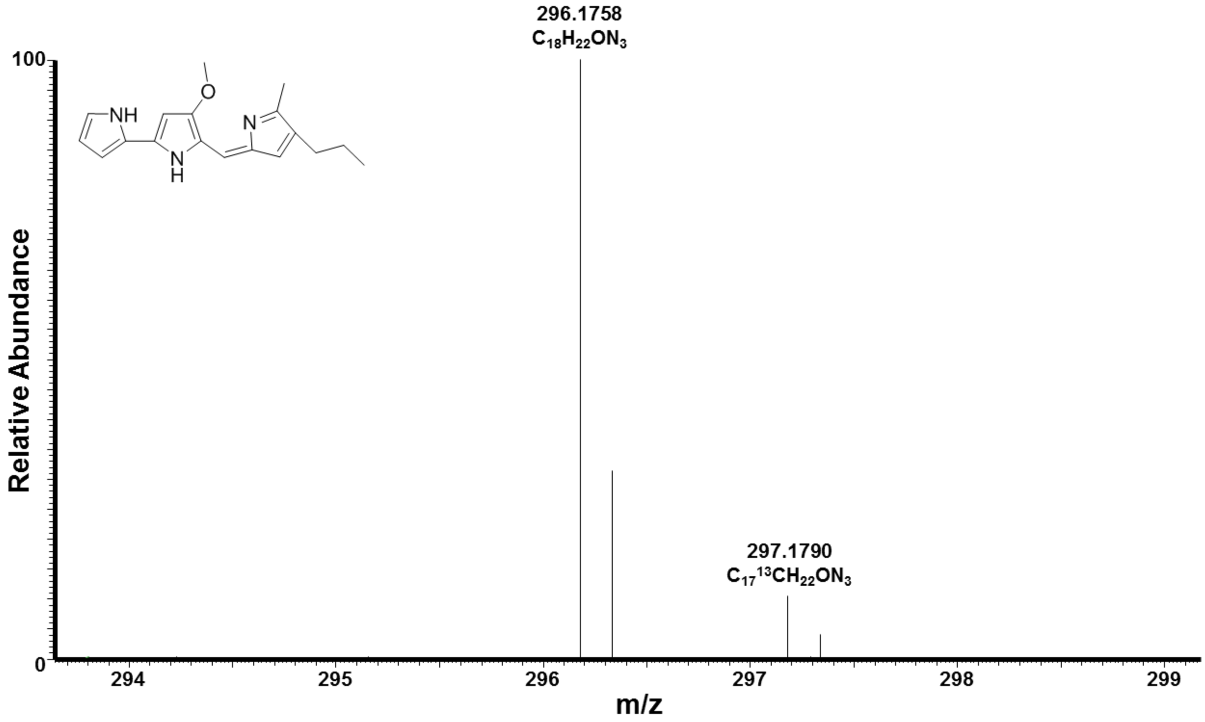

Fig. S93. HRMS spectra of 2-methyl-3-butyl prodiginine (compound **2** $[M+H]^+$: *m/z* 310, $C_{19}H_{24}N_3O$).

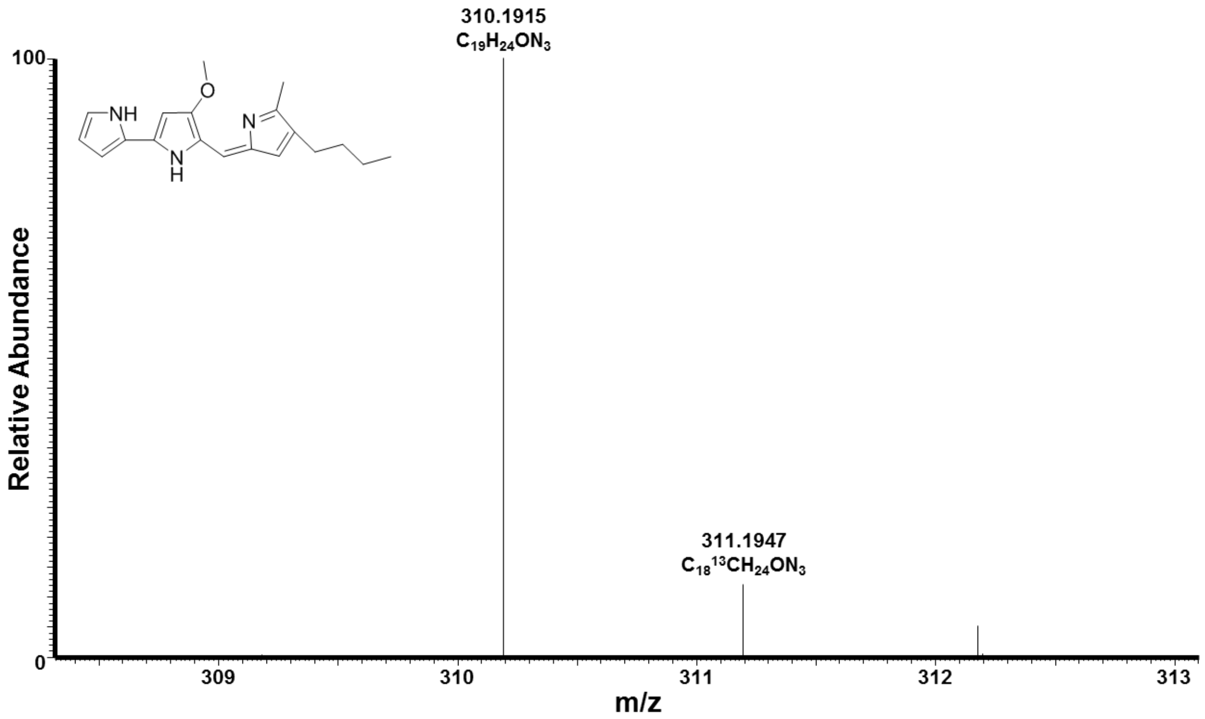

Fig. S94. HRMS spectra of prodigiosin (compound **3** $[M+H]^+$: *m/z* 324, $C_{20}H_{26}N_3O$).

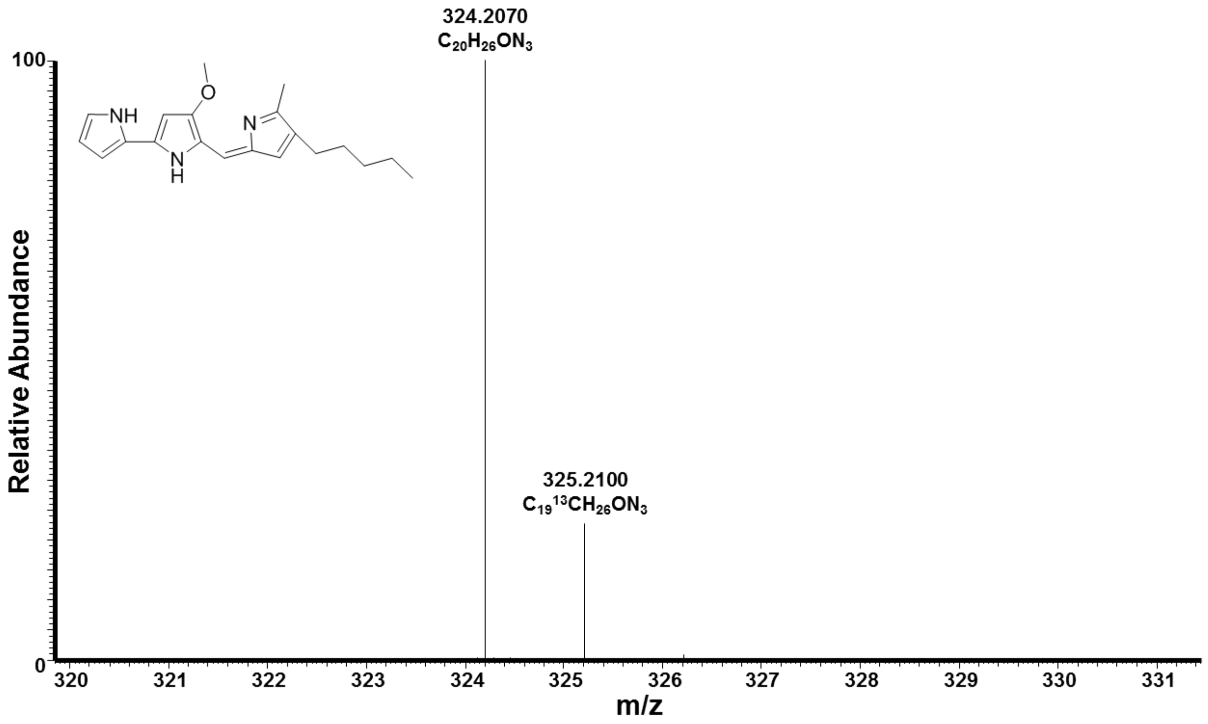

Fig. S95. HRMS spectra of 2-methyl-3-hexyl prodiginine (compound **4** $[M+H]^+$: *m/z* 338, $C_{21}H_{28}N_3O$).

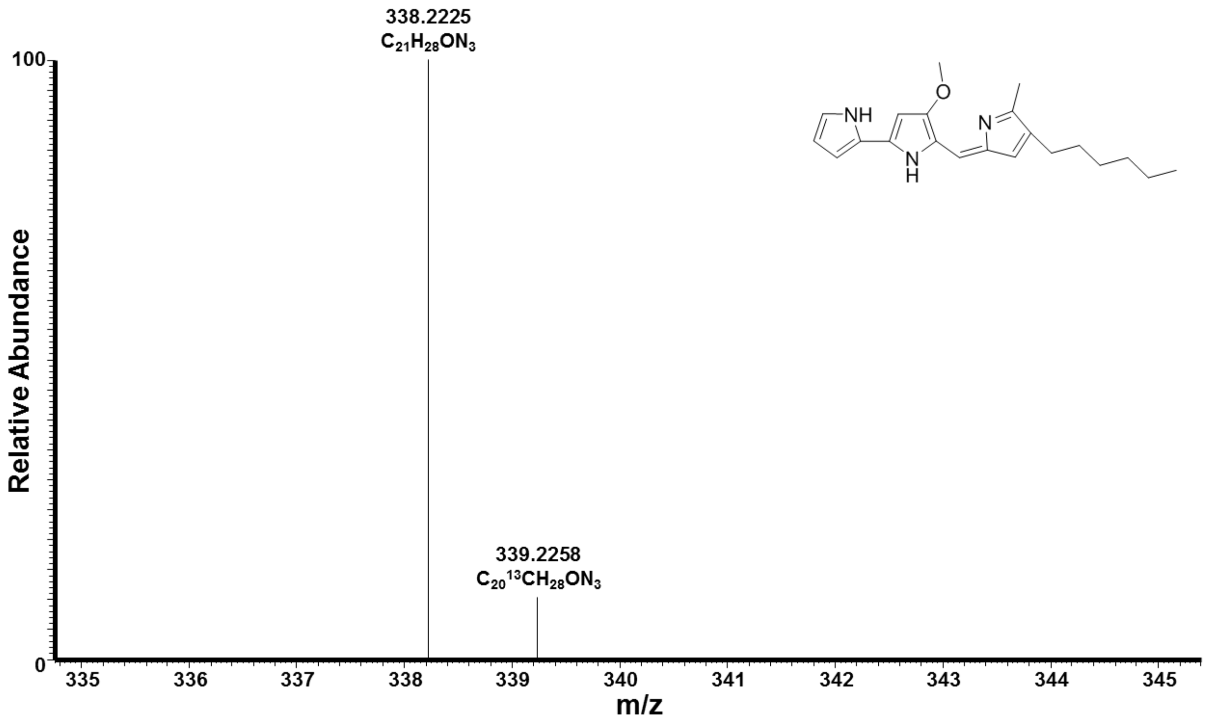

Fig. S96. HRMS spectra of 2-methyl-3-heptyl prodiginine (compound **5** $[M+H]^+$: *m/z* 352, $C_{22}H_{30}N_3O$).

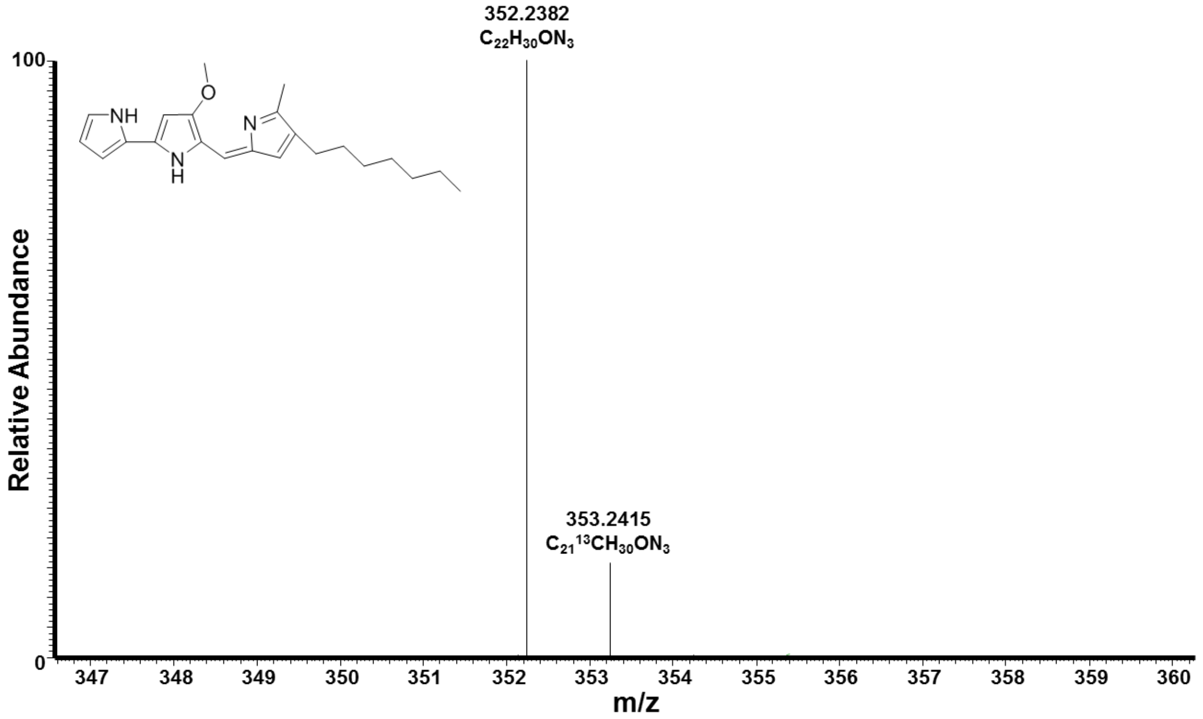

Fig. S97. HRMS spectra of 2-methyl-3-octyl prodiginine (compound **6** $[M+H]^+$: *m/z* 366, $C_{23}H_{32}N_3O$).

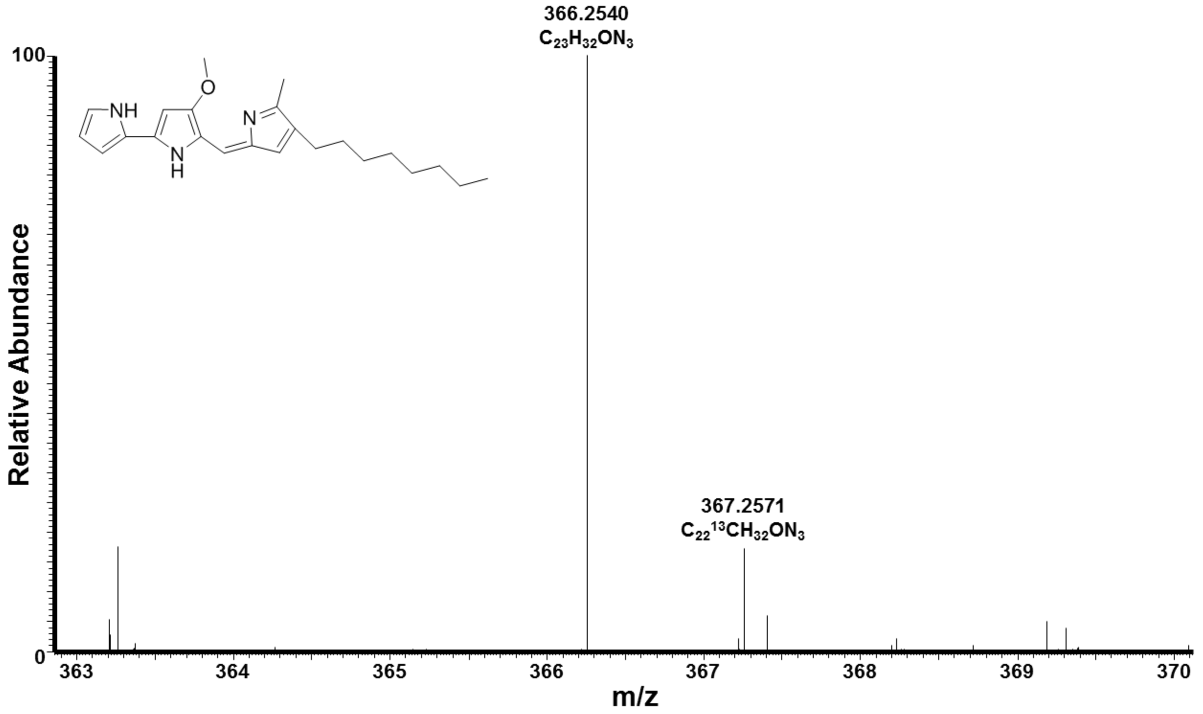

Fig. S98. HRMS spectra of 2-methyl-3-nonyl prodiginine (compound **7** $[M+H]^+$: *m/z* 380, $C_{24}H_{34}N_3O$).

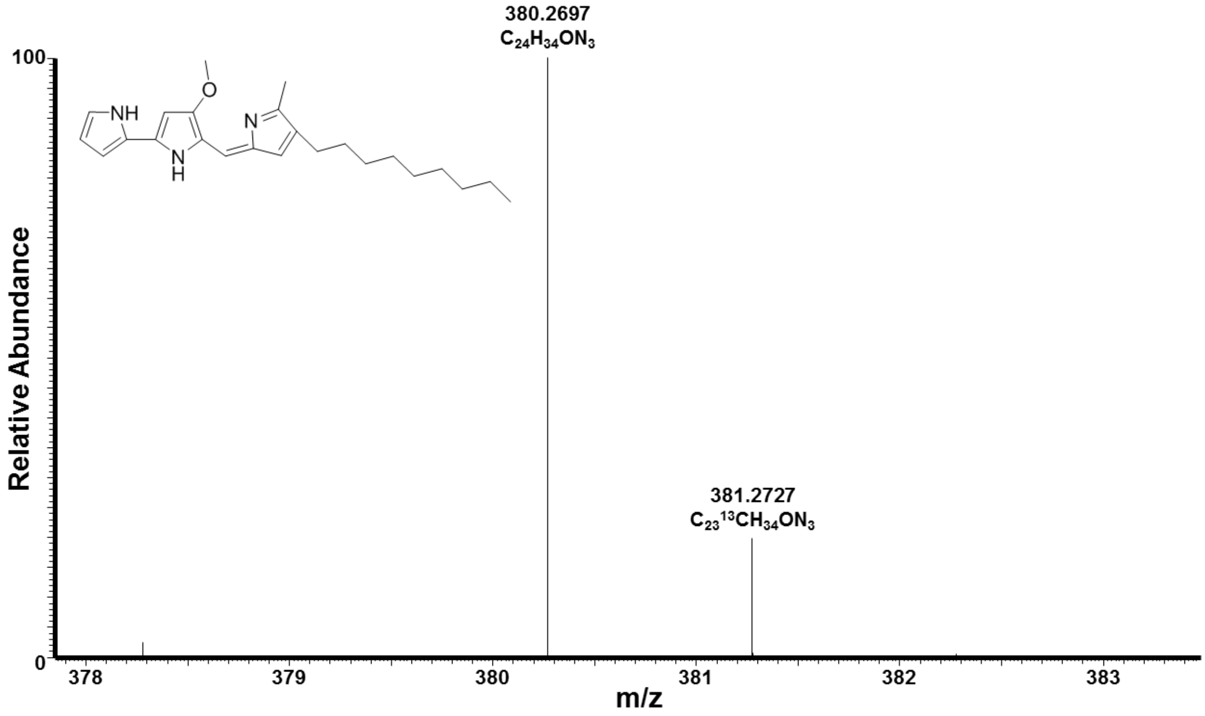

Fig. S99. HRMS spectra of 2-methyl-3-propyl prodiginine with incorporated ^{15}N derived from $^{15}NH_4Cl$ (compound **1** $[M+H]^+$: *m/z* 297, $C_{18}H_{22}N_2{}^{15}NO$).

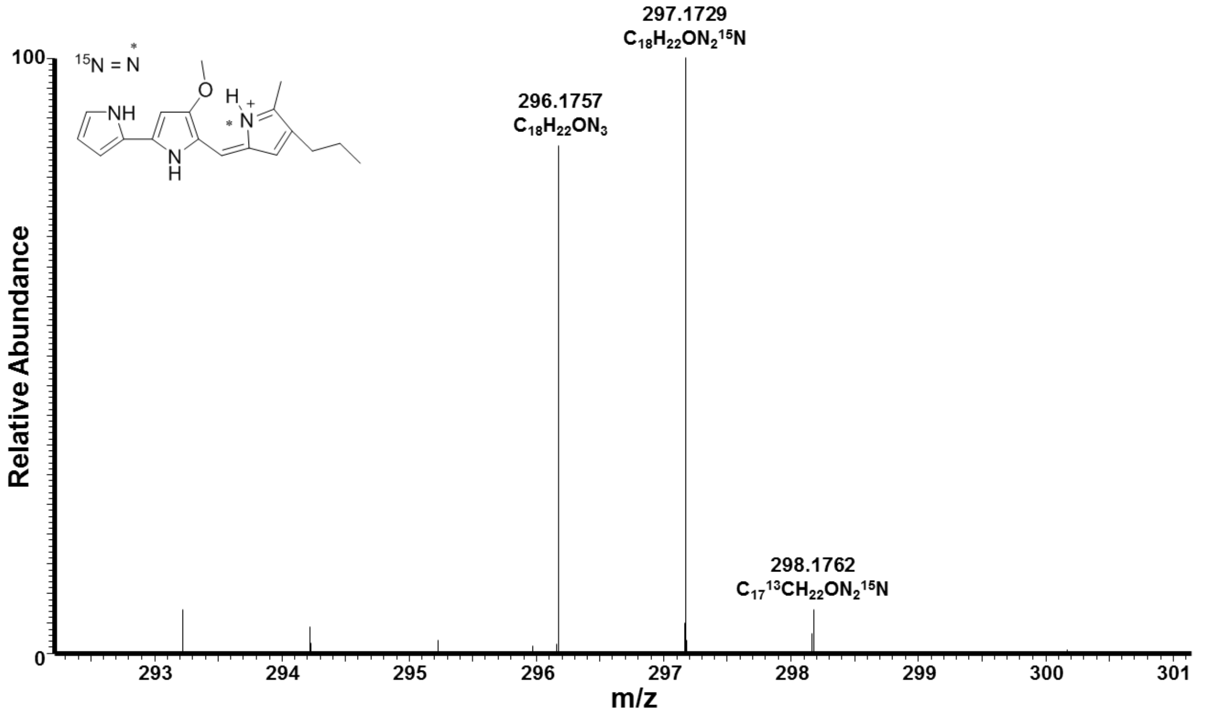

Fig. S100. HRMS spectra of 2-methyl-3-butyl prodiginine with incorporated ^{15}N derived from $^{15}NH_4Cl$ (compound **2** $[M+H]^+$: *m/z* 311, $C_{19}H_{24}N_2{}^{15}NO$).

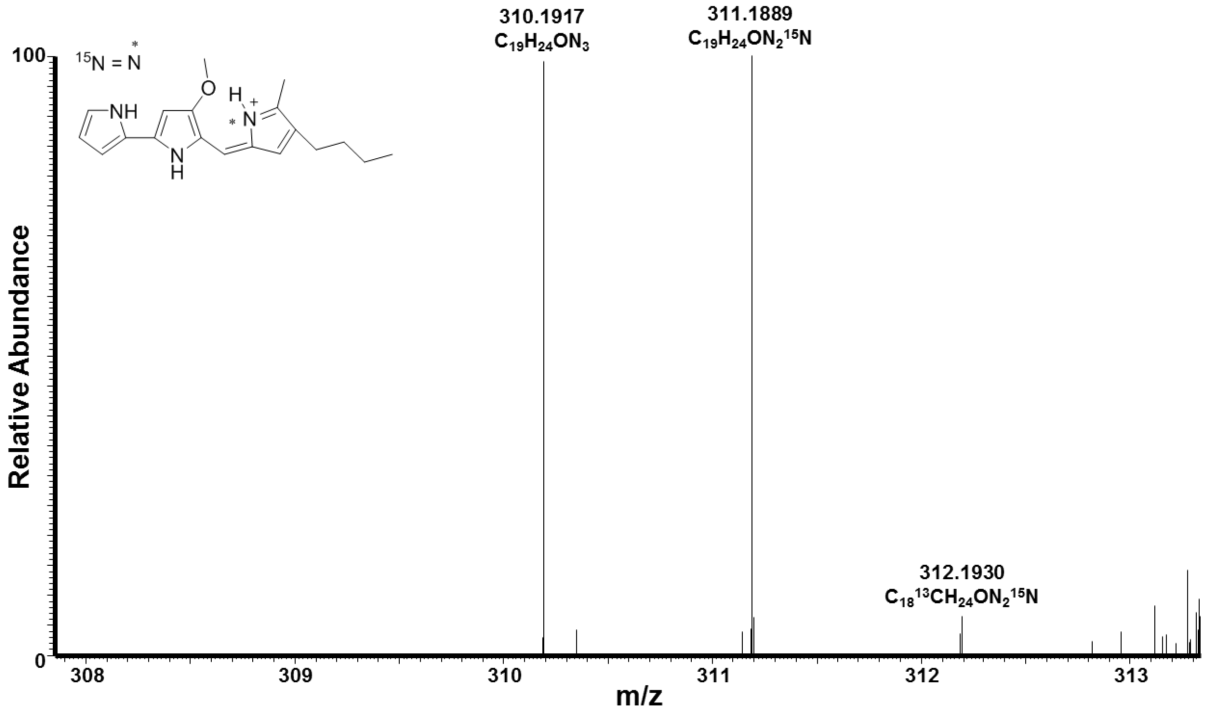

Fig. S101. HRMS spectra of prodigiosin with incorporated ^{15}N derived from $^{15}NH_4Cl$ (compound **3** $[M+H]^+$: *m/z* 325, $C_{20}H_{26}N_2{}^{15}NO$).

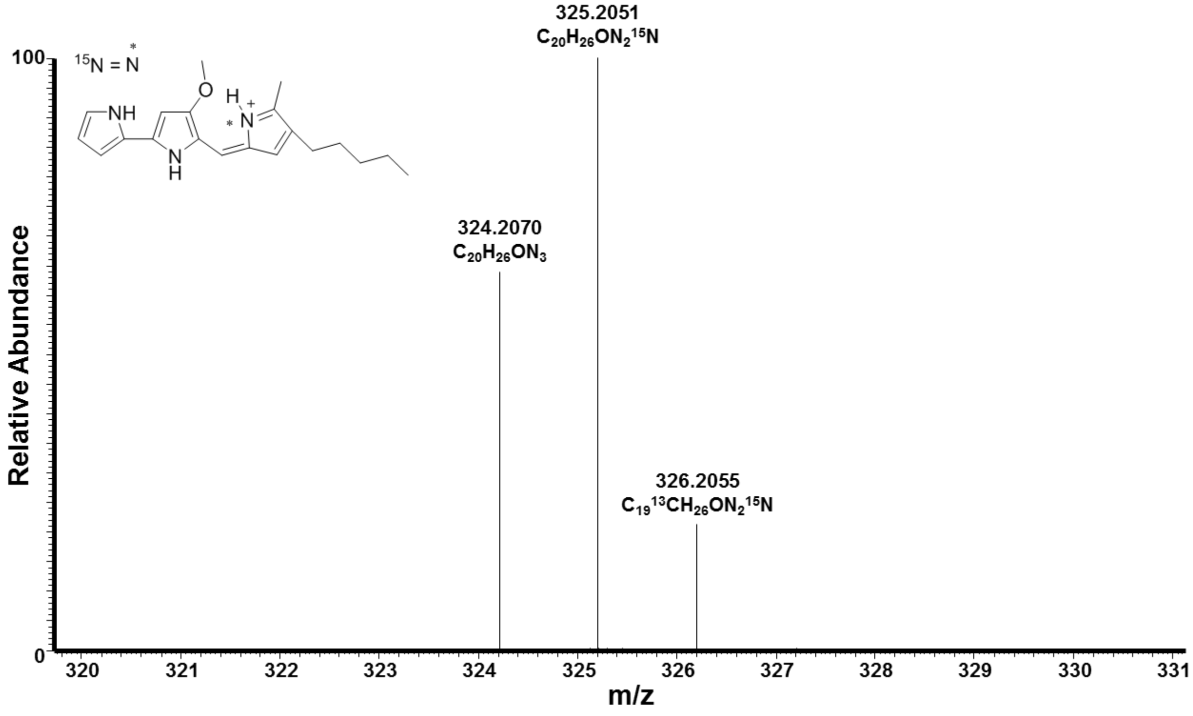

Fig. S102. HRMS spectra of 2-methyl-3-hexyl prodiginine with incorporated ^{15}N derived from $^{15}NH_4Cl$ (compound **4** $[M+H]^+$: *m/z* 339, $C_{21}H_{28}N_2{}^{15}NO$).

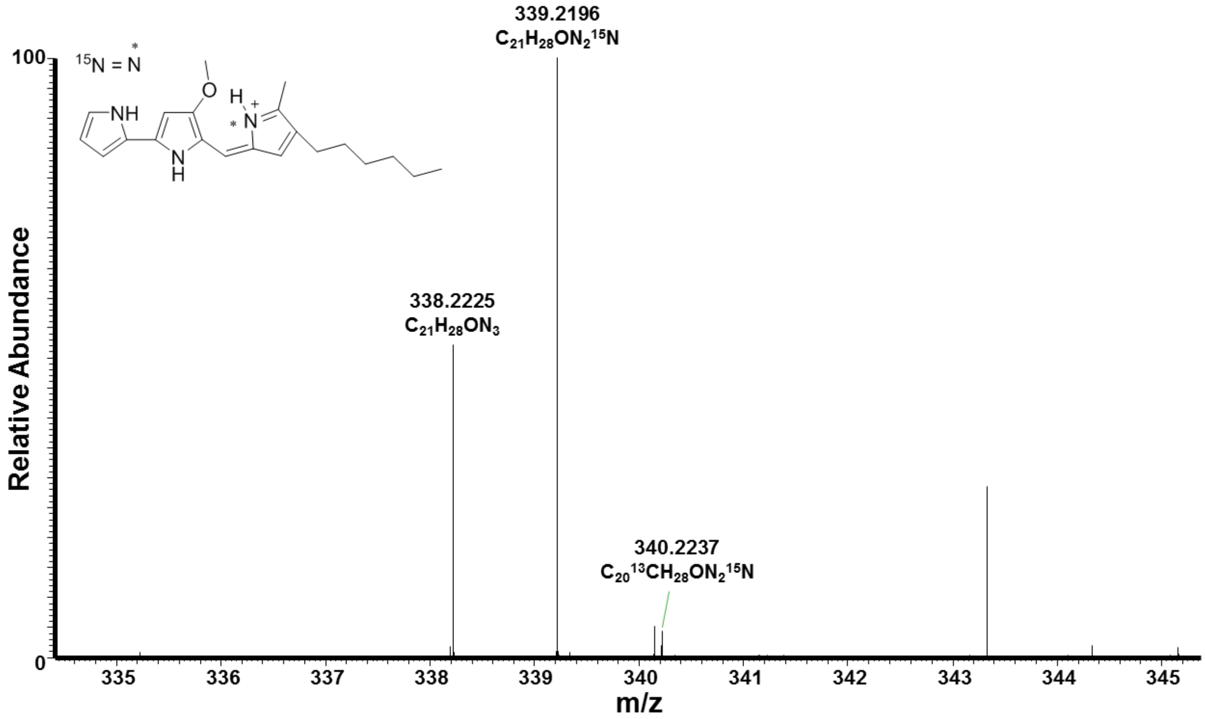

Fig. S103. HRMS spectra of 2-methyl-3-heptyl prodiginine with incorporated ^{15}N derived from $^{15}NH_4Cl$ (compound **5** $[M+H]^+$: *m/z* 353, $C_{22}H_{30}N_2{}^{15}NO$).

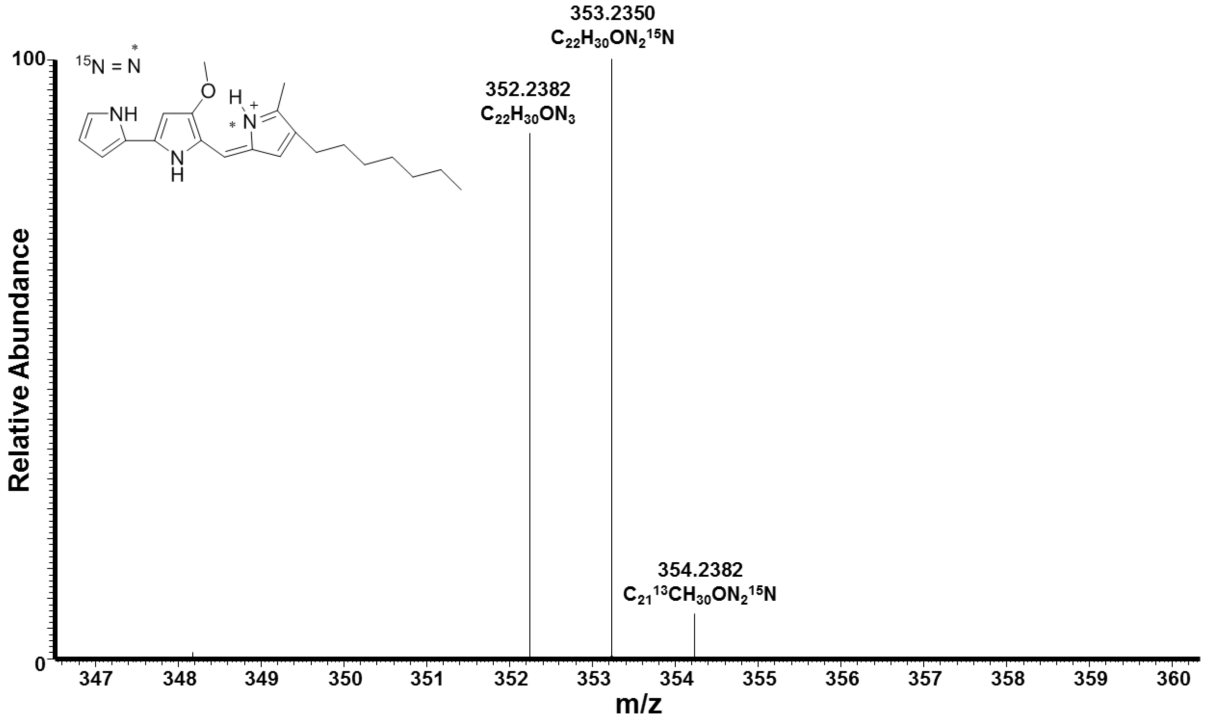

Fig. S104. HRMS spectra of 2-methyl-3-nonyl prodiginine with incorporated ^{15}N derived from $^{15}NH_4Cl$ (compound **7** $[M+H]^+$: *m/z* 381, $C_{24}H_{34}N_2{}^{15}NO$).

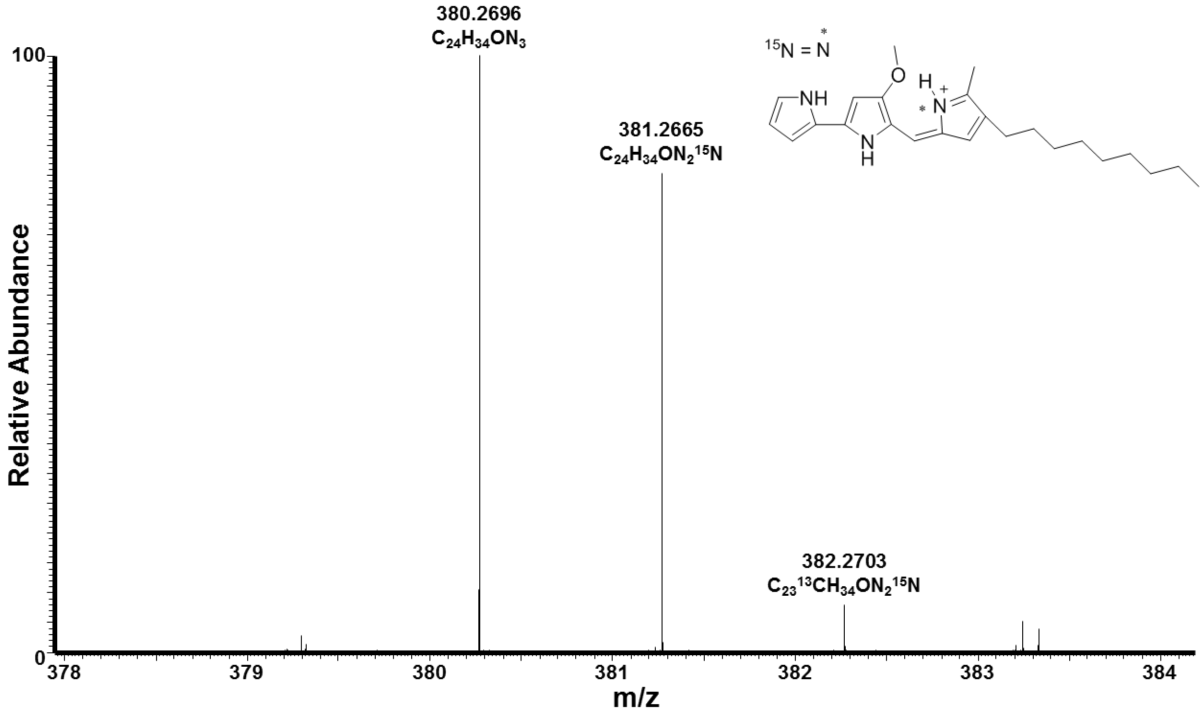

Fig. S105. HRMS spectra of 2-methyl-3-propyl prodiginine with incorporated methyl-D_3 group derived from [methyl-D_3]-L-methionine (compound **1** $[M+H]^+$: *m/z* 299, $C_{18}H_{19}D_3N_3O$).

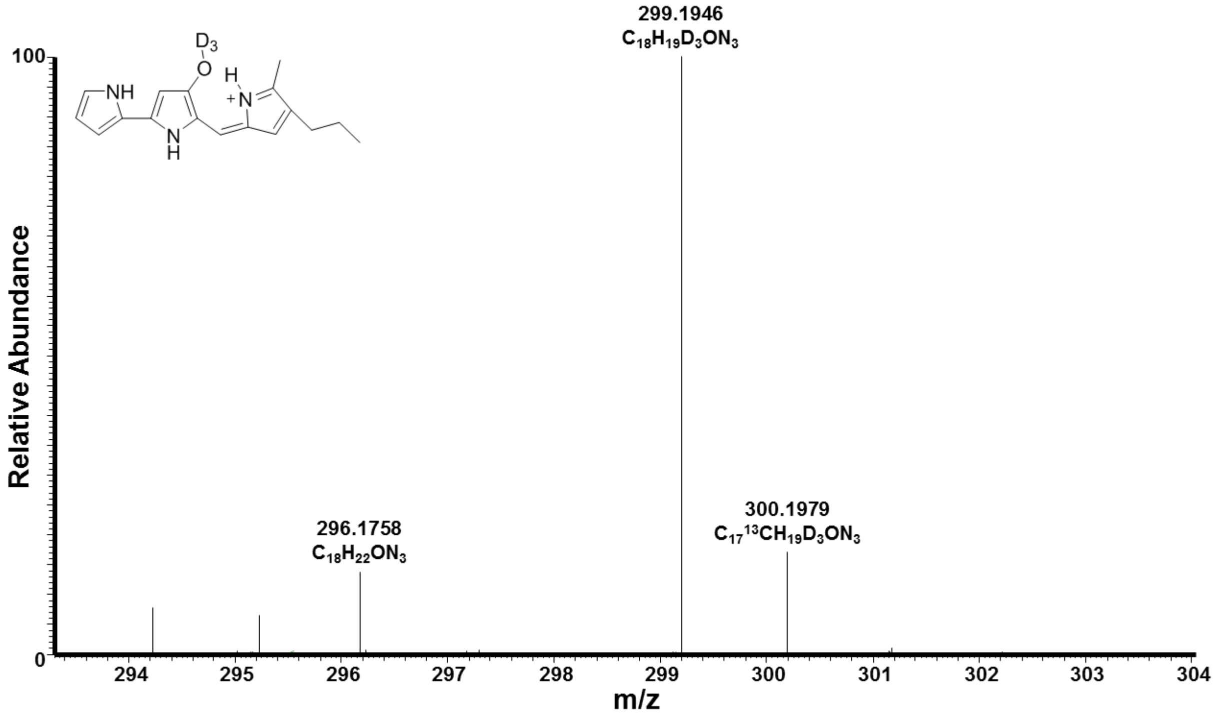

Fig. S106. HRMS spectra of 2-methyl-3-butyl prodiginine with incorporated methyl-D_3 group derived from [methyl-D_3]-L-methionine (compound **2** $[M+H]^+$: *m/z* 313, $C_{19}H_{21}D_3N_3O$).

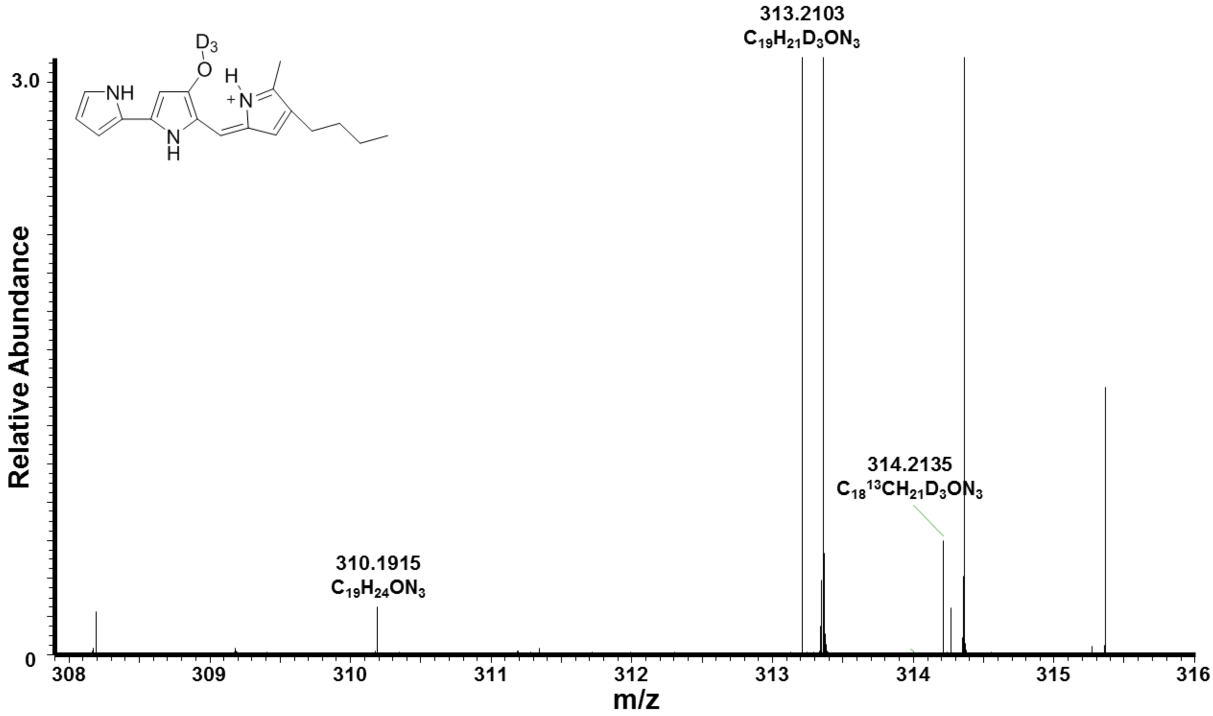

Fig. S107. HRMS spectra of prodigiosin with incorporated methyl-D_3 group derived from [methyl-D_3]-L-methionine (compound **3** $[M+H]^+$: *m/z* 327, $C_{20}H_{23}D_3N_3O$).

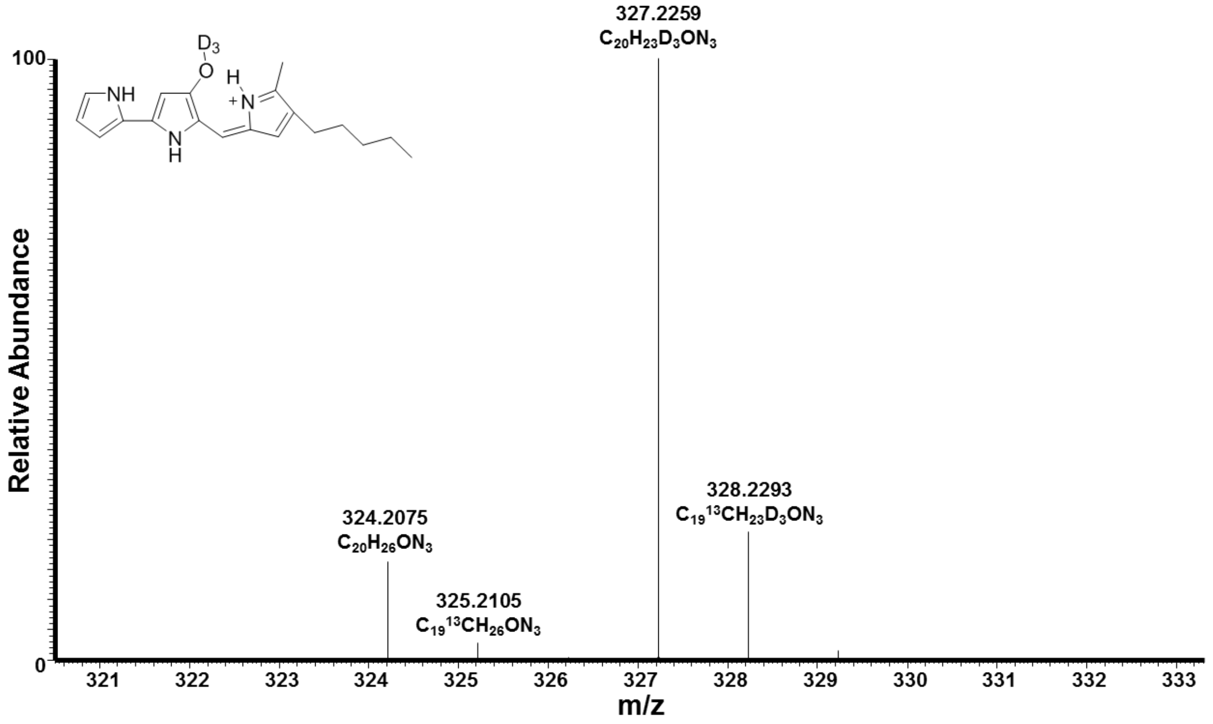

Fig. S108. HRMS spectra of 2-methyl-3-hexyl prodiginine with incorporated methyl-D_3 group derived from [methyl-D_3]-L-methionine (compound **4** $[M+H]^+$: *m/z* 341, $C_{21}H_{25}D_3N_3O$).

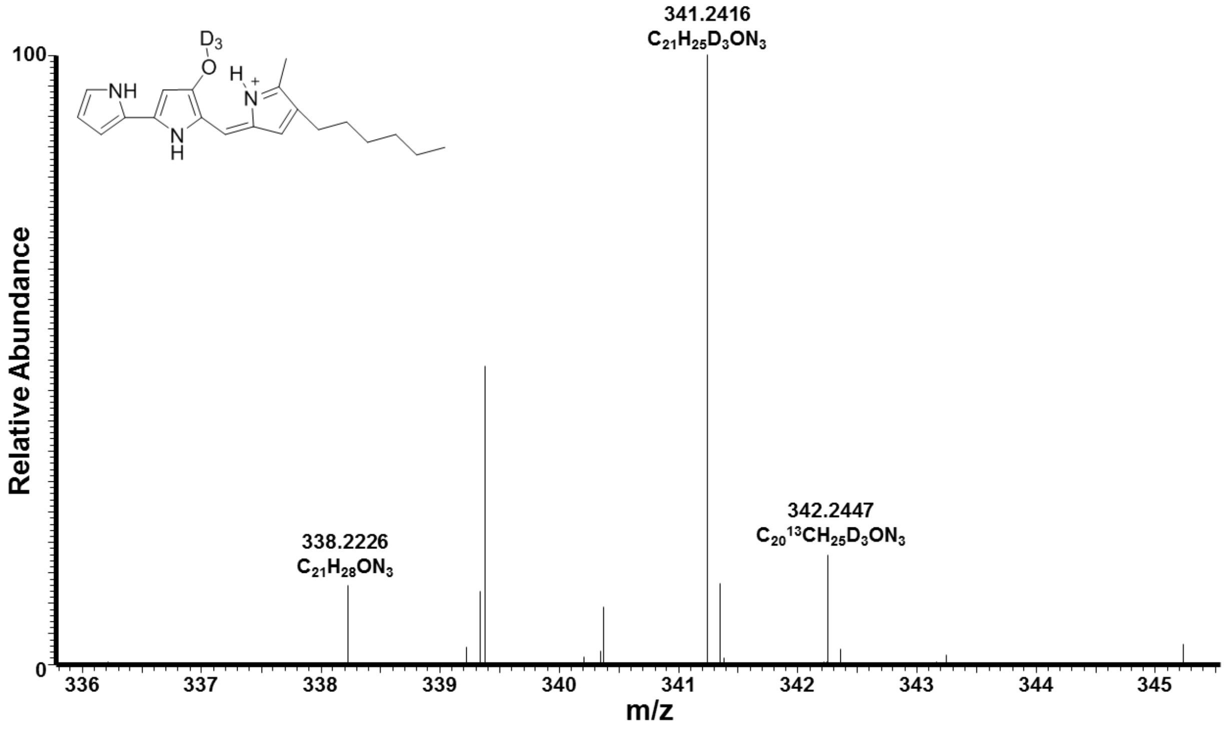

Fig. S109. HRMS spectra of 2-methyl-3-heptyl prodiginine with incorporated methyl-D_3 group derived from [methyl-D_3]-L-methionine (compound **5** $[M+H]^+$: *m/z* 355, $C_{22}H_{27}D_3N_3O$).

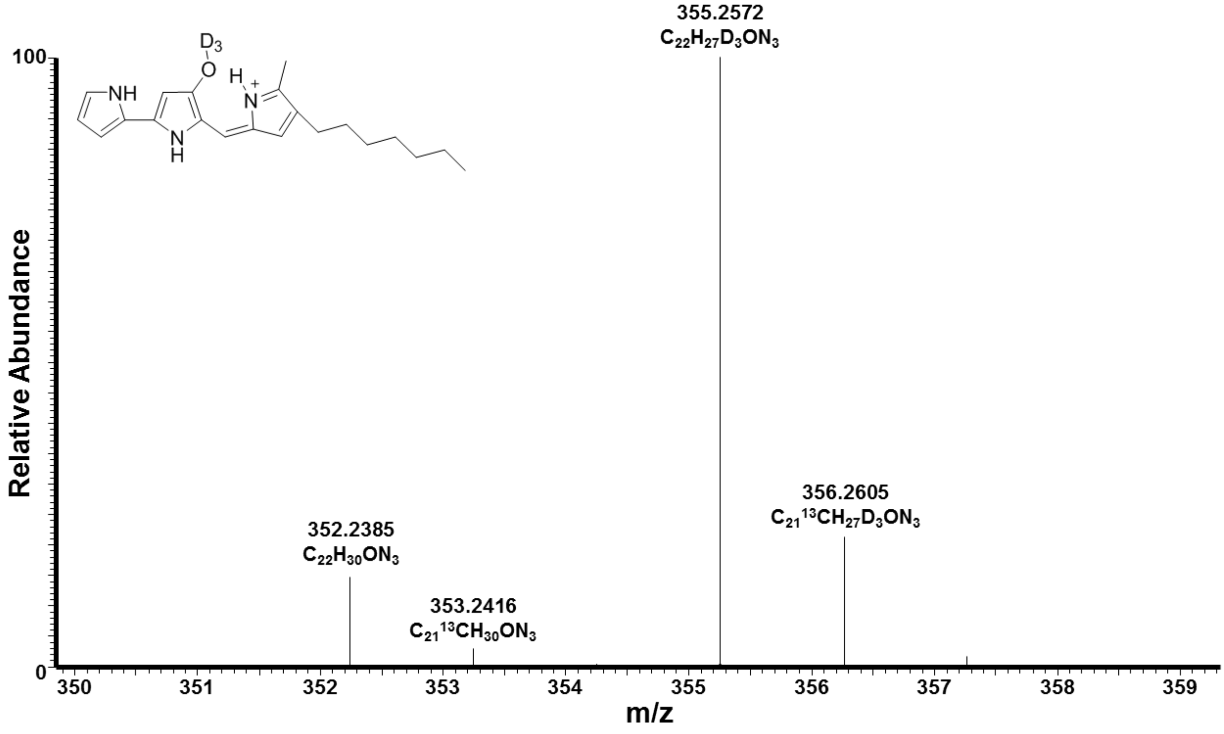

Fig. S110. HRMS spectra of 2-methyl-3-nonyl prodiginine with incorporated methyl-D_3 group derived from [methyl-D_3]-L-methionine (compound **7** $[M+H]^+$: *m/z* 383, $C_{24}H_{31}D_3N_3O$).

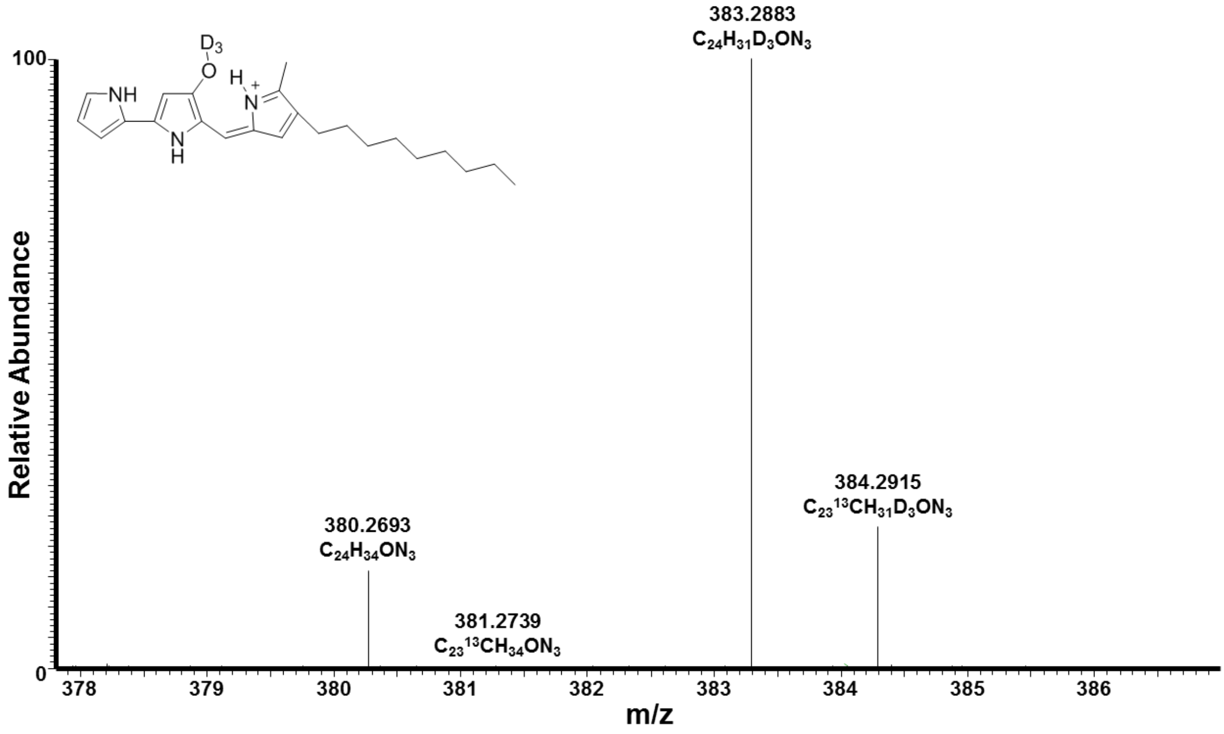

Fig. S111. HRMS spectra of 2-methyl-3-propyl prodiginine with incorporated [1-^{13}C]-L-proline (compound **1** $[M+H]^+$: *m/z* 297, $C_{17}{}^{13}CH_{22}N_3O$).

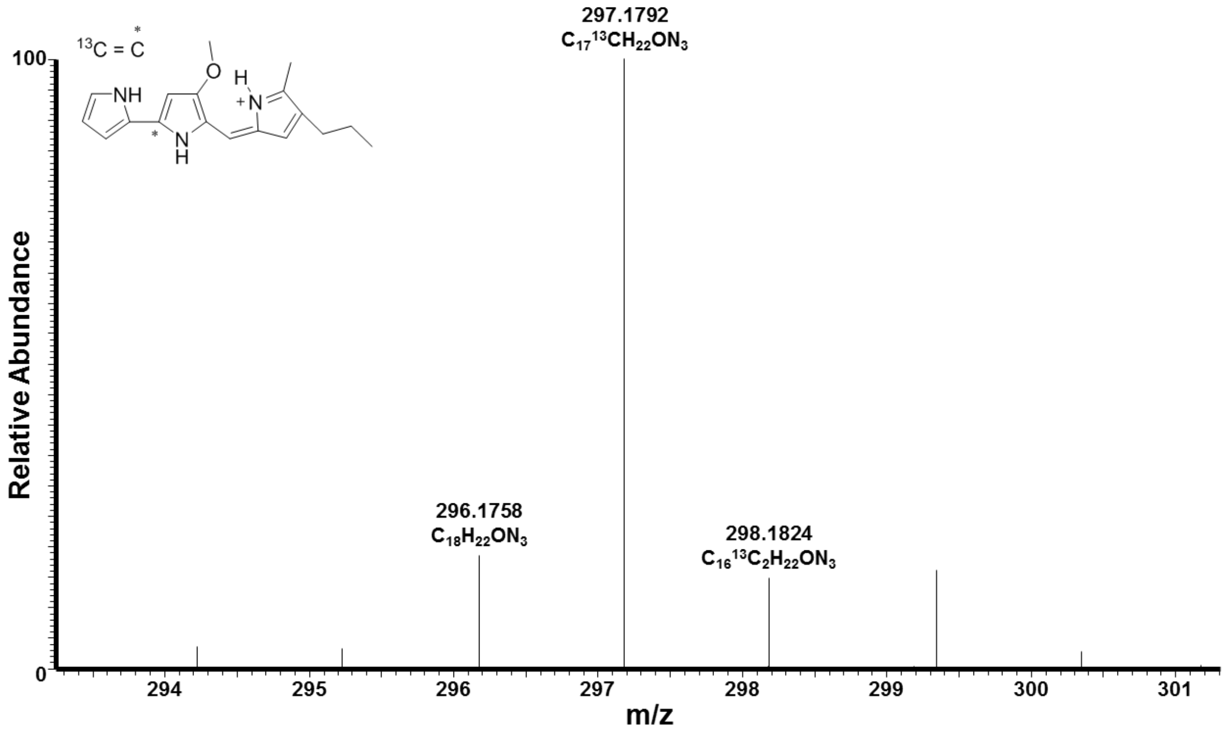

Fig. S112. HRMS spectra of 2-methyl-3-butyl prodiginine with incorporated [1-^{13}C]-L-proline (compound **2** $[M+H]^+$: *m/z* 311, $C_{18}{}^{13}CH_{24}N_3O$).

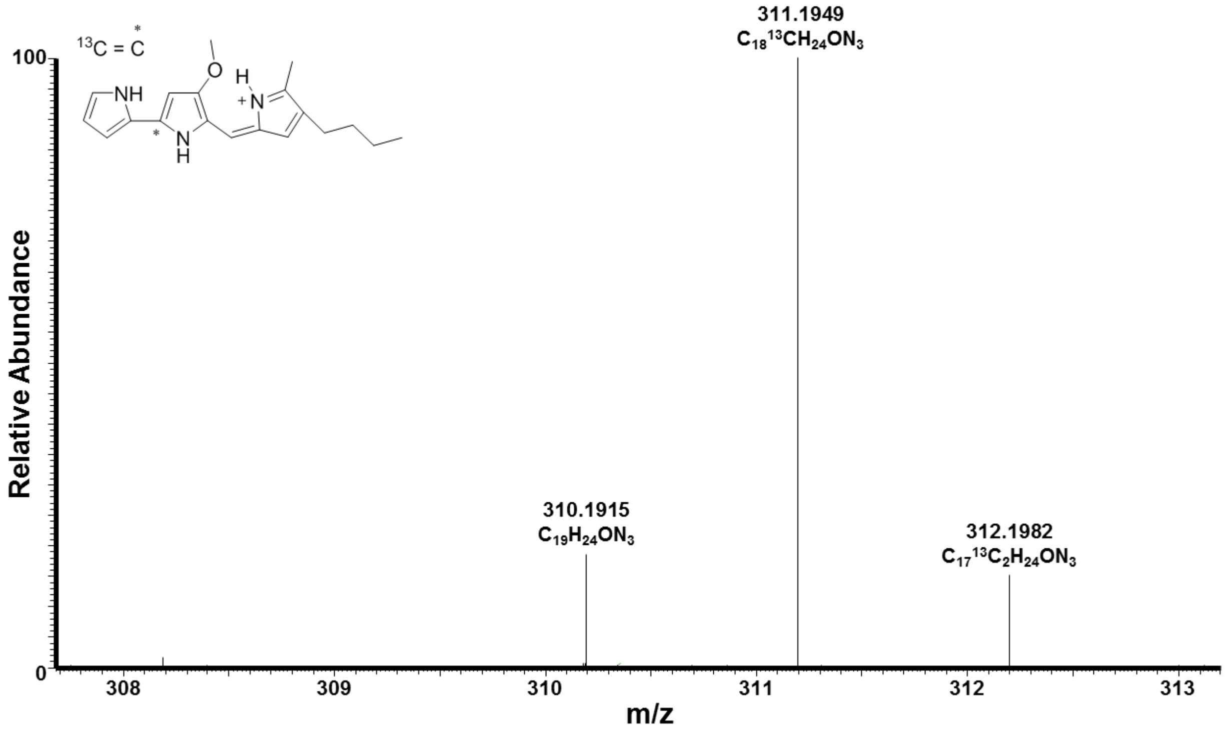

Fig. S113. HRMS spectra of prodigiosin with incorporated [1-^{13}C]-L-proline (compound **3** $[M+H]^+$: *m/z* 325, $C_{19}{}^{13}CH_{26}N_3O$).

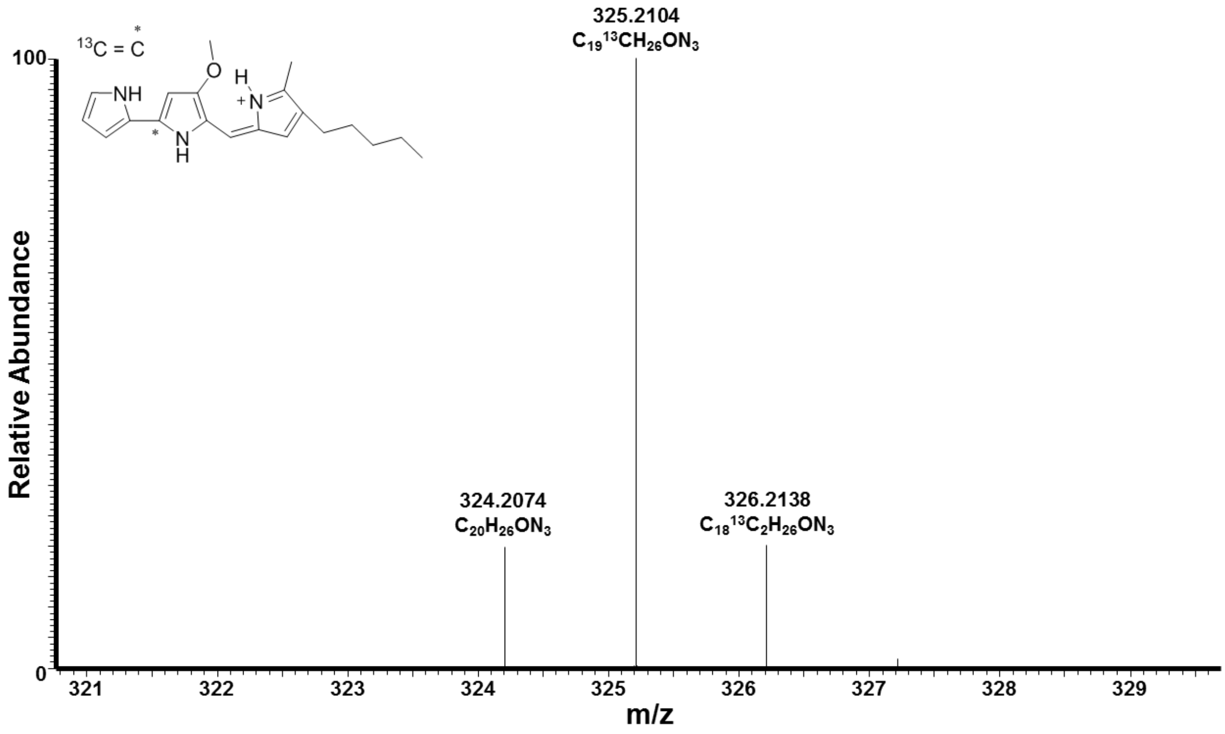

Fig. S114. HRMS spectra of 2-methyl-3-hexyl prodiginine with incorporated [1-^{13}C]-L-proline (compound **4** $[M+H]^+$: *m/z* 339, $C_{20}{}^{13}CH_{28}N_3O$).

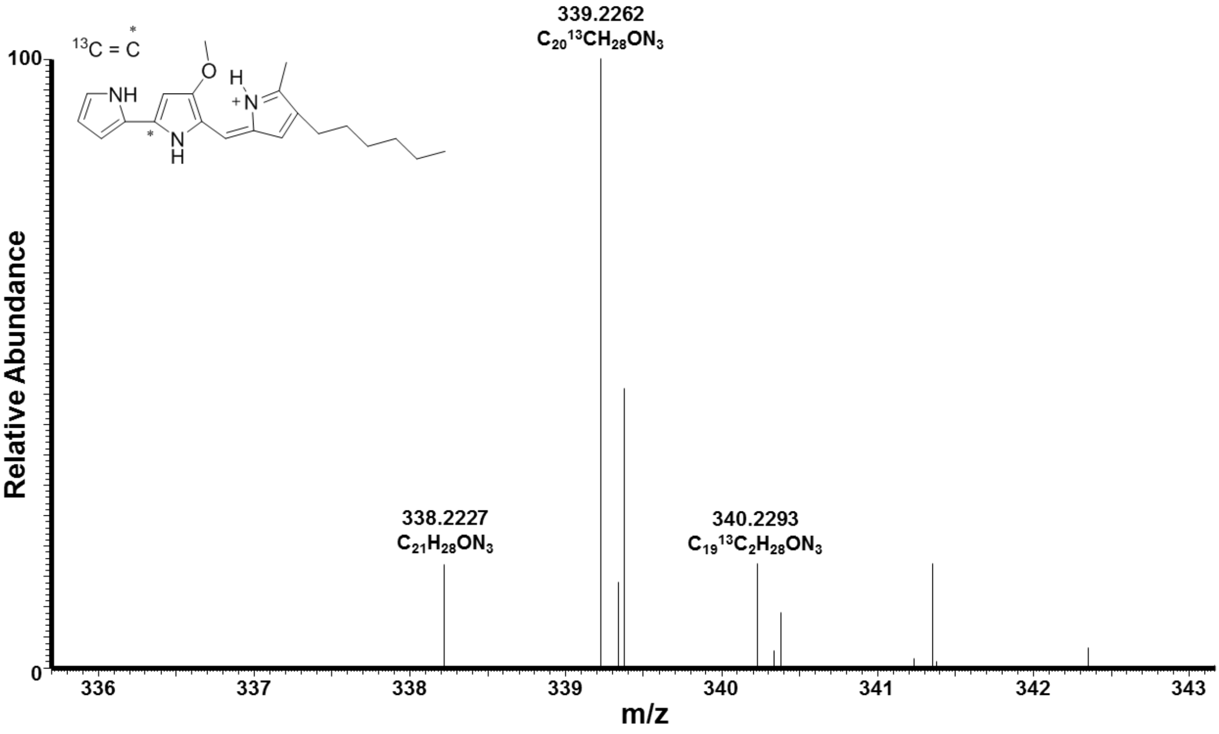

Fig. S115. HRMS spectra of 2-methyl-3-heptyl prodiginine with incorporated [1-^{13}C]-L-proline (compound **5** $[M+H]^+$: *m/z* 353, $C_{21}{}^{13}CH_{30}N_3O$).

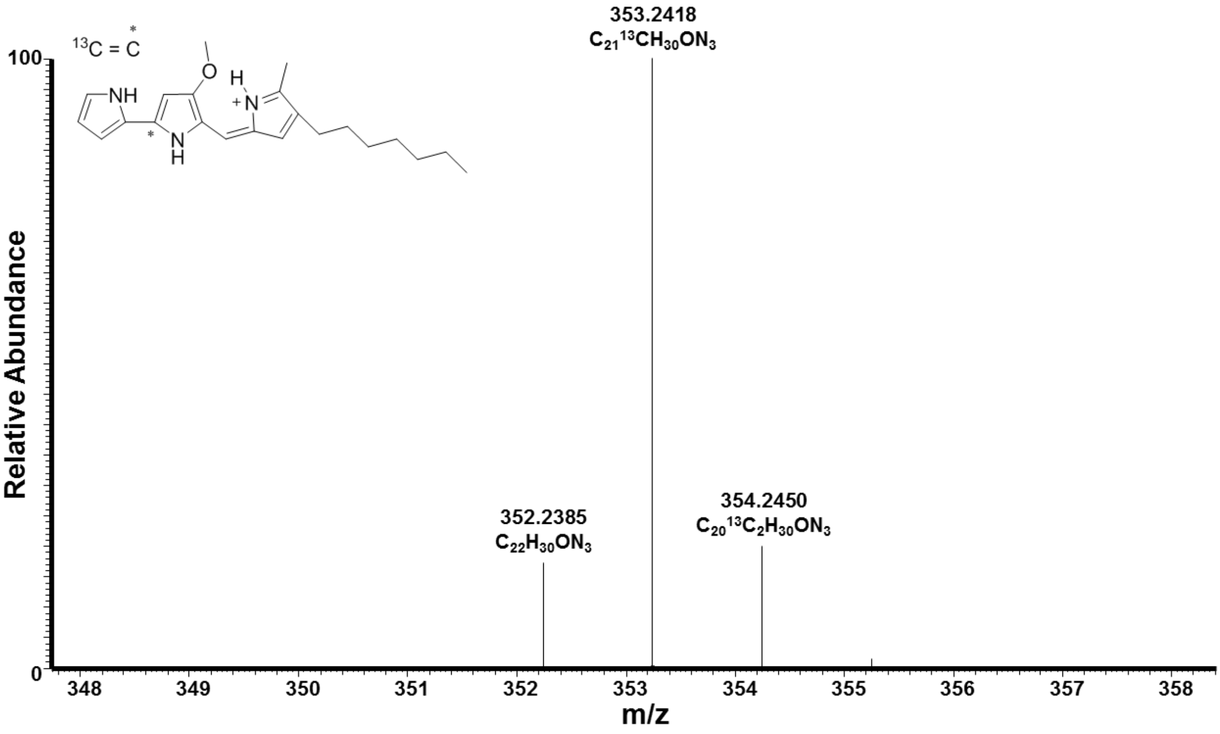

Fig. S116. HRMS spectra of 2-methyl-3-nonyl prodiginine with incorporated [1-^{13}C]-L-proline (compound **7** $[M+H]^+$: *m/z* 381, $C_{23}{}^{13}CH_{34}N_3O$).

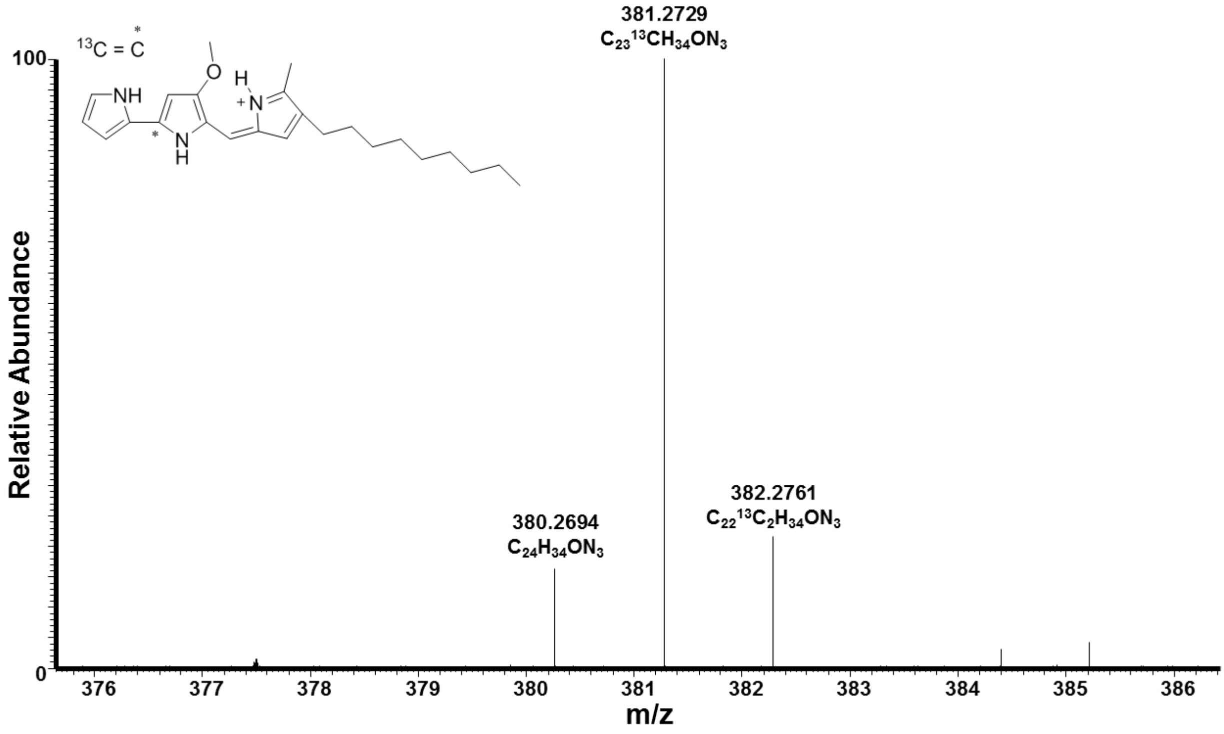

Fig. S117. HRMS[2] of 2-methyl-3-propyl prodiginine with incorporated ^{15}N derived from $^{15}NH_4Cl$ (compound **1** $[M+H]^+$: *m/z* 297, $C_{18}H_{22}N_2{}^{15}NO$).

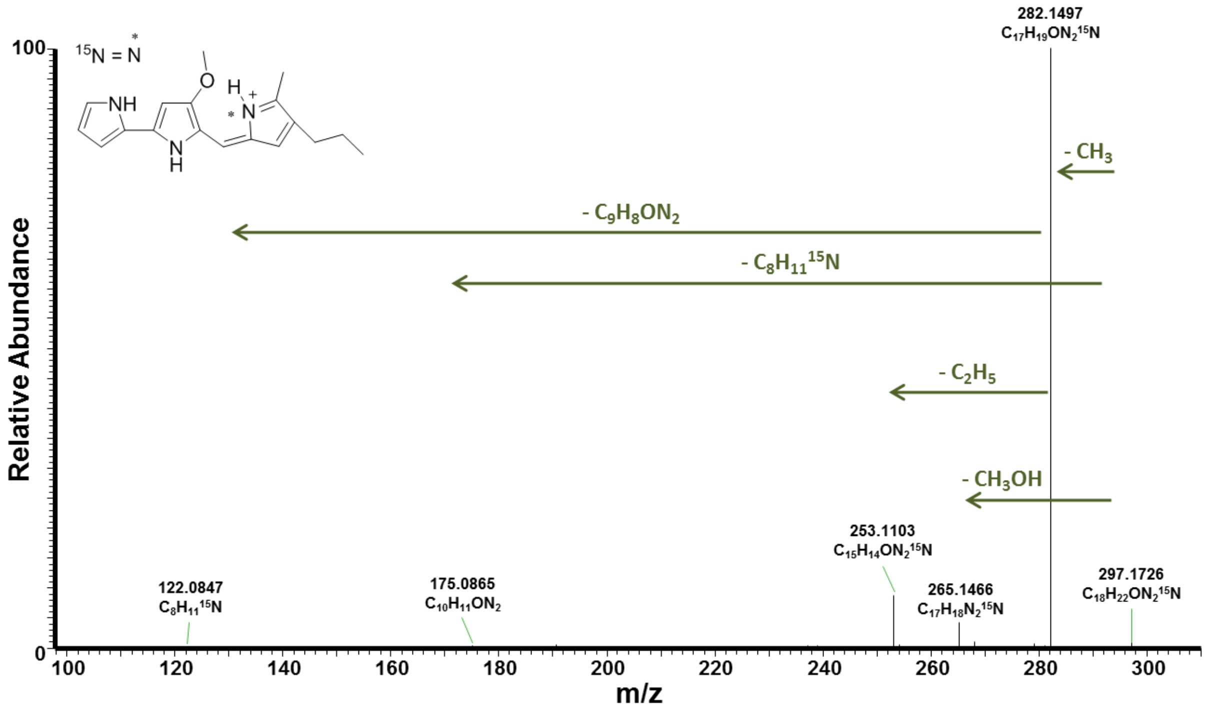

Fig. S118. HRMS[2] of 2-methyl-3-butyl prodiginine with incorporated ^{15}N derived from $^{15}NH_4Cl$ (compound **2** $[M+H]^+$: *m/z* 311, $C_{19}H_{24}N_2{}^{15}NO$).

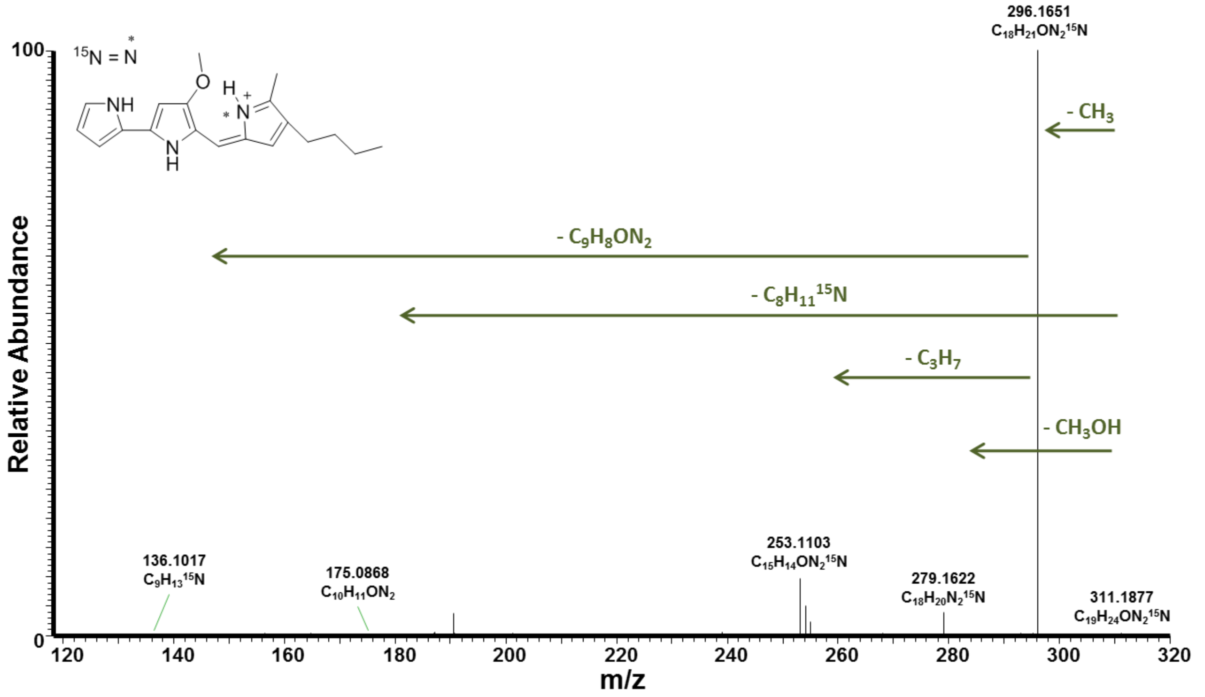

Fig. S119. HRMS^2 of prodigiosin with incorporated ^{15}N derived from $^{15}NH_4Cl$ (compound **3** $[M+H]^+$: *m/z* 325, $C_{20}H_{26}N_2{}^{15}NO$).

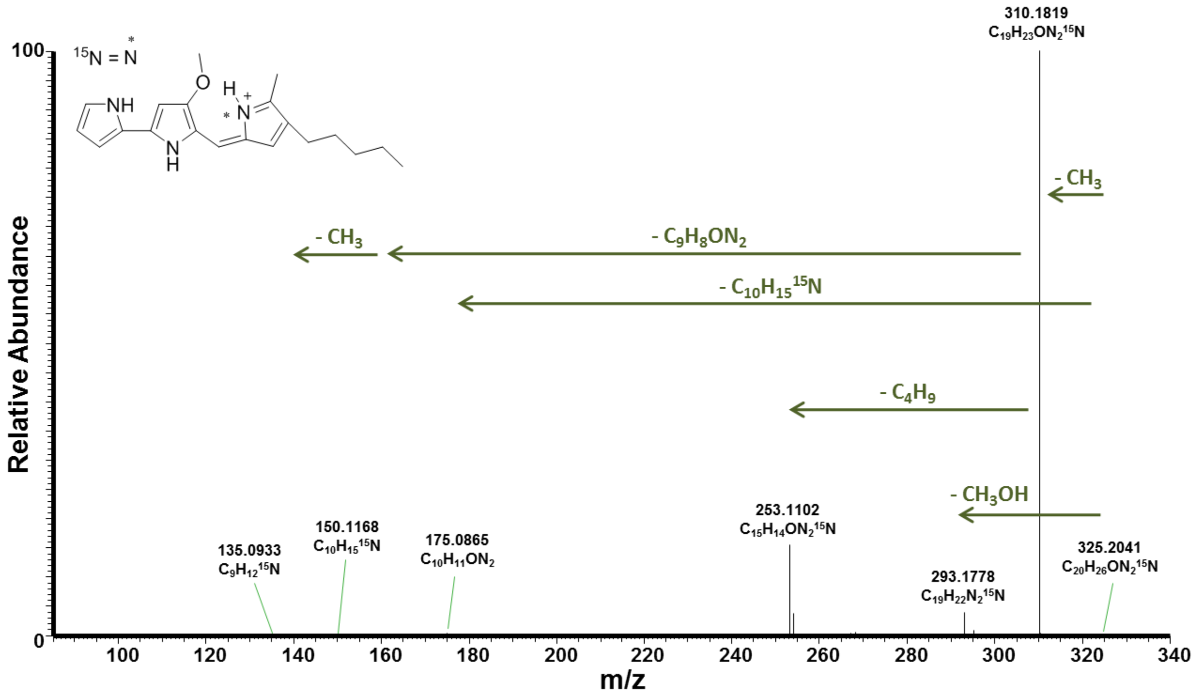

Fig. S120. HRMS^2 of 2-methyl-3-hexyl prodiginine with incorporated ^{15}N derived from $^{15}NH_4Cl$ (compound **4** $[M+H]^+$: *m/z* 339, $C_{21}H_{28}N_2{}^{15}NO$).

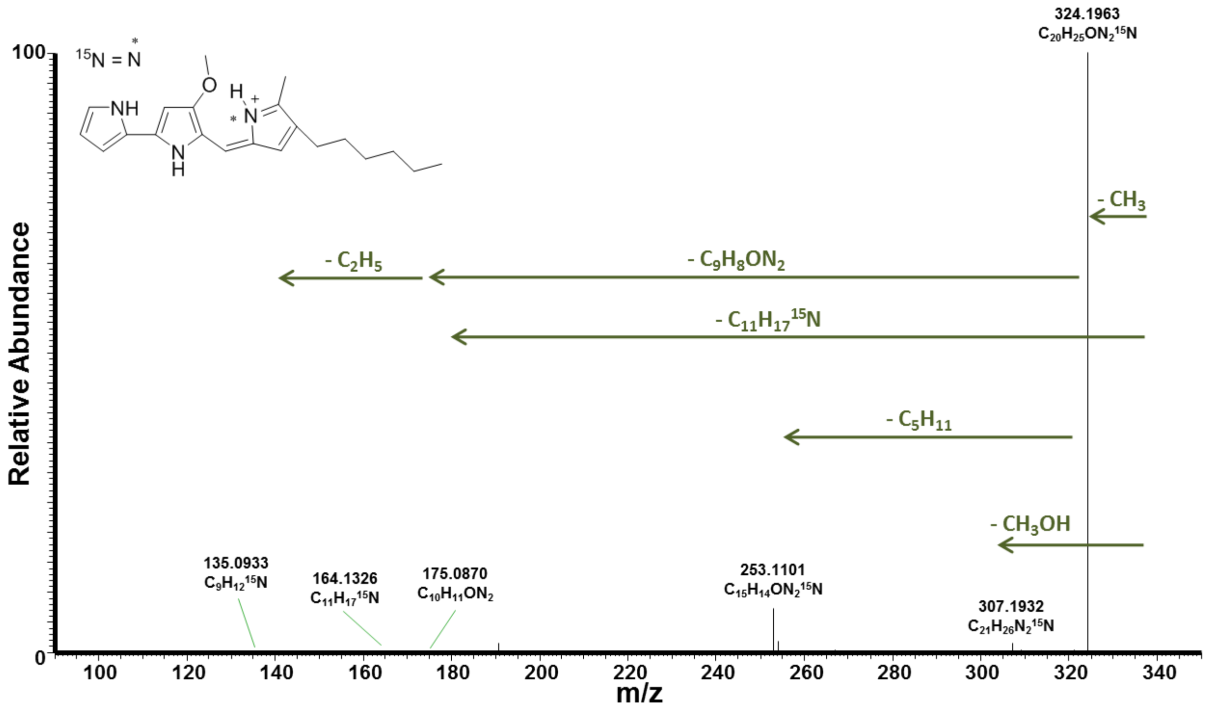

Fig. S121. HRMS[2] of 2-methyl-3-heptyl prodiginine with incorporated ^{15}N derived from $^{15}NH_4Cl$ (compound **5** $[M+H]^+$: *m/z* 353, $C_{22}H_{30}N_2{}^{15}NO$).

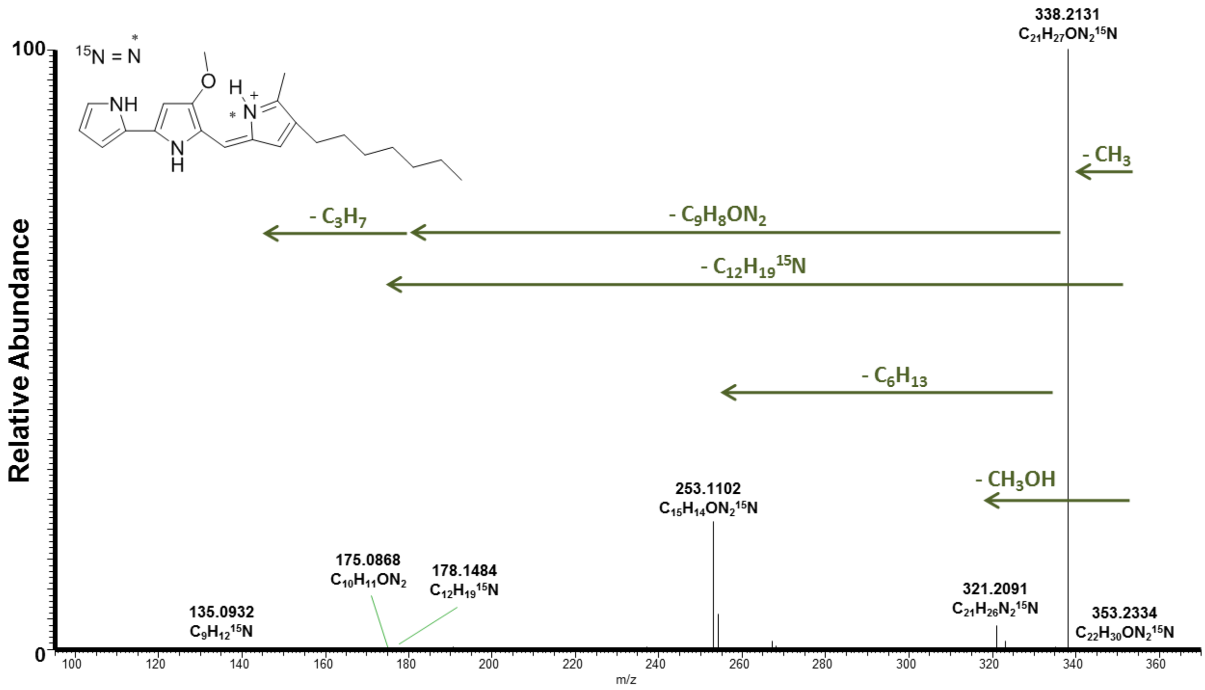

Fig. S122. HRMS[2] of 2-methyl-3-nonyl prodiginine with incorporated ^{15}N derived from $^{15}NH_4Cl$ (compound **7** $[M+H]^+$: *m/z* 381, $C_{24}H_{34}N_2{}^{15}NO$).

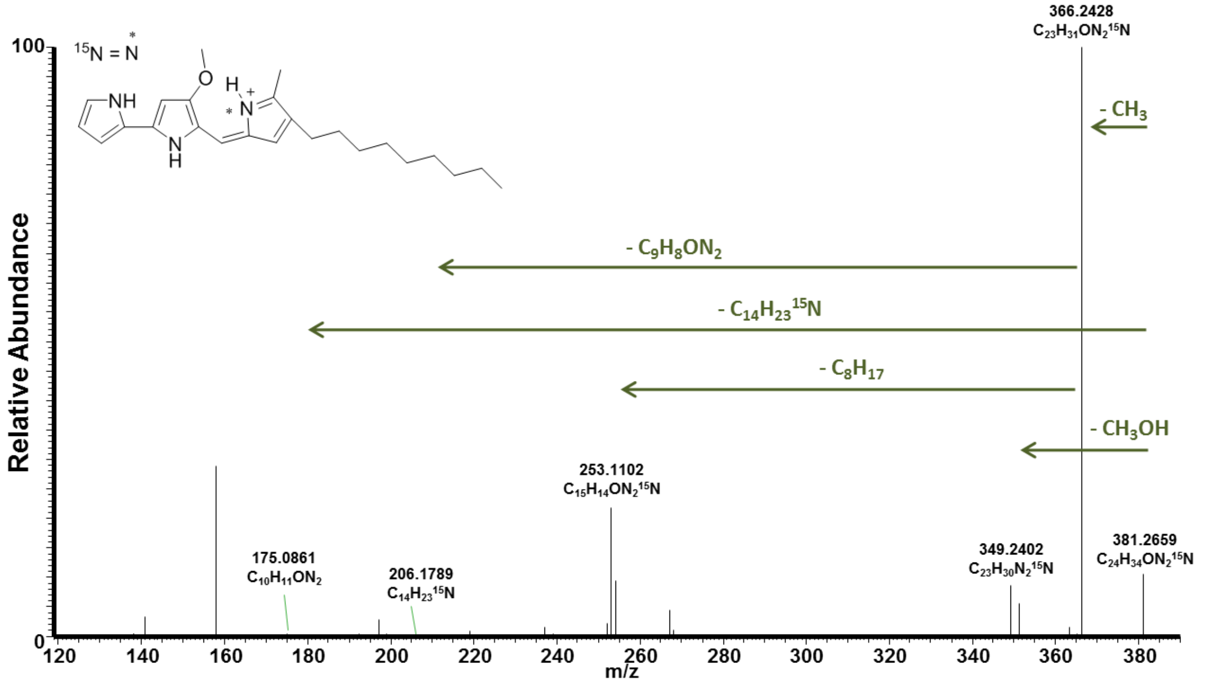

Fig. S123. HRMS[3] of 2-methyl-3-propyl prodiginine with incorporated ^{15}N derived from $^{15}NH_4Cl$ (compound **1** $[M+H]^+$: *m/z* 297 via 272, $C_{18}H_{22}N_2{}^{15}NO$).

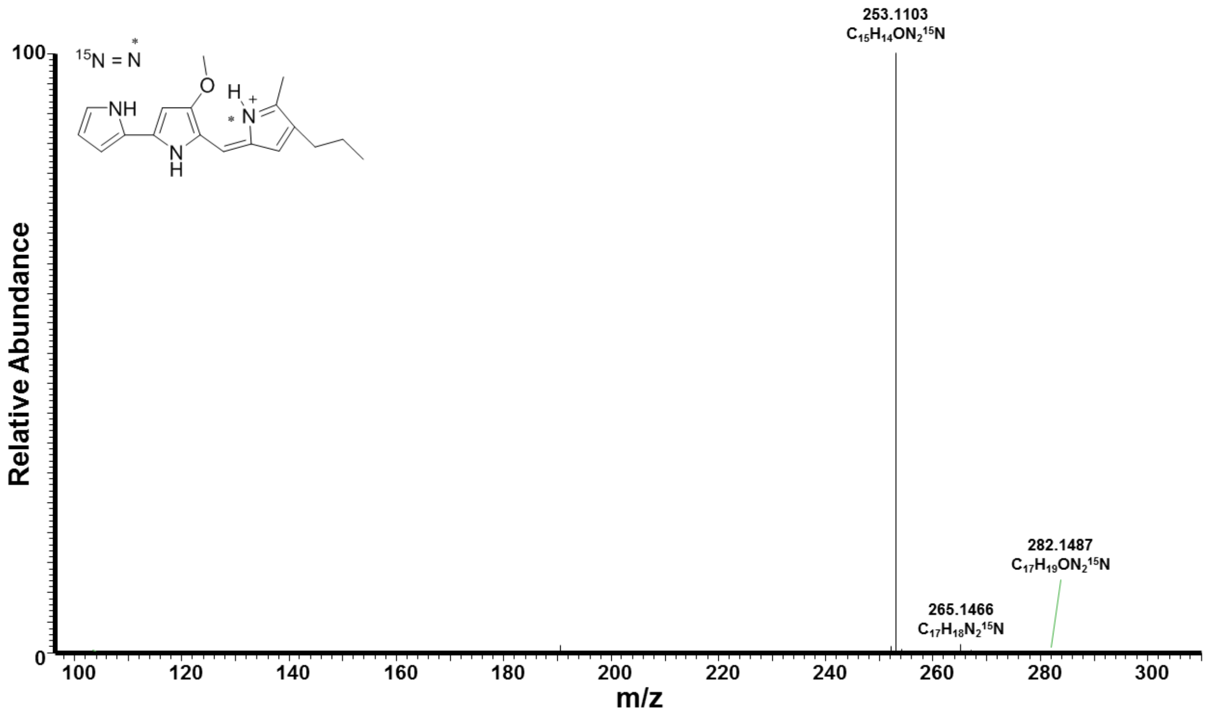

Fig. S124. HRMS[3] of 2-methyl-3-butyl prodiginine with incorporated ^{15}N derived from $^{15}NH_4Cl$ (compound **2** $[M+H]^+$: *m/z* 311 via 296, $C_{19}H_{24}N_2{}^{15}NO$).

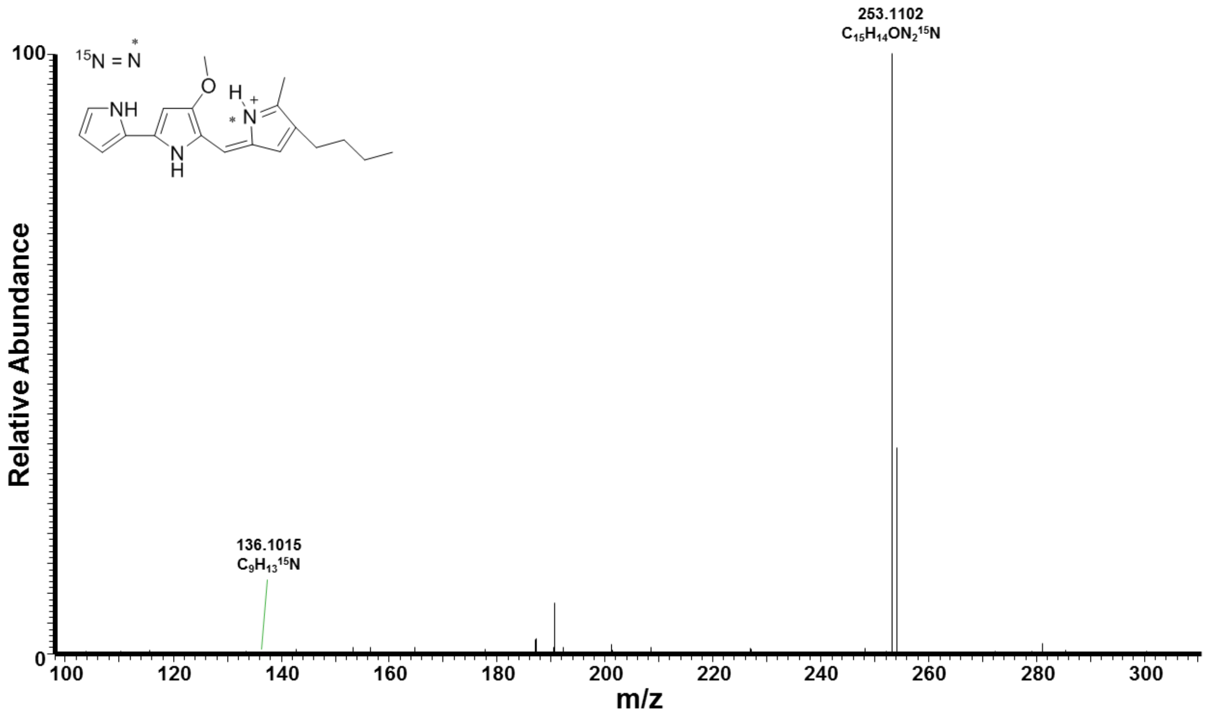

Fig. S125. HRMS[3] of prodigiosin with incorporated ^{15}N derived from $^{15}NH_4Cl$ (compound **3** $[M+H]^+$: *m/z* 325 via 310, $C_{20}H_{26}N_2{}^{15}NO$).

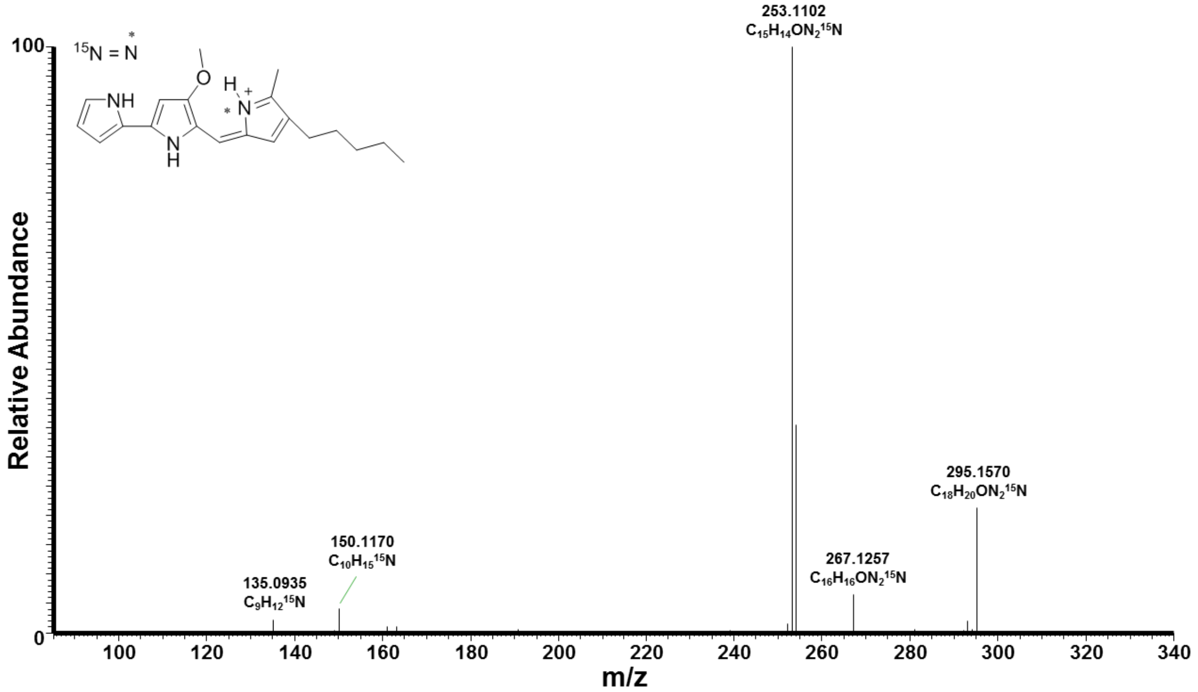

Fig. S126. HRMS[3] of 2-methyl-3-hexyl prodiginine with incorporated ^{15}N derived from $^{15}NH_4Cl$ (compound **4** $[M+H]^+$: *m/z* 339 via 324, $C_{21}H_{28}N_2{}^{15}NO$).

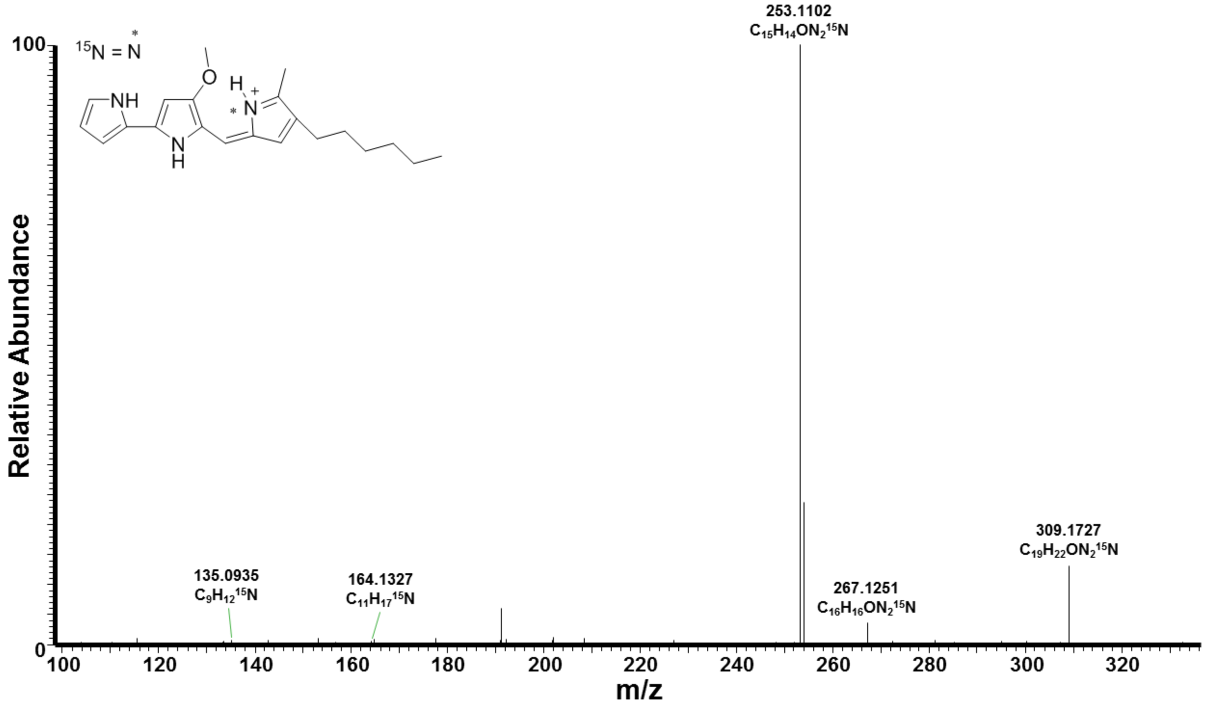

Fig. S127. HRMS[3] of 2-methyl-3-heptyl prodiginine with incorporated ^{15}N derived from $^{15}NH_4Cl$ (compound **5** $[M+H]^+$: *m/z* 353 via 338, $C_{22}H_{30}N_2{}^{15}NO$).

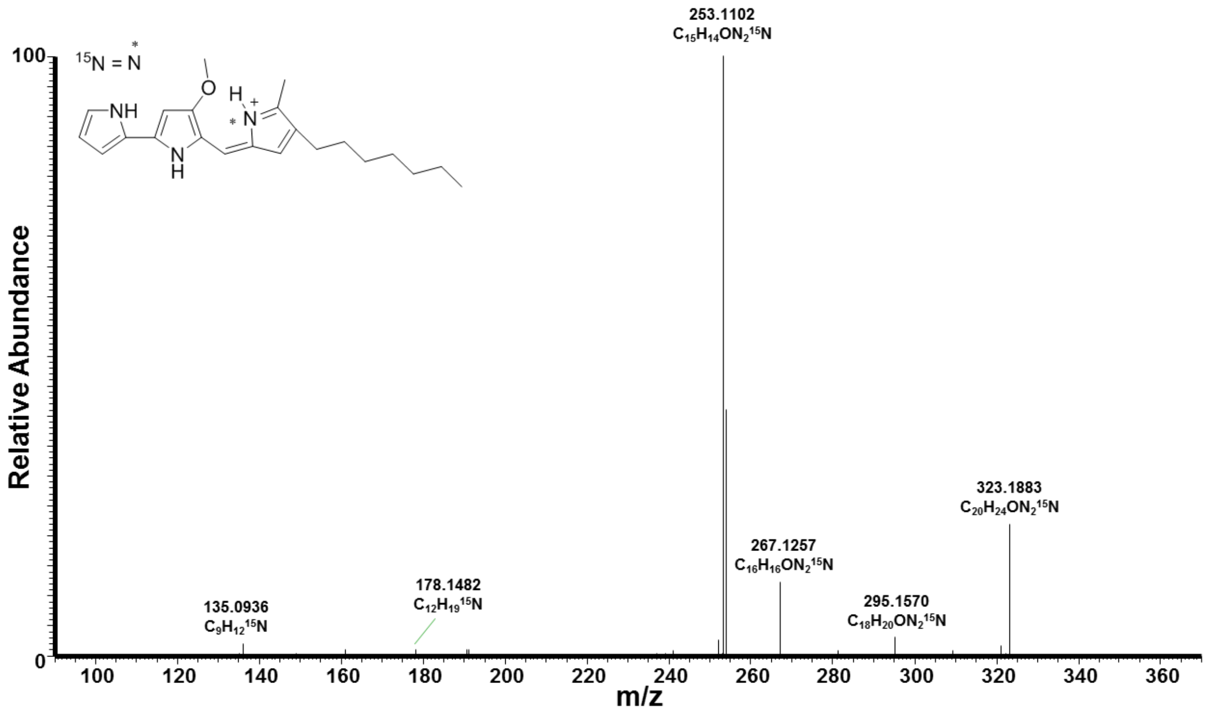

Fig. S128. HRMS[3] of 2-methyl-3-nonyl prodiginine with incorporated ^{15}N derived from $^{15}NH_4Cl$ (compound **7** $[M+H]^+$: *m/z* 381 via 366, $C_{24}H_{34}N_2{}^{15}NO$).

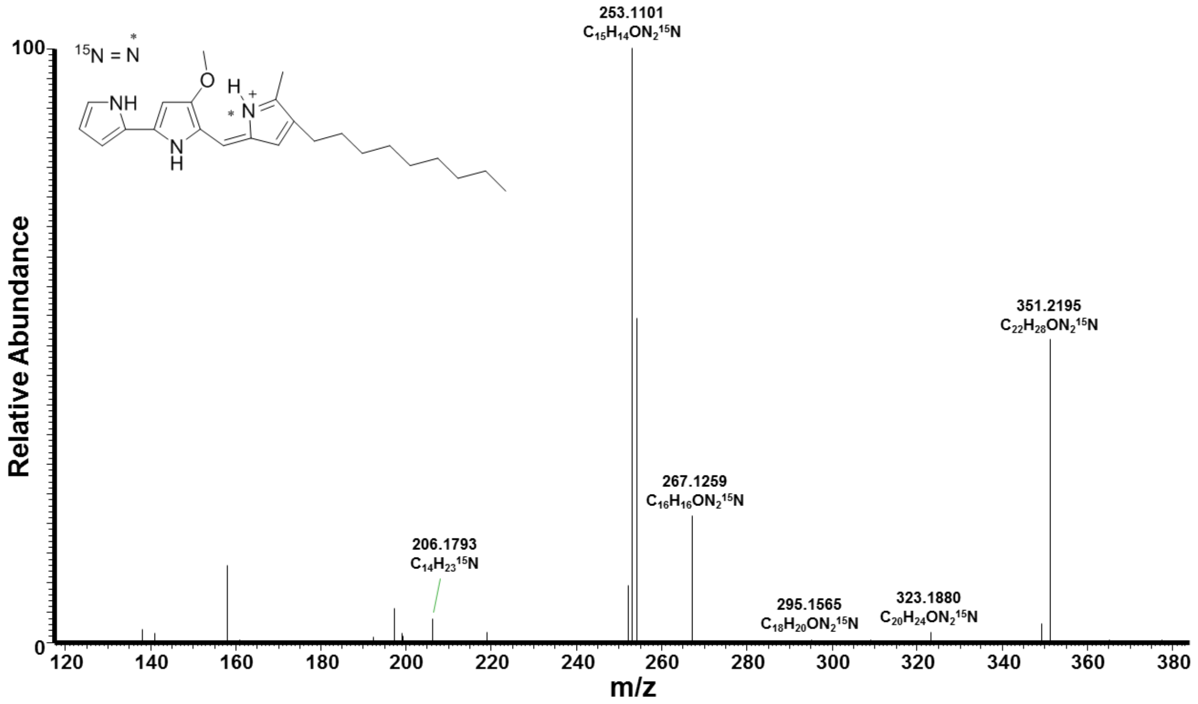

Fig. S129. Proposed mass spectral fragmentation pathway of 2-methyl-3-propyl prodiginine with incorporated ^{15}N derived from $^{15}NH_4Cl$ (compound **1** $[M+H]^+$: *m/z* 297, $C_{18}H_{22}N_2{}^{15}NO$).

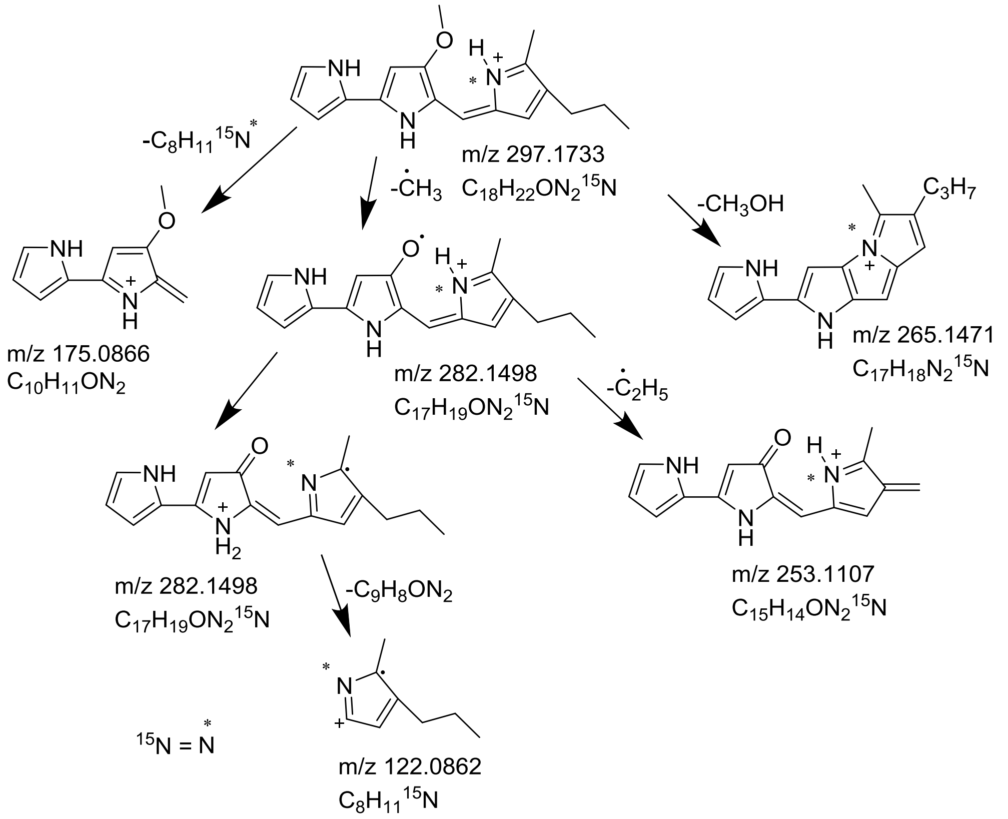

Fig. S130. Proposed mass spectral fragmentation pathway of 2-methyl-3-butyl prodiginine with incorporated ^{15}N derived from $^{15}NH_4Cl$ (compound **2** $[M+H]^+$: *m/z* 311, $C_{19}H_{24}N_2{}^{15}NO$).

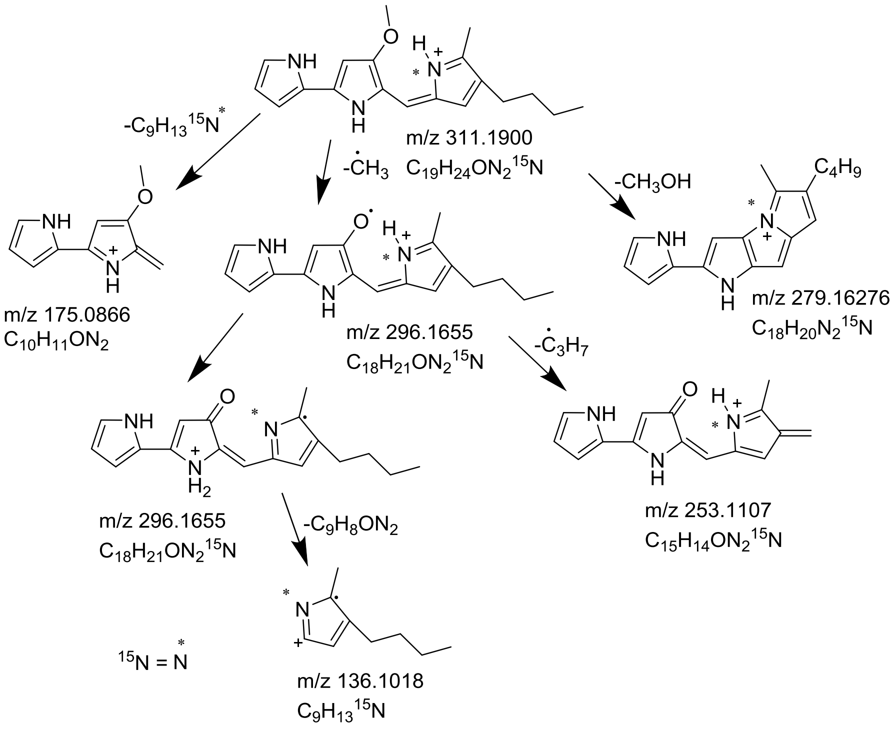

Fig. S131. Proposed mass spectral fragmentation pathway of prodigiosin with incorporated ^{15}N derived from $^{15}NH_4Cl$ (compound **3** $[M+H]^+$: *m/z* 325, $C_{20}H_{26}N_2{}^{15}NO$).

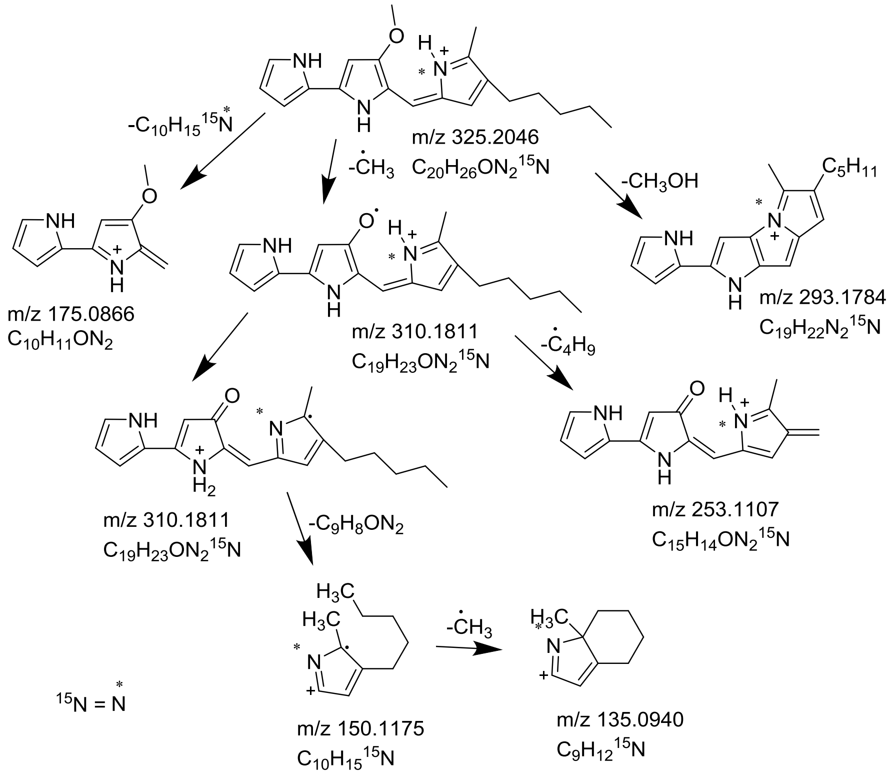

Fig. S132. Proposed mass spectral fragmentation pathway of 2-methyl-3-hexyl prodiginine with incorporated ^{15}N derived from $^{15}NH_4Cl$ (compound **4** $[M+H]^+$: *m/z* 339, $C_{21}H_{28}N_2{}^{15}NO$).

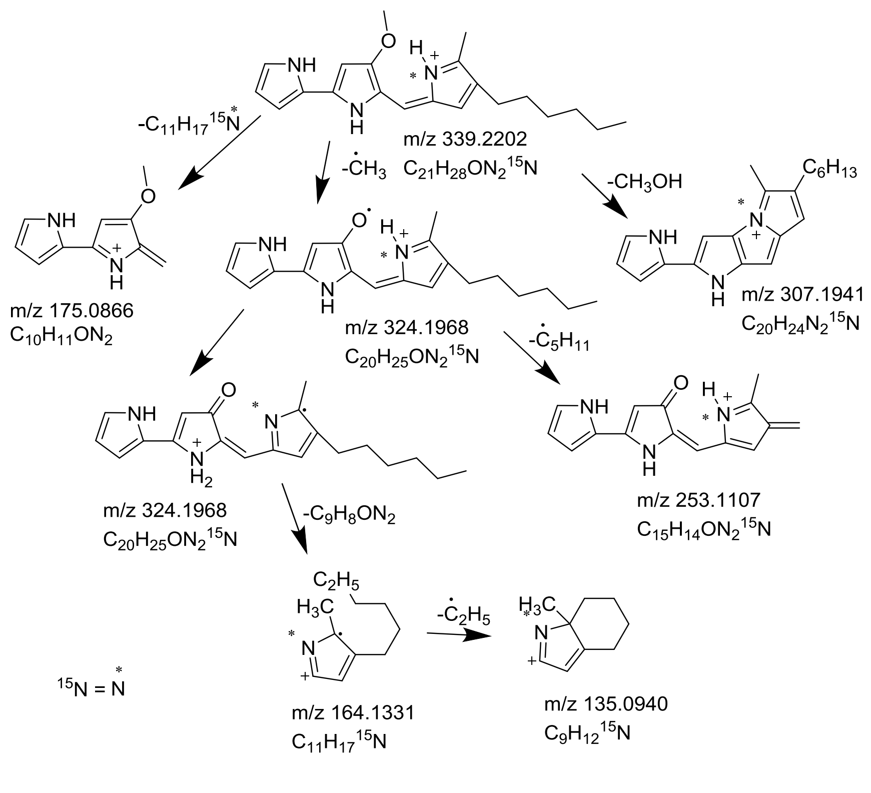

Fig. S133. Proposed mass spectral fragmentation pathway of 2-methyl-3-heptyl prodiginine with incorporated ^{15}N derived from $^{15}NH_4Cl$ (compound **5** $[M+H]^+$: *m/z* 353, $C_{22}H_{30}N_2{}^{15}NO$).

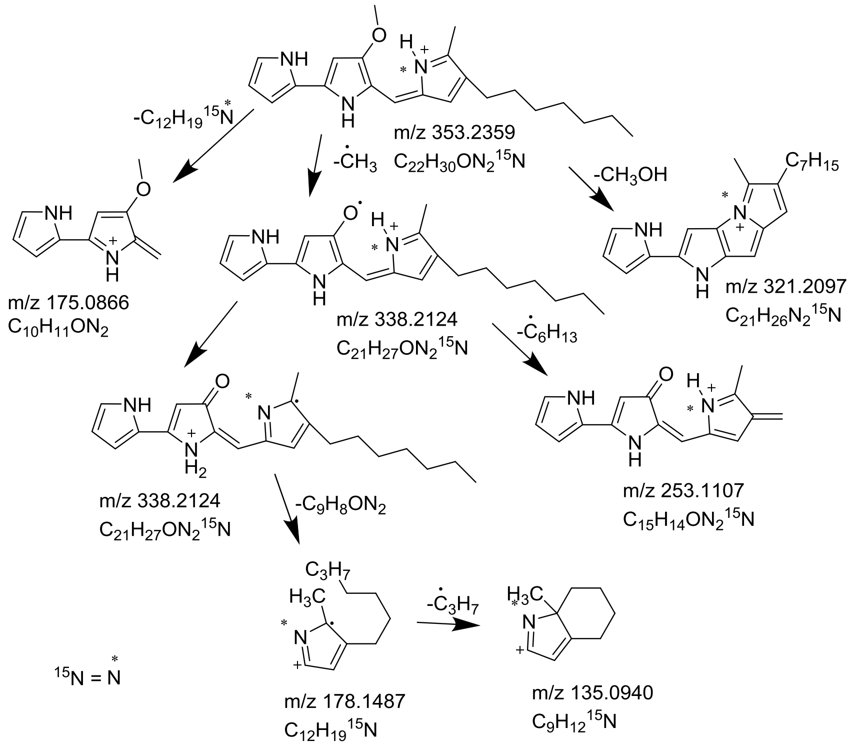

Fig. S134. Proposed mass spectral fragmentation pathway of 2-methyl-3-nonyl prodiginine with incorporated ^{15}N derived from $^{15}NH_4Cl$ (compound **7** $[M+H]^+$: *m/z* 381 via 366, $C_{24}H_{34}N_2{}^{15}NO$).

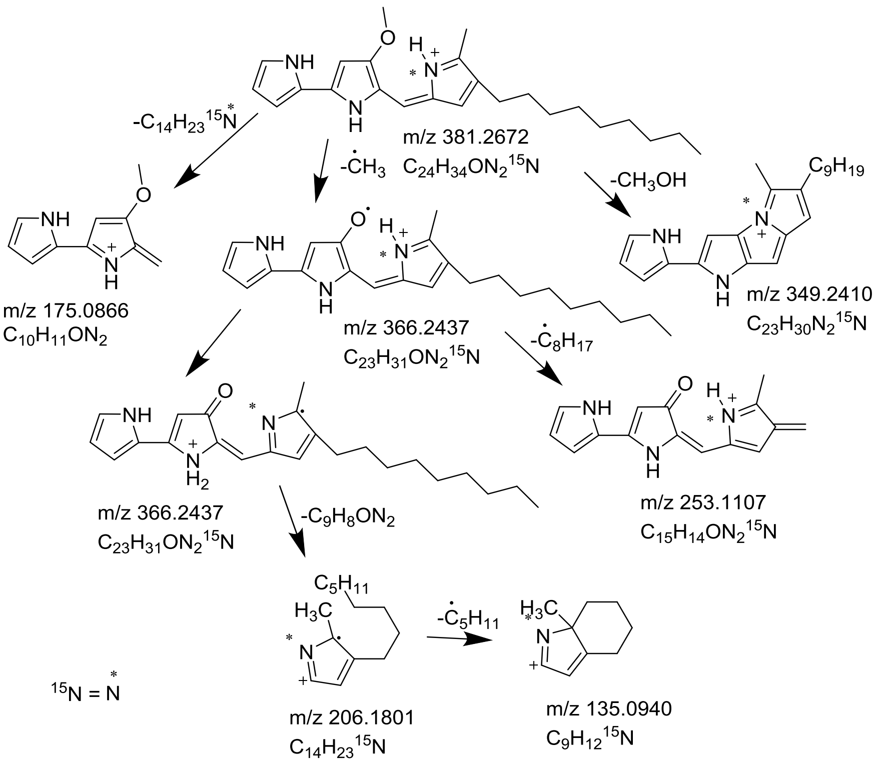

Fig. S135. HRMS[2] of 2-methyl-3-propyl prodiginine with incorporated methyl-D3 group derived from [methyl-D3]-L-methionine (compound **1** $[M+H]^+$: *m/z* 299, $C_{18}H_{19}D_3N_3O$).

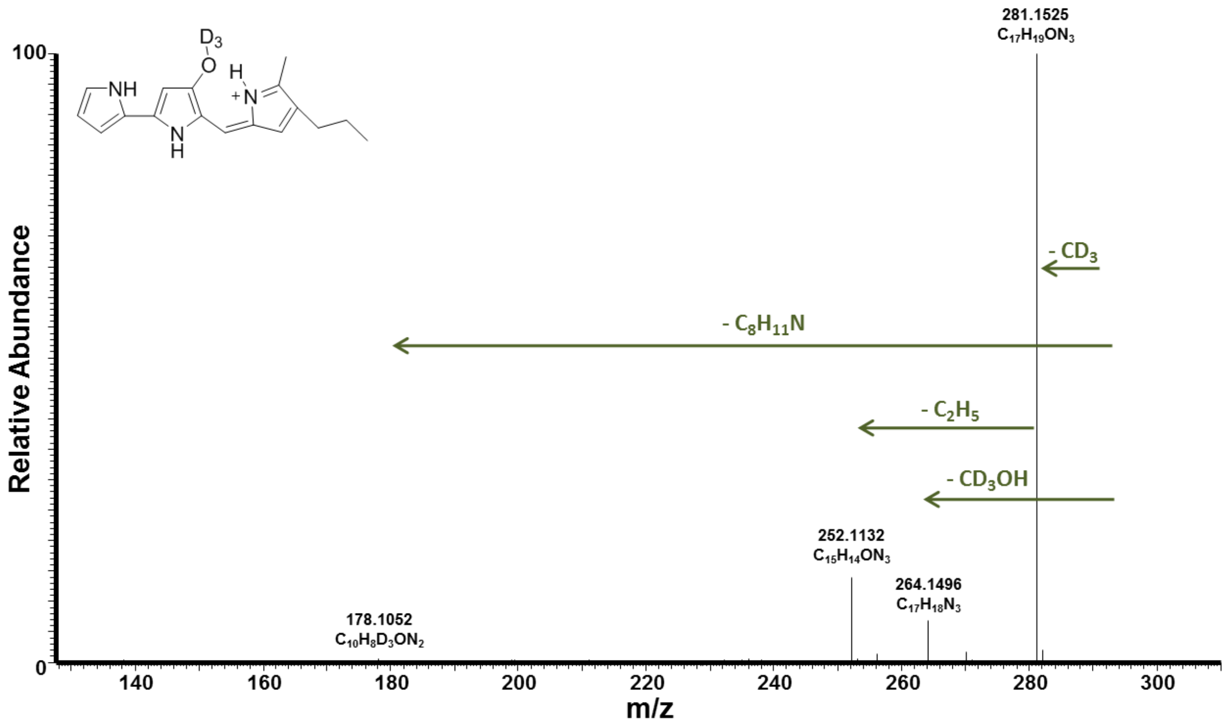

Fig. S136. HRMS[2] of 2-methyl-3-butyl prodiginine with incorporated methyl-D3 group derived from [methyl-D3]-L-methionine (compound **2** $[M+H]^+$: *m/z* 313, $C_{19}H_{21}D_3N_3O$).

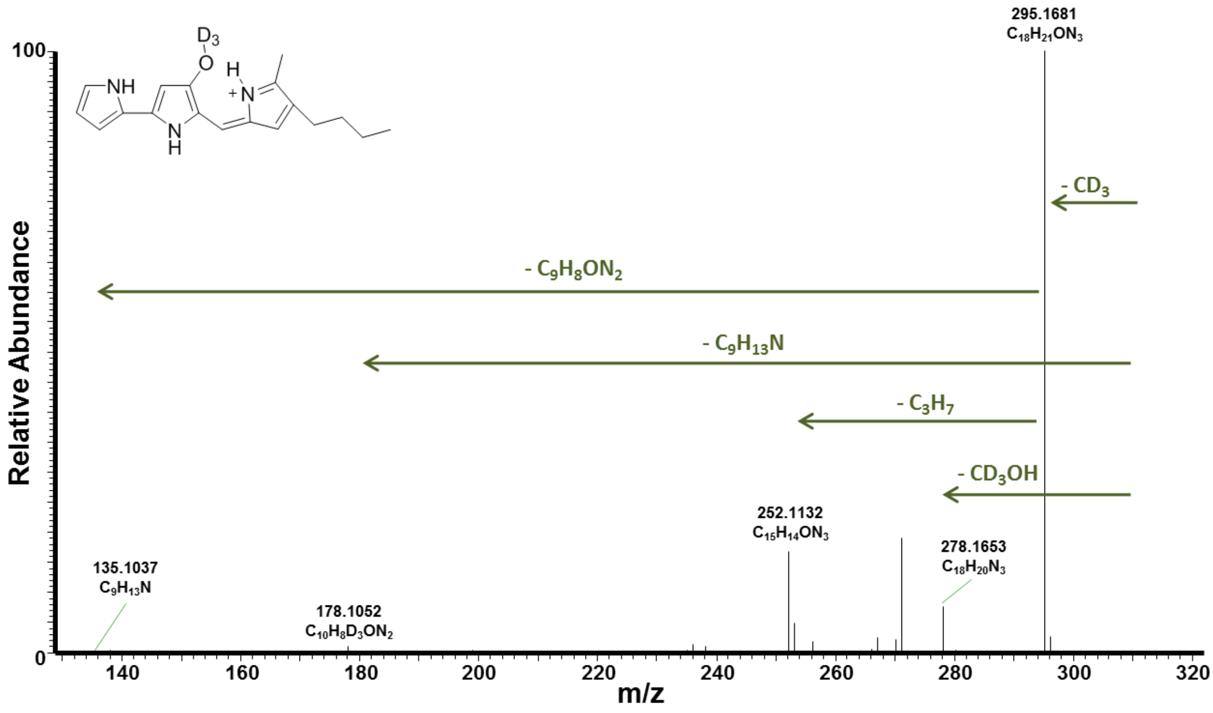

Fig. S137. HRMS[2] of prodigiosin with incorporated methyl-D_3 group derived from [methyl-D_3]-L-methionine (compound **3** $[M+H]^+$: *m/z* 327, $C_{20}H_{23}D_3N_3O$).

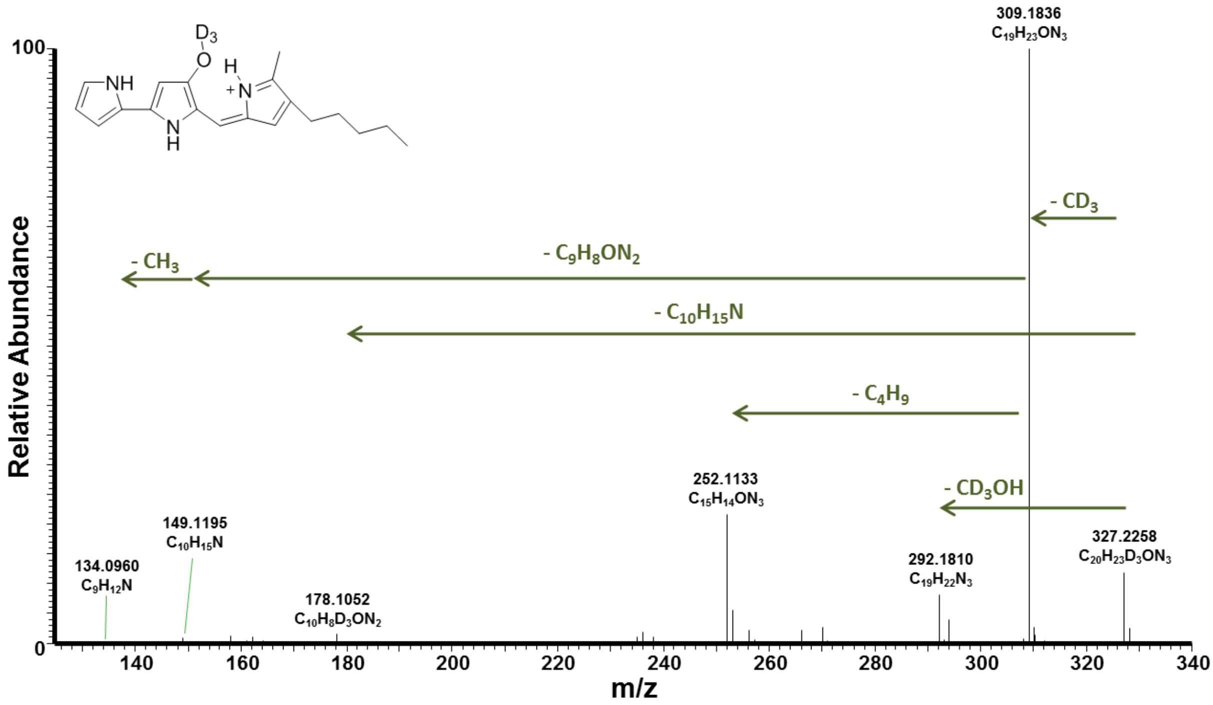

Fig. S138. HRMS[2] of 2-methyl-3-hexyl prodiginine with incorporated methyl-D_3 group derived from [methyl-D_3]-L-methionine (compound **4** $[M+H]^+$: *m/z* 341, $C_{21}H_{25}D_3N_3O$).

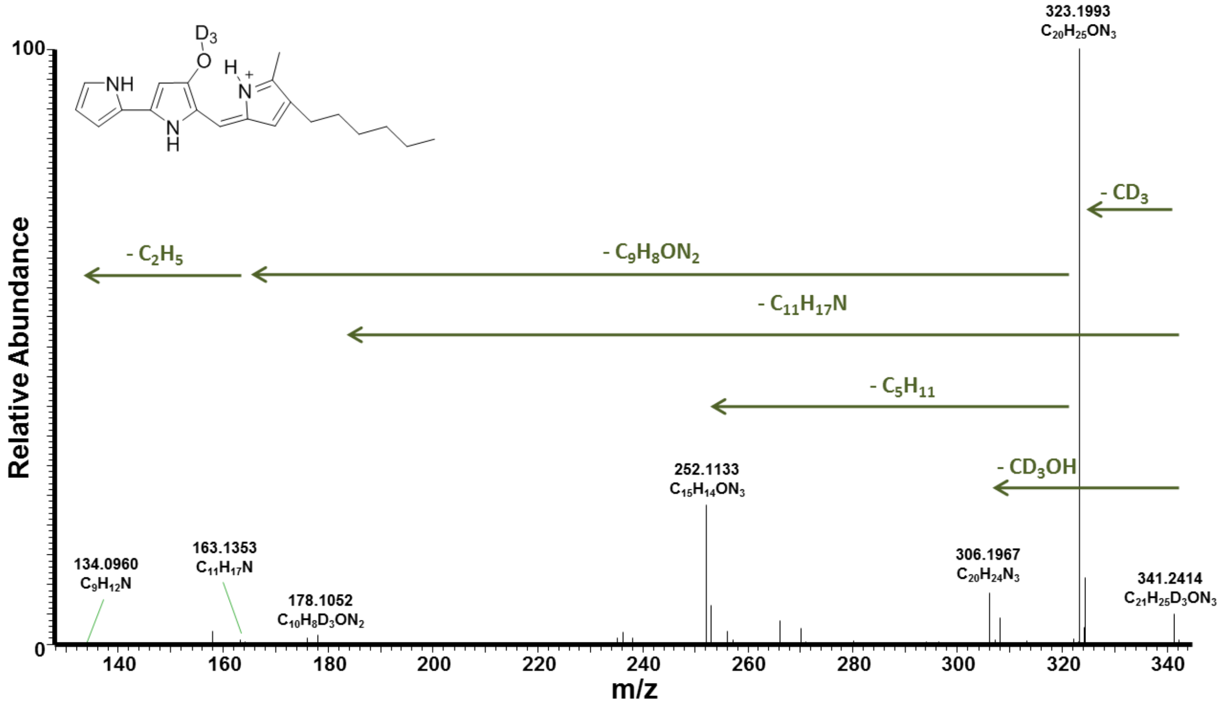

Fig. S139. HRMS[2] of 2-methyl-3-heptyl prodiginine with incorporated methyl-D3 group derived from [methyl-D3]-L-methionine (compound **5** $[M+H]^+$: *m/z* 355, $C_{22}H_{27}D_3N_3O$).

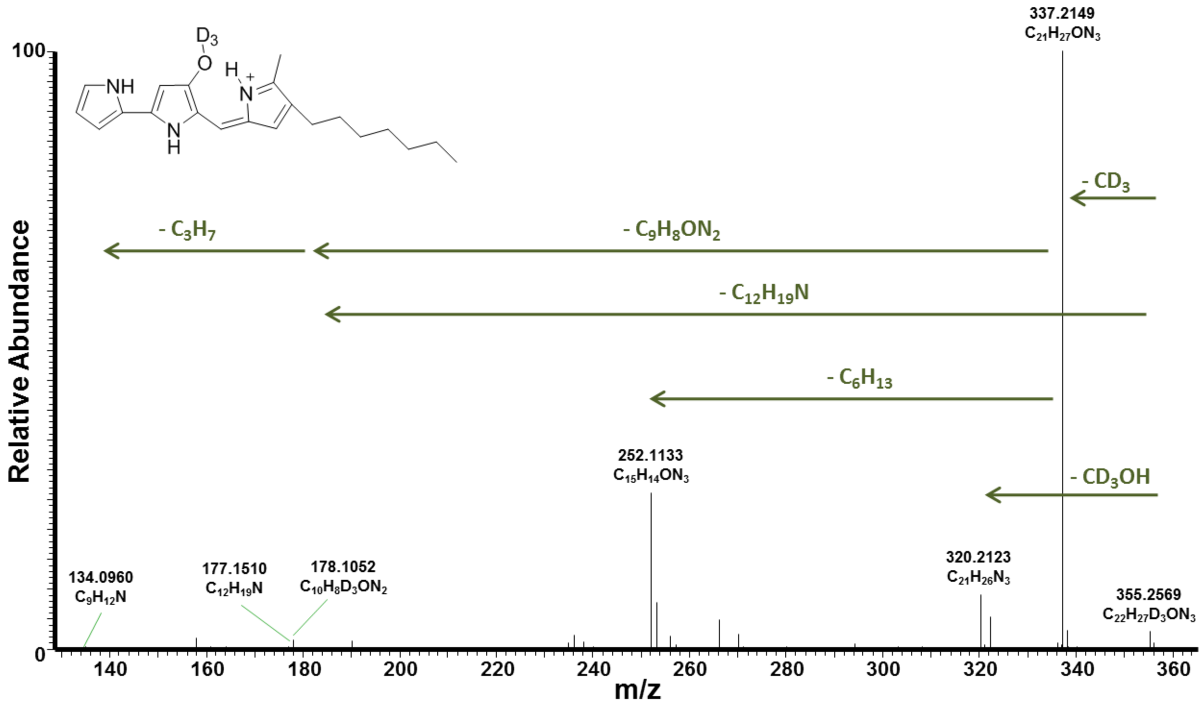

Fig. S140. HRMS[2] of 2-methyl-3-nonyl prodiginine with incorporated methyl-D3 group derived from [methyl-D3]-L-methionine (compound **7** $[M+H]^+$: *m/z* 383, $C_{24}H_{31}D_3N_3O$).

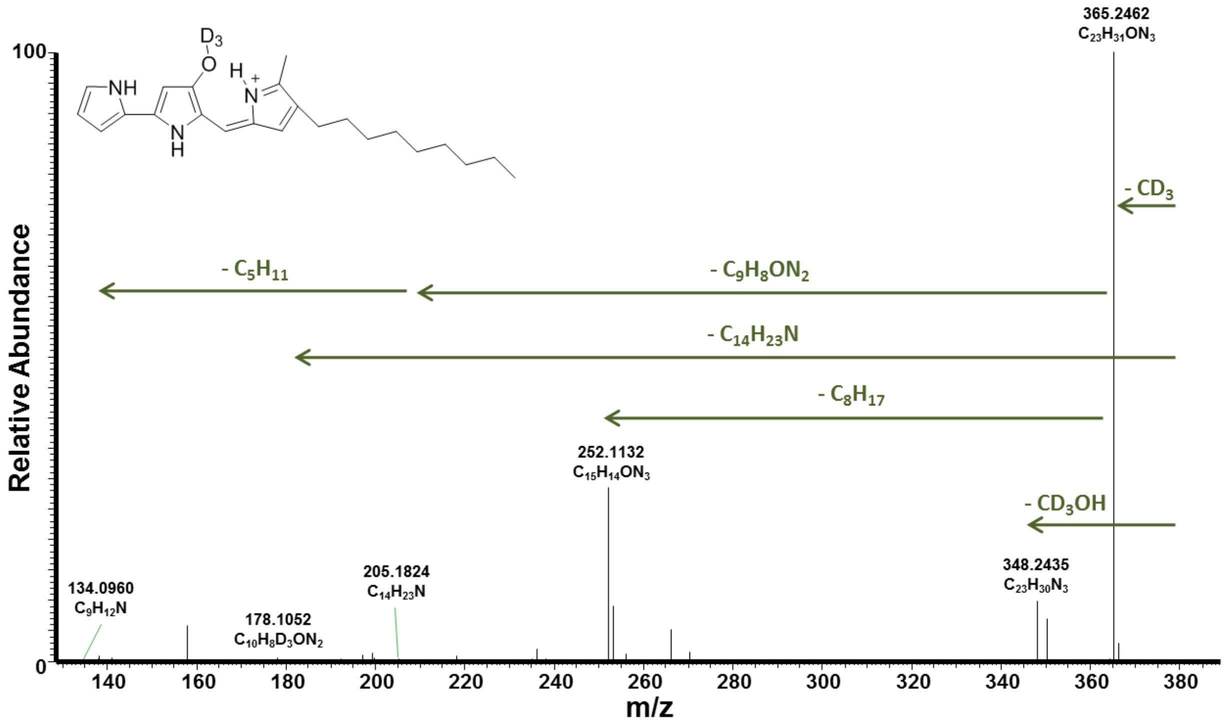

Fig. S141. HRMS[3] of 2-methyl-3-propyl prodiginine with incorporated methyl-D3 group derived from [methyl-D3]-L-methionine (compound **1** $[M+H]^+$: *m/z* 299 via 281, $C_{18}H_{19}D_3N_3O$).

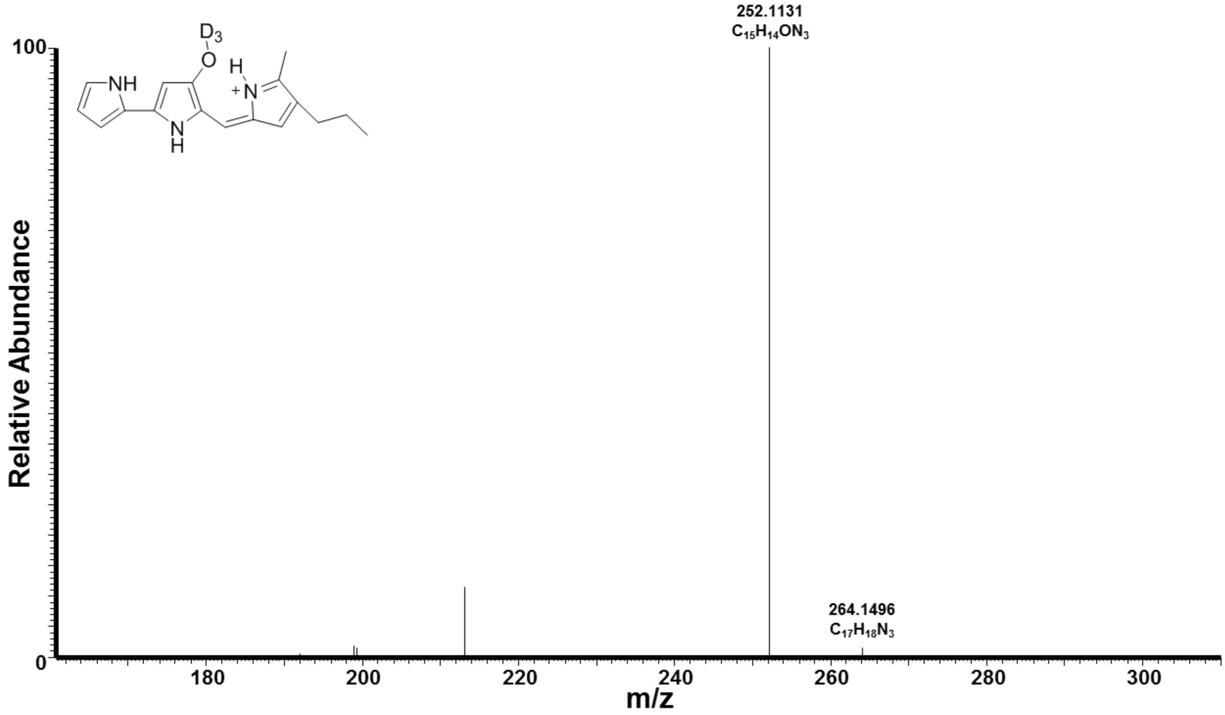

Fig. S142. HRMS[3] of 2-methyl-3-butyl prodiginine with incorporated methyl-D3 group derived from [methyl-D3]-L-methionine (compound **2** $[M+H]^+$: *m/z* 313 via 295, $C_{19}H_{21}D_3N_3O$).

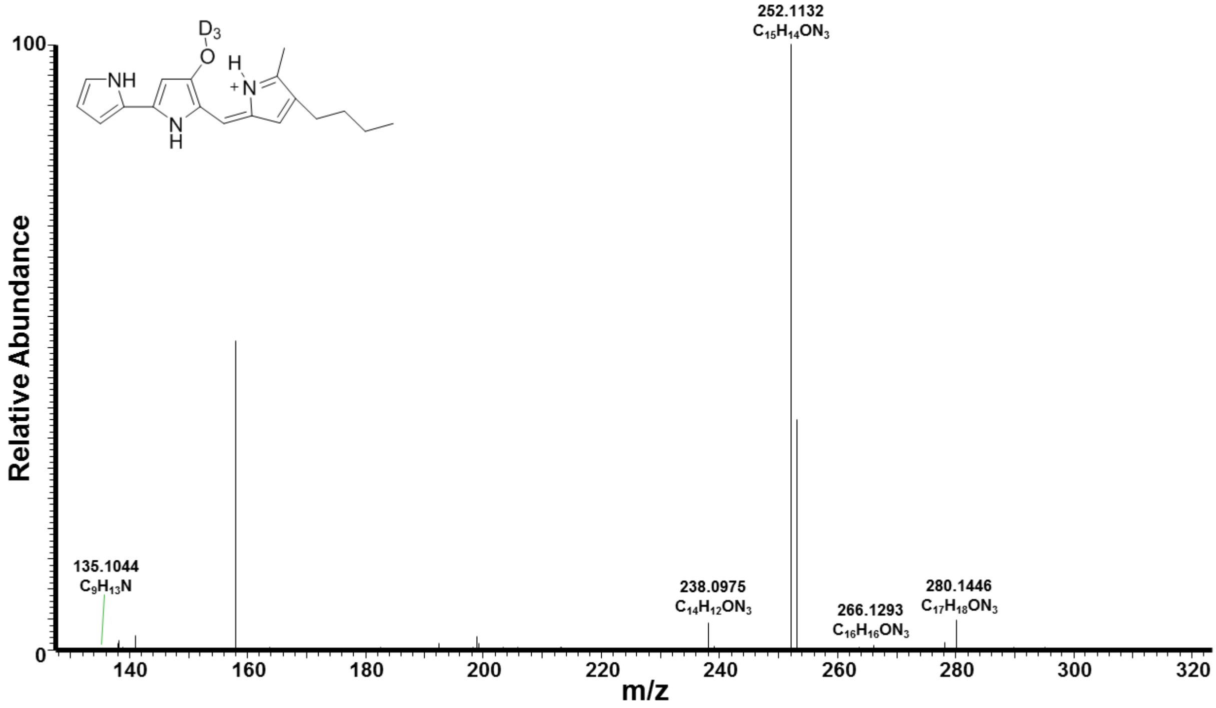

Fig. S143. HRMS[3] of prodigiosin with incorporated methyl-D_3 group derived from [methyl-D_3]-L-methionine (compound **3** $[M+H]^+$: *m/z* 327 via 309, $C_{20}H_{23}D_3N_3O$).

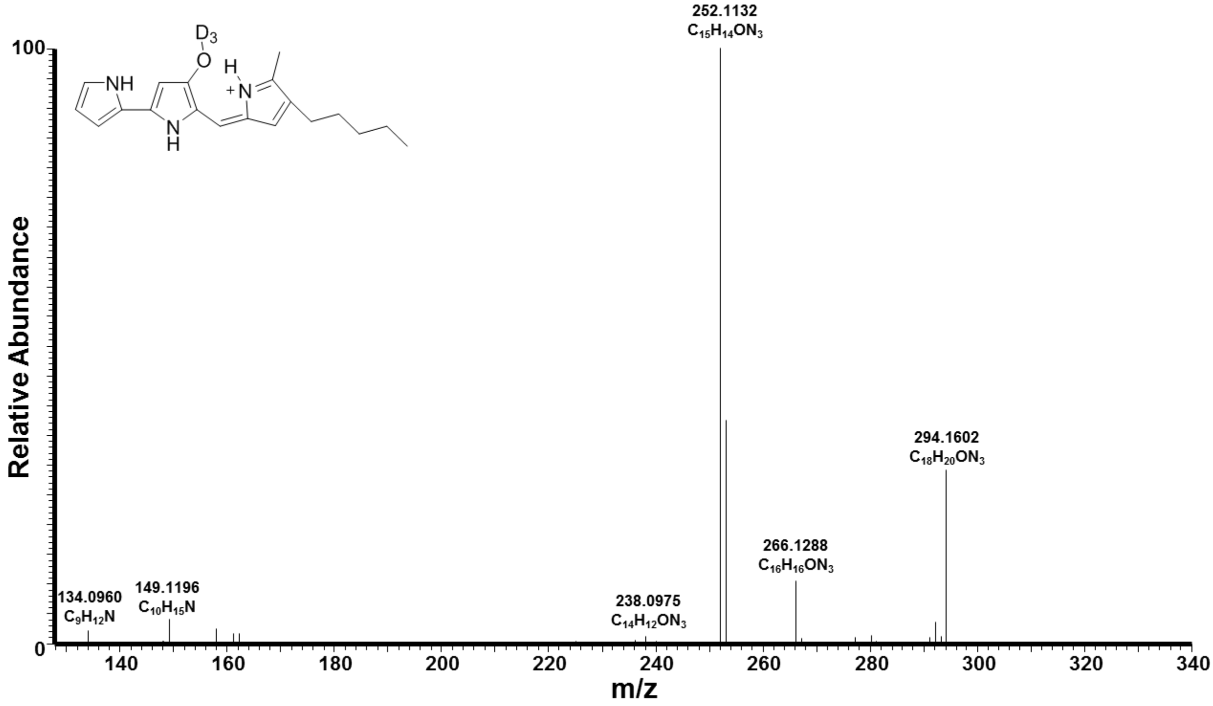

Fig. S144. HRMS[3] of 2-methyl-3-hexyl prodiginine with incorporated methyl-D_3 group derived from [methyl-D_3]-L-methionine (compound **4** $[M+H]^+$: *m/z* 341 via 323, $C_{21}H_{25}D_3N_3O$).

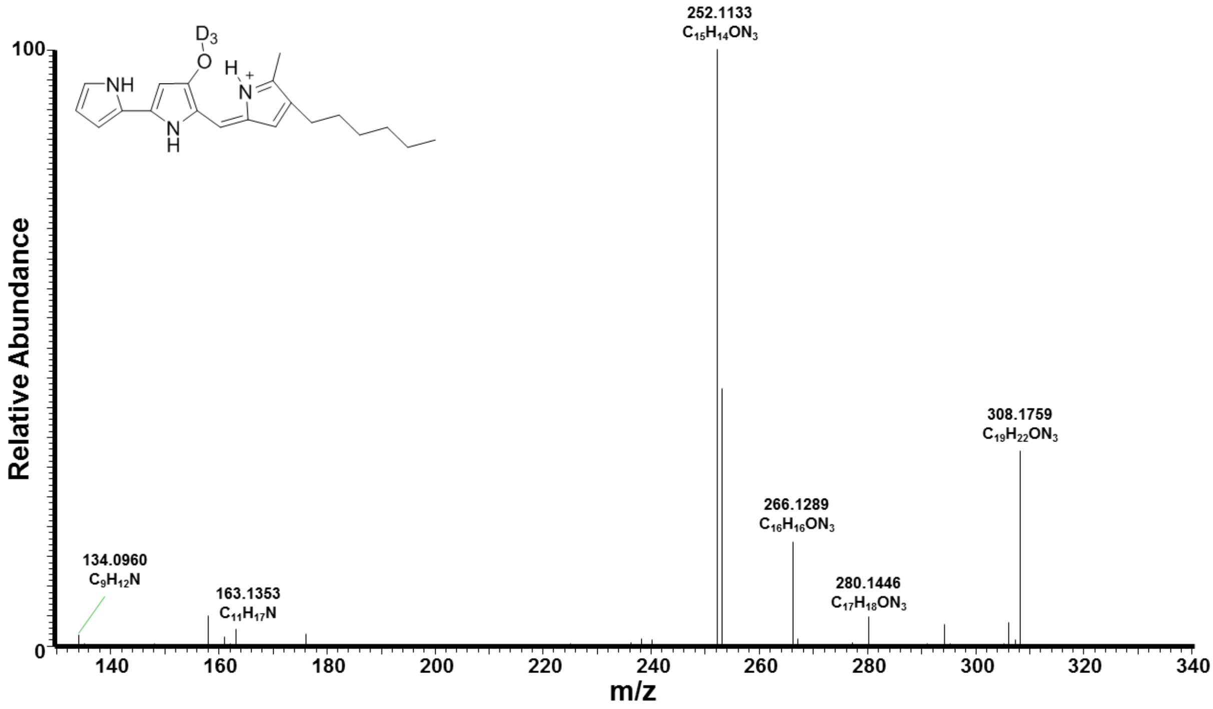

Fig. S145. HRMS[3] of 2-methyl-3-heptyl prodiginine with incorporated methyl-D3 group derived from [methyl-D3]-L-methionine (compound **5** $[M+H]^+$: *m/z* 355 via 337, $C_{22}H_{27}D_3N_3O$).

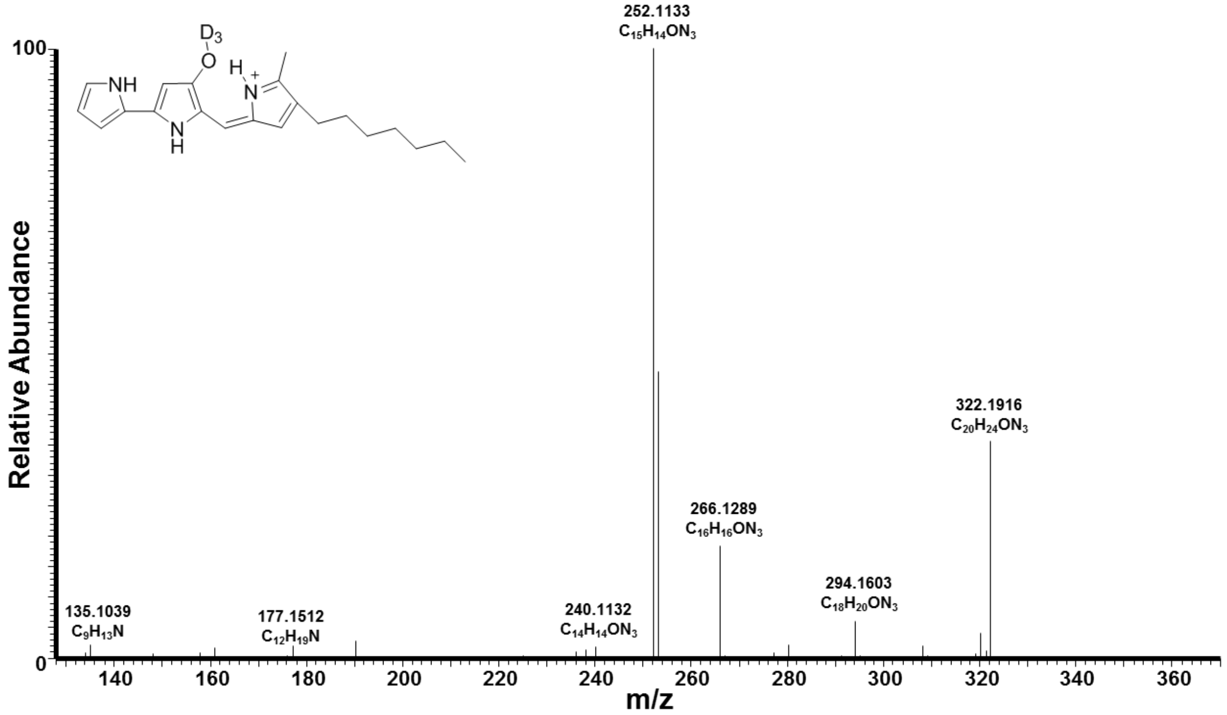

Fig. S146. HRMS[3] of 2-methyl-3-nonyl prodiginine with incorporated methyl-D3 group derived from [methyl-D3]-L-methionine (compound **7** $[M+H]^+$: *m/z* 383 via 366, $C_{24}H_{31}D_3N_3O$).

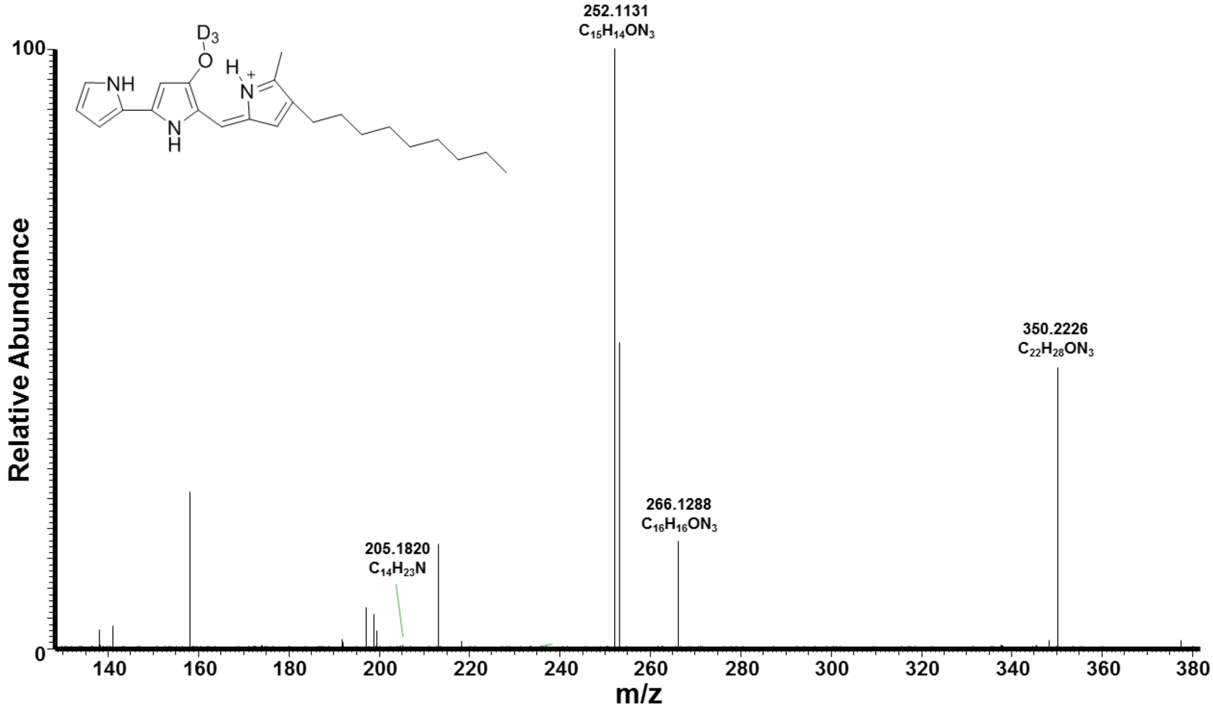

Fig. S147. Proposed mass spectral fragmentation pathway of 2-methyl-3-propyl prodiginine with incorporated methyl-D_3 group derived from [methyl-D_3]-L-methionine (compound **1** $[M+H]^+$: *m/z* 299, $C_{18}H_{19}D_3N_3O$).

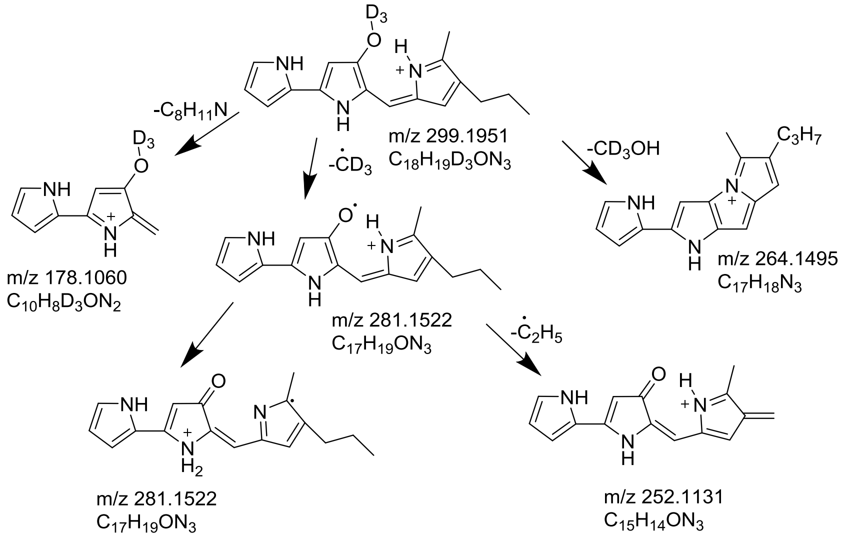

Fig. S148. Proposed mass spectral fragmentation pathway of 2-methyl-3-butyl prodiginine with incorporated methyl-D_3 group derived from [methyl-D_3]-L-methionine (compound **2** $[M+H]^+$: *m/z* 313, $C_{19}H_{21}D_3N_3O$).

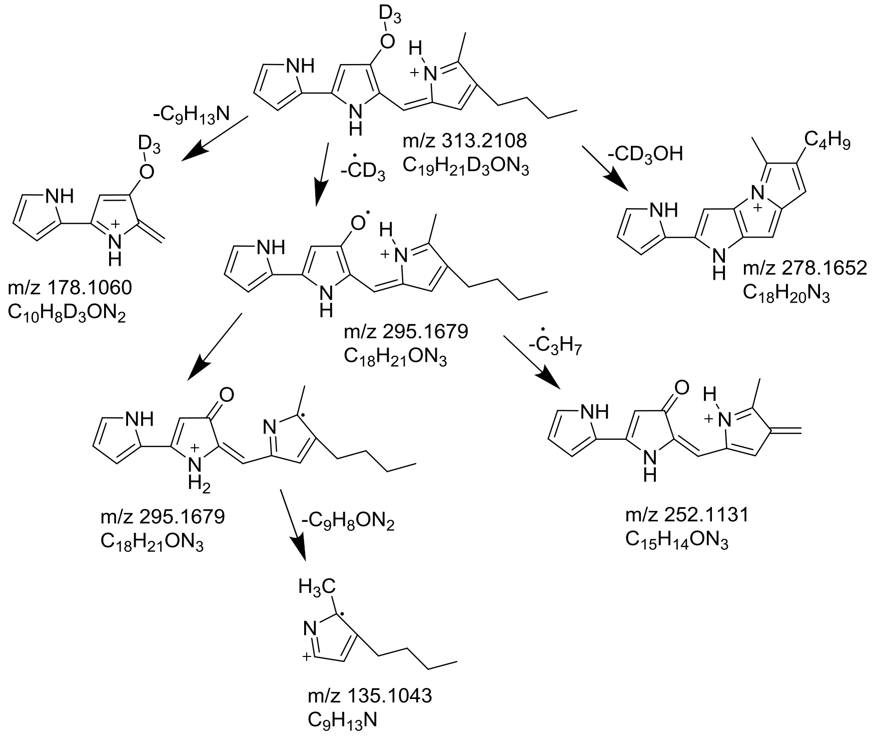

Fig. S149. Proposed mass spectral fragmentation pathway of prodigiosin with incorporated methyl-D_3 group derived from [methyl-D_3]-L-methionine (compound **3** $[M+H]^+$: *m/z* 327, $C_{20}H_{23}D_3N_3O$).

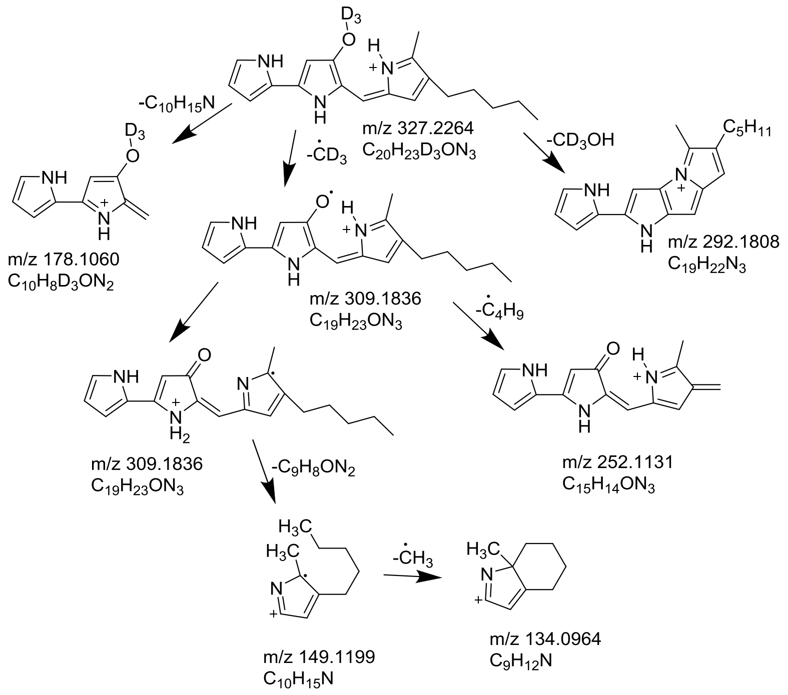

Fig. S150. Proposed mass spectral fragmentation pathway of 2-methyl-3-hexyl prodiginine with incorporated methyl-D_3 group derived from [methyl-D_3]-L-methionine (compound **4** $[M+H]^+$: *m/z* 341, $C_{21}H_{25}D_3N_3O$).

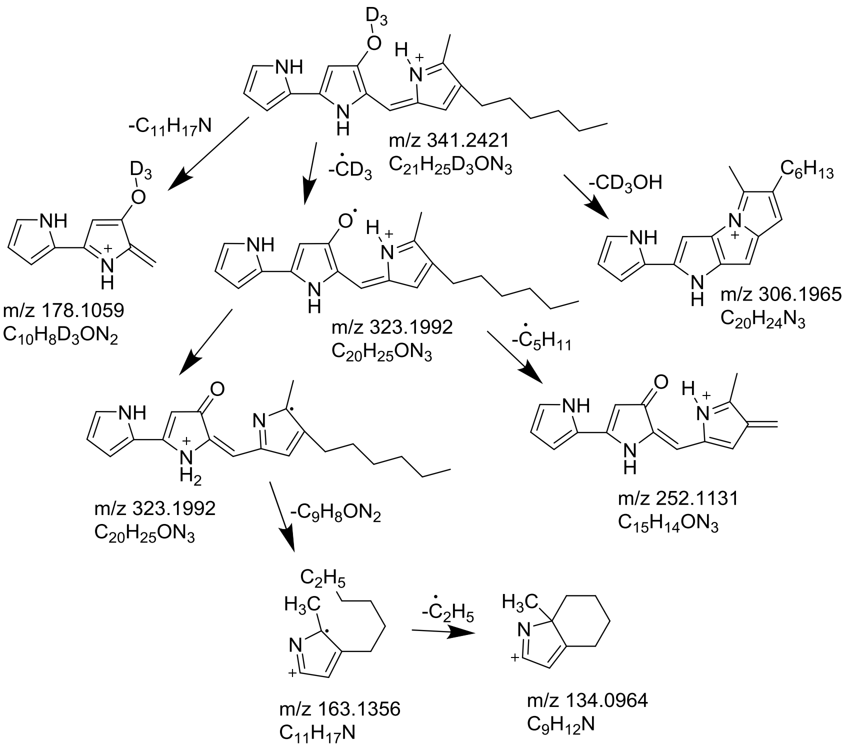

Fig. S151. Proposed mass spectral fragmentation pathway of 2-methyl-3-heptyl prodiginine with incorporated methyl-D_3 group derived from [methyl-D_3]-L-methionine (compound **5** $[M+H]^+$: *m/z* 355, $C_{22}H_{27}D_3N_3O$).

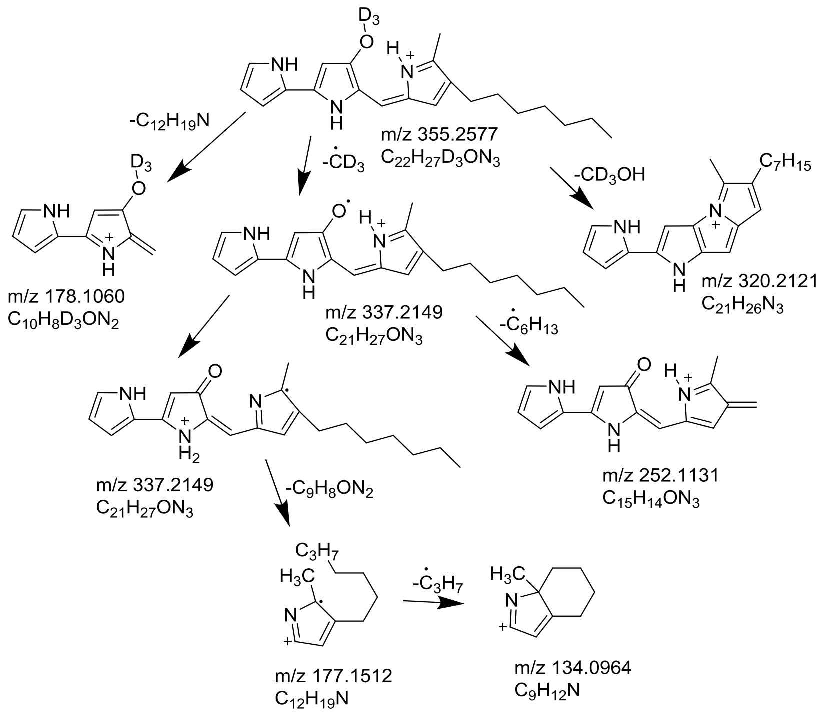

Fig. S152. Proposed mass spectral fragmentation pathway of 2-methyl-3-nonyl prodiginine with incorporated methyl-D_3 group derived from [methyl-D_3]-L-methionine (compound **7** $[M+H]^+$: *m/z* 383, $C_{24}H_{31}D_3N_3O$).

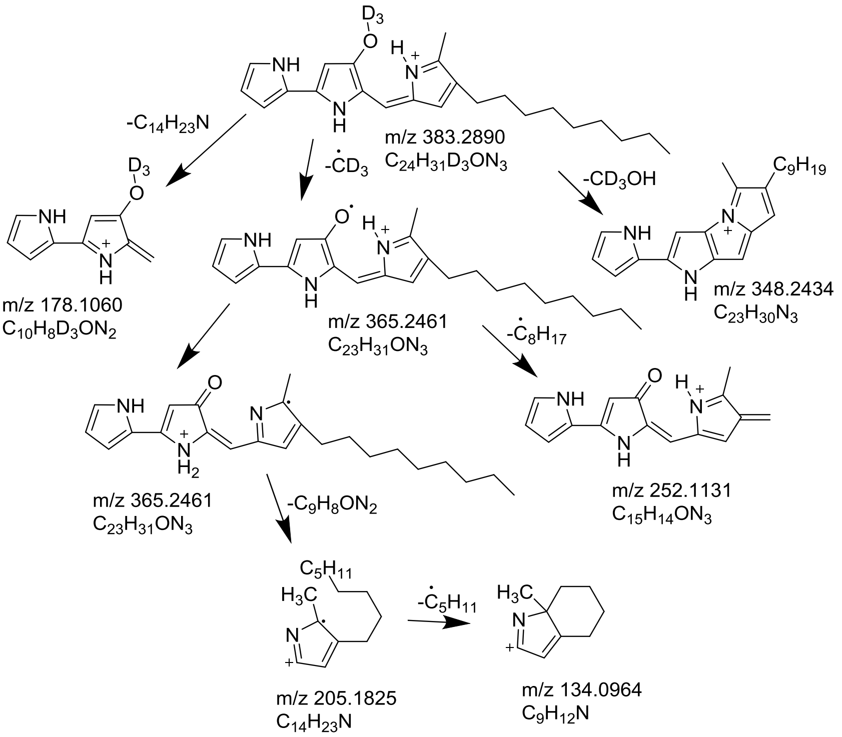

Fig. S153. HRMS[2] of 2-methyl-3-propyl prodiginine with incorporated [1-^{13}C]-L-proline (compound **1** $[M+H]^+$: *m/z* 297, $C_{17}{}^{13}CH_{22}N_3O$).

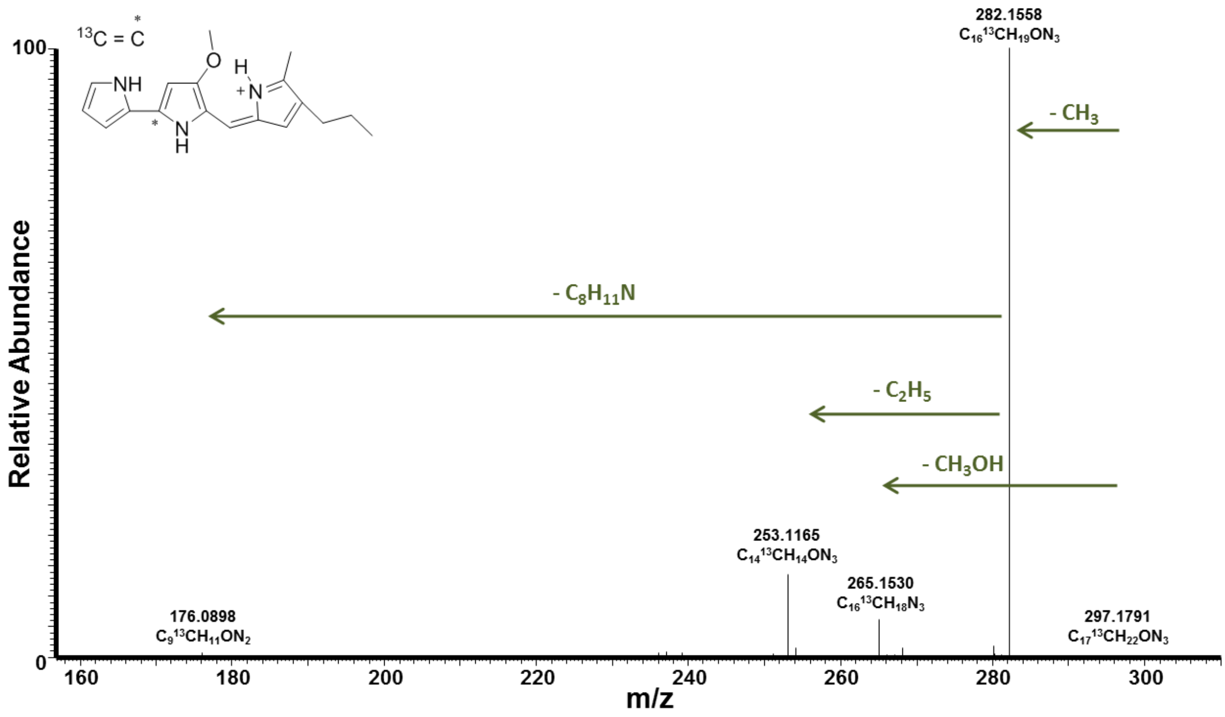

Fig. S154. HRMS[2] of 2-methyl-3-butyl prodiginine with incorporated [1-^{13}C]-L-proline (compound **2** $[M+H]^+$: *m/z* 311, $C_{18}{}^{13}CH_{24}N_3O$).

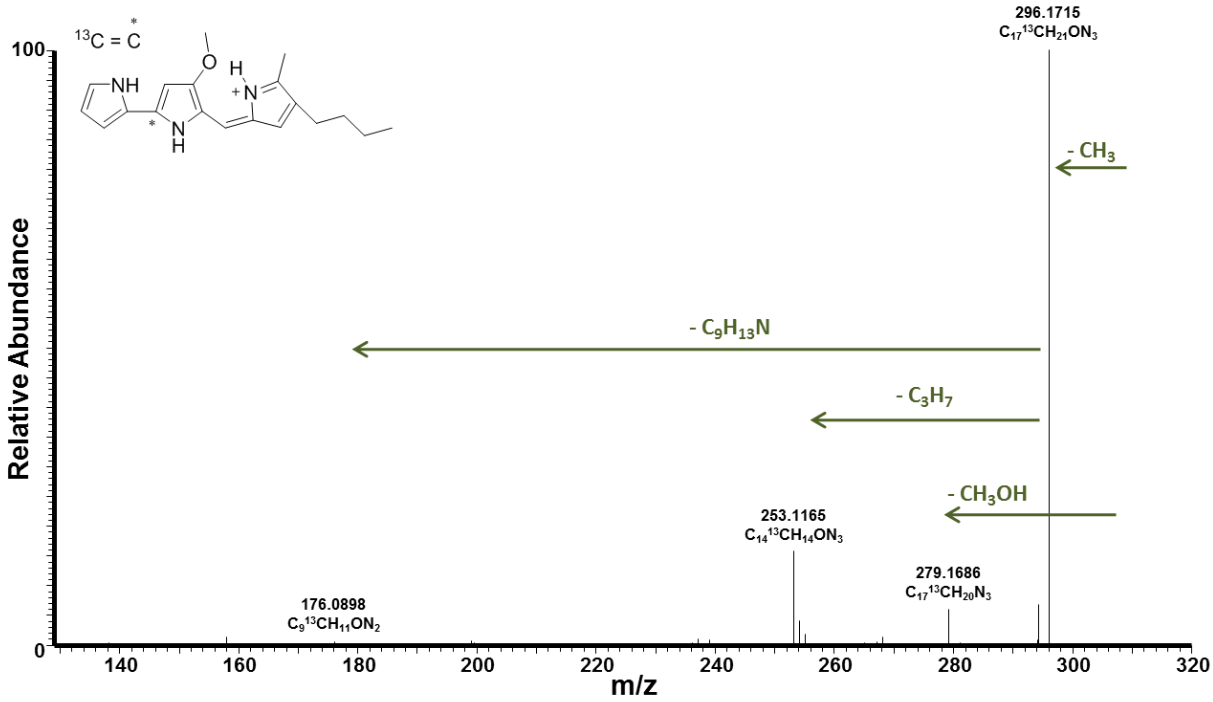

Fig. S155. HRMS^2 of prodigiosin with incorporated [1-^{13}C]-L-proline (compound **3** $[M+H]^+$: *m/z* 325, $C_{19}{}^{13}CH_{26}N_3O$).

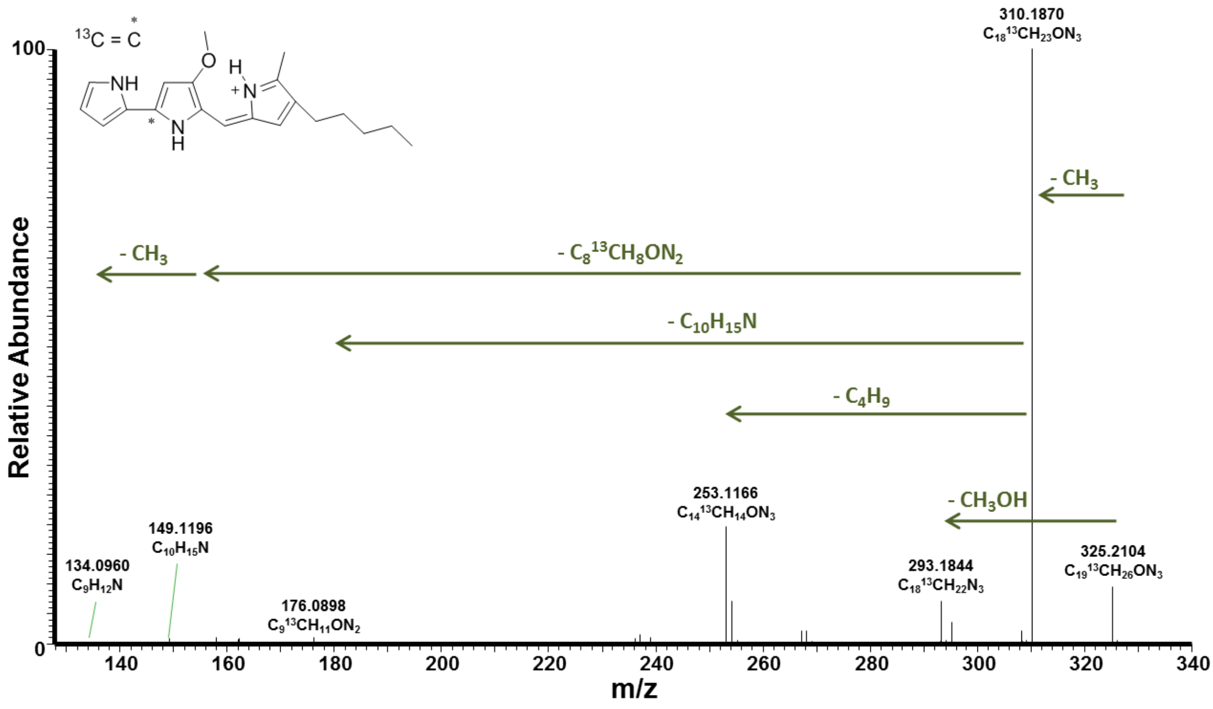

Fig. S156. HRMS^2 of 2-methyl-3-hexyl prodiginine with incorporated [1-^{13}C]-L-proline (compound **4** $[M+H]^+$: *m/z* 339, $C_{20}{}^{13}CH_{28}N_3O$).

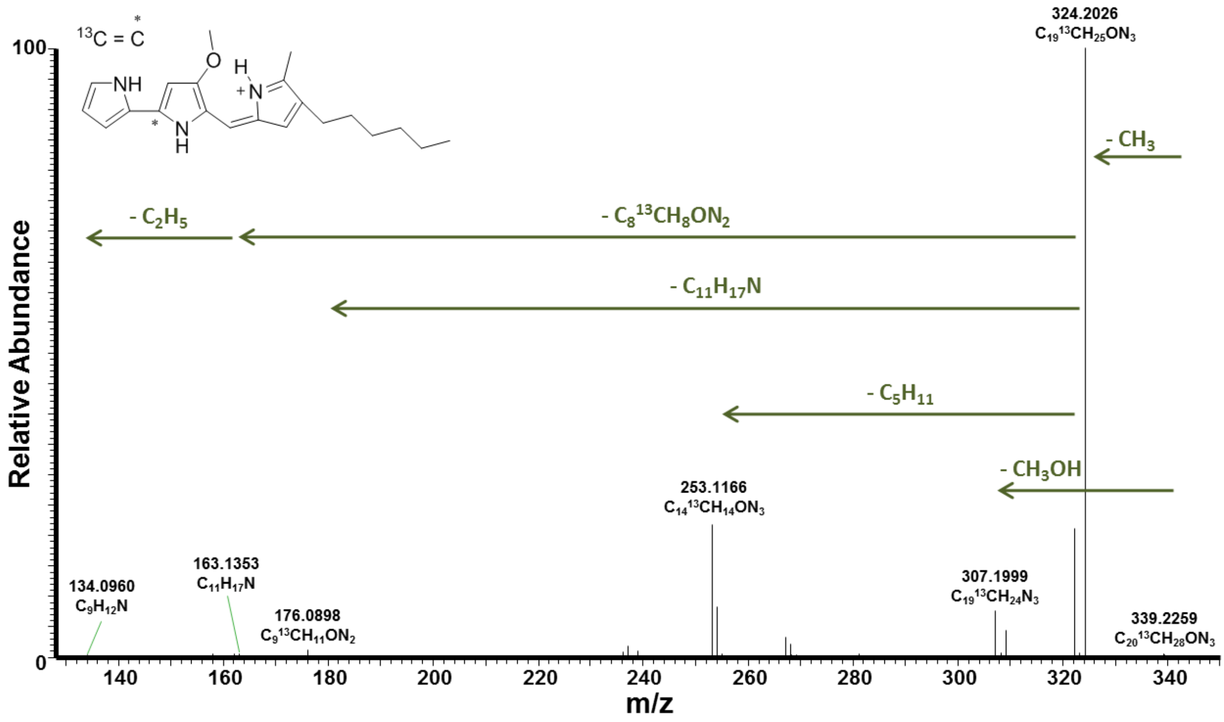

Fig. S157. HRMS^2 of 2-methyl-3-heptyl prodiginine with incorporated [1-^{13}C]-L-proline (compound **5** $[M+H]^+$: *m/z* 353, $C_{21}{}^{13}CH_{30}N_3O$).

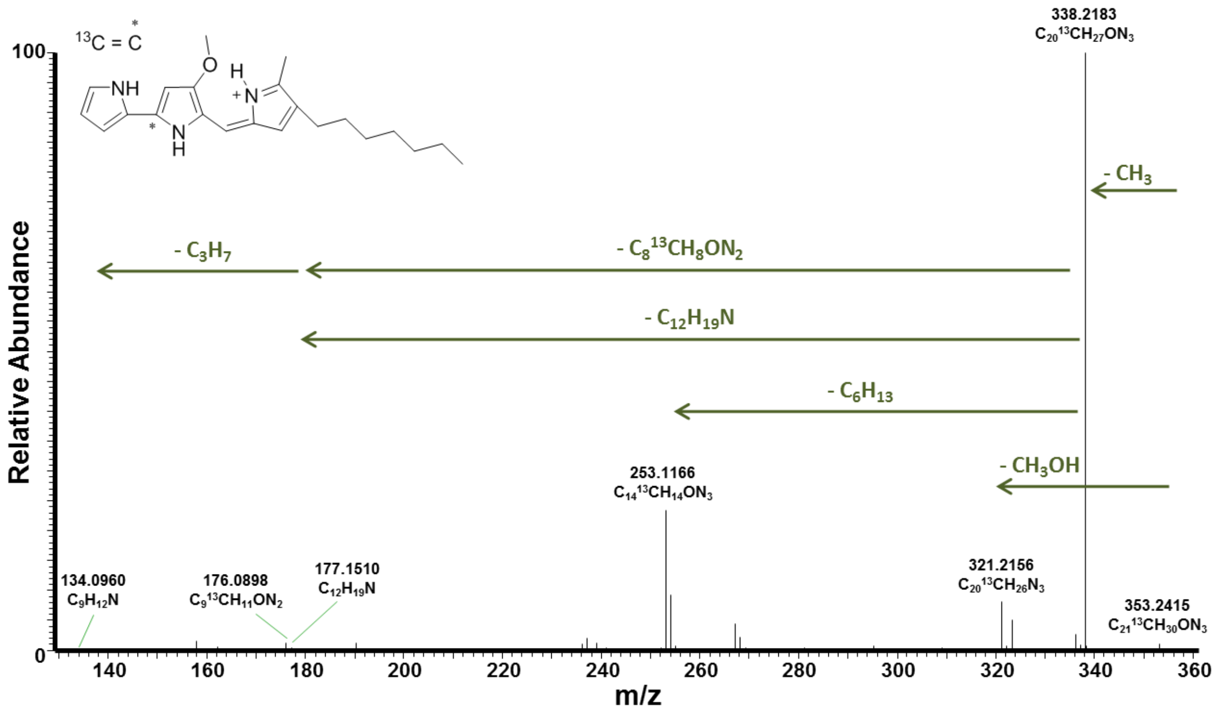

Fig. S158. HRMS^2 of 2-methyl-3-nonyl prodiginine with incorporated [1-^{13}C]-L-proline (compound **7** $[M+H]^+$: *m/z* 381, $C_{23}{}^{13}CH_{34}N_3O$).

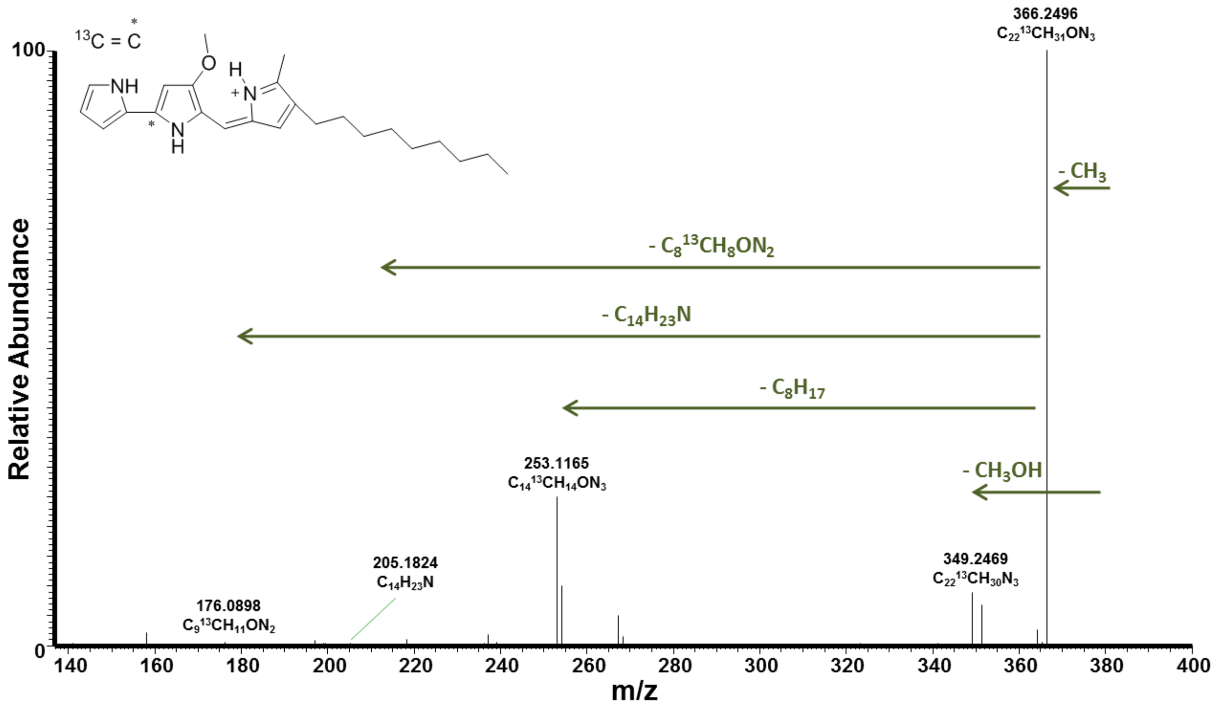

Fig. S159. HRMS[3] of 2-methyl-3-propyl prodiginine with incorporated [1-^{13}C]-L-proline (compound **1** $[M+H]^+$: *m/z* 297 via 282, $C_{17}{}^{13}CH_{22}N_3O$).

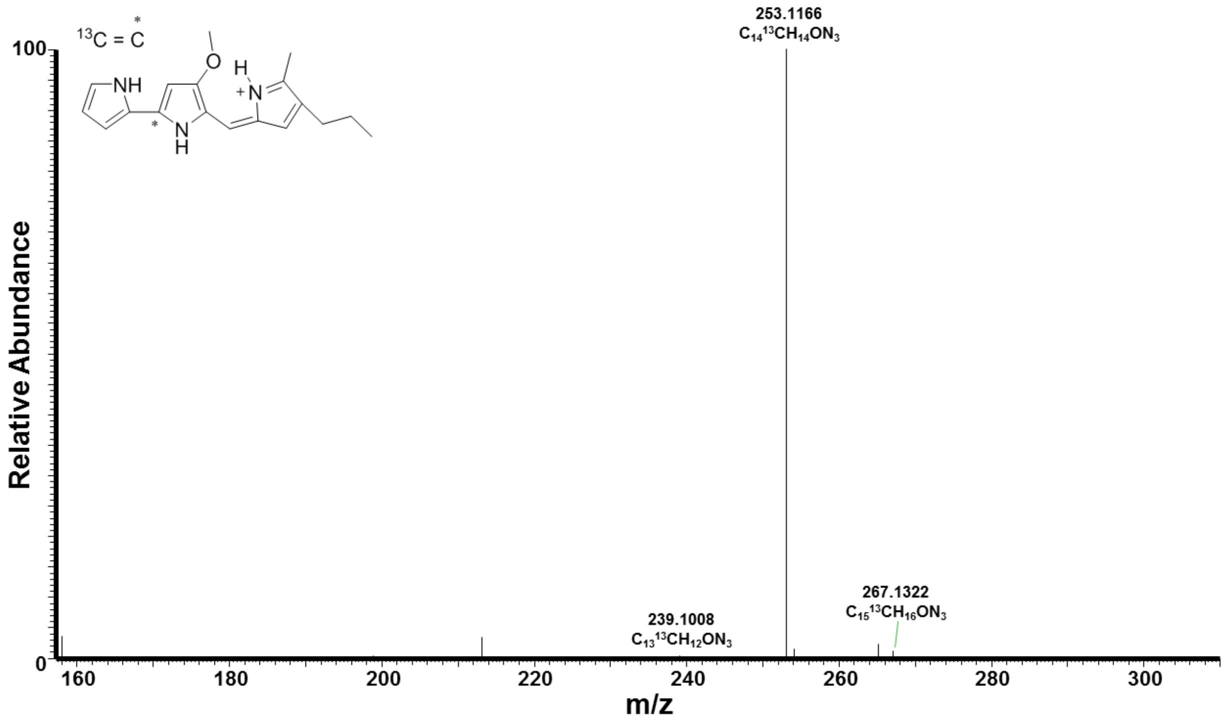

Fig. S160. HRMS[3] of 2-methyl-3-butyl prodiginine with incorporated [1-^{13}C]-L-proline (compound **2** $[M+H]^+$: *m/z* 311 via 296, $C_{18}{}^{13}CH_{24}N_3O$).

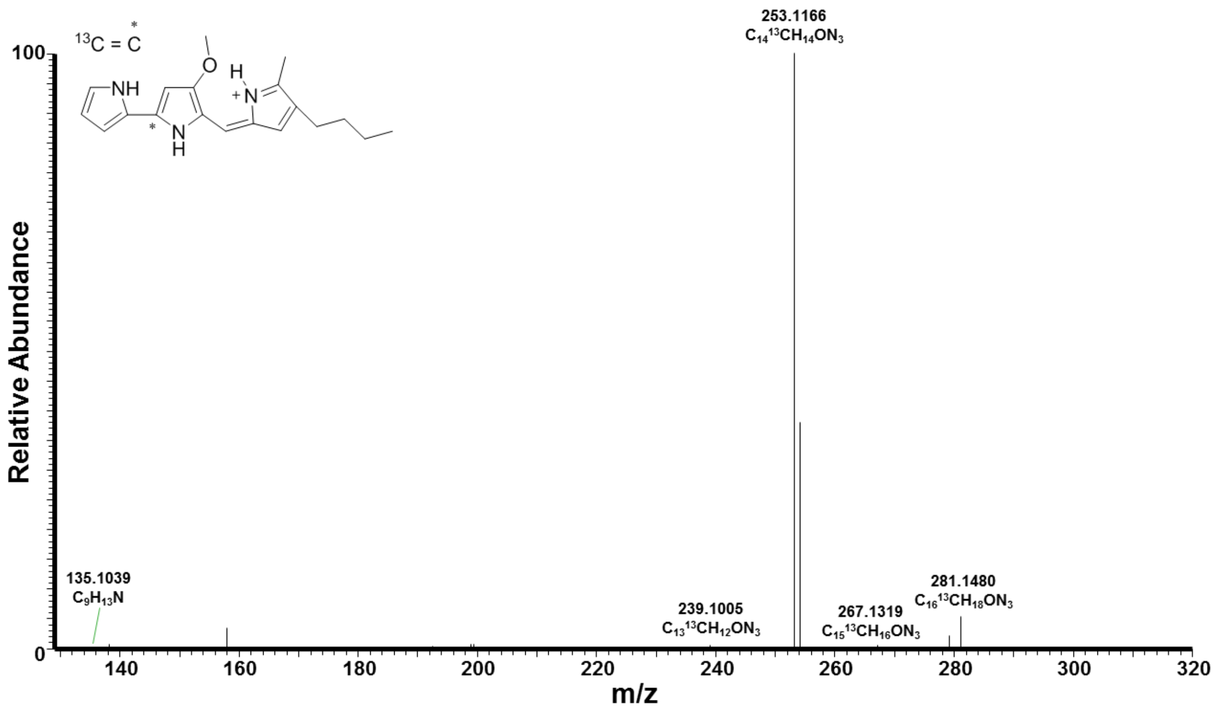

Fig. S161. HRMS[3] of prodigiosin with incorporated [1-^{13}C]-L-proline (compound **3** $[M+H]^+$: *m/z* 325 via 310, $C_{19}{}^{13}CH_{26}N_3O$).

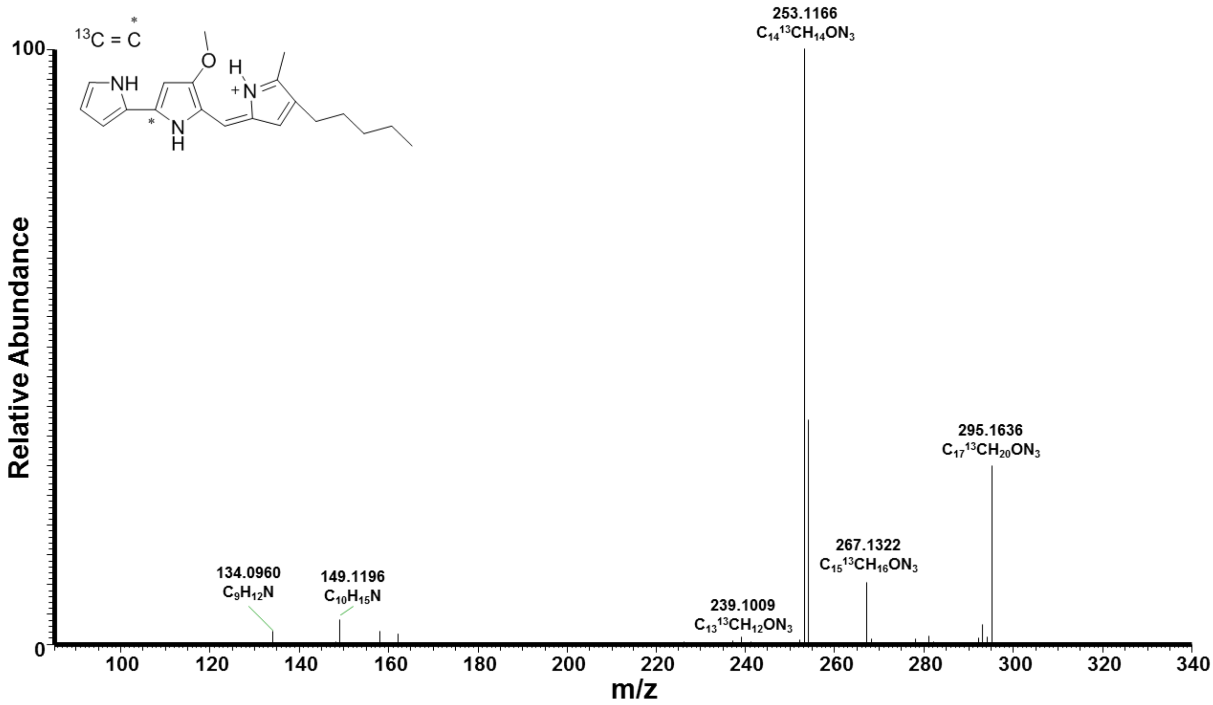

Fig. S162. HRMS[3] of 2-methyl-3-hexyl prodiginine with incorporated [1-^{13}C]-L-proline (compound **4** $[M+H]^+$: *m/z* 339 via 324, $C_{20}{}^{13}CH_{28}N_3O$).

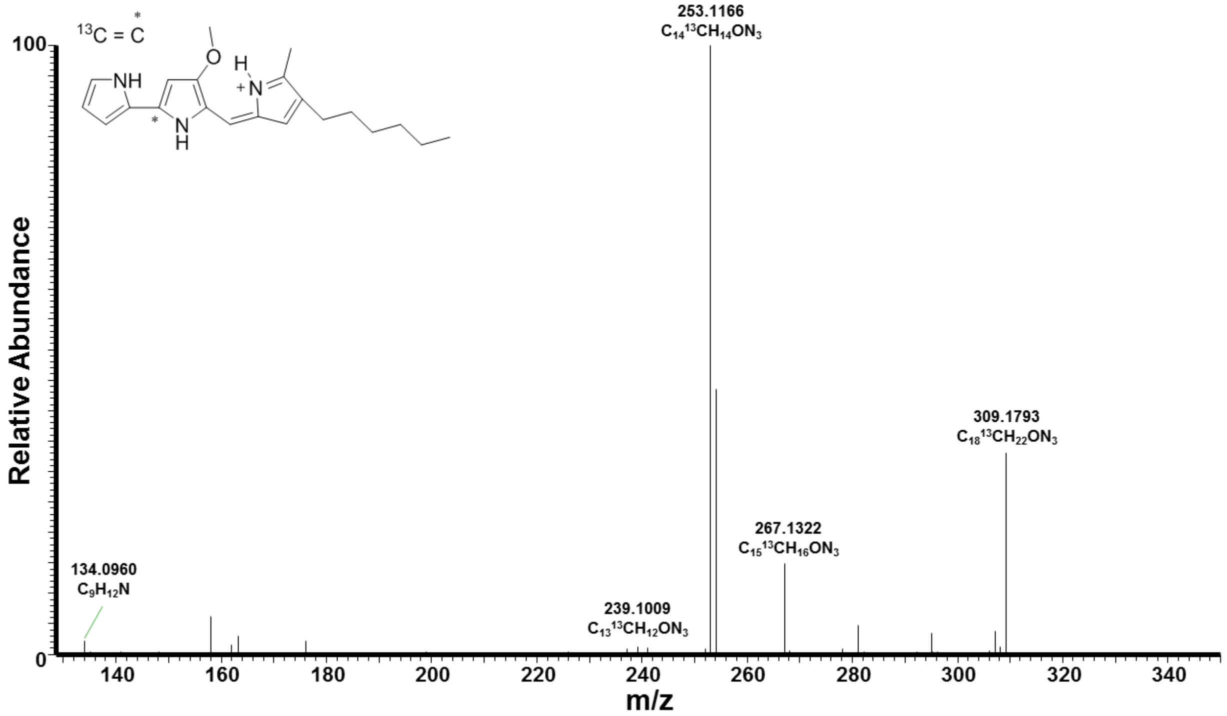

Fig. S163. HRMS[3] of 2-methyl-3-heptyl prodiginine with incorporated [1-^{13}C]-L-proline (compound **5** $[M+H]^+$: *m/z* 353 via 338, $C_{21}{}^{13}CH_{30}N_3O$).

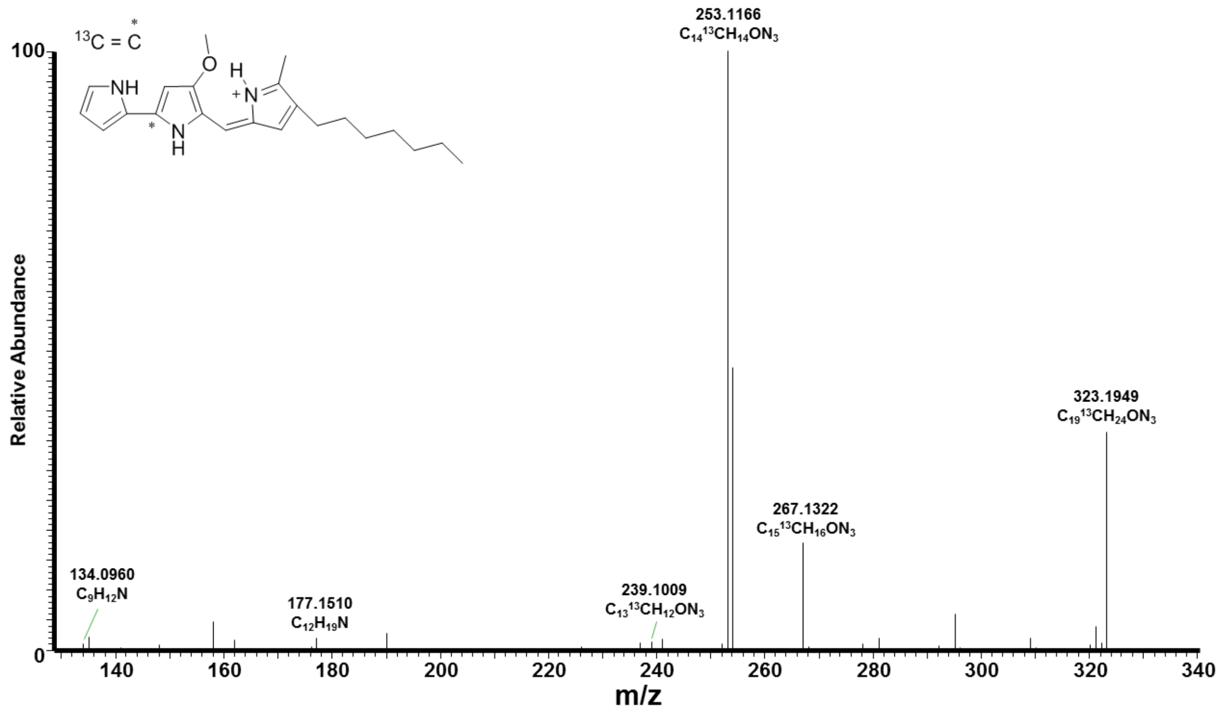

Fig. S164. HRMS[3] of 2-methyl-3-nonyl prodiginine with incorporated [1-^{13}C]-L-proline (compound **7** $[M+H]^+$: *m/z* 381 via 366, $C_{23}{}^{13}CH_{34}N_3O$).

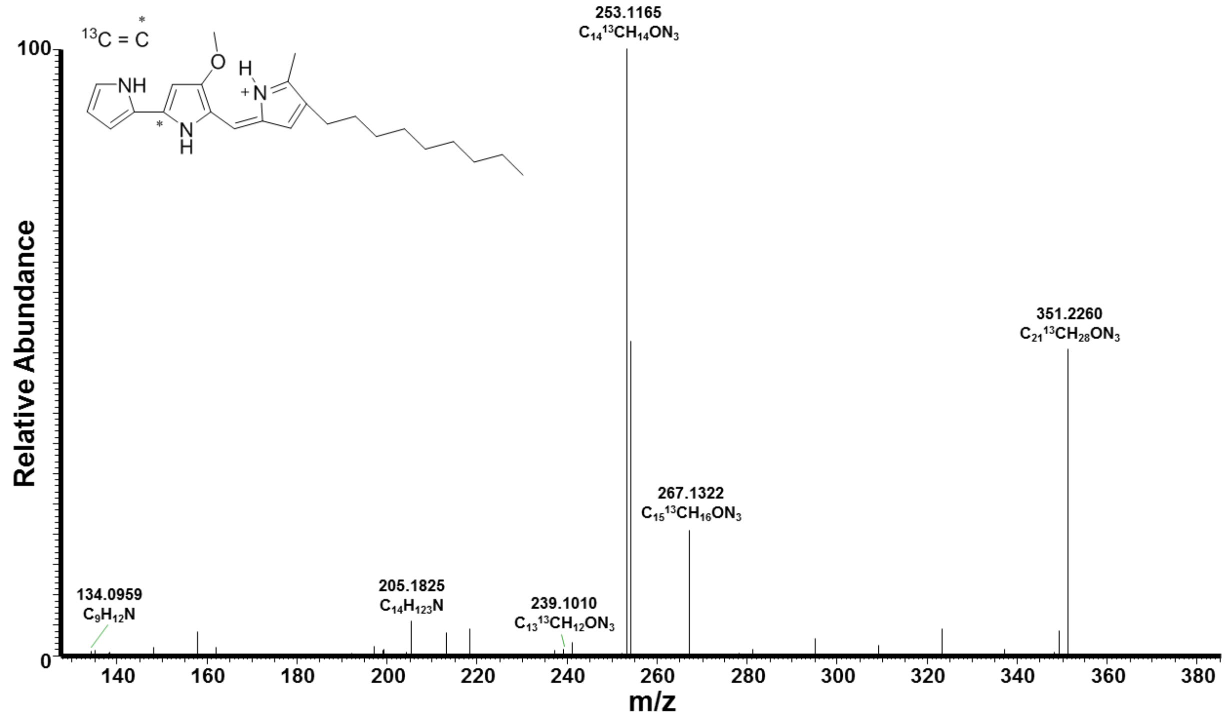

Fig. S165. Proposed mass spectral fragmentation pathway of 2-methyl-3-propyl prodiginine with incorporated [1-^{13}C]-L-proline (compound **1** $[M+H]^+$: *m/z* 297, $C_{17}{}^{13}CH_{22}N_3O$).

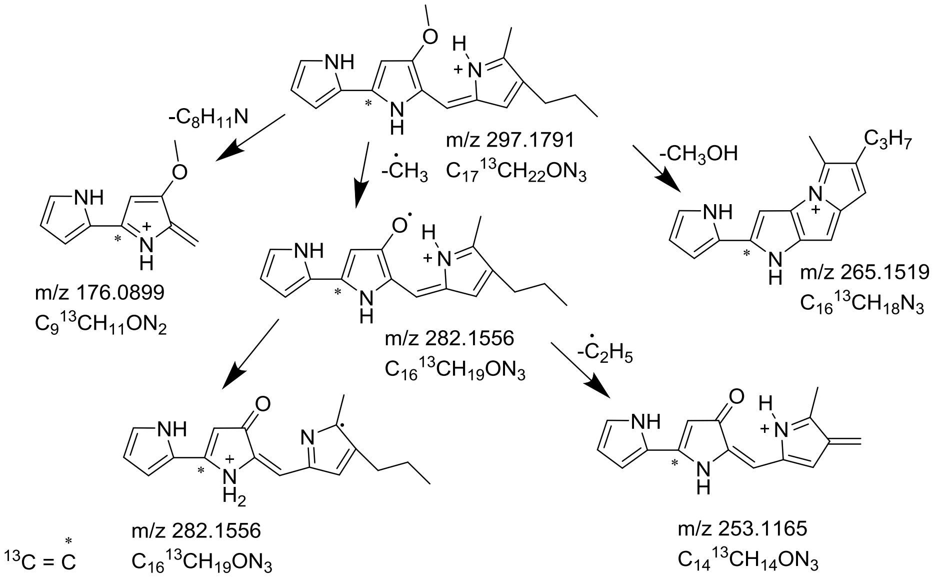

Fig. S166. Proposed mass spectral fragmentation pathway of 2-methyl-3-butyl prodiginine with incorporated [1-^{13}C]-L-proline (compound **2** $[M+H]^+$: *m/z* 311, $C_{18}{}^{13}CH_{24}N_3O$).

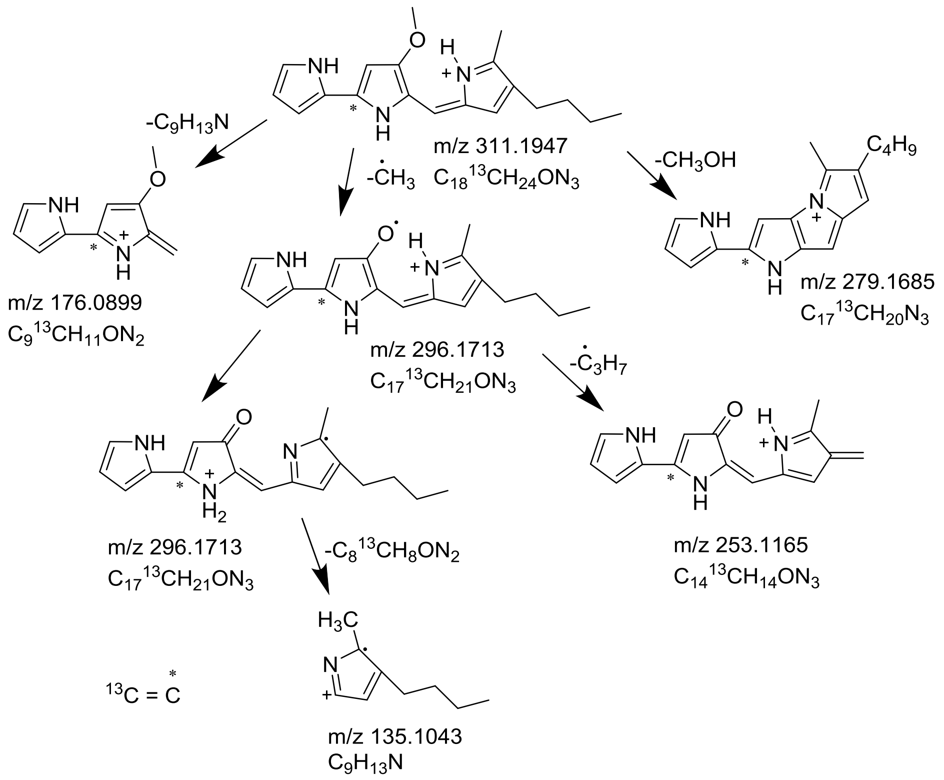

Fig. S167. Proposed mass spectral fragmentation pathway of prodigiosin with incorporated [1-^{13}C]-L-proline (compound **3** $[M+H]^+$: *m/z* 325, $C_{19}{}^{13}CH_{26}N_3O$).

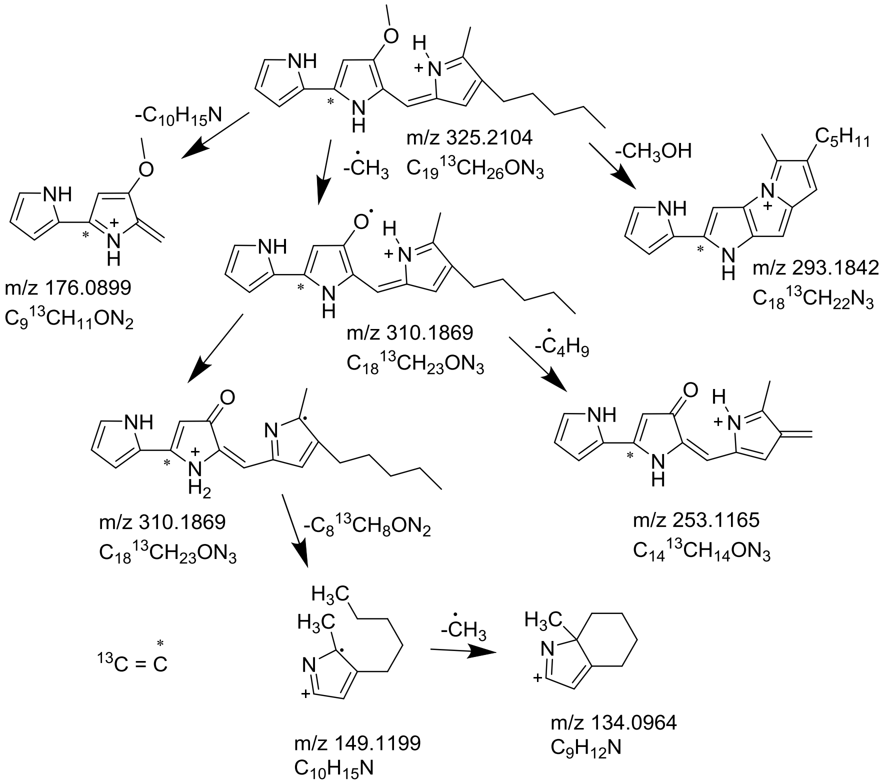

Fig. S168. Proposed mass spectral fragmentation pathway of 2-methyl-3-hexyl prodiginine with incorporated [1-^{13}C]-L-proline (compound **4** $[M+H]^+$: *m/z* 339, $C_{20}{}^{13}CH_{28}N_3O$).

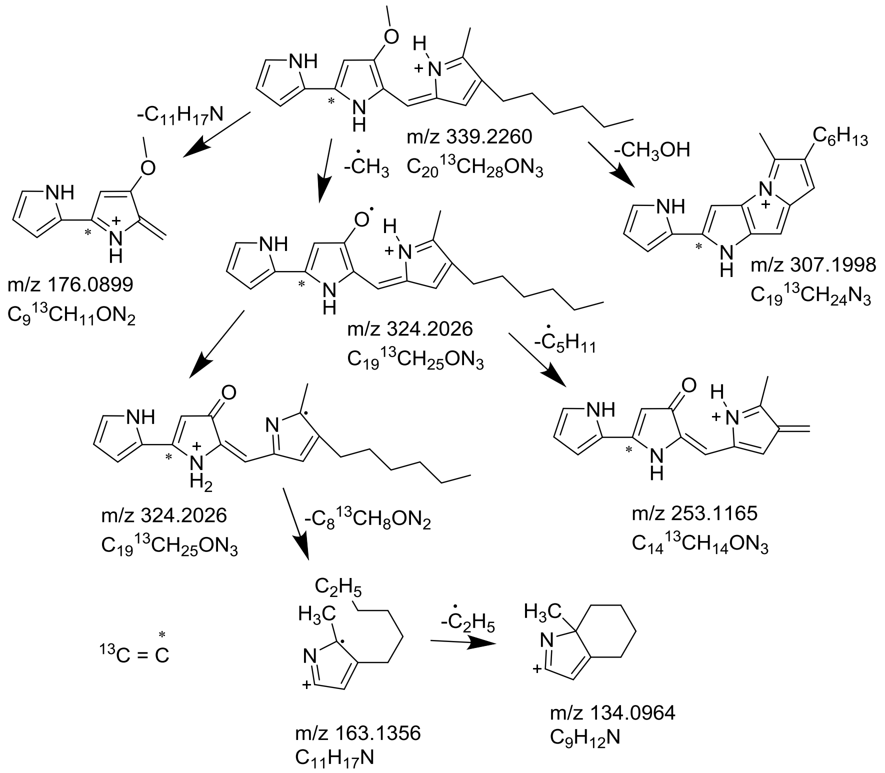

Fig. S169. Proposed mass spectral fragmentation pathway of 2-methyl-3-heptyl prodiginine with incorporated [1-^{13}C]-L-proline (compound **5** $[M+H]^+$: *m/z* 353, $C_{21}{}^{13}CH_{30}N_3O$).

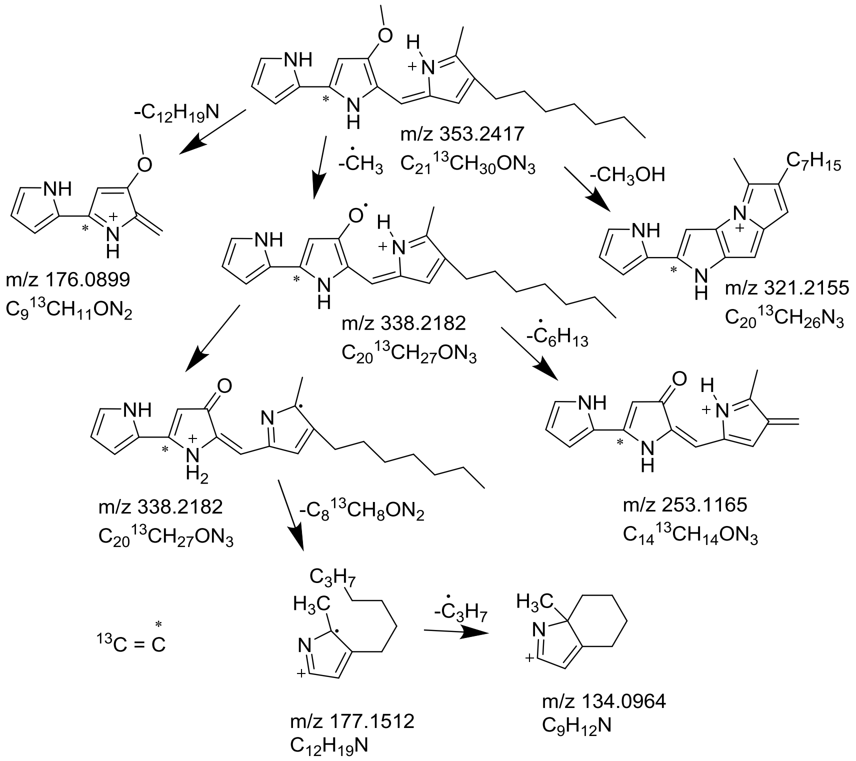

Fig. S170. Proposed mass spectral fragmentation pathway of 2-methyl-3-nonyl prodiginine with incorporated [1-^{13}C]-L-proline (compound **7** $[M+H]^+$: *m/z* 381, $C_{23}{}^{13}CH_{34}N_3O$).

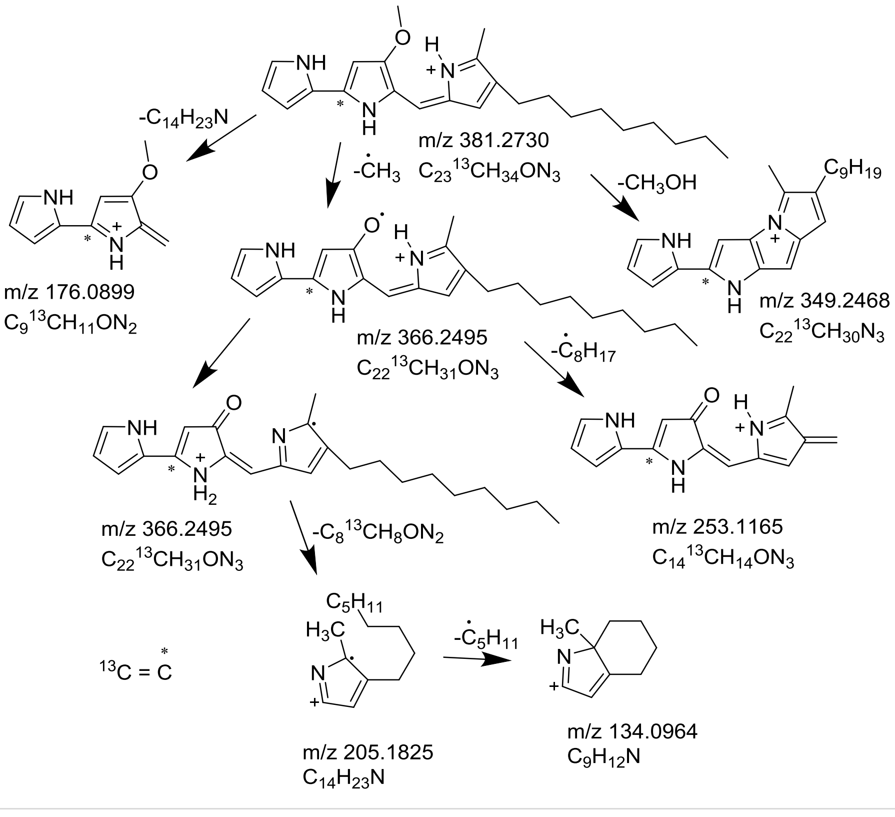

Fig. S171. HRMS^2 of prodigiosin with 5 incorporated [1,2-$^{13}C_2$]-acetate units (compound **3** $[M+H]^+$: *m/z* 334, $C_{10}{}^{13}C_{10}H_{26}N_3O$).

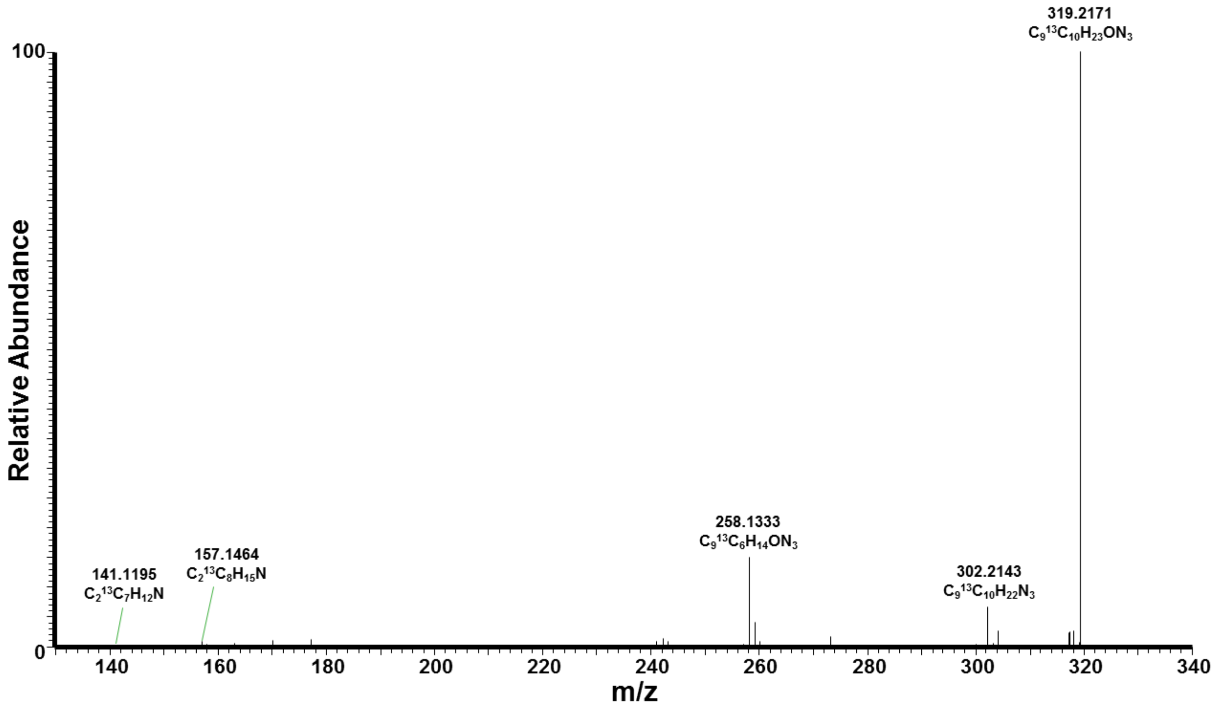

Fig. S172. HRMS^2 of prodigiosin with 6 incorporated [1,2-$^{13}C_2$]-acetate units (compound **3** $[M+H]^+$: *m/z* 336, $C_8{}^{13}C_{12}H_{26}N_3O$).

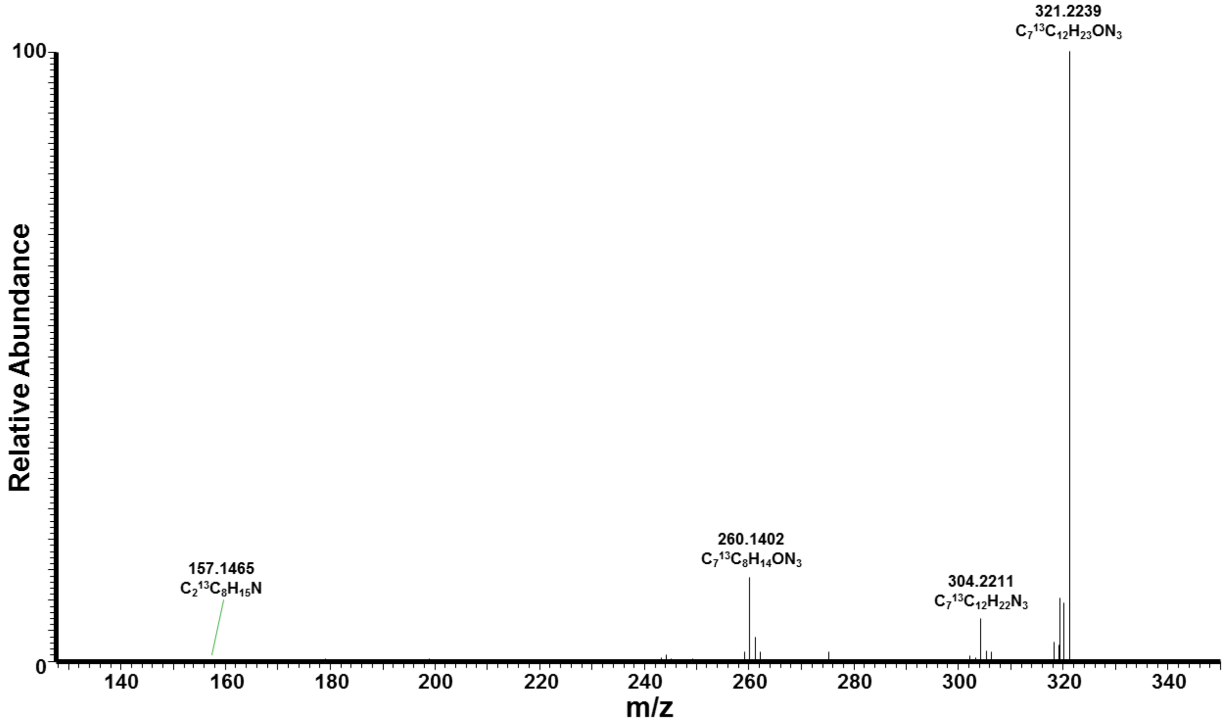

Fig. S173. HRMS[2] of prodigiosin with 7 incorporated [1,2-$^{13}C_2$]-acetate units (compound **3** $[M+H]^+$: *m/z* 338, $C_6{}^{13}C_{14}H_{26}N_3O$).

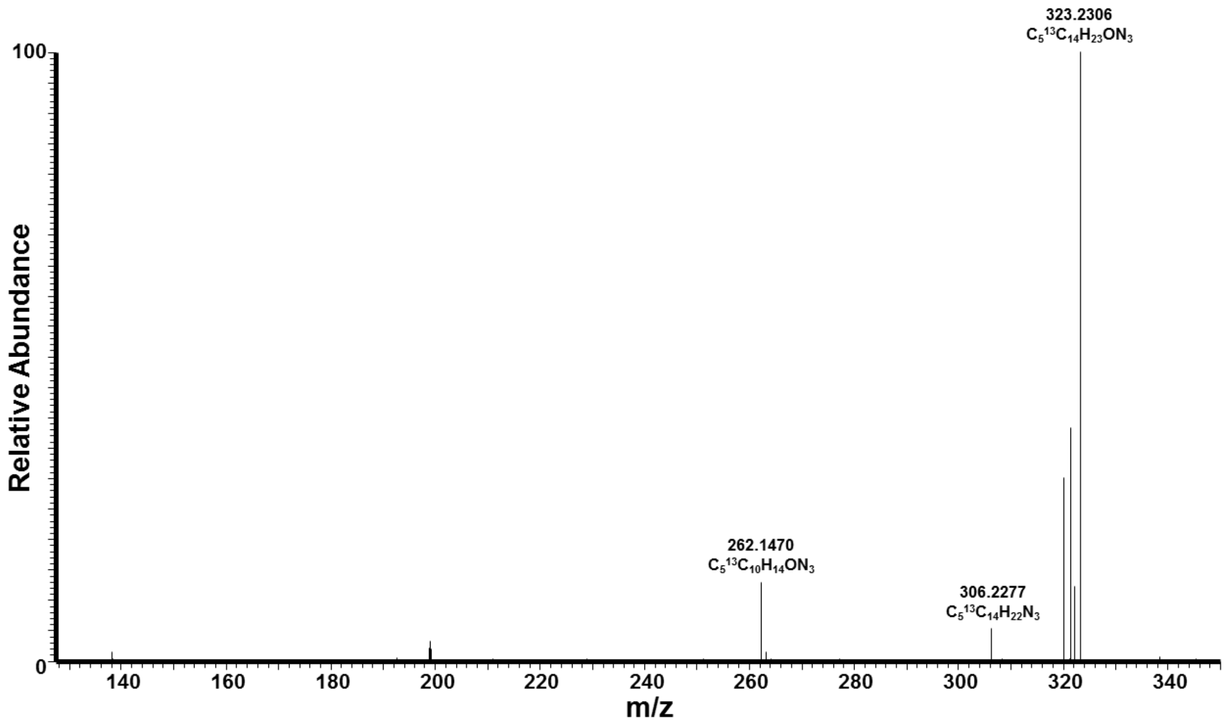

Fig. S174. Confrontation assay of endophytic *Pichia* spp. against *S. aureus* (DSM 799) and *E. coli* (DSM 682).

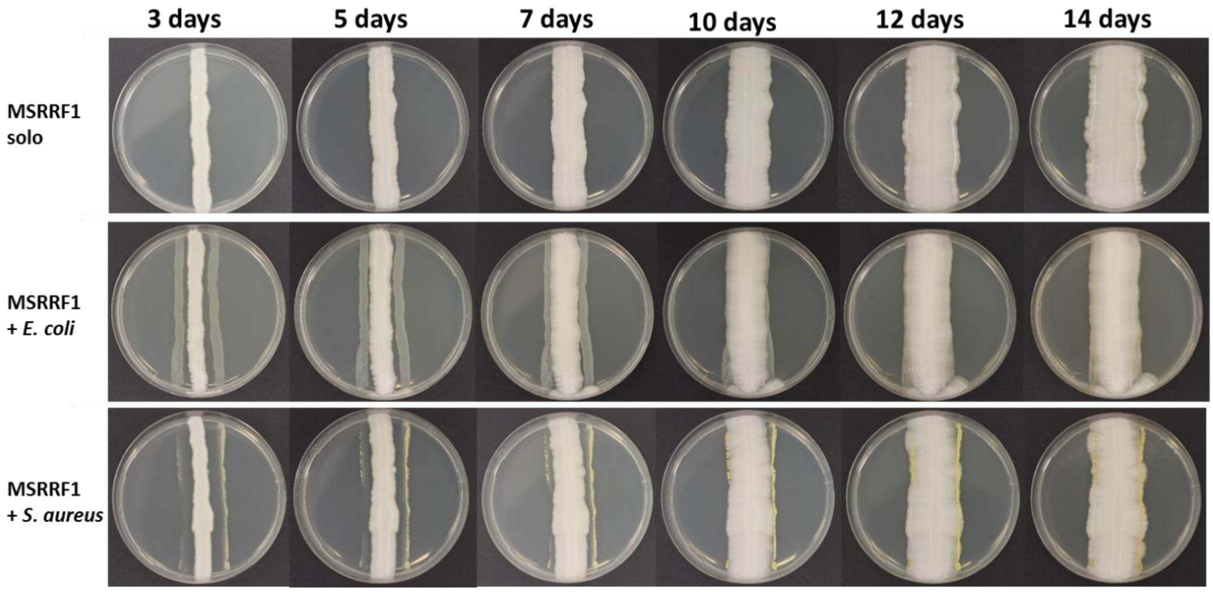

Fig. S175. Confrontation assay of endophytic *A. caesiellus* against *S. aureus* (DSM 799) and *E. coli* (DSM 682).

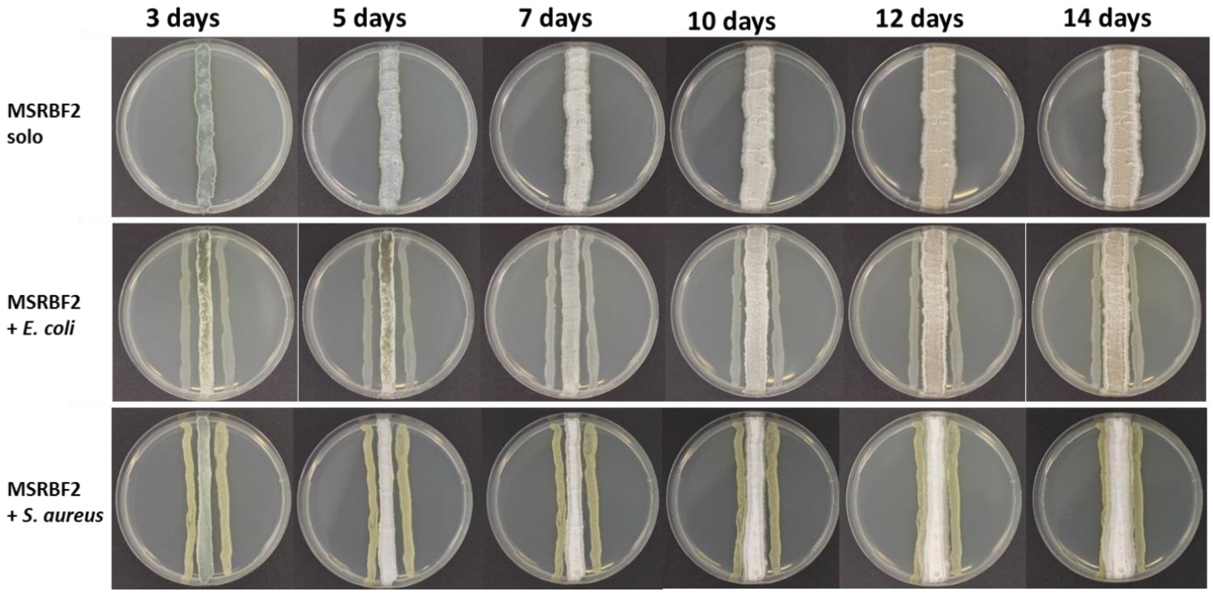

Fig. S176. Confrontation assay of endophytic *P. virgatula* against *S. aureus* (DSM 799) and *E. coli* (DSM 682).

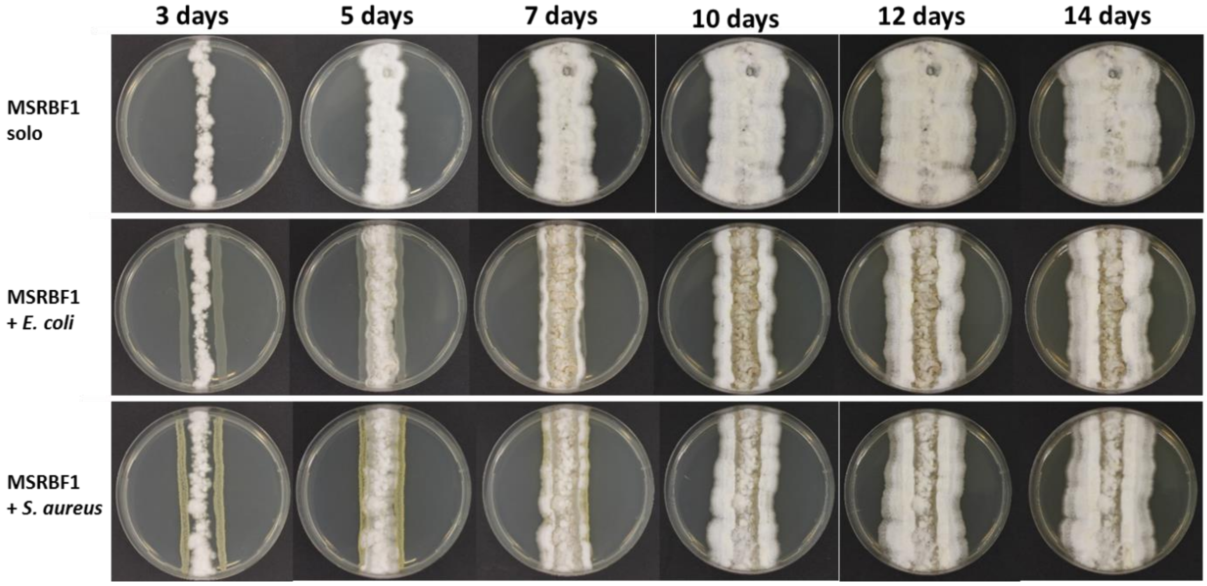

Fig. S177. Scanning electron microscopic images of endophytic *S. marcescens* MSRBB2.

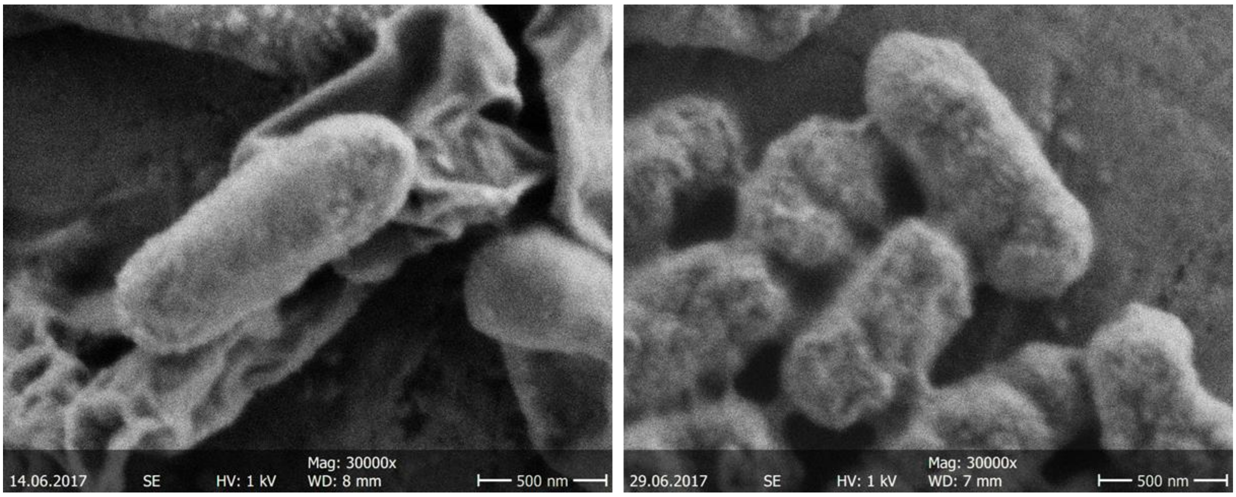

Fig. S178. Scanning electron microscopic images of endophytic *P. virgatula*.

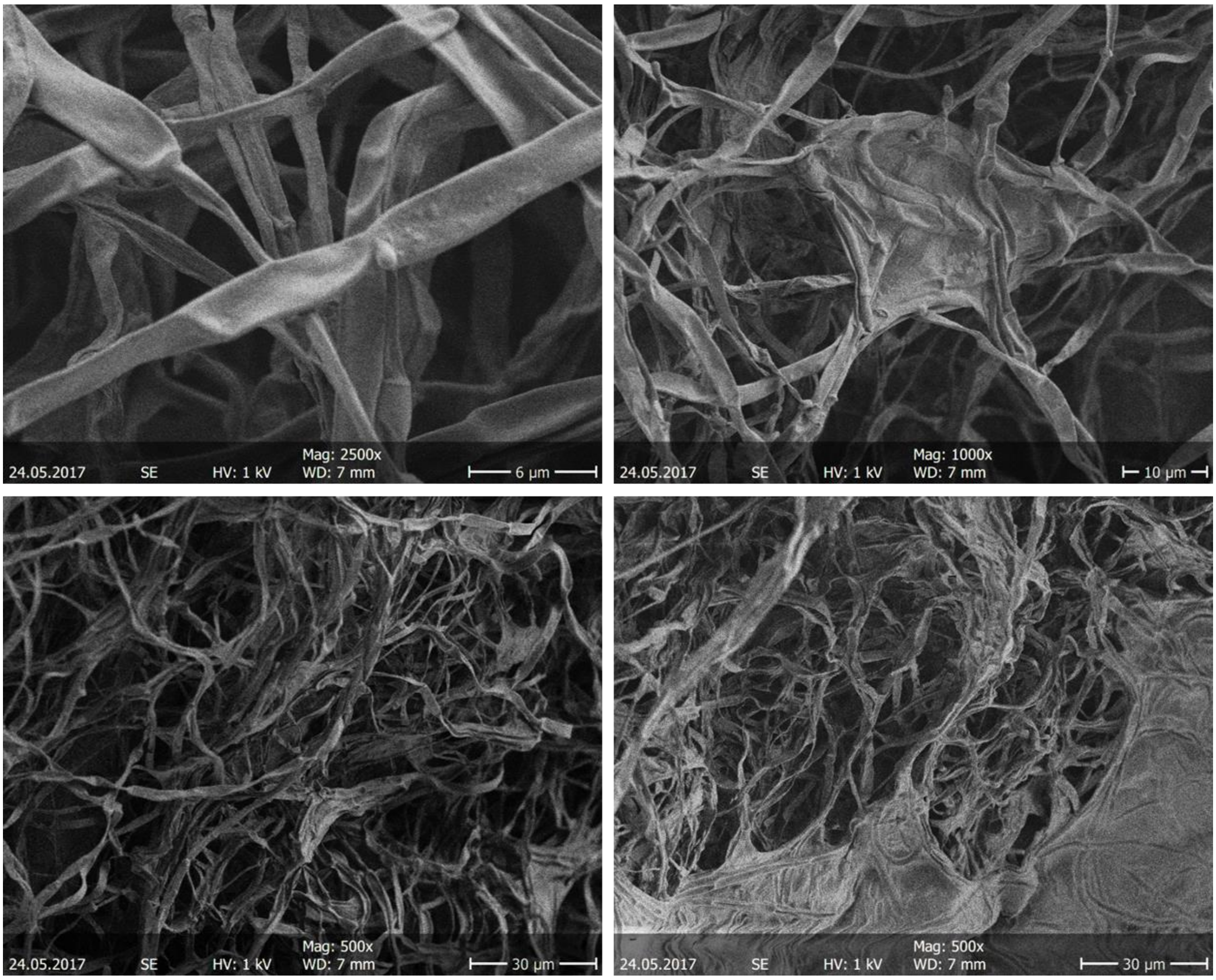

Fig. S179. Scanning electron microscopic images (1) of endophytic *A. caesiellus*.

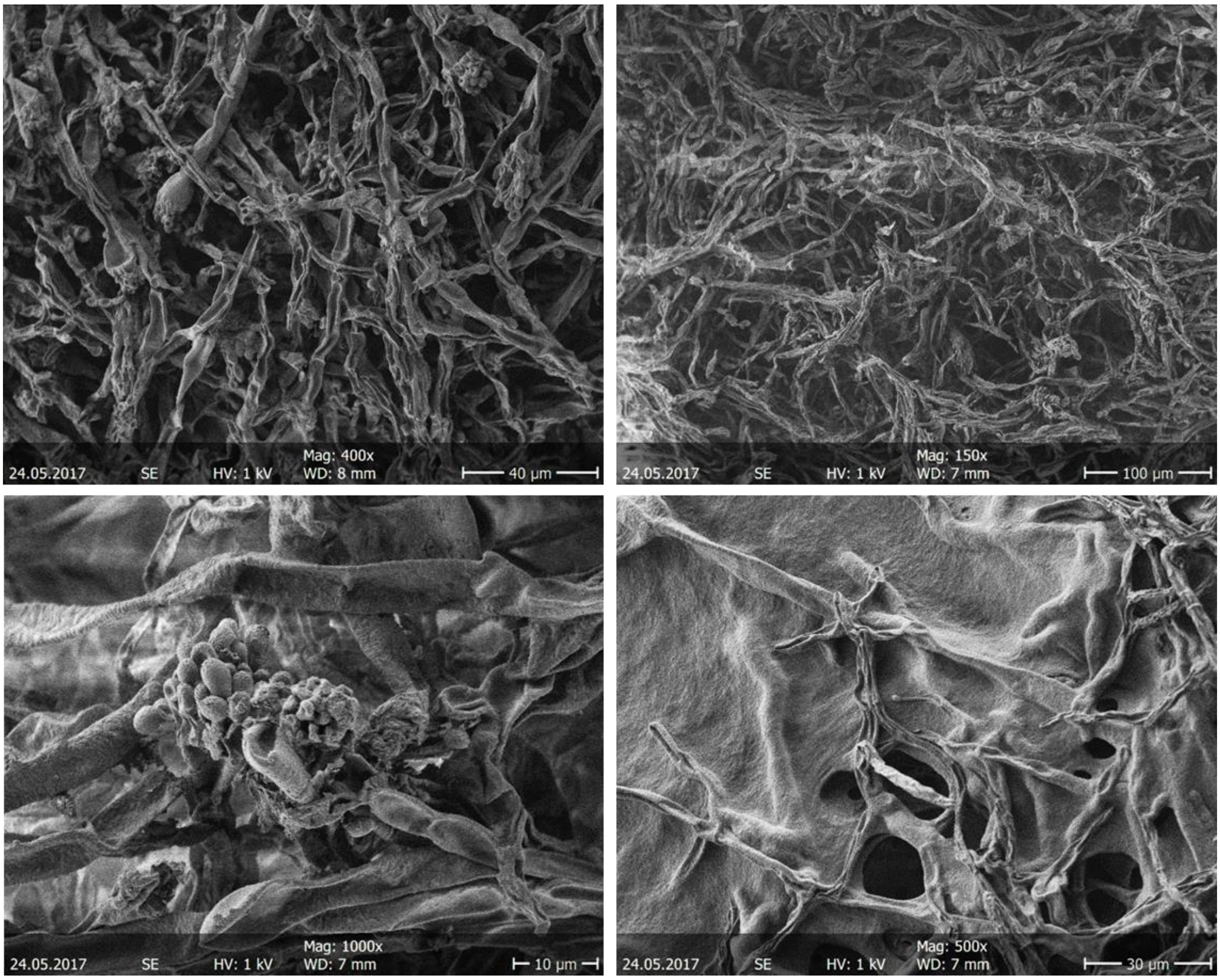

Fig. S180. Scanning electron microscopic images (2) of endophytic *A. caesiellus*.

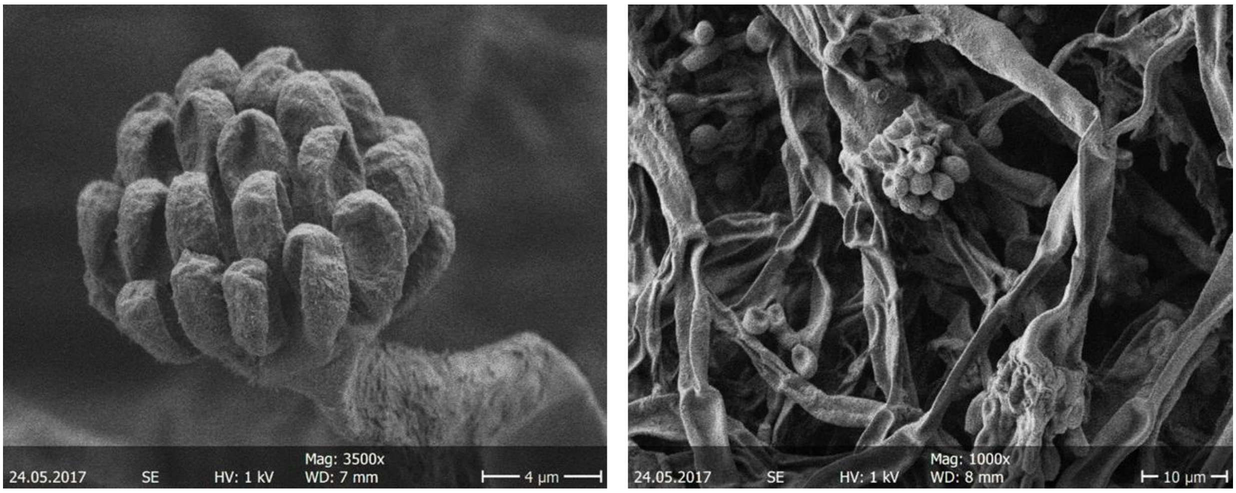

Fig. S181. Scanning electron microscopic images (1) of endophytic *Pichia* spp.

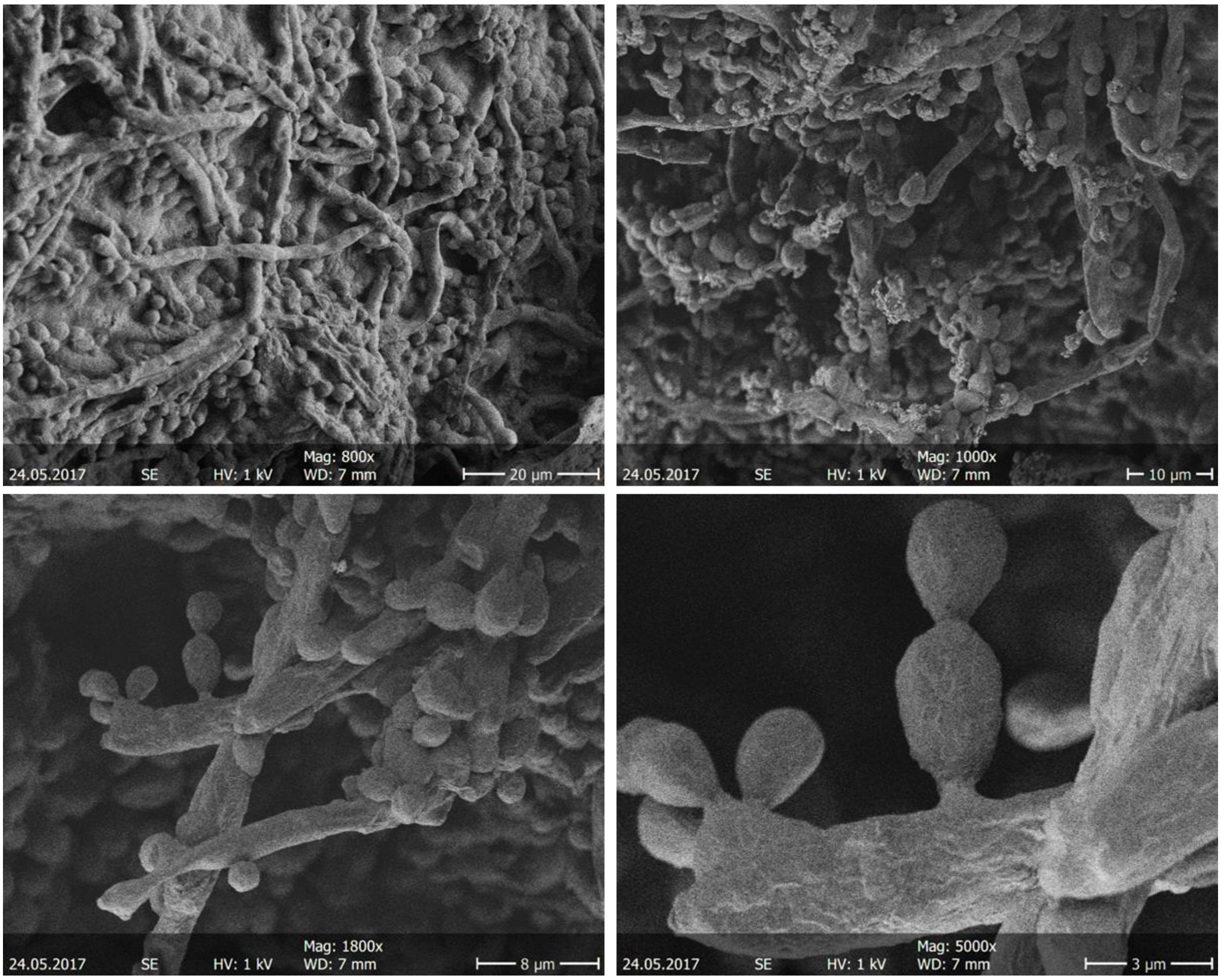

Fig. S182. Scanning electron microscopic images (2) of endophytic *Pichia* spp.

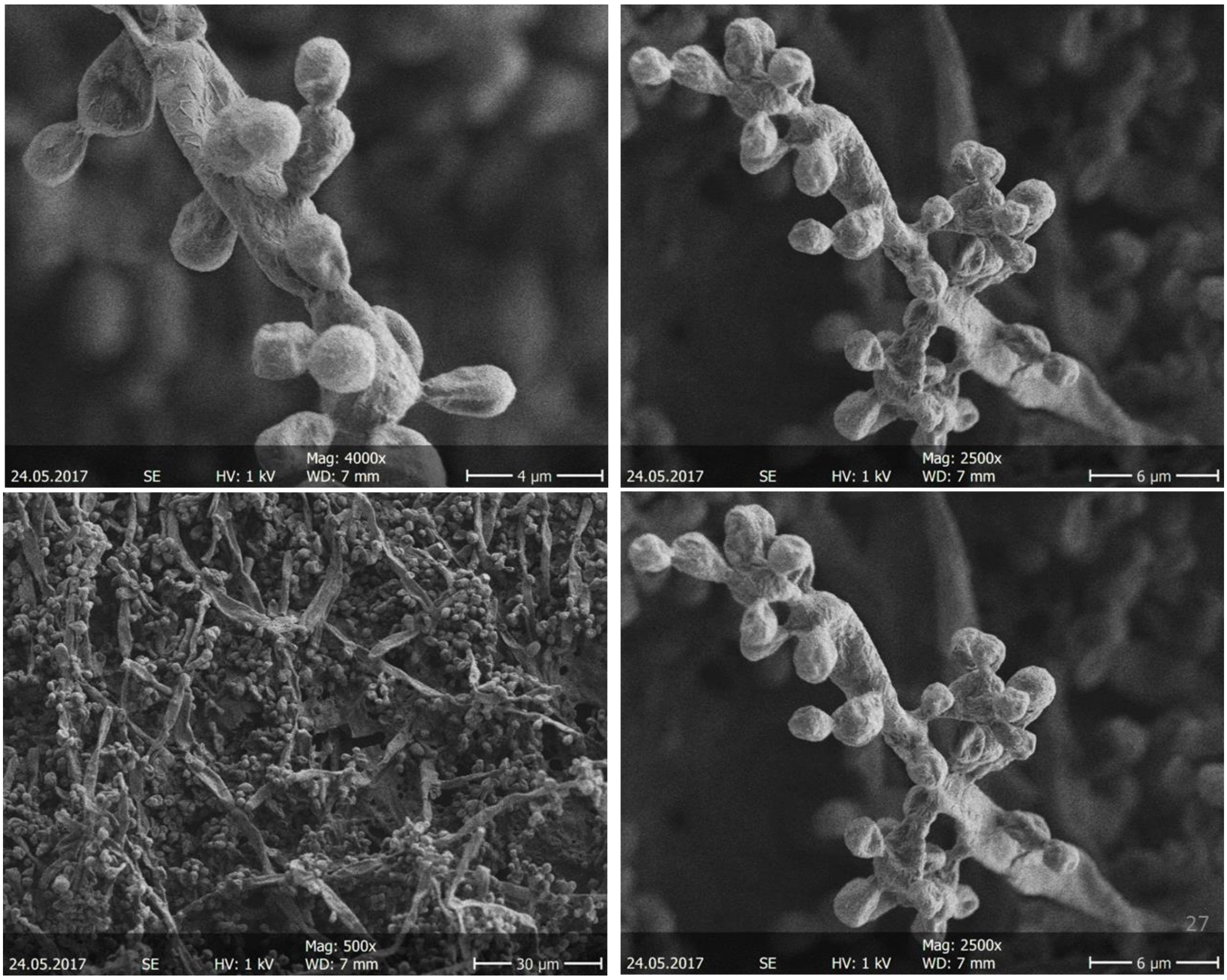

Fig. S183. Scanning electron microscopic images (1) of co-cultivation of endophytic *P. virgatula* and *S. marcescens* MSRBB2.

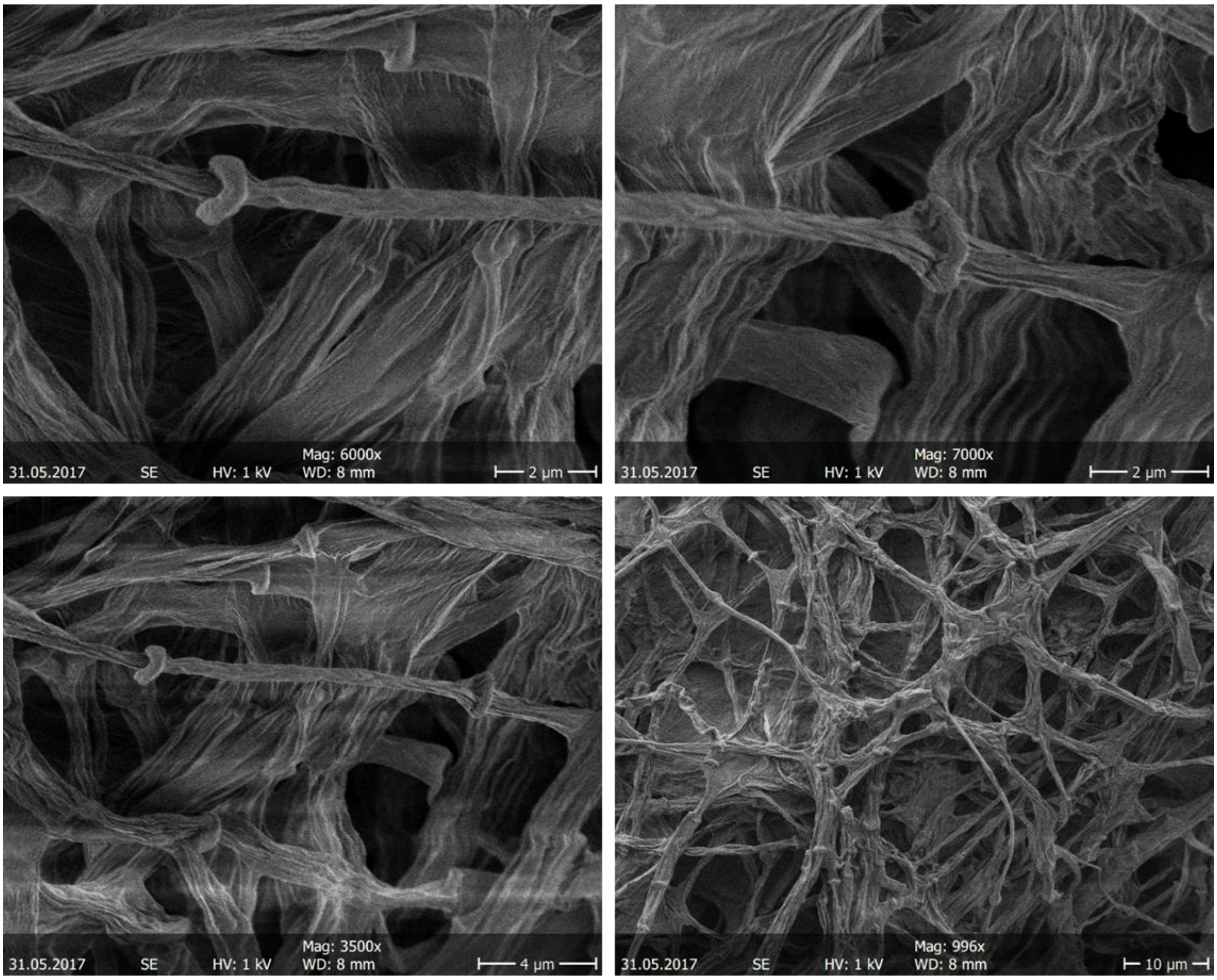

Fig. S184. Scanning electron microscopic images (2) of co-cultivation of endophytic *P. virgatula* and *S. marcescens* MSRBB2.

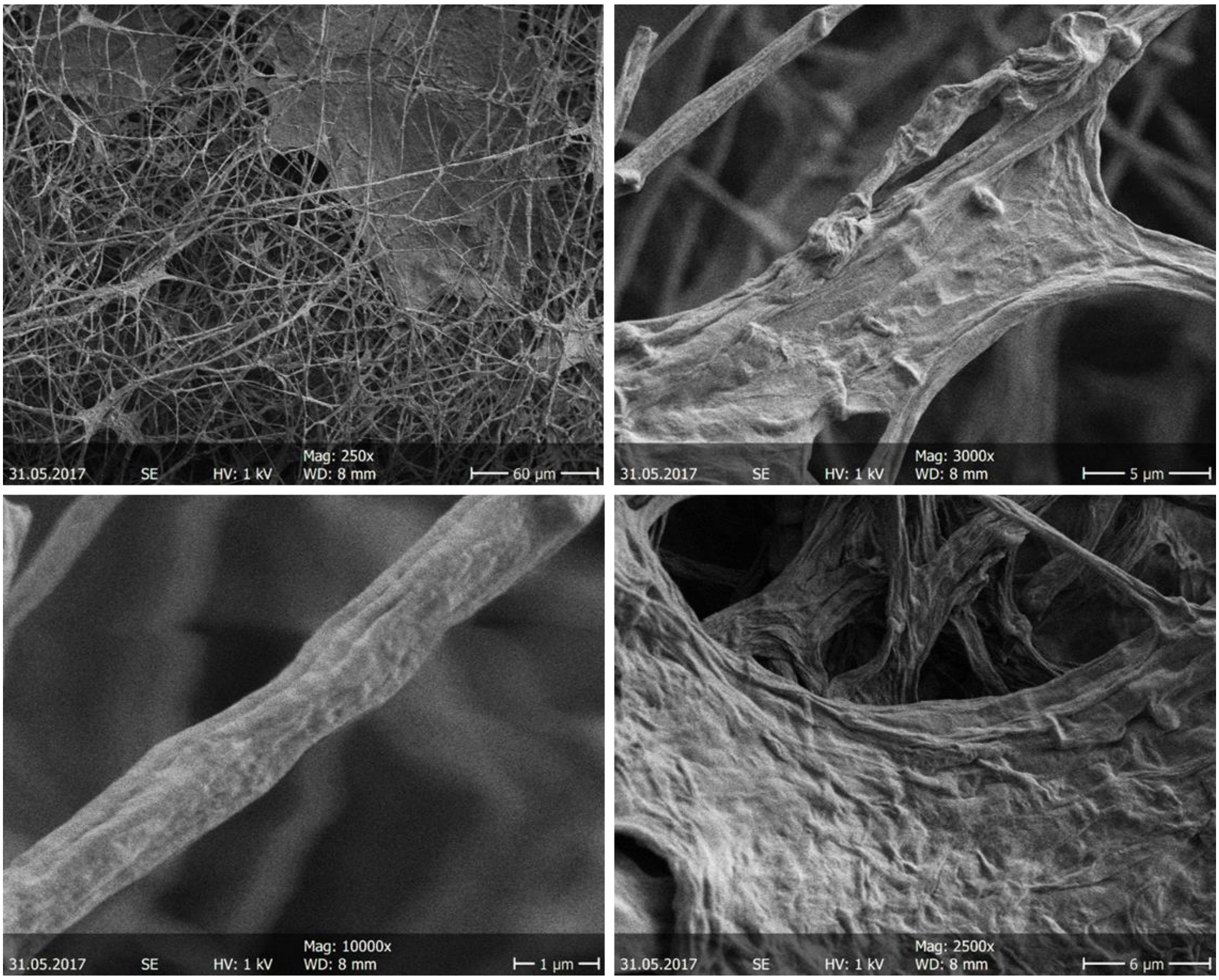

Fig. S185. Scanning electron microscopic images (3) of co-cultivation of endophytic *P. virgatula* and *S. marcescens* MSRBB2.

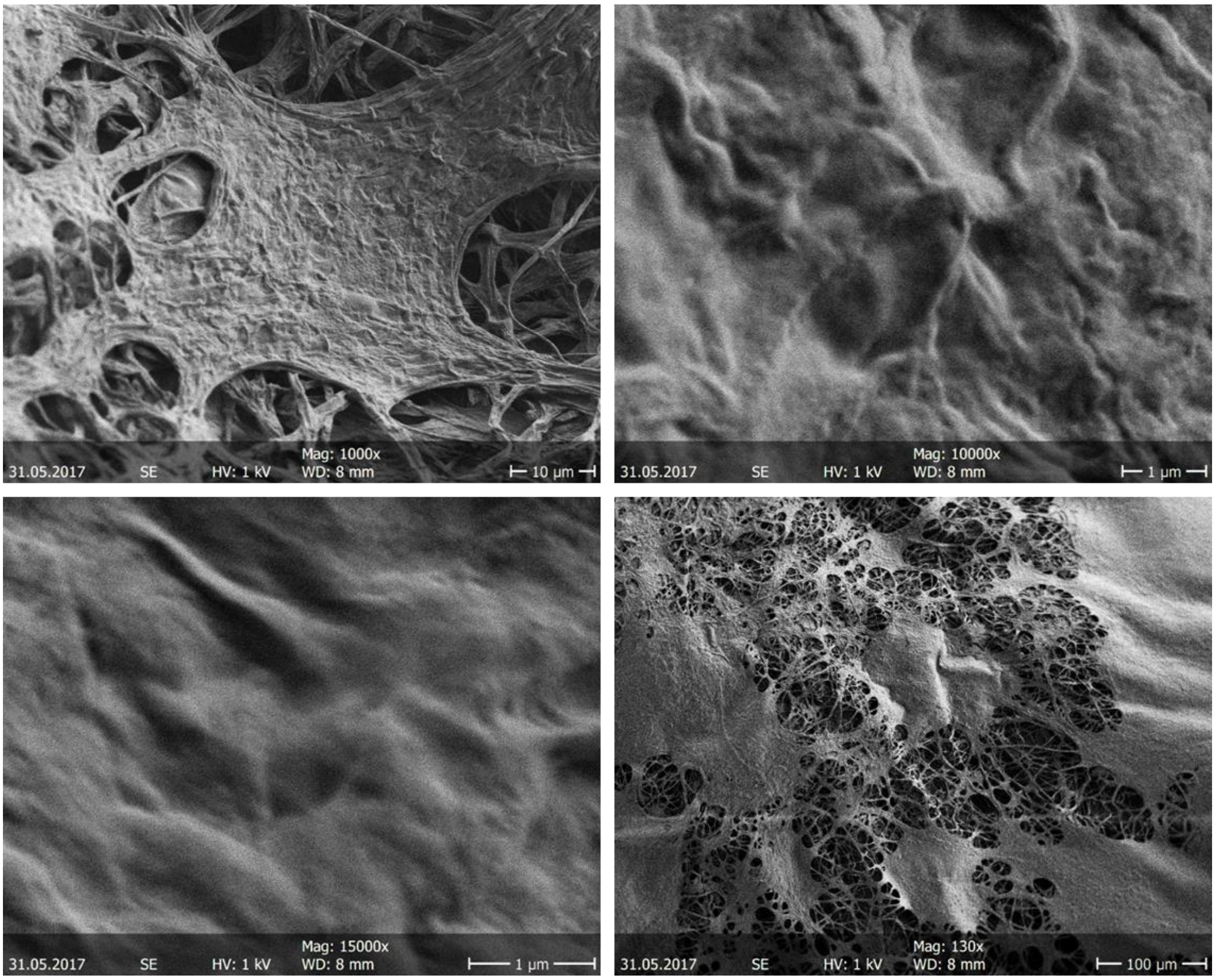

Fig. S186. Scanning electron microscopic images (1) of co-cultivation of endophytic *A. caesiellus* and *S. marcescens* MSRBB2.

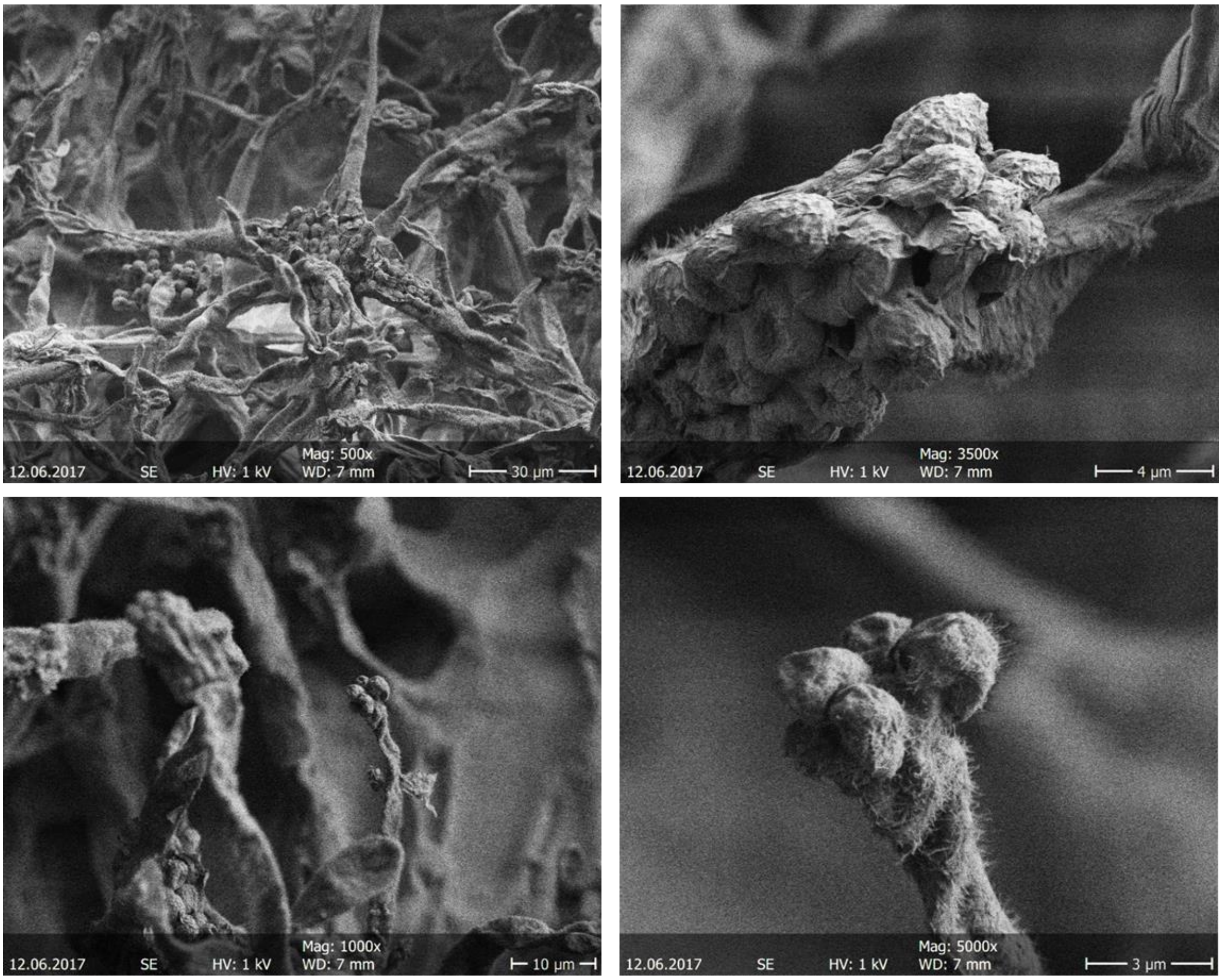

Fig. S187. Scanning electron microscopic images (2) of co-cultivation of endophytic *A. caesiellus* and *S. marcescens* MSRBB2.

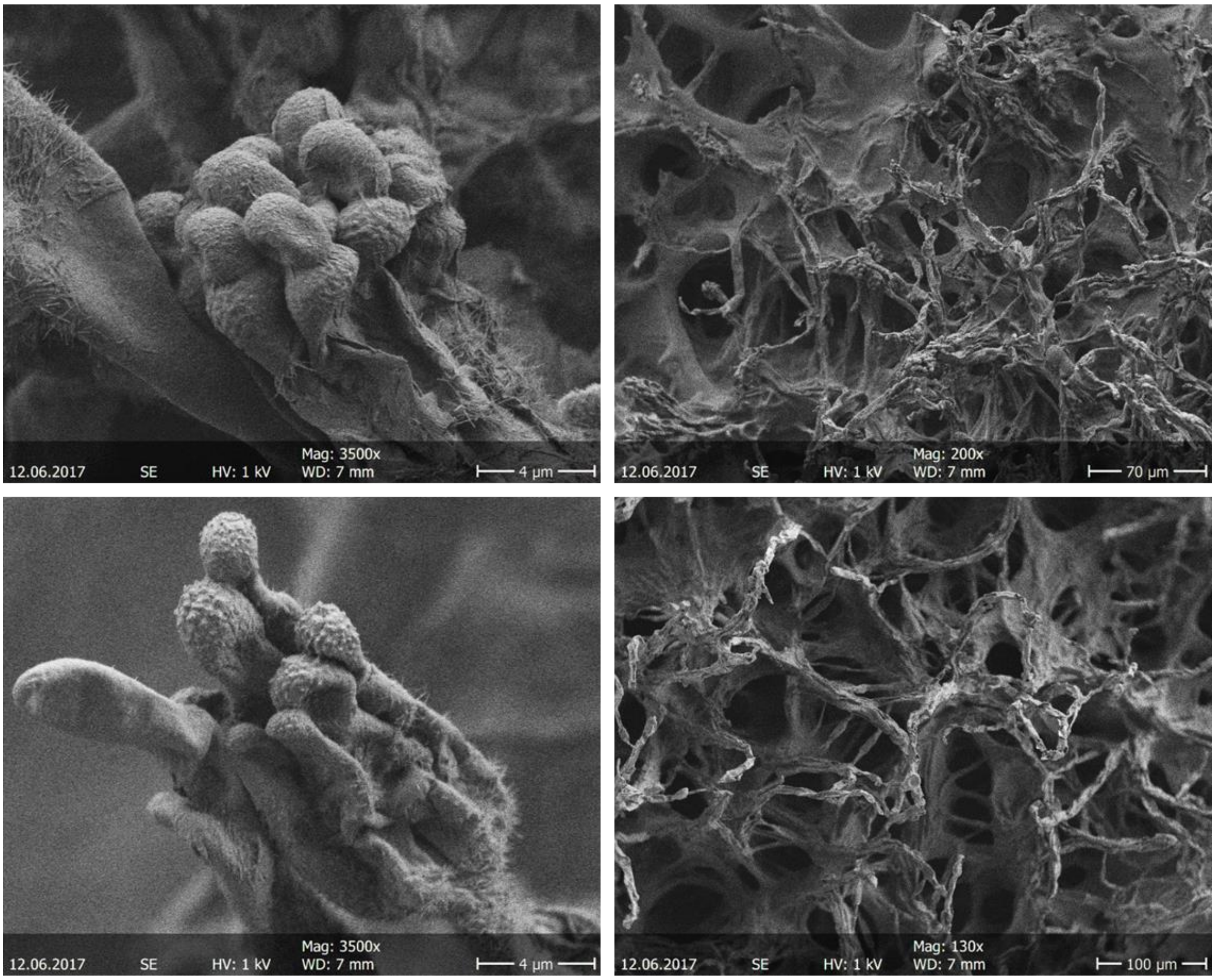

Fig. S189. Scanning electron microscopic images (3) of co-cultivation of endophytic *A. caesiellus* and *S. marcescens* MSRBB2.

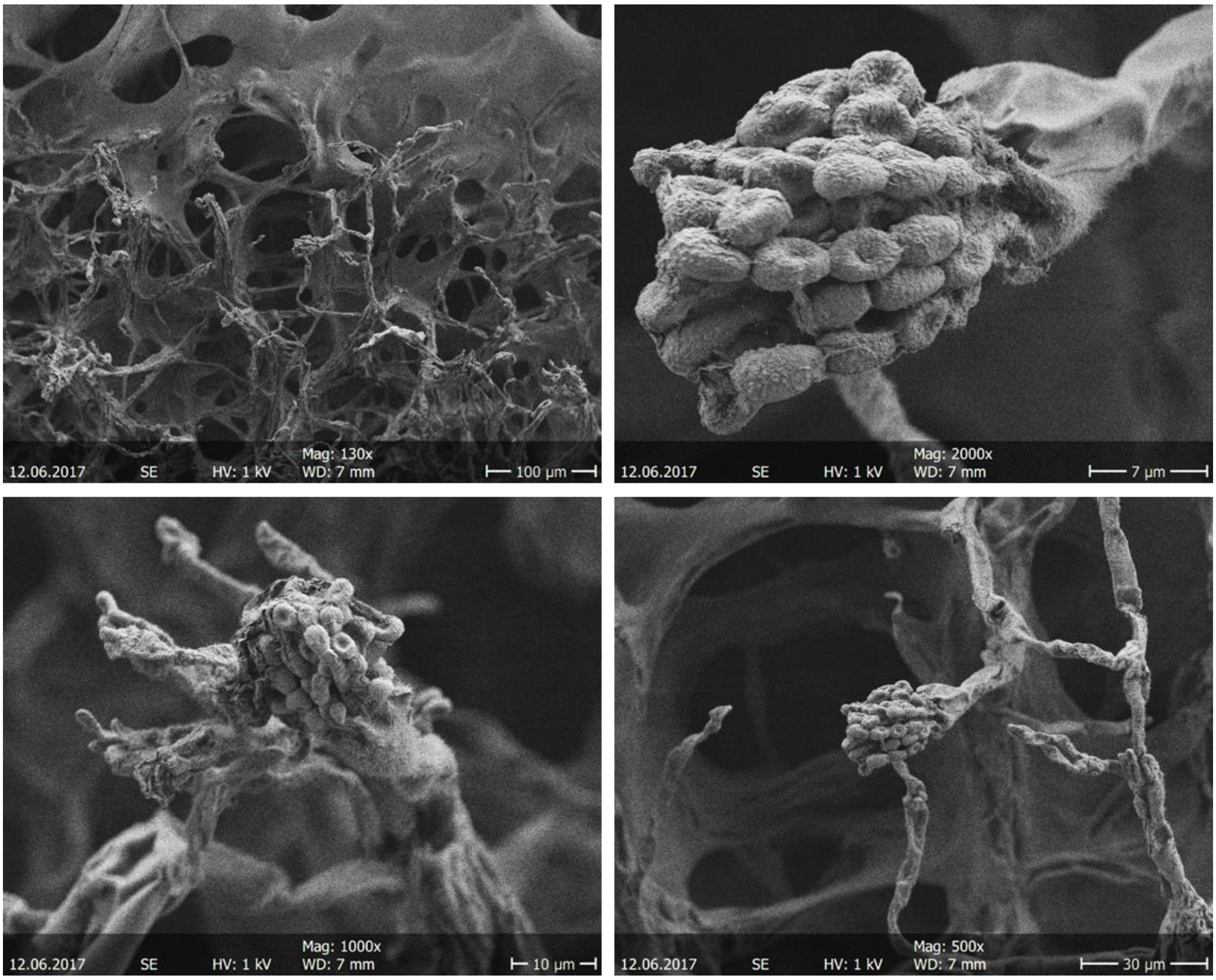

Fig. S190. Scanning electron microscopic images (1) of co-cultivation of endophytic *Pichia* spp. and *S. marcescens* MSRBB2.

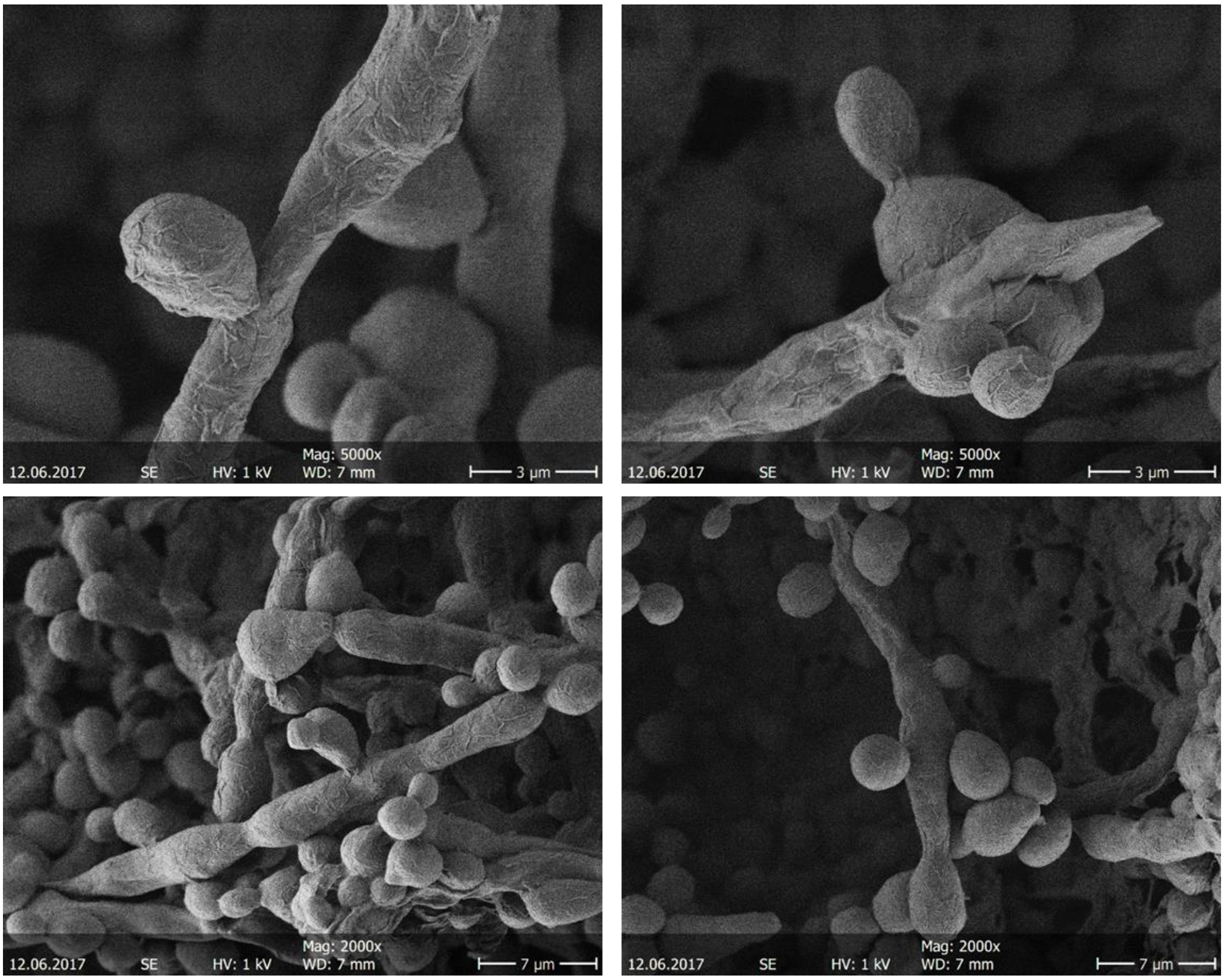

Fig. S191. Scanning electron microscopic images (2) of co-cultivation of endophytic *Pichia* spp. and *S. marcescens* MSRBB2.

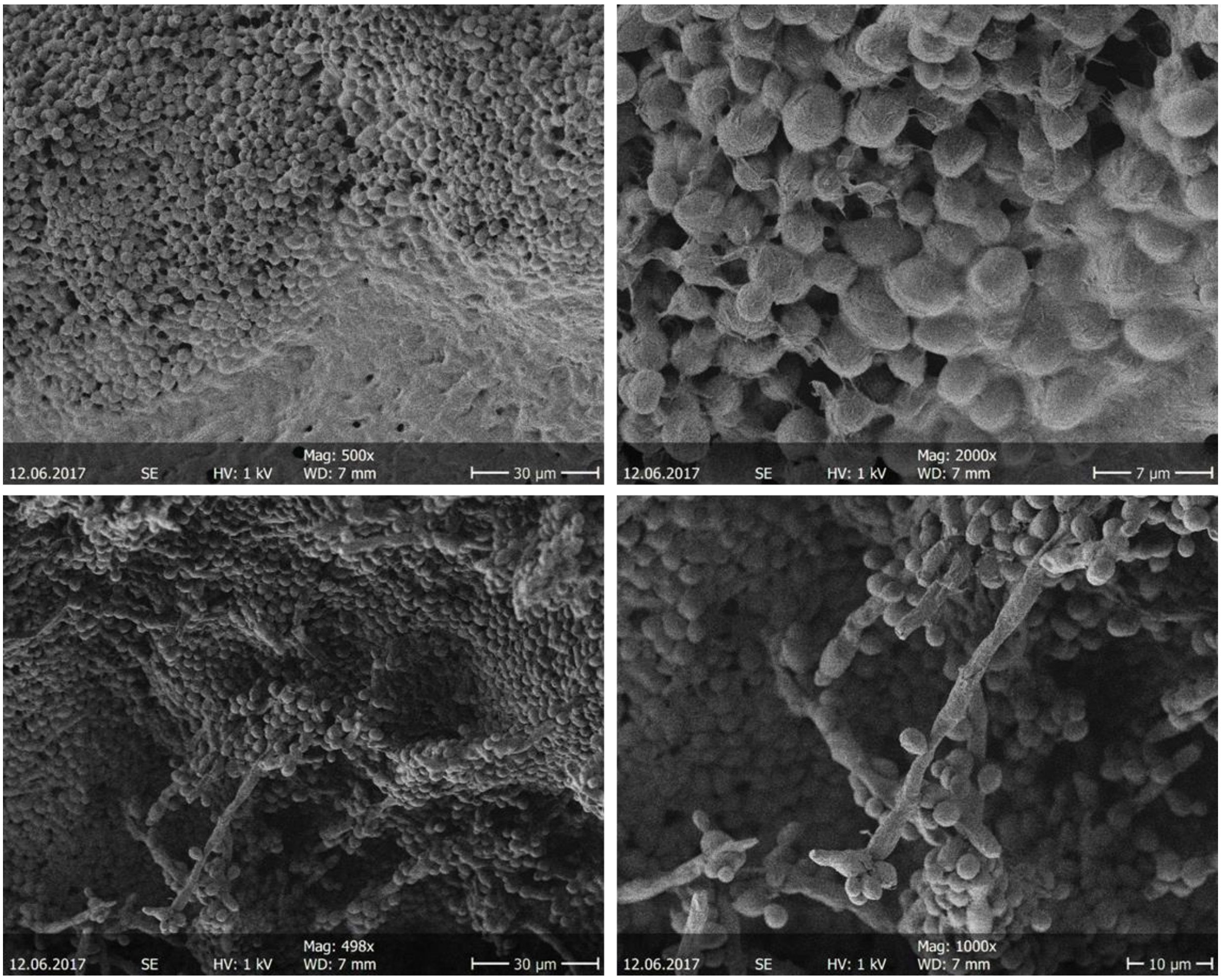

Fig. S192. Scanning electron microscopic images of *Aspergillus fumigatus* (DSM 21023).

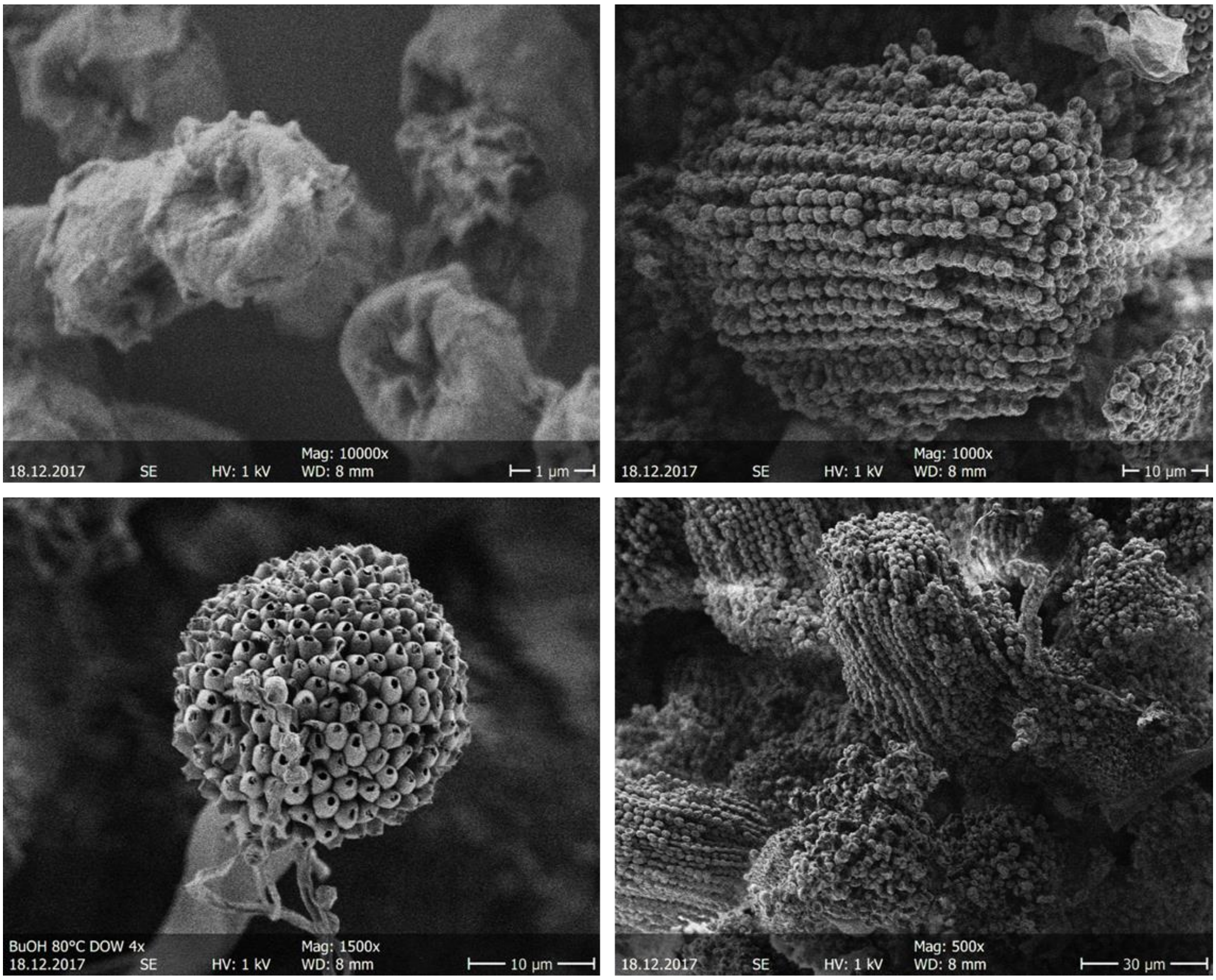

Fig. S193. Scanning electron microscopic images of co-cultivation of endophytic *S. marcescens* MSRBB2 and *Aspergillus fumigatus* (DSM 21023).

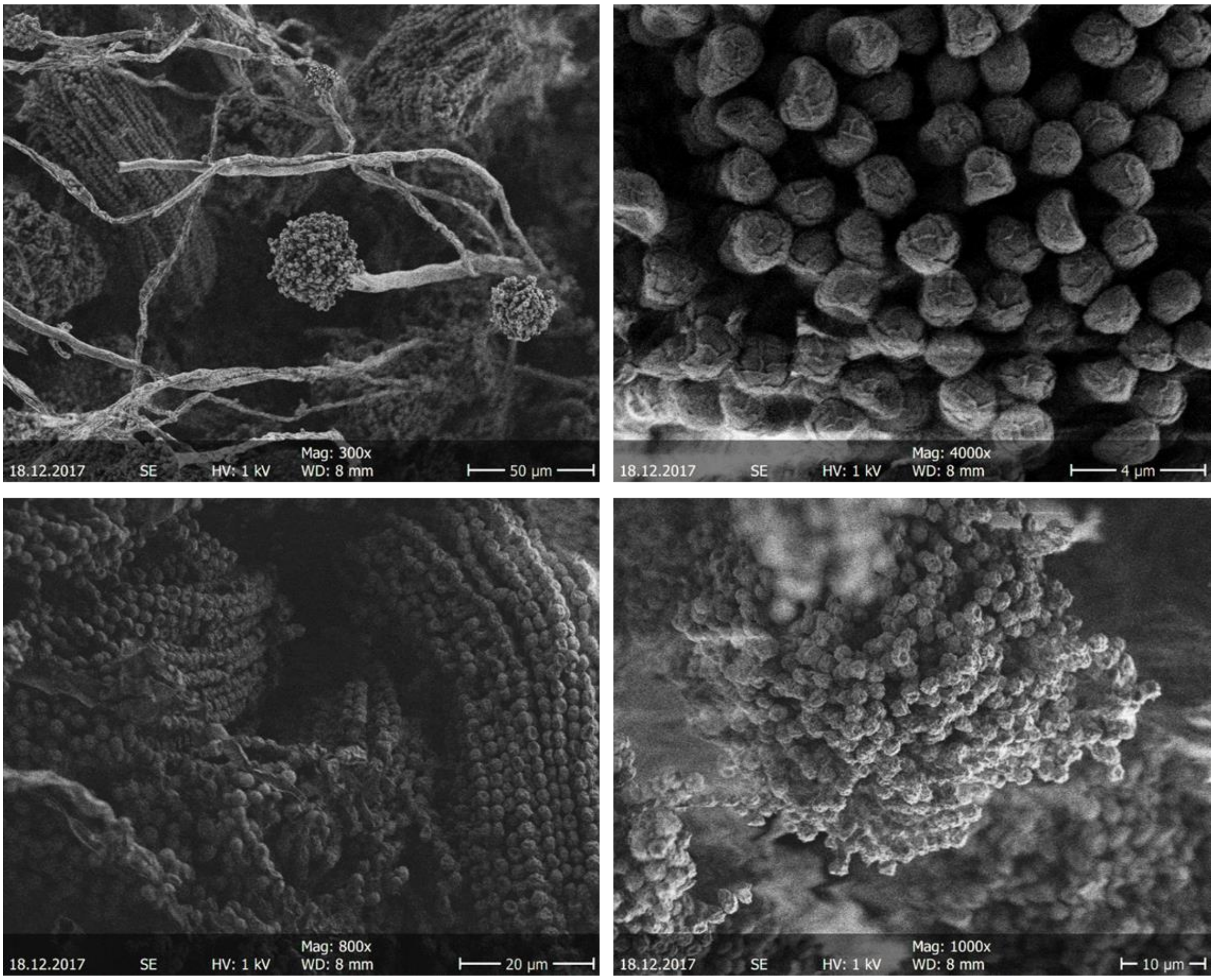

Fig. S194. Scanning electron microscopic images of *Pichia membranifaciens* (DSM 70366).

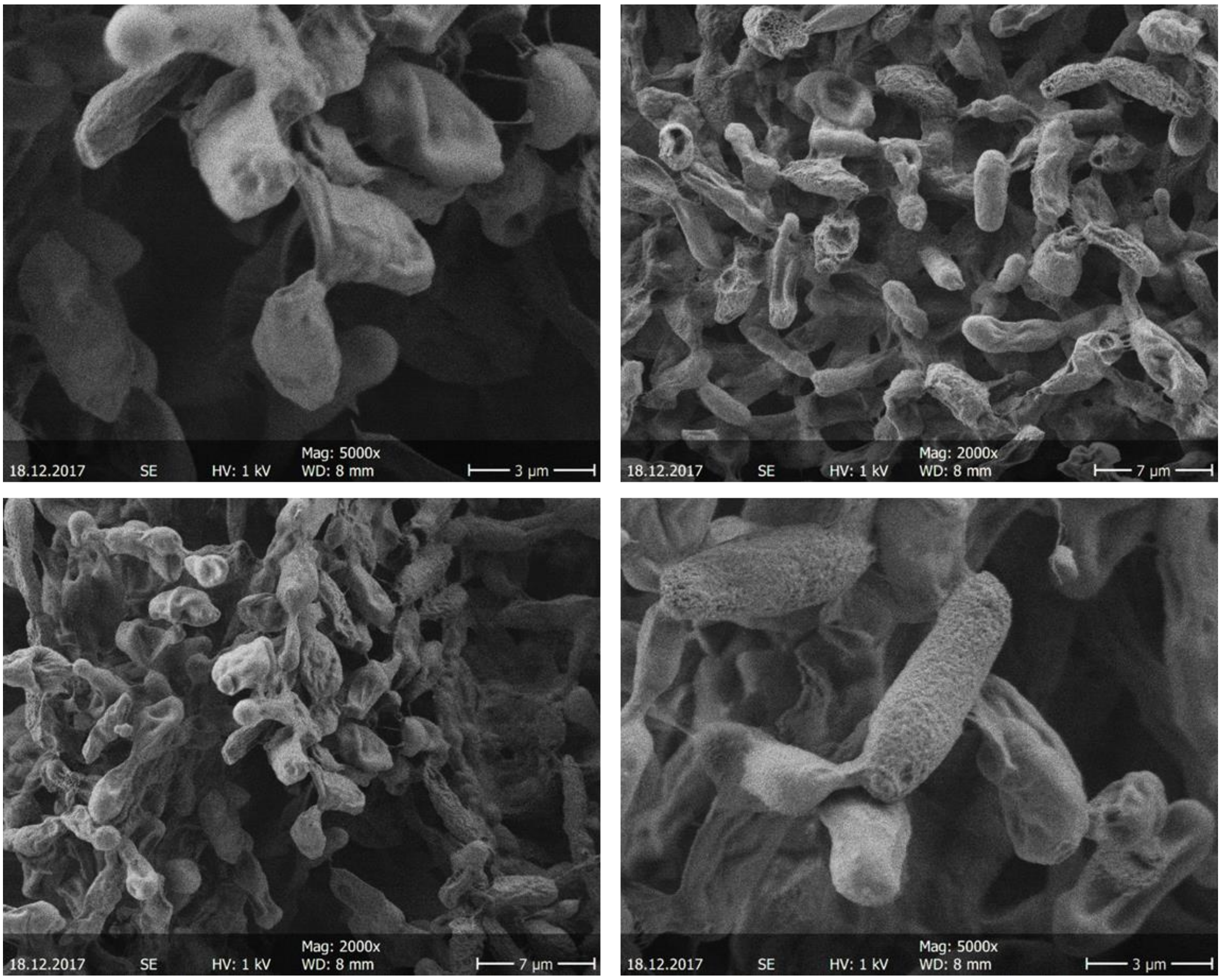

Fig. S195. Scanning electron microscopic images of co-cultivation of endophytic *S. marcescens* MSRBB2 and *Pichia membranifaciens* (DSM 70366).

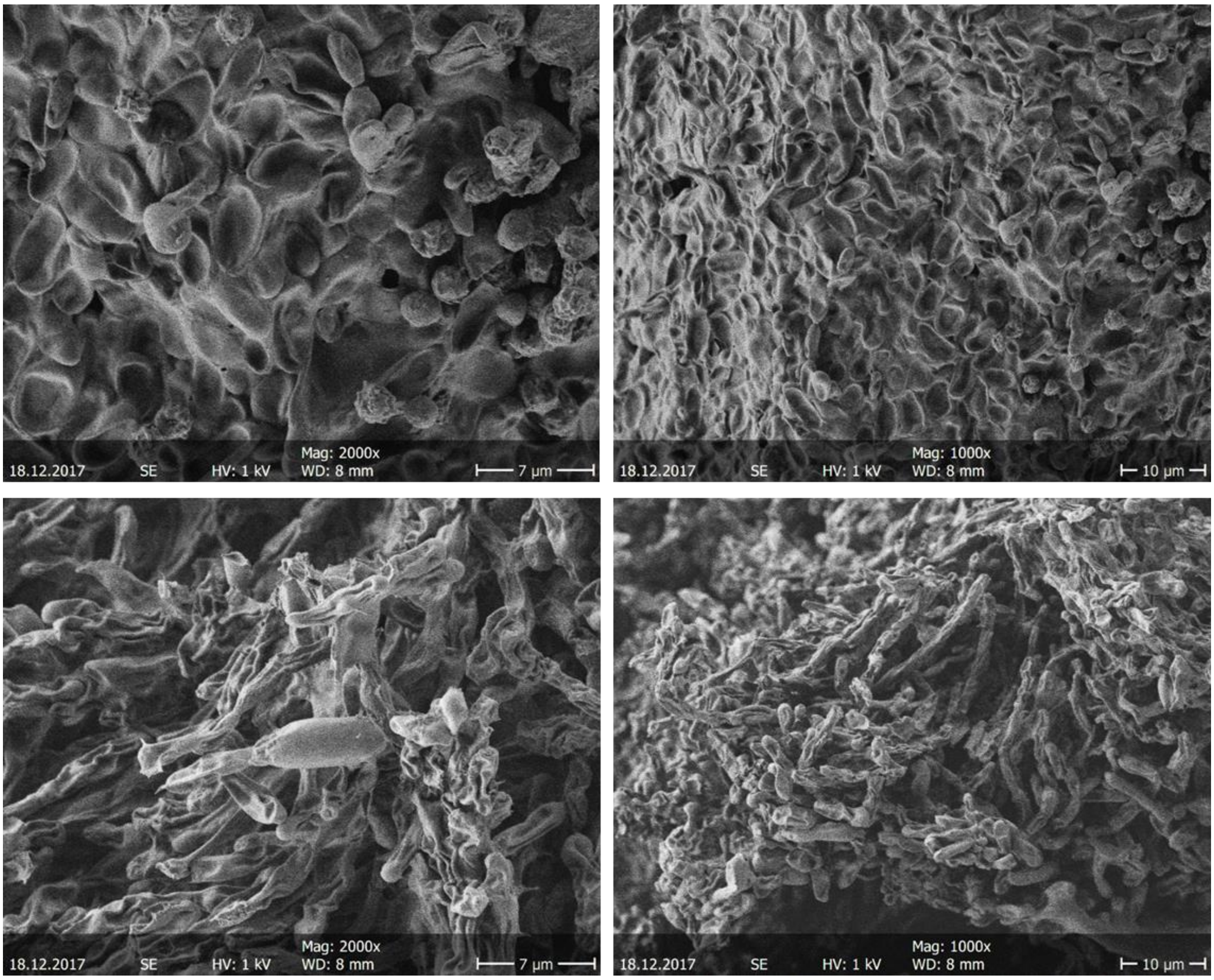

Fig. S196. Scanning electron microscopic images of *Pestalotiopsis versicolor* (DSM 62887).

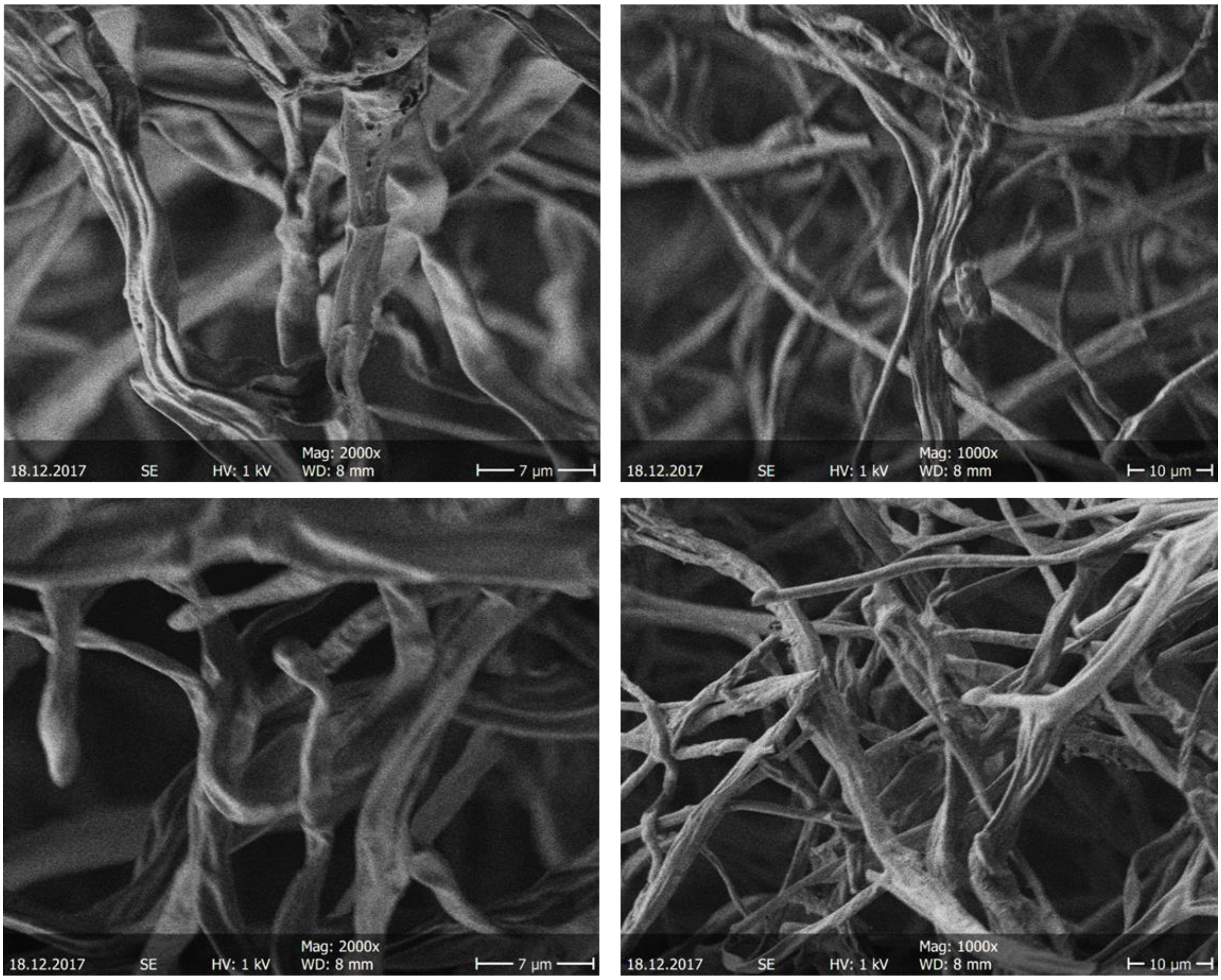

Fig. S197. Scanning electron microscopic images of co-cultivation of endophytic *S. marcescens* MSRBB2 and *Pestalotiopsis versicolor* (DSM 62887).

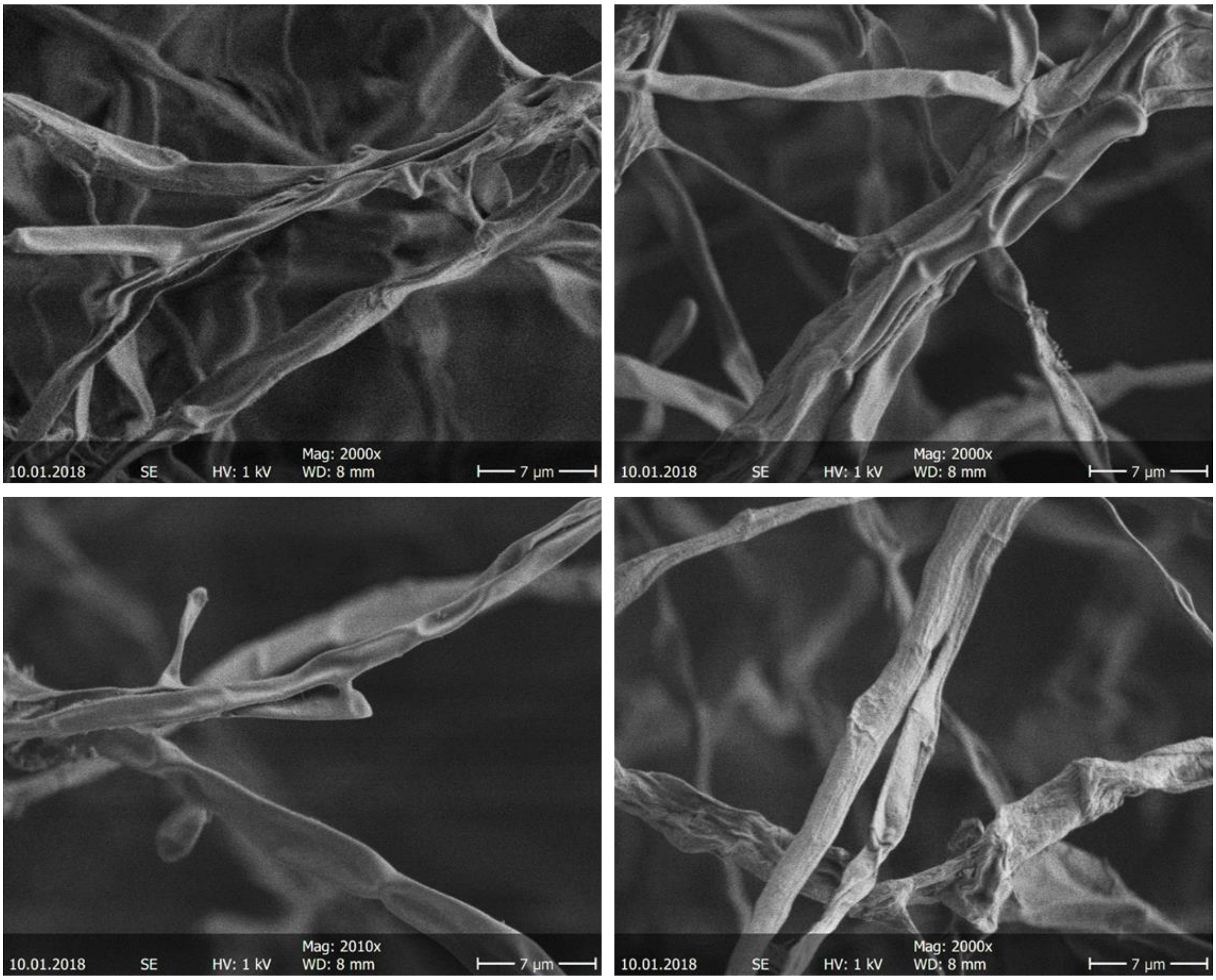

Fig. S198. Dose and time dependent disc line assay of different prodigiosin concentrations against *A. caesiellus*

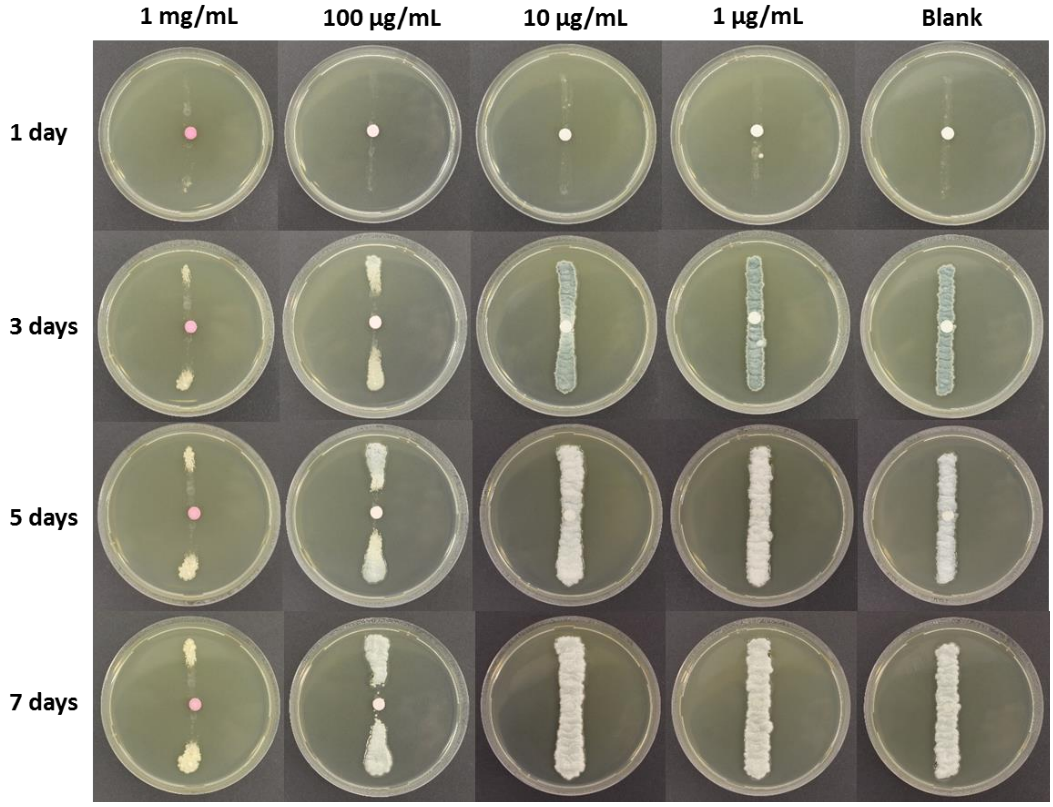

Fig. S199. Dose and time dependent Y-shape disc assay of different prodigiosin concentrations against *A. caesiellus*

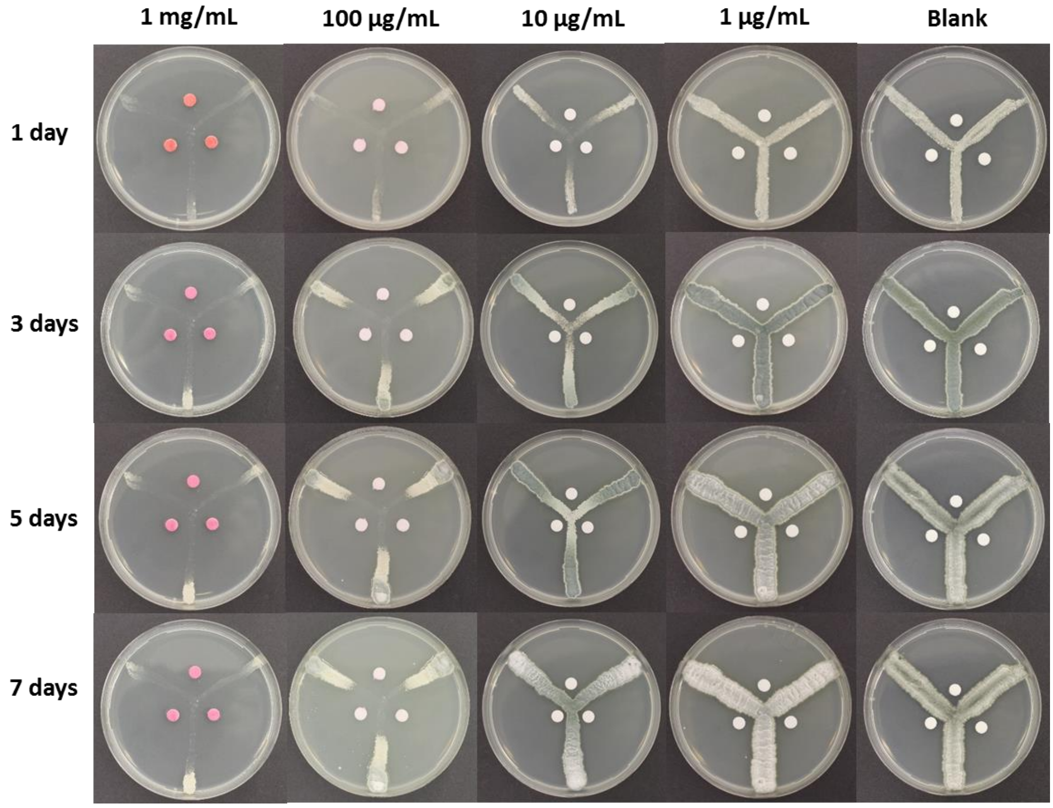

Fig. S200. Dose and time dependent disc line assay of different prodigiosin concentrations against *Pichia* spp.

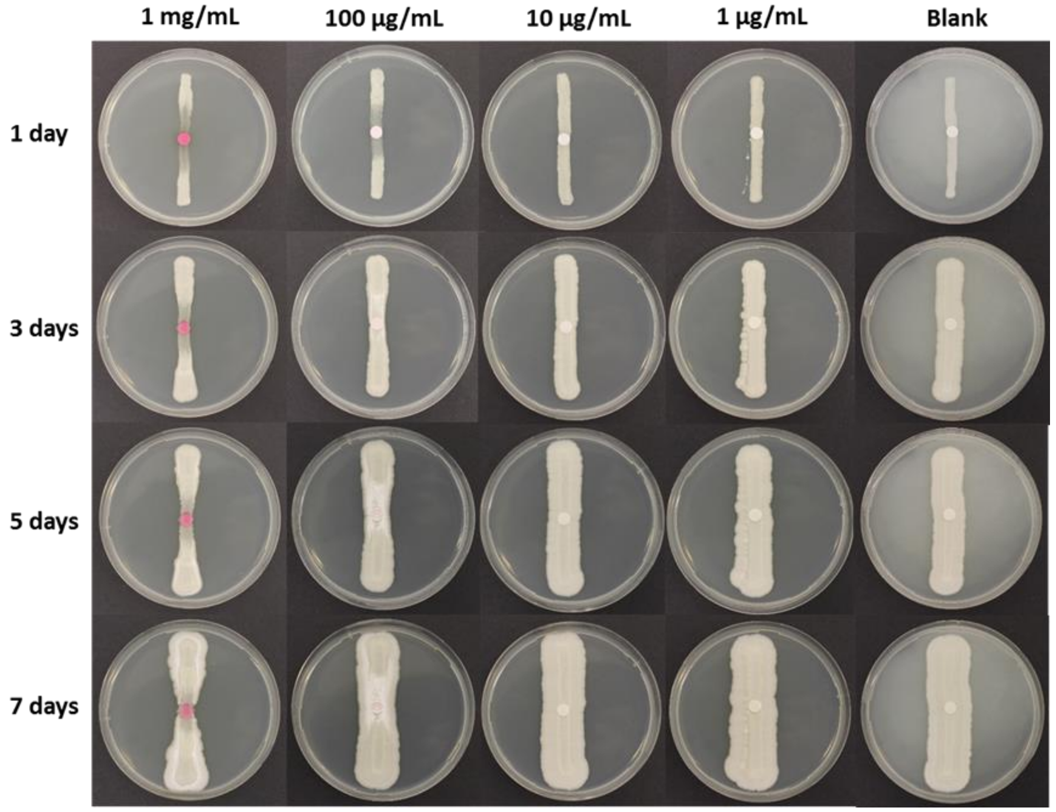

Fig. S201. Dose and time dependent Y-shape disc assay of different prodigiosin concentrations against *Pichia* spp.

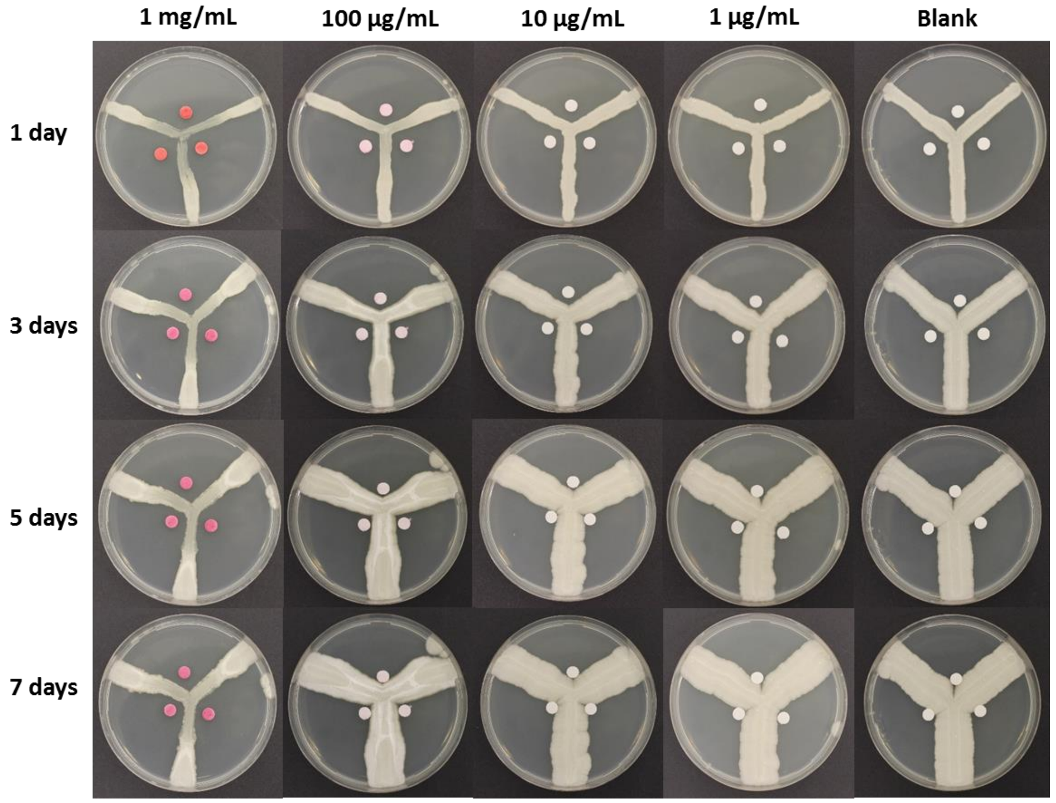

Fig. S202. Dose and time dependent disc line assay of different prodigiosin concentrations against *P. virgatula*

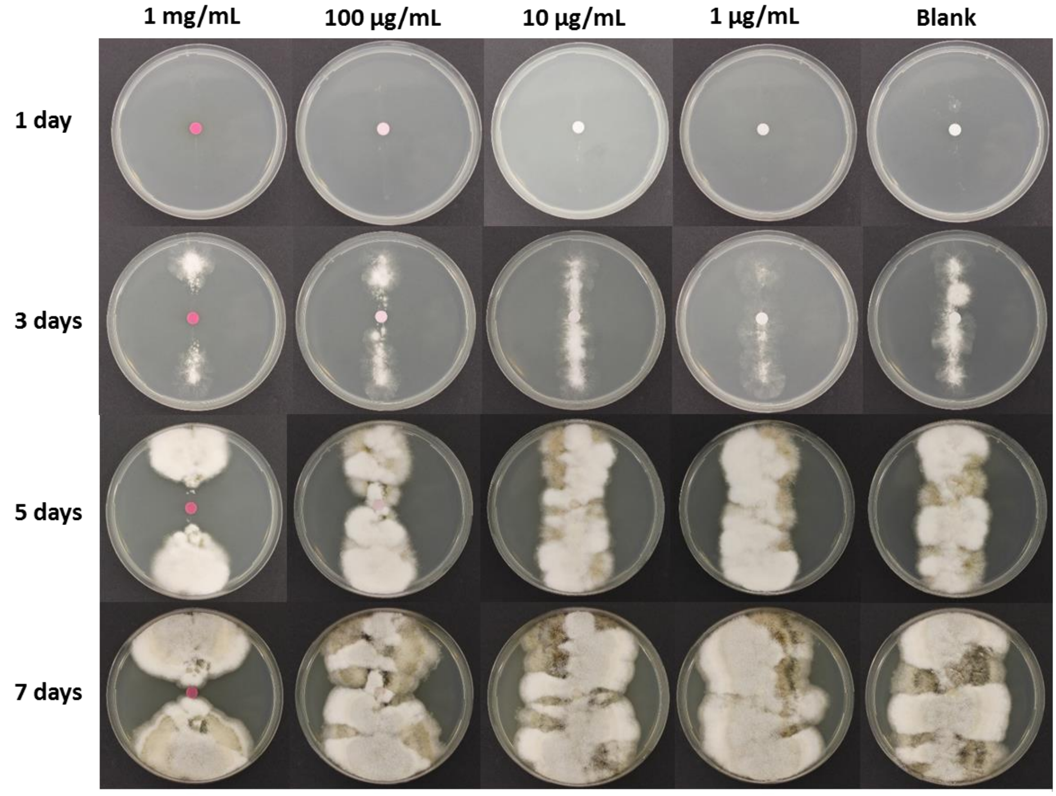

Fig. S203. Dose and time dependent Y-shape disc assay of different prodigiosin concentrations against *P. virgatula*

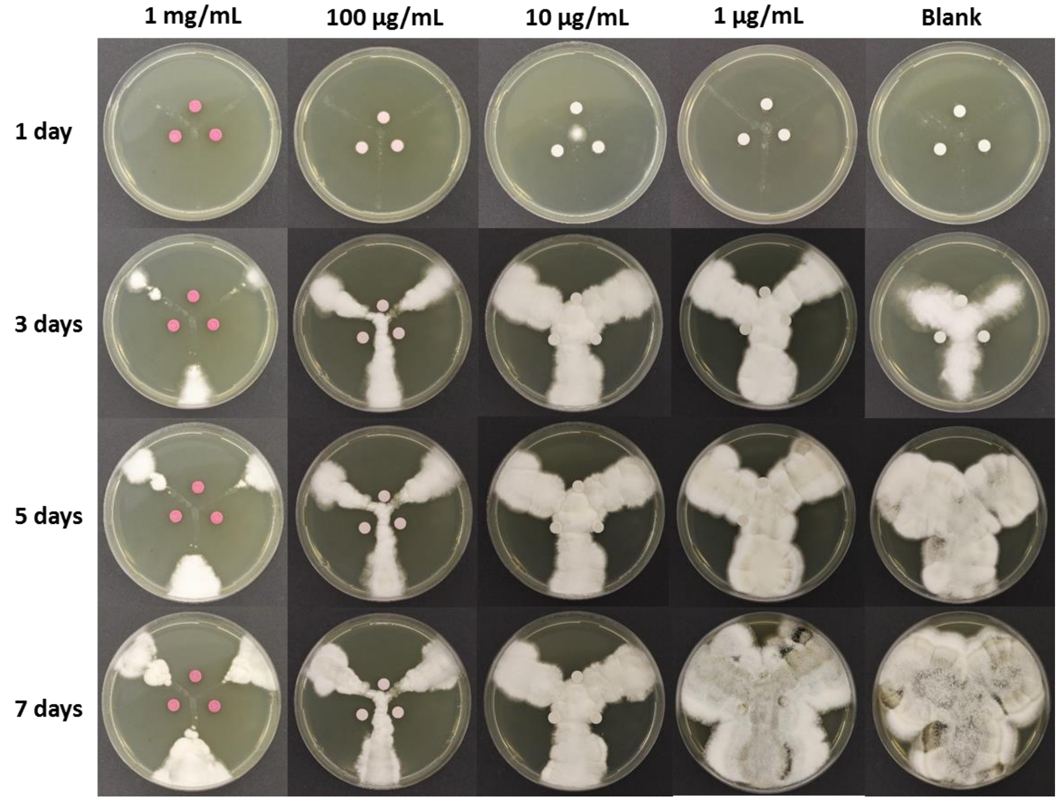

Fig. S204. *S. marcescens* MSRBB2 on chitinase detection agar incubated at 30 °C and monitored for 14 days. Chitinase detection media[1] (A) showing degradation of chitin by chitinase enzymes resulting in pH change and color change of Bromocresol purple dye. Chitinase detection media with supplemented glucose (20 g/L, B) showing no chitinase enzyme expression. Glucose is inhibiting chitinase expression.

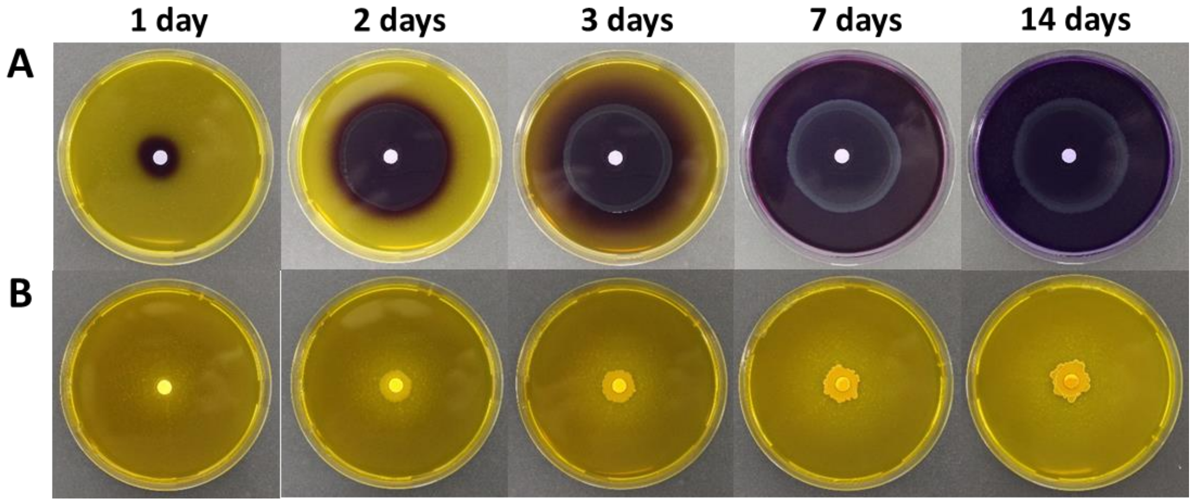

Fig. S205. Digital microscope images with different magnifications of the colonization of endophytic *A. caesiellus* by *S. marcescens* MSRBB2. Hyphae of the fungus is overgrown with *S. marcescens* MSRBB2 colonies.

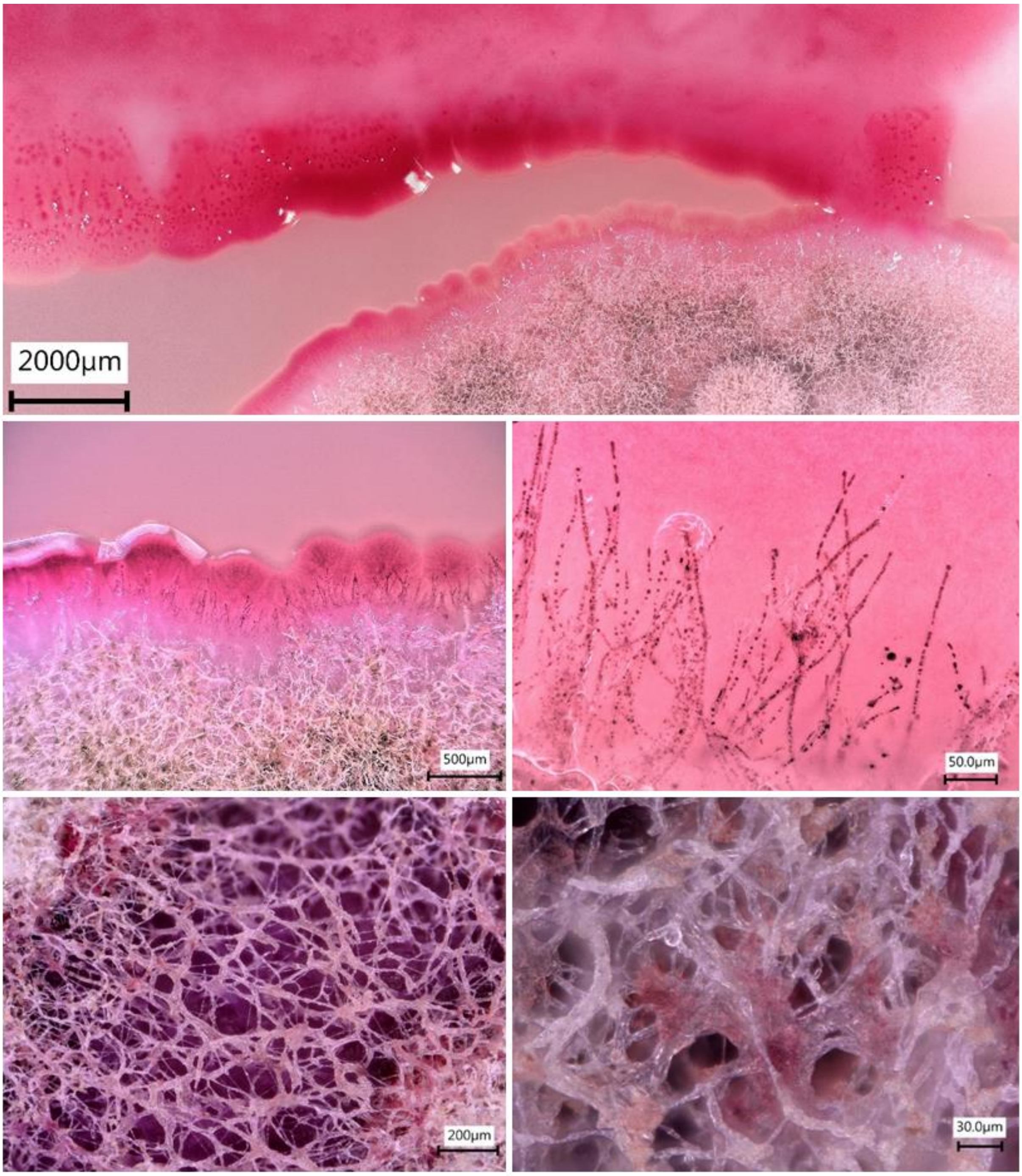

Fig. S206. MALDI-imaging-HRMS of the co-cultivation of bacterial endophyte *S. marcescens* MSRBB2 and fungal endophyte *A. caesiellus* after 3 days. Optical image with assigned scan area and localization of prodiginines ($[M+H]^+$ ± 2 ppm, (A)). Different fragments of MALDI-imaging-HRMS^2 of prodigiosin (B).

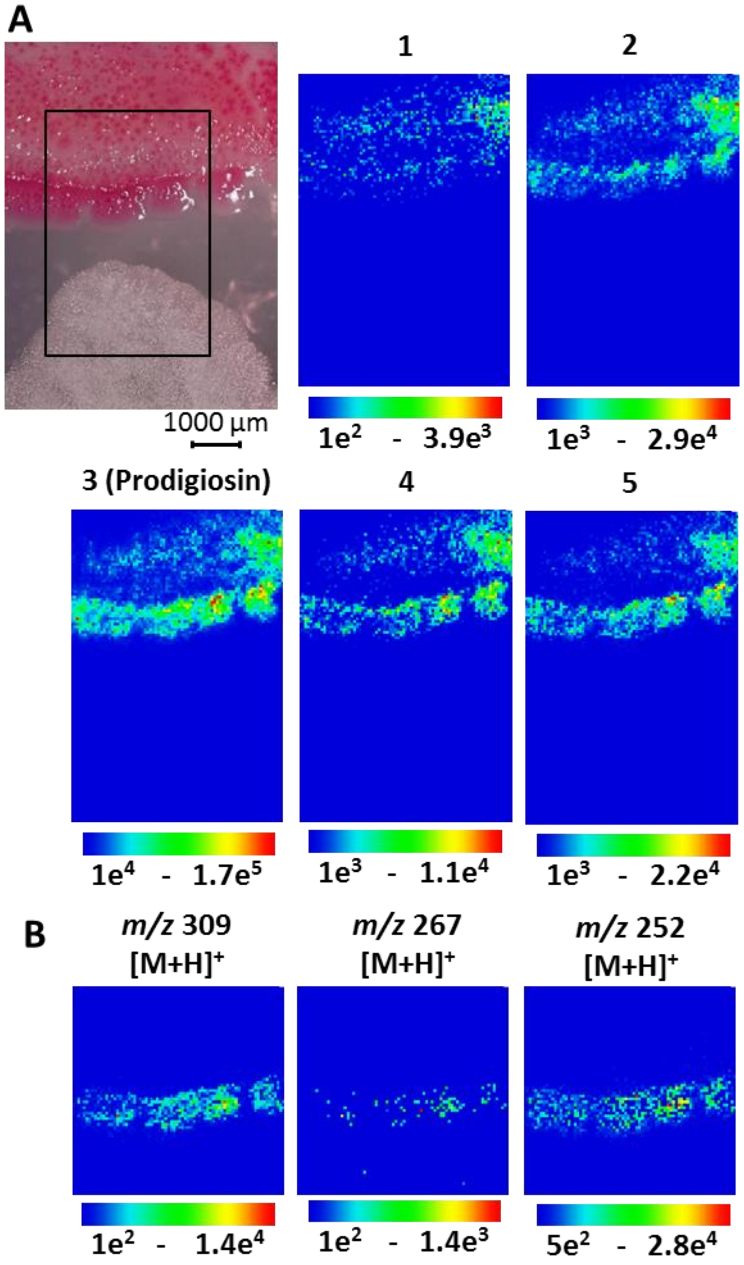

Fig. S207. MALDI-imaging-HRMS of the co-cultivation of bacterial endophyte *S. marcescens* MSRBB2 and fungal endophyte *A. caesiellus* after 3 days. Optical image with assigned scan area and localization of prodiginines ($[M+H]^+ \pm 2$ ppm).

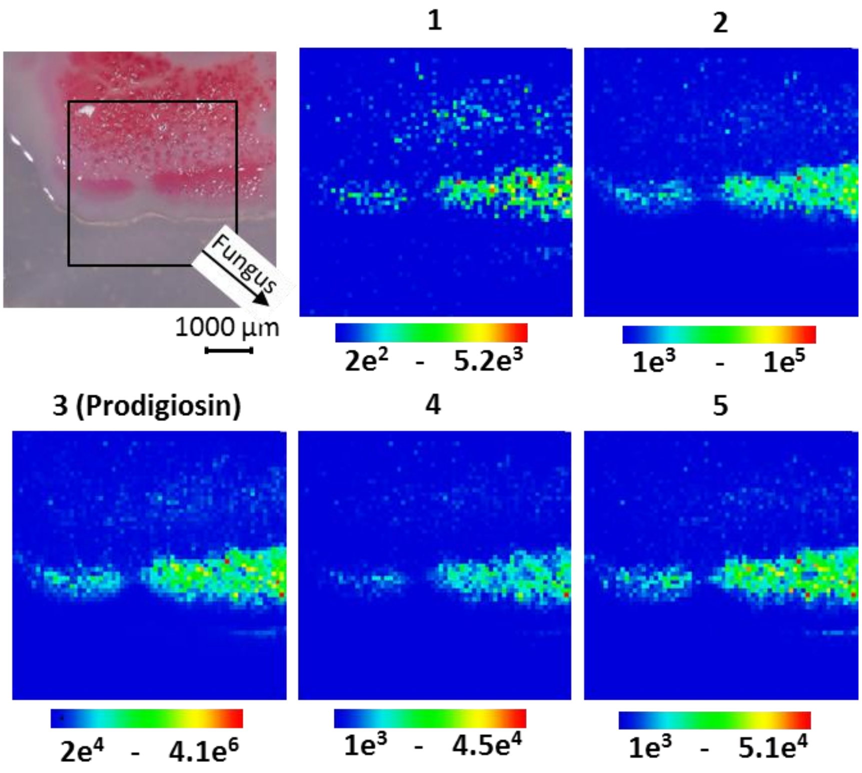

Fig. S208. MALDI-imaging-HRMS of the co-cultivation of bacterial endophyte *S. marcescens* MSRBB2 and fungal endophyte *A. caesiellus* after 5 days. Optical image with assigned scan area and localization of prodiginines ($[M+H]^+ \pm 2$ ppm).

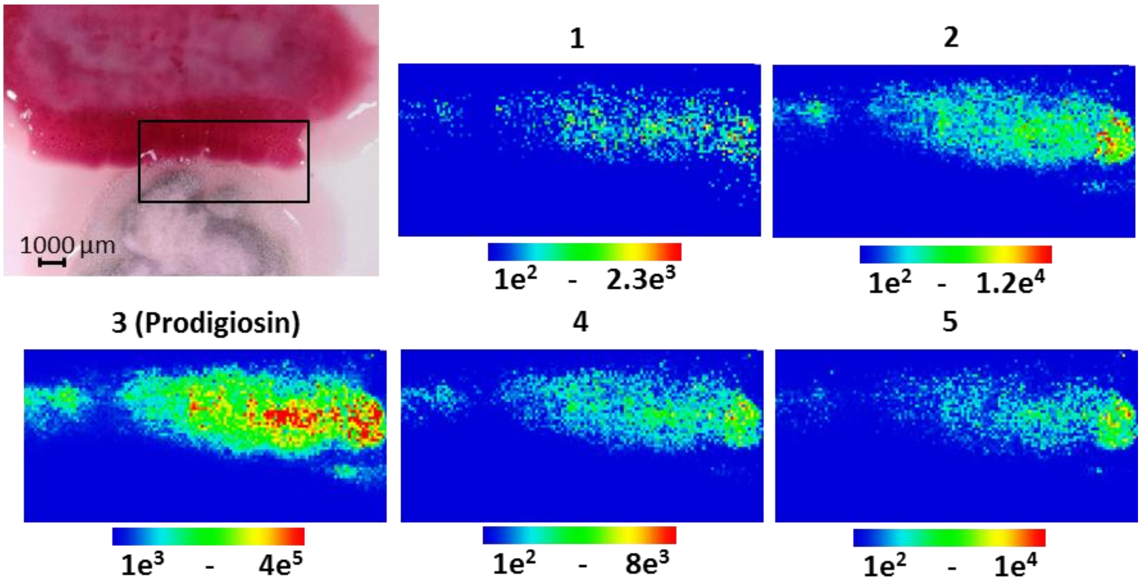

Fig. S209. MALDI-imaging-HRMS of the co-cultivation of bacterial endophyte *S. marcescens* MSRBB2 and fungal endophyte *A. caesiellus* after 7 days. Optical image with assigned scan area and localization of prodiginines ($[M+H]^+$ ± 2 ppm, (A)). Different fragments of MALDI-imaging-$HRMS^2$ of prodigiosin (B).

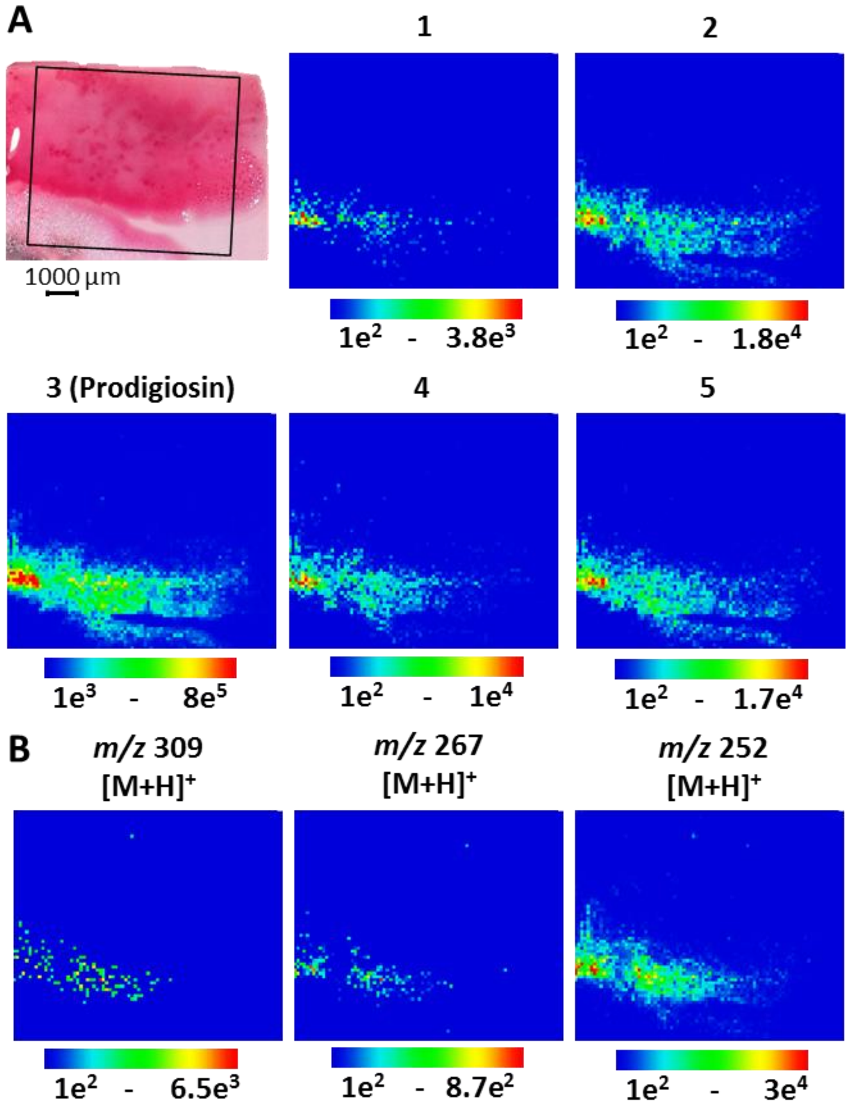

Fig. S210. MALDI-imaging-HRMS of the co-cultivation of bacterial endophyte *S. marcescens* MSRBB2 and fungal endophyte *A. caesiellus* after 7 days. Optical image with assigned scan area and localization of prodiginines ($[M+H]^+$ ± 2 ppm, (A)). Different fragments of MALDI-imaging-HRMS^2 of prodigiosin (B).

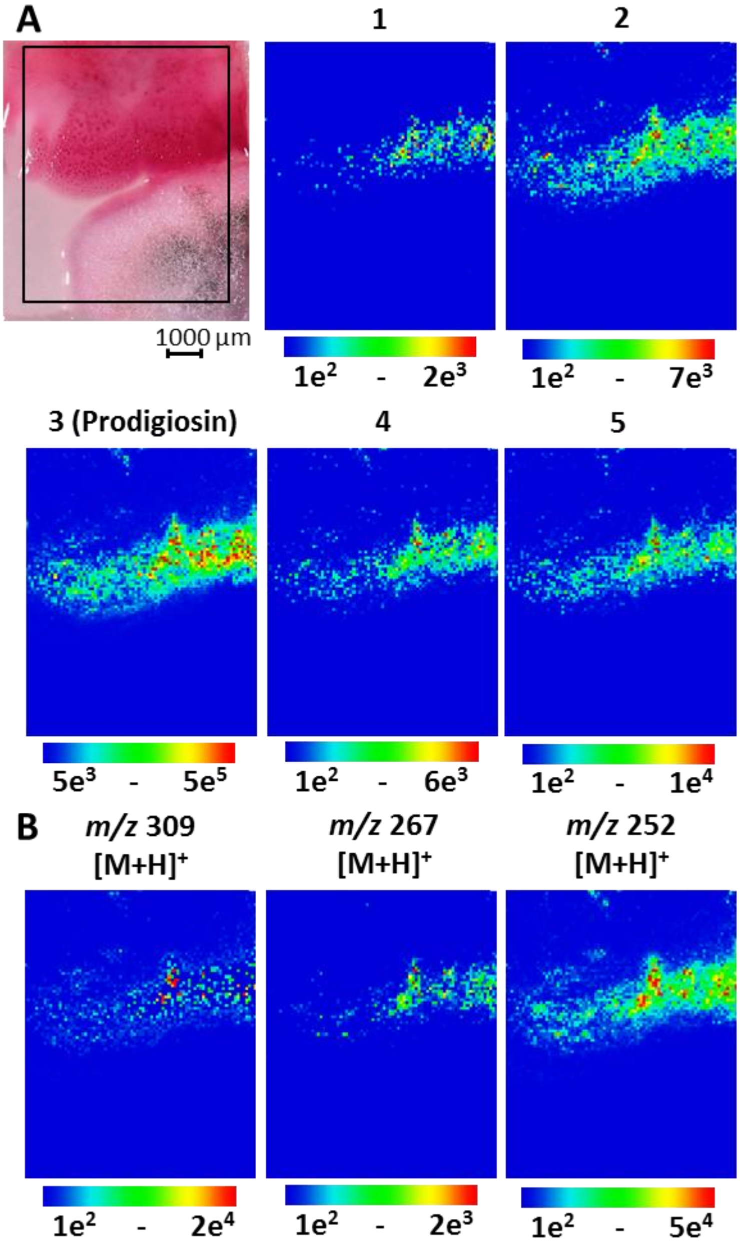

Fig. S211. Images of solo cultivation of endophytic *S. marcescens* MSRBB2 (Control).

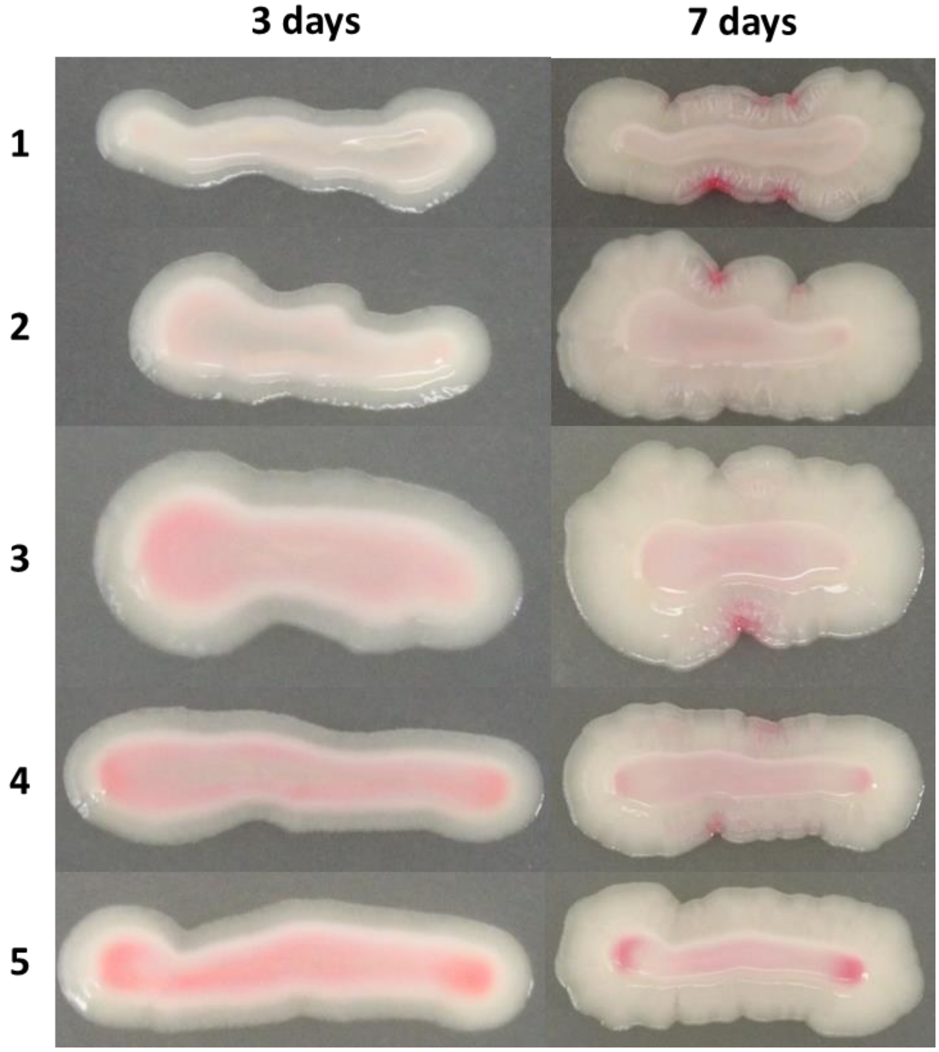

Fig. S212. MALDI-imaging-HRMS of solo cultivation of bacterial endophyte *S. marcescens* MSRBB2 after 7 days. Optical image with assigned scan area and localization of prodiginines **1-5** ([M+H]+ ± 2 ppm).

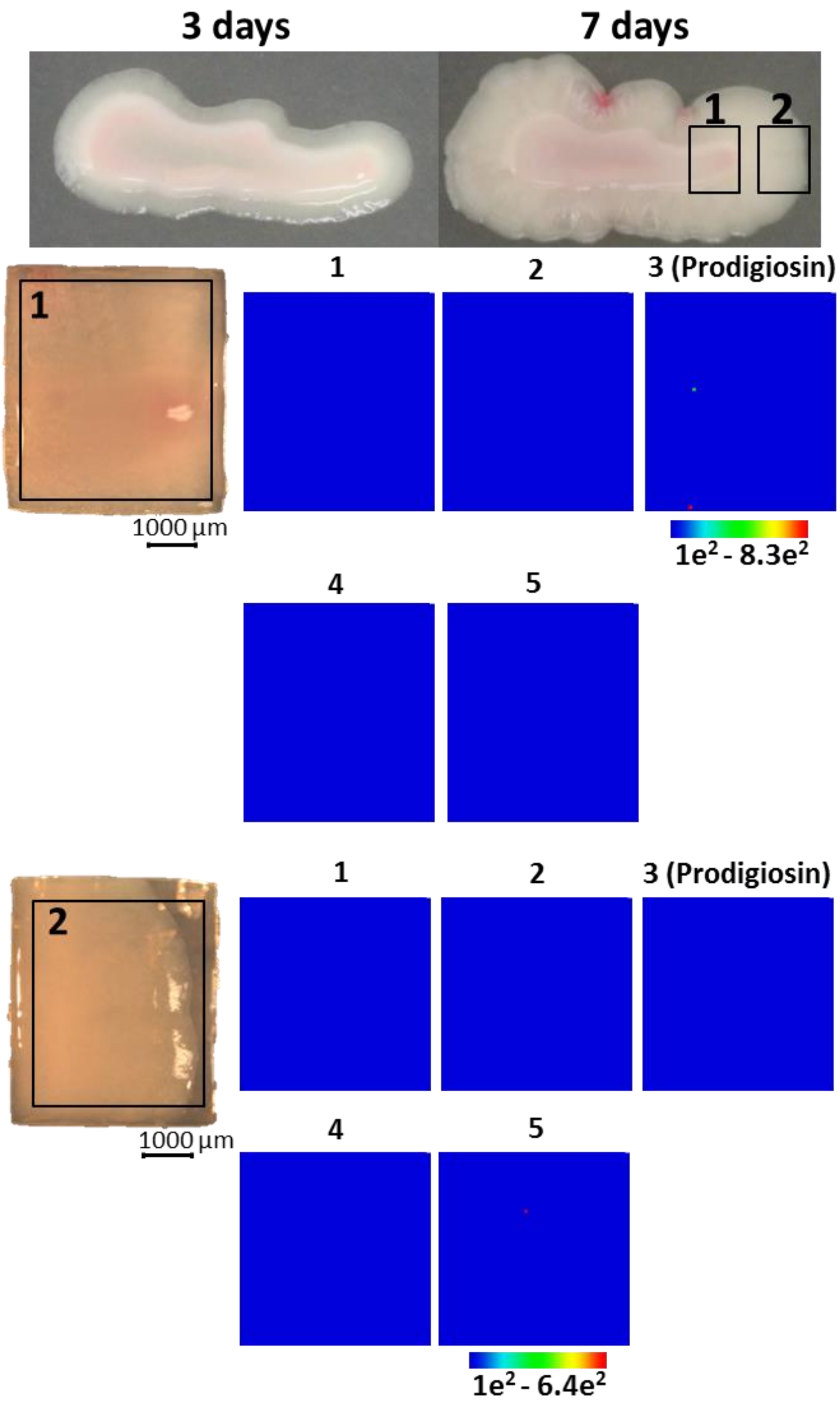

Fig. S213. HPLC-HRMS comparison of prodigiosin (Extracted ion chromatograms, $[M+H]^+ \pm$ 2 ppm) production by endophytic *S. marcescens* MSRBB2 of mono-cultivation and co-cultivations with fungal endophytes *A. caesiellus* and *Pichia* spp. (five Petri dishes) after 7 days incubation (30°C).

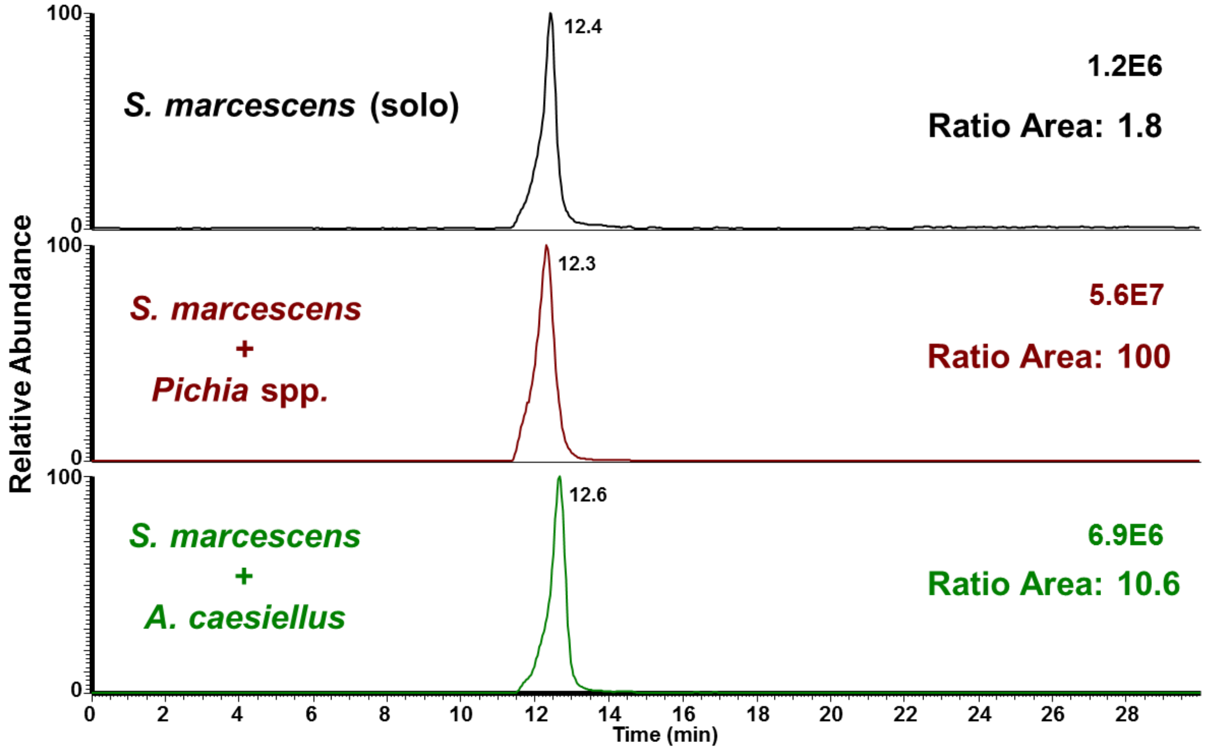

Fig. S214. HPLC-HRMS of extracts of bacterial endophyte *S. marcescens* MSRBB2 incubated at 30°C and 37°C for 24h, optical images of incubated nutrient agar Petri dishes, and extracted ion chromatograms of serrawettin w1 ($[M+H]^+$ ± 2 ppm) (A) Enlarged microscopic images (B).

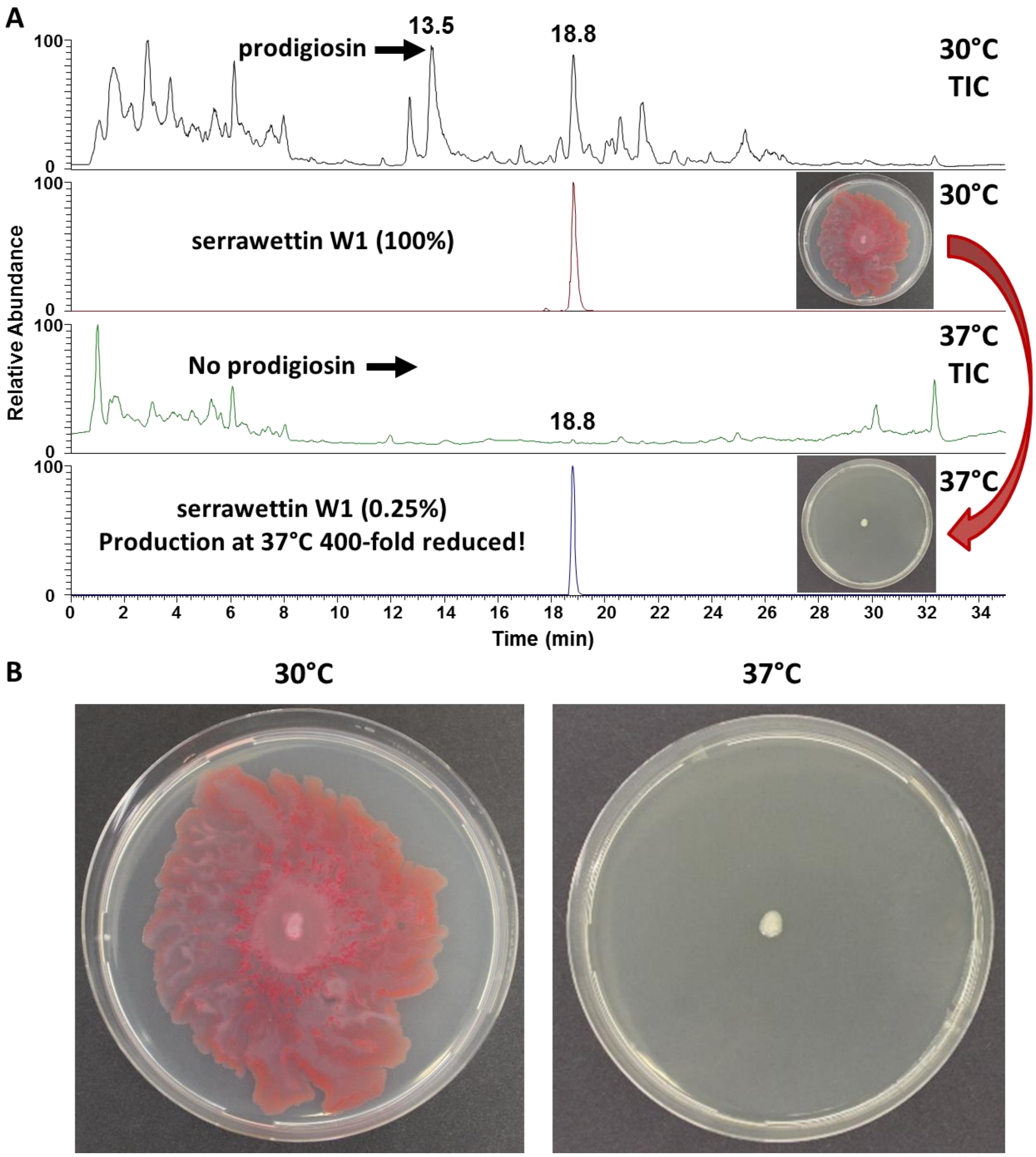

Fig. S215. MALDI-imaging-HRMS of the co-cultivation of bacterial endophyte *S. marcescens* MSRBB2 and fungal endophyte *A. caesiellus* after 3 days. Optical image with assigned scan area and localization of serratamolides ($[M+K]^+ \pm 2$ ppm).

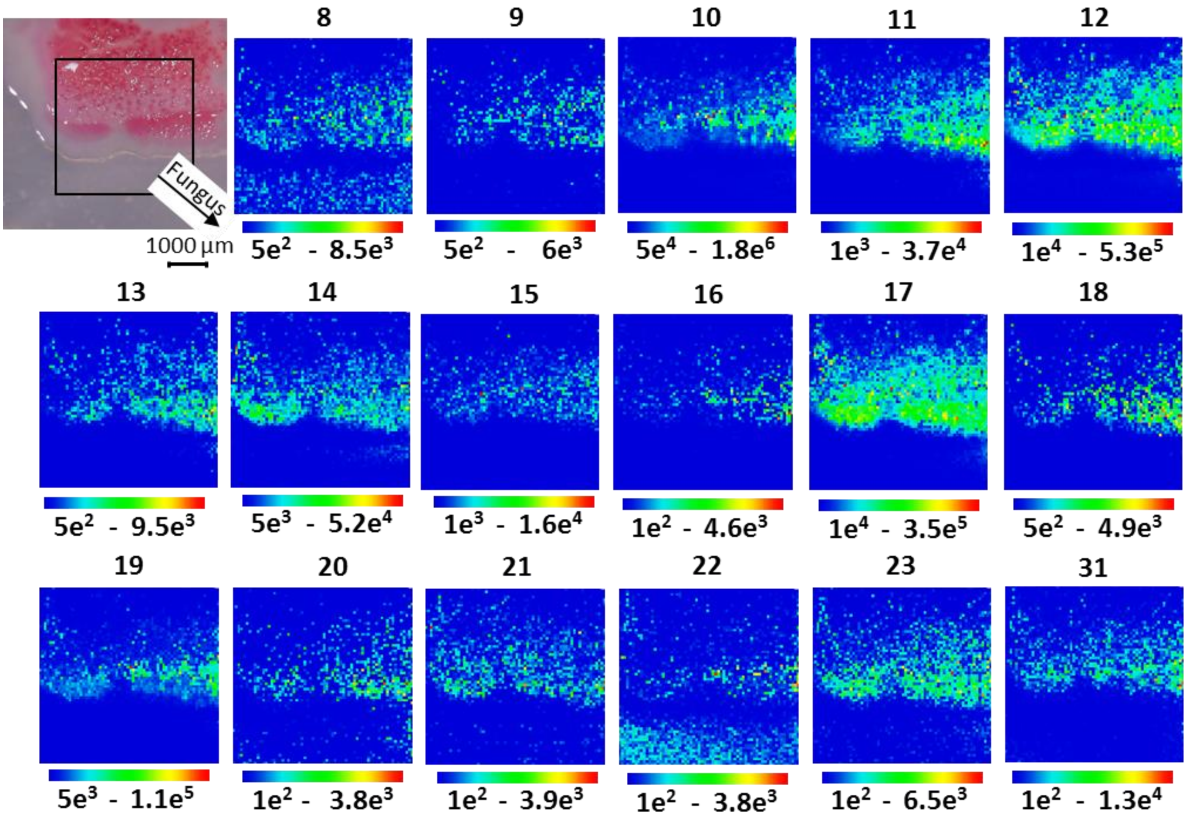

Fig. S216. MALDI-imaging-HRMS of the co-cultivation of bacterial endophyte *S. marcescens* MSRBB2 and fungal endophyte *A. caesiellus* after 5 days. Optical image with assigned scan area and localization of serratamolides ($[M+K]^+ \pm 2$ ppm).

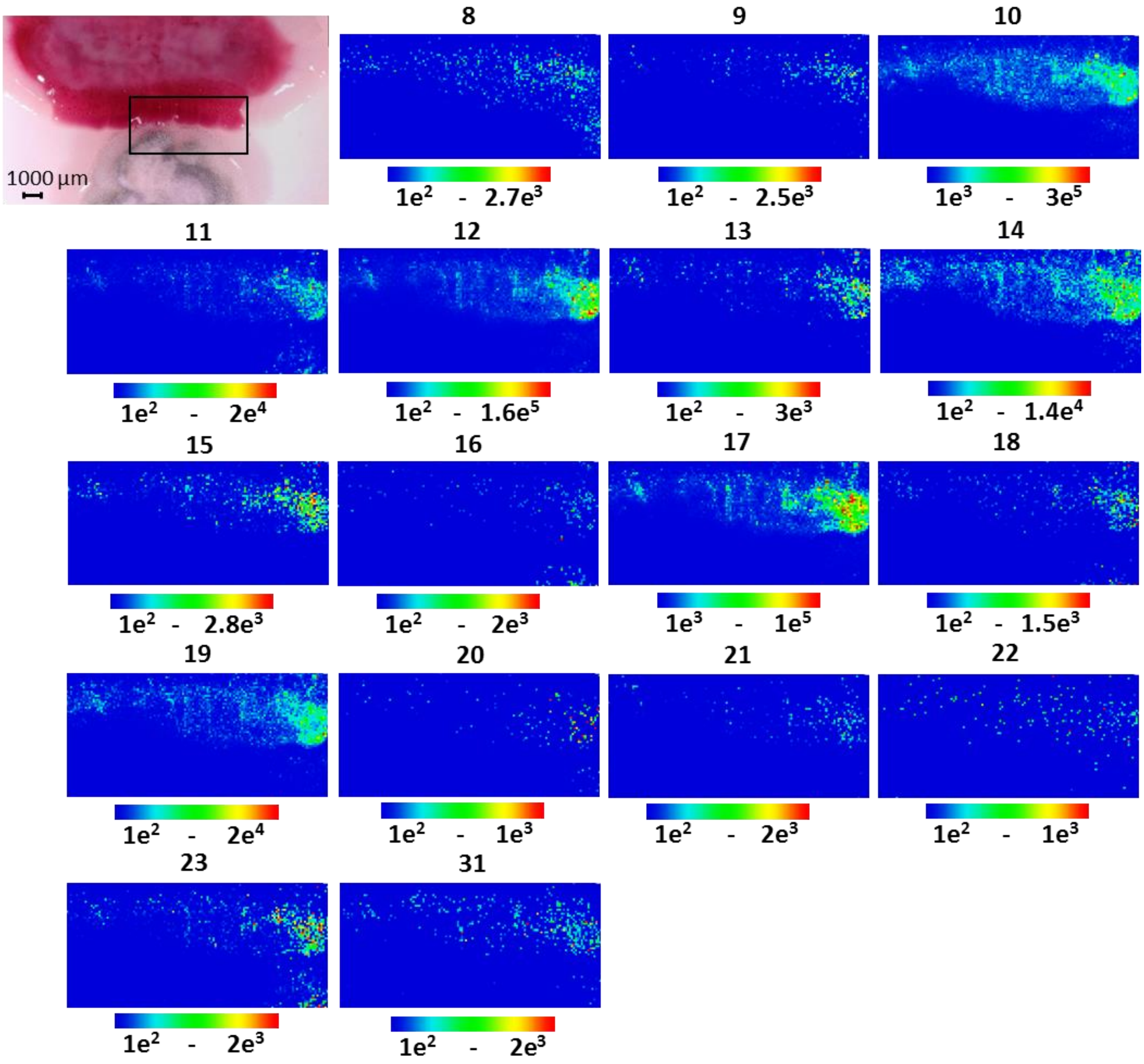

Fig. S217. MALDI-imaging-HRMS of the co-cultivation of bacterial endophyte *S. marcescens* MSRBB2 and fungal endophyte *A. caesiellus* after 7 days. Optical image with assigned scan area and localization of serratamolides ($[M+K]^+ \pm 2$ ppm).

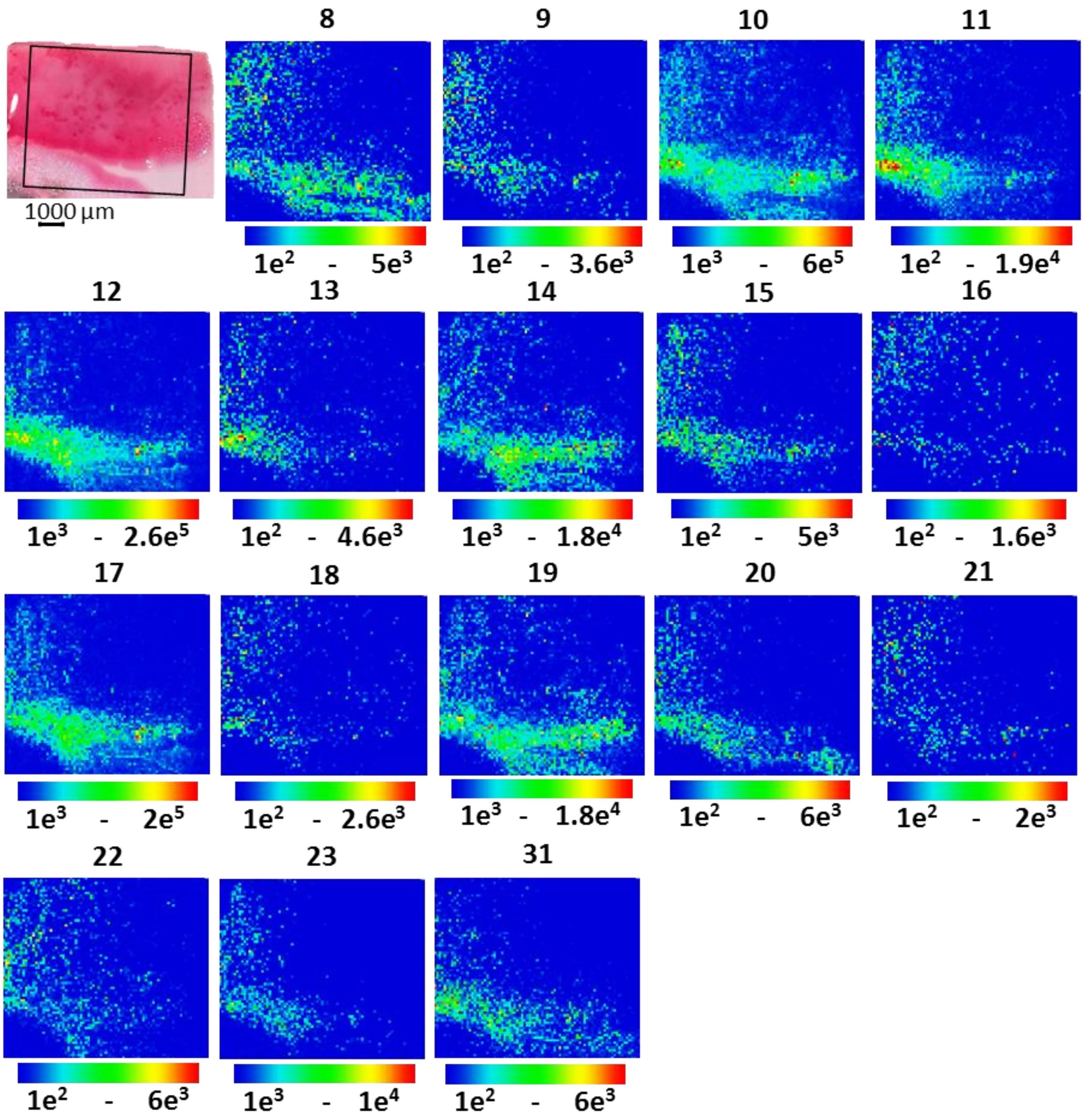

Fig. S218. MALDI-imaging-HRMS of the co-cultivation of bacterial endophyte *S. marcescens* MSRBB2 and fungal endophyte *A. caesiellus* after 7 days. Optical image with assigned scan area and localization of serratamolides ($[M+K]^+ \pm 2$ ppm).

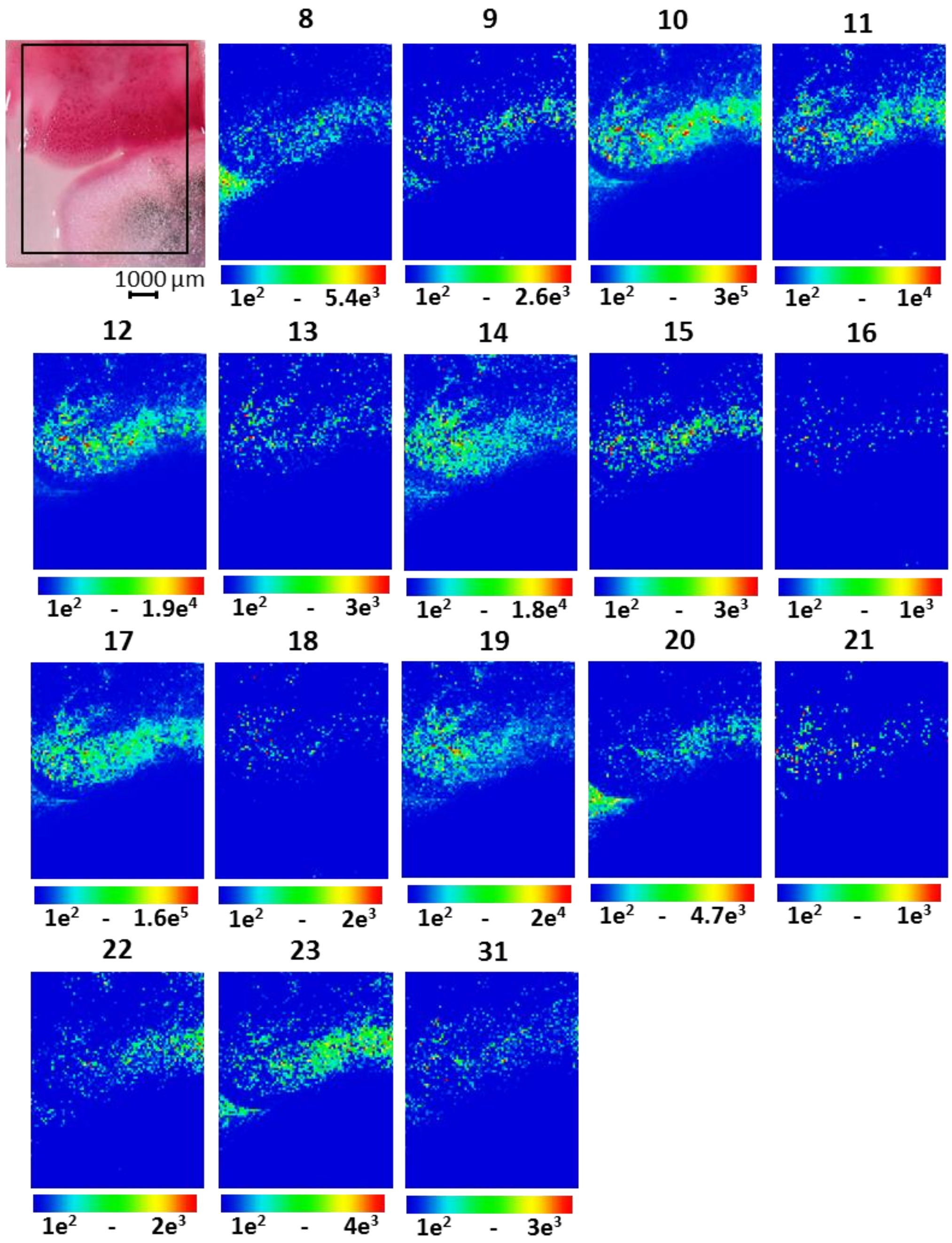

Fig. S219. MALDI-imaging-HRMS of solo cultivation of bacterial endophyte *S. marcescens* MSRBB2 after 7 days. Optical image with assigned scan area and localization of serratamolides ($[M+K]^+$ ± 2 ppm).

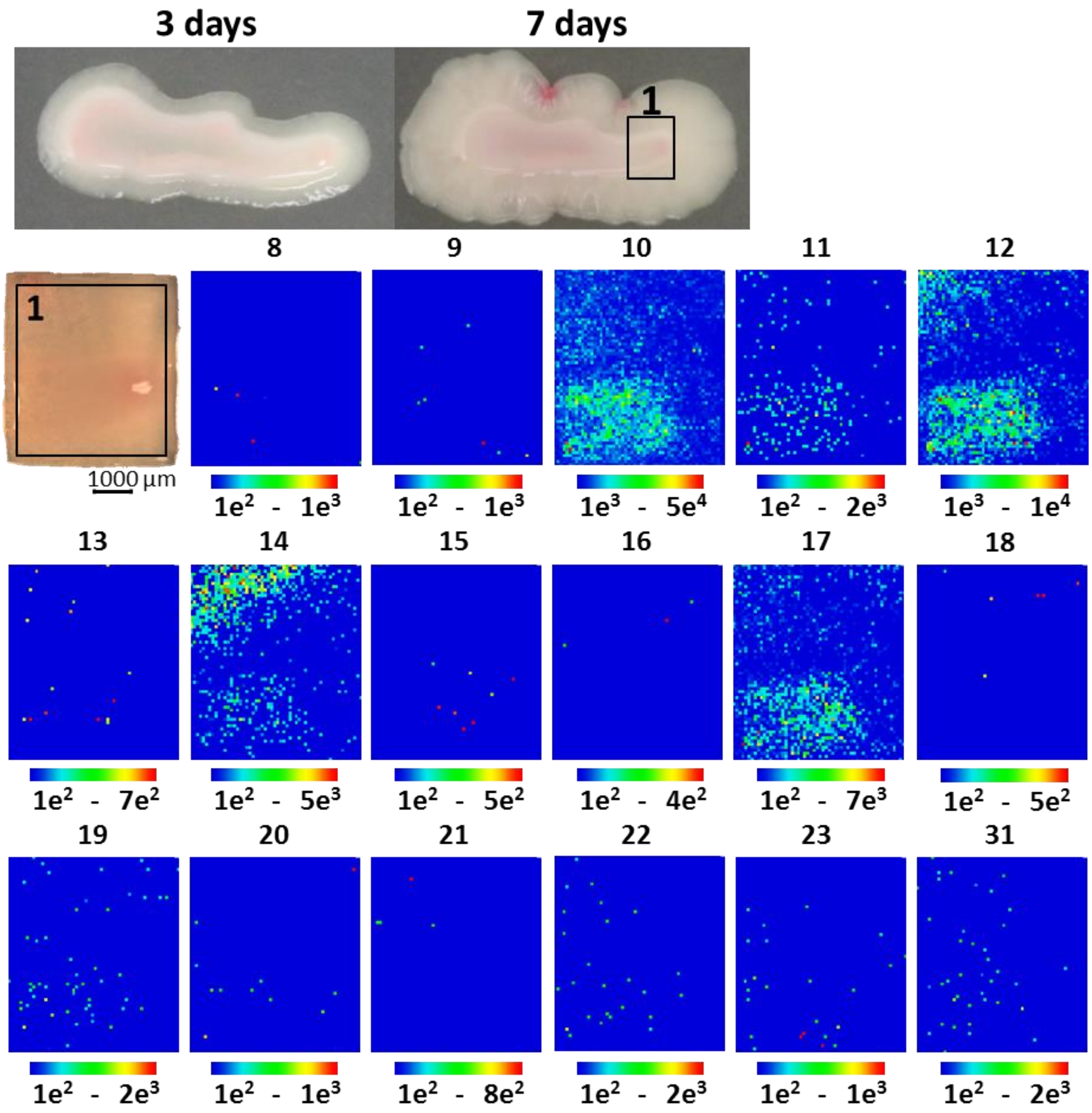

Fig. S220. MALDI-imaging-HRMS of solo cultivation of bacterial endophyte *S. marcescens* MSRBB2 after 7 days. Optical image with assigned scan area and localization of serratamolides ($[M+K]^+$ ± 2 ppm).

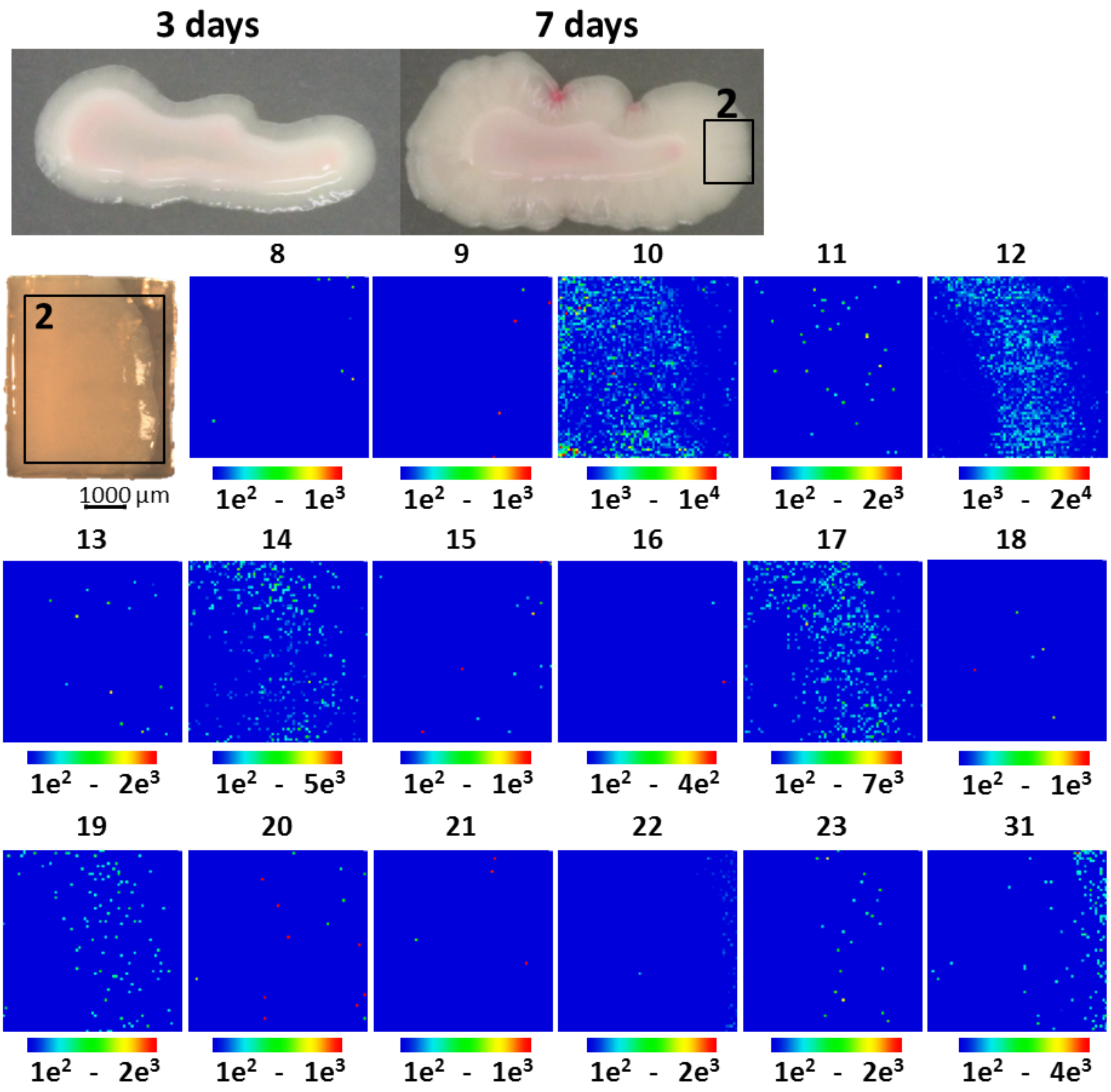

Fig. S221. Possible decomposition of serratamolides by hydrolysis resulting in open-ring serratamolides and/ or "monomers".

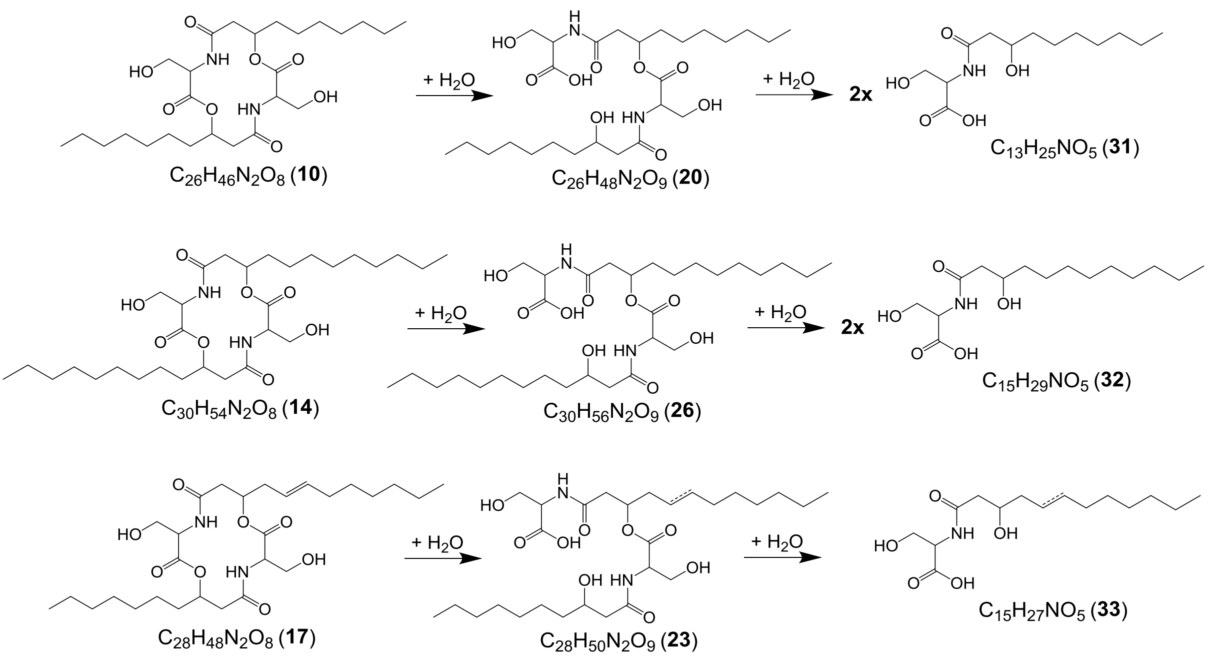

Fig. S222. Digital microscope images of leaf puncture bioassay of *M. canariensis*, *M. heterophylla*, and *M. senegalensis* against prodigiosin (B) and serrawettin W1 (C) after 6 days (Scale bar = 500 µm; Blank (A).

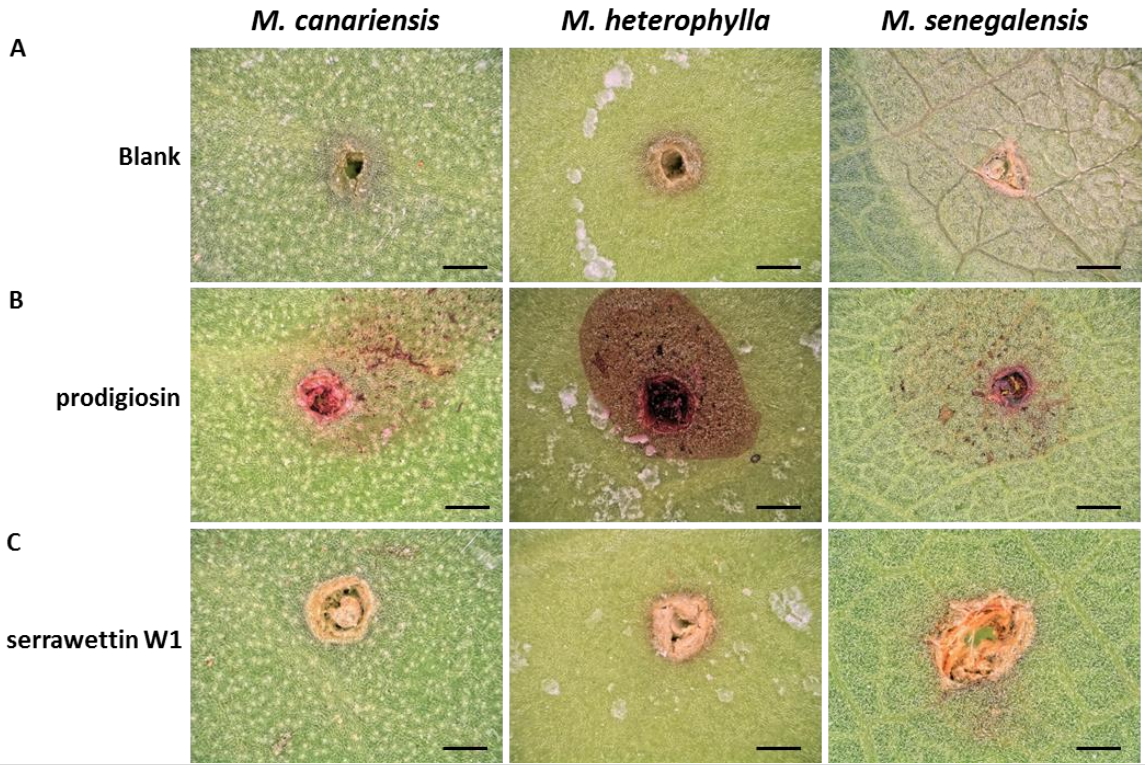

Fig. S223. Isolation of endophytic *S. marcescens* MSRBB2 from *M. serrata* stem. Primary isolation plate (A) along with magnification (C) and spread axenic isolate (B).

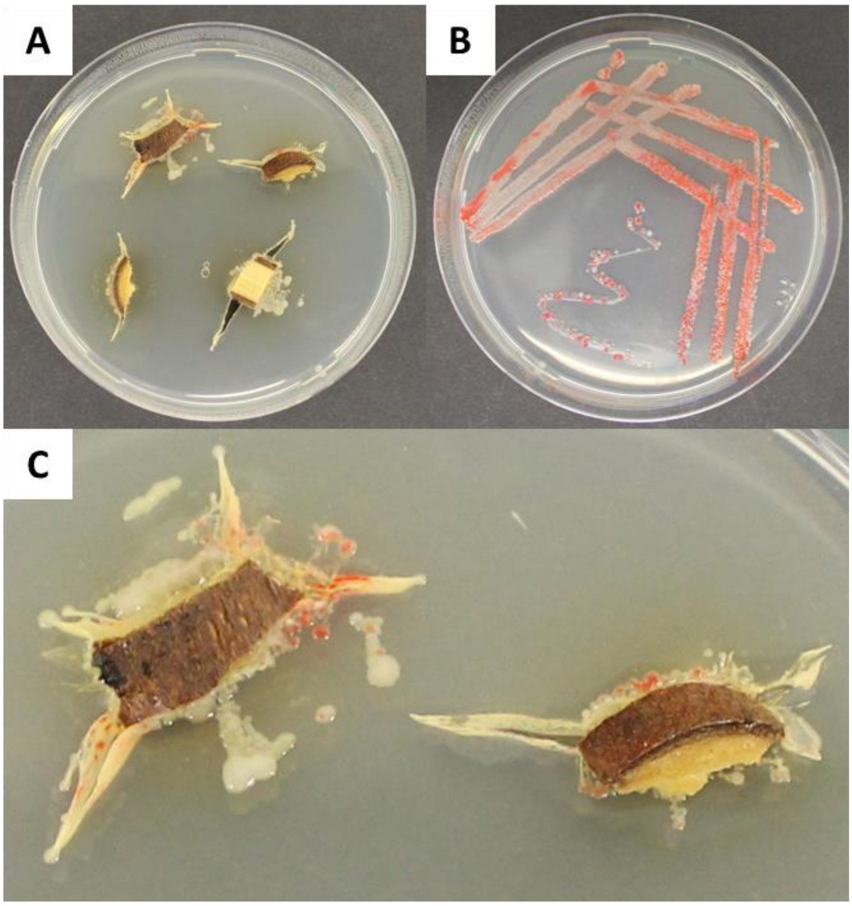

Fig. S224. Microscopic images of endophytic fungi *Pichia* spp. (A), *A. caesiellus* (B), and *P. virgatula* (C) isolated from *M. serrata*.

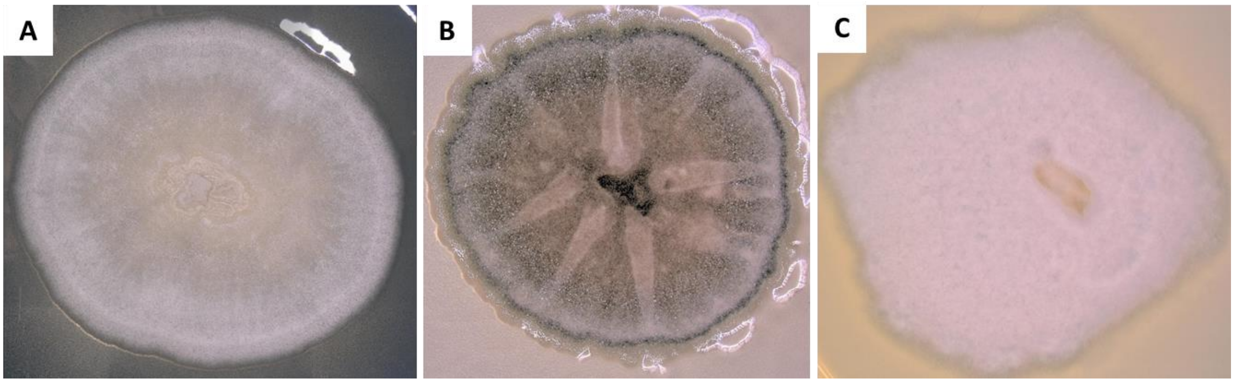

II. SUPPLEMENTARY TABLES

Table S1. Peak height ratios from high resolution mass spectrometry of serratamolides labeled with [1,2-$^{13}C_2$]-sodium acetate (n.d. = not detected; * = observed peak height in % of M+4).

	Sum formula	Observed peak height (% of M)											
**No.**	**[M+H]**	**M+2**	**M+4**	**M+6**	**M+8**	**M+10**	**M+12**	**M+14**	**M+16**	**M+18**	**M+20**	**M+22**	**M+24**
**8**	$C_{24}H_{43}N_2O_8$	n.d.	100*	139	228	127	n.d.	n.d.	n.d.	n.d.	n.d.	n.d.	n.d.
**9**	$C_{25}H_{45}N_2O_8$	248	648	1139	857	423	115	n.d.	n.d.	n.d.	n.d.	n.d.	n.d.
**10**	$C_{26}H_{47}N_2O_8$	140	334	603	783	763	554	295	118	31	3	n.d.	n.d.
**11**	$C_{27}H_{49}N_2O_8$	323	634	1077	1090	891	466	198	n.d.	n.d.	n.d.	n.d.	n.d.
**12**	$C_{28}H_{51}N_2O_8$	80	205	390	556	606	518	344	176	65	16	1	n.d.
**13**	$C_{29}H_{53}N_2O_8$	111	139	616	737	678	344	168	30	n.d.	n.d.	n.d.	n.d.
**14**	$C_{30}H_{55}N_2O_8$	33	88	215	312	386	363	285	171	82	27	1	n.d.
**15**	$C_{26}H_{45}N_2O_8$	n.d.	n.d.	n.d.	n.d.	n.d.	n.d.	n.d.	n.d.	n.d.	n.d.	n.d.	n.d.
**16**	$C_{27}H_{47}N_2O_8$	n.d.	n.d.	n.d.	n.d.	n.d.	n.d.	n.d.	n.d.	n.d.	n.d.	n.d.	n.d.
**17**	$C_{28}H_{49}N_2O_8$	116	324	631	889	984	848	571	294	109	20	1	n.d.
**18**	$C_{29}H_{51}N_2O_8$	93	80	100	241	218	n.d.	n.d.	n.d.	n.d.	n.d.	n.d.	n.d.
**19**	$C_{30}H_{53}N_2O_8$	37	151	323	504	591	599	477	303	126	31	3	4
**20**	$C_{26}H_{49}N_2O_9$	113	279	491	647	631	474	265	108	27	4	n.d.	n.d.
**21**	$C_{27}H_{51}N_2O_9$	161	474	865	1086	1040	776	416	163	45	5	1	n.d.
**22**	$C_{28}H_{53}N_2O_9$	76	205	369	524	573	481	329	165	62	16	1	n.d.
**23**	$C_{28}H_{51}N_2O_9$	81	217	414	602	686	595	409	209	76	5	1	n.d.
**24**	$C_{25}H_{47}N_2O_9$	448	736	1132	1206	773	129	n.d.	n.d.	n.d.	n.d.	n.d.	n.d.
**25**	$C_{29}H_{55}N_2O_9$	343	718	983	1067	924	597	318	118	25	1	n.d.	n.d.
**26**	$C_{30}H_{57}N_2O_9$	43	117	233	333	404	400	314	189	89	30	2	n.d.
**27**	$C_{31}H_{59}N_2O_9$	142	978	1805	3971	4689	4343	3218	2286	246	54	n.d.	n.d.
**28**	$C_{29}H_{53}N_2O_9$	209	559	1173	1634	1847	1561	1034	577	198	54	5	n.d.
**29**	$C_{30}H_{55}N_2O_9$	34	98	197	327	383	367	307	188	90	27	6	n.d.
**30**	$C_{31}H_{57}N_2O_9$	50	183	668	1099	1381	1429	1112	610	305	16	n.d.	n.d.
**31**	$C_{13}H_{26}NO_5$	207	328	273	115	21	n.d.	-	-	-	-	-	-
**32**	$C_{15}H_{30}NO_5$	166	287	295	189	80	13	n.d.	-	-	-	-	-
**33**	$C_{15}H_{28}NO_5$	170	329	365	245	96	15	n.d.	-	-	-	-	-

III. REFERENCES

[1] Agrawal, T. & Kotasthane, A. S. Chitinolytic assay of indigenous *Trichoderma* isolates collected from different geographical locations of Chhattisgarh in Central India. *SpringerPlus* **1**:73 (2012).
